# Updating the therapeutic role of ginsenosides in breast cancer: a bibliometrics study to an in-depth review

**DOI:** 10.3389/fphar.2023.1226629

**Published:** 2023-09-25

**Authors:** Xianguang Deng, Juan Wang, Chenyi Lu, Yao Zhou, Lele Shen, Anqi Ge, Hongqiao Fan, Lifang Liu

**Affiliations:** Department of Galactophore, The First Hospital of Hunan University of Chinese Medicine, Changsha, Hunan, China

**Keywords:** ginsenosides, breast cancer, bibliometrics, ginseng, review

## Abstract

Breast cancer is currently the most common malignancy and has a high mortality rate. Ginsenosides, the primary bioactive constituents of ginseng, have been shown to be highly effective against breast cancer both *in vitro* and *in vivo*. This study aims to comprehensively understand the mechanisms underlying the antineoplastic effects of ginsenosides on breast cancer. Through meticulous bibliometric analysis and an exhaustive review of pertinent research, we explore and summarize the mechanism of action of ginsenosides in treating breast cancer, including inducing apoptosis, autophagy, inhibiting epithelial-mesenchymal transition and metastasis, and regulating miRNA and lncRNA. This scholarly endeavor not only provides novel prospects for the application of ginsenosides in the treatment of breast cancer but also suggests future research directions for researchers.

## Graphical Abstract

**Figure:**
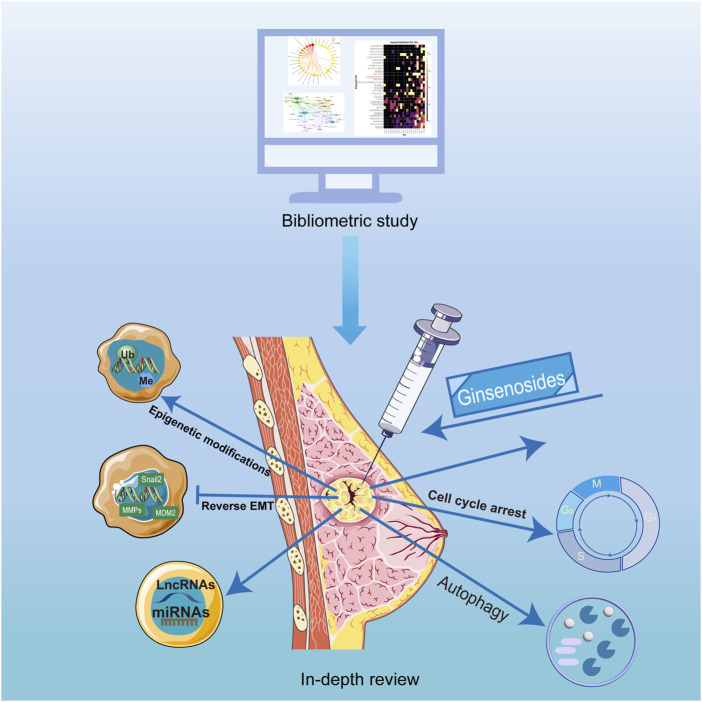

## 1 Introduction

Breast cancer stands as the most prevalent neoplastic affliction globally, with higher rates in developed countries than in developing countries (Sung et al., 2021). Notably, the growth velocity in developing nations outpaces that of developed regions, a trend anticipated to persist (Lim et al., 2022). Compounding this issue, the incidence of breast cancer is increasing annually, and the affected demographic is increasingly skewed toward the younger populace (Fitzmaurice and Global Burden of Disease Cancer Collaboration, 2018). This has resulted in a huge public health burden as well as a huge physical and psychological toll on patients with breast cancer. With the advancement of medicine, not only surgery but also many other treatments have been applied to breast cancer, such as chemotherapy, targeted therapy, and endocrine therapy. These treatments have improved disease-free survival (DFS) and overall survival (OS) of breast cancer patients to some extent. Nevertheless, there are several adverse effects associated with these treatments, and not all breast cancer patients benefit from these treatments due to drug resistance. Especially for triple-negative breast cancer. Consequently, the survival of breast cancer patients remains a challenge. Therefore, it is necessary to develop new drugs to improve the DFS and OS of patients with breast cancer.

The pathologic process of breast cancer development broadly consists of four aspects: precancerous lesions, ductal carcinoma *in situ*, invasive breast cancer, and metastatic breast cancer. Each stage involves multiple molecular mechanisms. Pre-cancerous breast lesions are mainly characterized by pathologic morphological and structural abnormalities compared to normal breast tissue (Ellis, 2010). Ductal carcinoma *in situ* (DCIS) is the earliest stage of breast cancer in which the abnormal cells are confined to the milk ducts of the breast. The development of DCIS generally begins through hereditary or acquired mutations that affect tumor suppressor genes (e.g., BRCA1, BRCA2) or oncogenic genes (e.g., HER2/neu), which disrupt cellular regulatory control and promotes unrestricted proliferation, resistance to apoptosis is enhanced (Solin, 2019). Invasive breast cancers include invasive ductal carcinoma and invasive lobular carcinoma, which differ pathologically from DCIS in that the growth of invasive breast cancers breaks through the basement membrane by a mechanism that is mainly due to the increased invasive potential of epithelial-mesenchymal transition (EMT). And invasive breast cancer has active angiogenesis, which promotes tumor growth (Keirsse et al., 2014). Metastatic breast cancer is a coordinated process of tumor cell invasion, circulating cell survival, and distant extravasation driven by EMT, extracellular matrix degradation, and immune evasion. In addition, with modern drug interventions, breast cancers with drug resistance emerge, and the mechanisms of resistance emergence are often related to alterations in the tumor microenvironment, epigenetic changes, EMT and immune evasion (Hashemi et al., 2022).

In recent years, natural compounds in Chinese medicine have gained the attention of many researchers due to their excellent efficacy and mild side effects. Among these, ginseng, an enduring perennial herb hailing from the Araliaceae family, is known for its ability to strengthen the body and improve longevity. Ginsenosides are its principal ingredients, which are thought to have anti-inflammatory, antioxidant, and anti-tumor properties. These ginsenosides are mainly classified into four distinct categories, namely, protopanaxadiol (ginsenosides Ra1, Ra2, Ra3, etc.), protopanaxatriol (ginsenosides Re, Rf, Rg1, etc.), C17 side-chain varied (Rg5, Rk1, Rh4, etc.) and oleanolic acid (ginsenosides R0, Rh3, R1, etc.). Numerous investigations demonstrated that ginsenosides had great potential for the treatment of breast cancer (Ratan et al., 2021). And it is very necessary to summarize and analyze these researches.

Bibliometrics is a way of using powerful statistical methods to retrospectively analyze and summarize research findings, calculate data correlations, and predict the development of future research (Li et al., 2018). Bibliometrics plays an important role in summarizing breast cancer research, ginseng research, and big data medical research (Özen Çınar, 2020, 2009–2018; Zeng et al., 2022). By encapsulating the evolving landscape of scholarly output, bibliometrics lays bare prevailing research focal points while illuminating the compass for impending inquiries. Therefore, we believe that bibliometrics is an appropriate strategy for exploring research hotspots for ginsenosides in treating breast cancer. The bibliometric studies summarized current research hotspots and provided future research directions in specific areas. Therefore, we believe that bibliometrics is an appropriate strategy to summarize ginsenoside for breast cancer research as well as to discover research hotspots. However, to date, there are no bibliometric studies on ginsenosides for breast cancer in relevant articles. In this review, we first performed a bibliometric overview of ginsenosides in the treatment of breast cancer, followed by an in-depth analysis of the therapeutic effects of ginsenosides in breast cancer based on the bibliometric results.

## 2 Bibliometric research

### 2.1 Data and methods

#### 2.1.1 Data download and filtering

**FIGURE 1:**
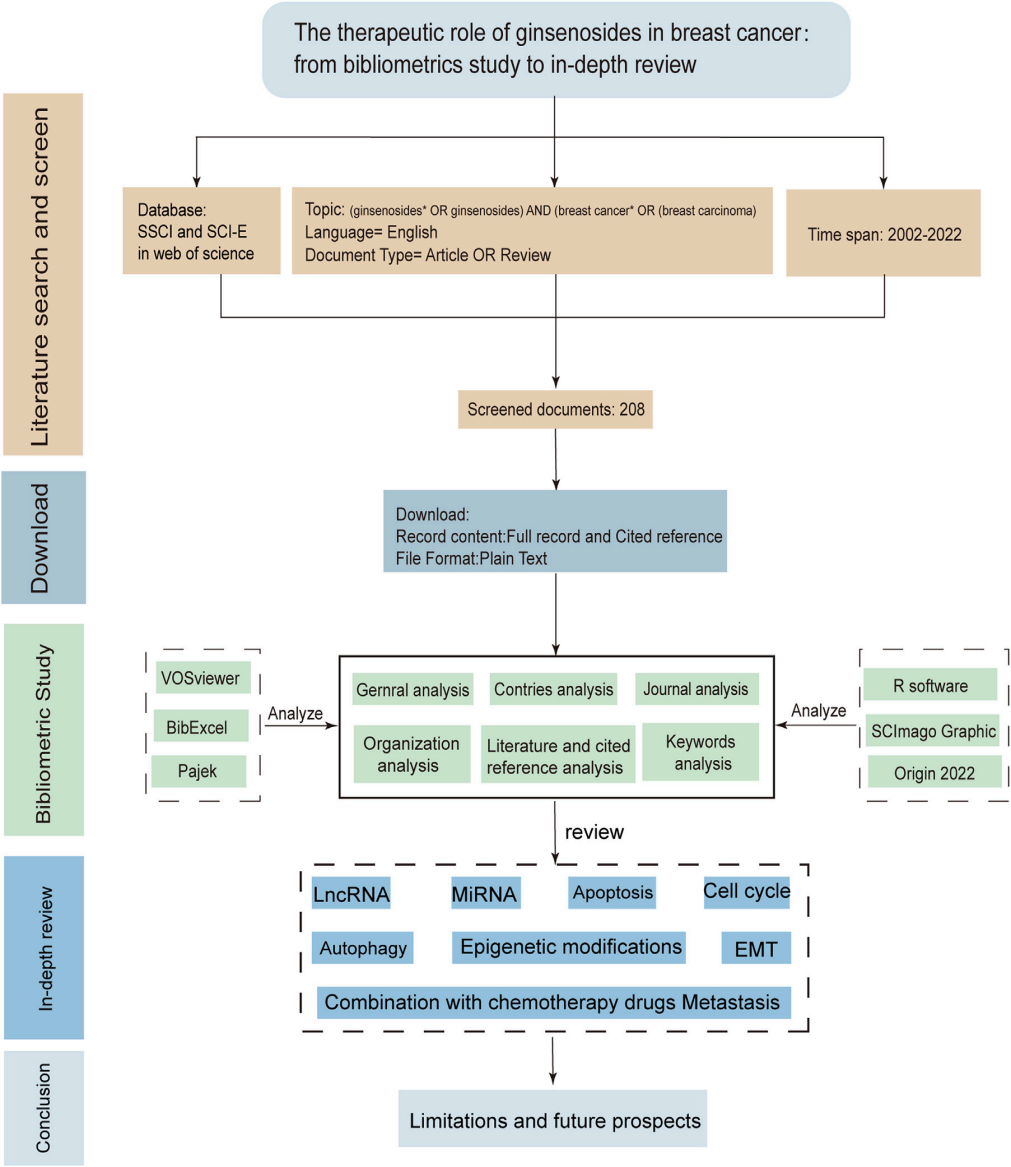
The flow chart of study of ginsenosides on breast cancer.

The dataset utilized for the bibliometric analysis was obtained from the Web of Science (WoS) Core Collection. The search strategy was devised as follows: SU= [(Ginsenosides*) OR (ginsenoside*)] AND SU = [(breast cancer*) OR (breast carcinoma*)]. The language of the publication was restricted to English, and the time frame spanned from 1 January 2002, to 31 December 2022. The record contents of data were complete records and cited references. To avoid bias due to frequent database updates, all data retrieval and collection was completed within 1 day on 5 April 2023. Details of the search strategy were provided in Supplementary Table S1. The file format was plain text. We filtered the raw data through Bibliometrix (Version 3.13) in the R language (Version 4.2.1). The flow chart of the study of the therapeutic effect of ginsenosides on breast cancer was shown in Figure 1.

#### 2.1.2 Bibliometric analysis and visualization

The main information was extracted using Bibliometrix and VOSviewer, including the annual number of publications, countries, and authors. The aforementioned variables were processed and visualized using Scimago Graphica (Version 2.0), R language, Origin 2022, and VOSviewer (version 1.6.11). For keyword clustering and co-occurrence analysis, we used Pajek, Bibexcel, and Vosviewer to visualize and analyze. The annual occurrences of keywords were visualized using R software.

### 2.2 General analysis

**FIGURE 2:**
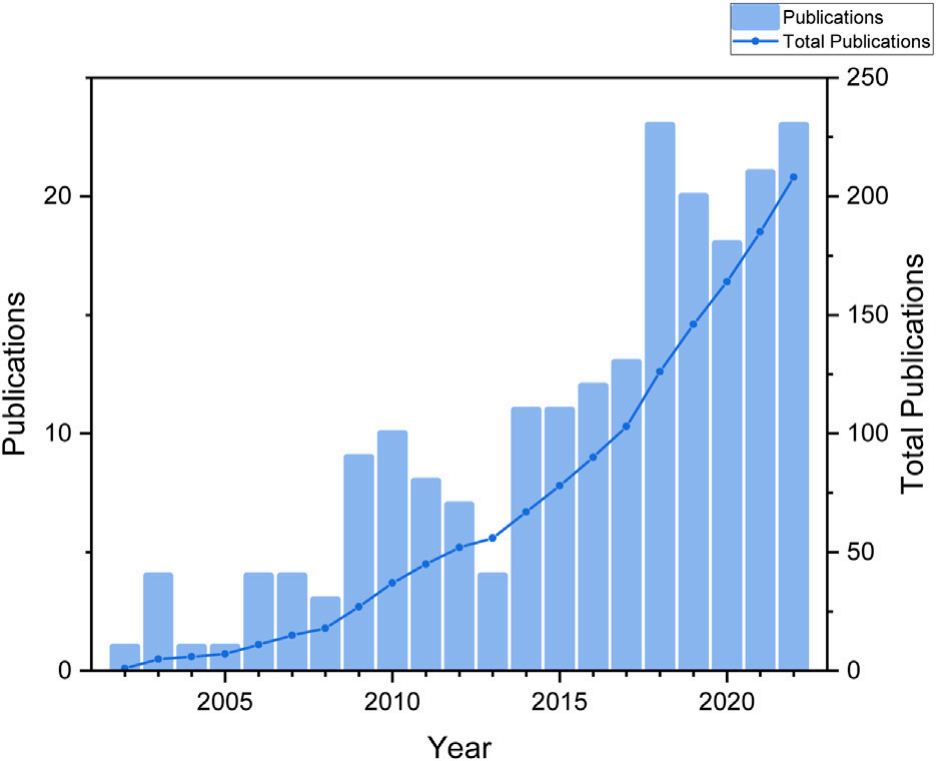
The number of publications by year.

Based on our search strategy and screening, a total of 208 publications were collected that met the requirements. These publications were written by 1,057 authors from 302 organizations in 28 countries, and were published in 131 journals. Figure 2 displayed the temporal distribution of the number of publications in the area of research on ginsenosides for treating breast cancer. Overall, research on ginsenosides and breast cancer was rare before 2013, with an average of about 5 publications per year. However, the average number of publications per year from 2014 to 2022 reached about 17, indicating a fast growth rate a fast growth rate, and so far, the field of therapeutic effects of ginsenosides on breast cancer is increasingly being studied.

### 2.3 Distribution of countries/territories and institutions

**FIGURE 3:**
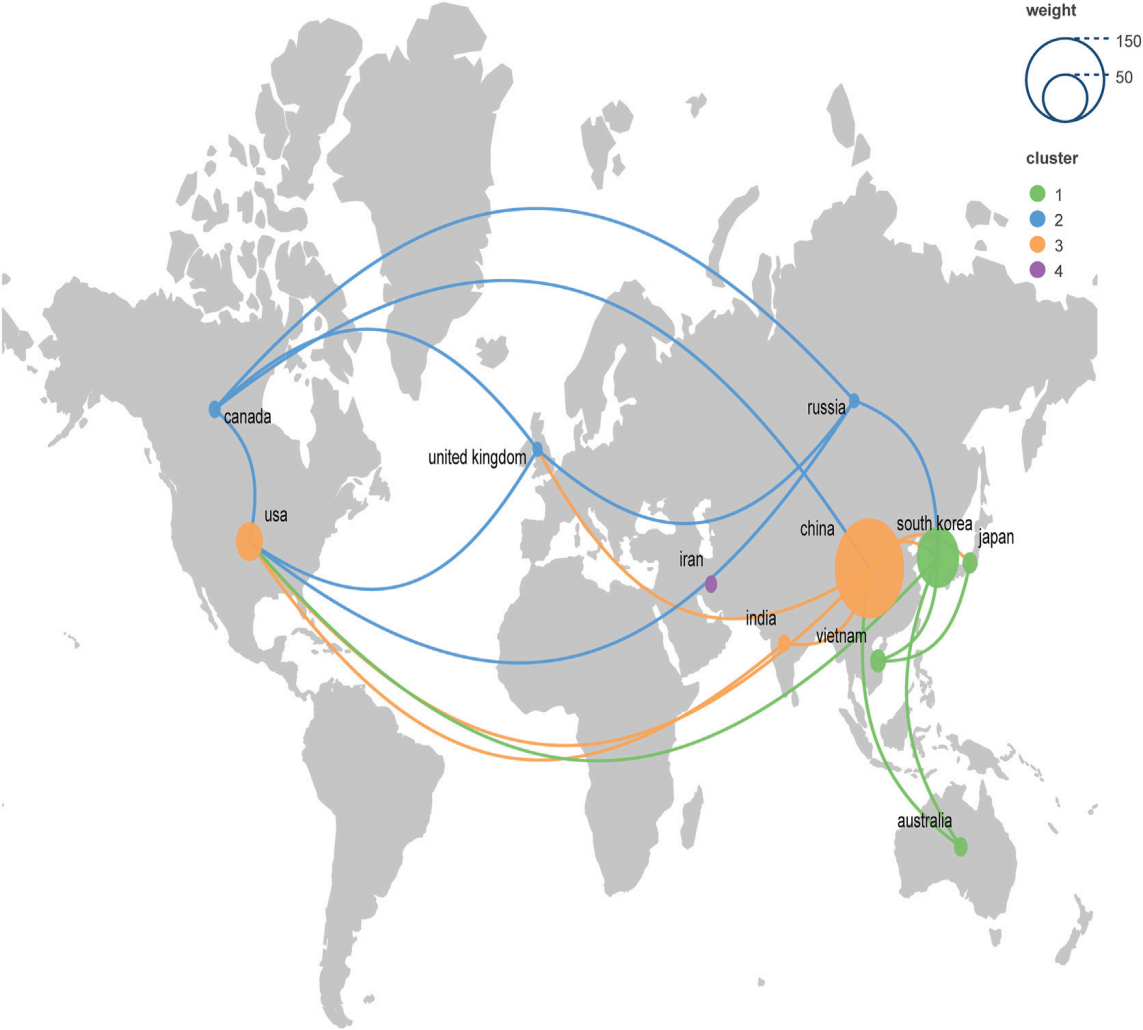
Geographic bibliometric map based on a network of co-authorship relationships in the top 10 countries in terms of number of articles published.

A total of 28 countries contributed to this study. Table 1 presented the top 14 countries in terms of the number of articles published. Among them, China had the highest number of publications, followed by South Korea, the United States, Vietnam, and Japan, respectively. In terms of article citations, China unsurprisingly had the highest number of citations due to having a large number of publications. But the average number of citations was not high. In contrast, the UK, with only 3 publications, had an average of 60 citations, which reflected the high quality of the articles. Figure 3 presented a geographic bibliometric map based on a network of co-authorship relationships in the top 10 countries in terms of the number of articles published. It is noteworthy that although China and South Korea contributed the most articles, other countries such as the United States, Vietnam, and Japan also provided a significant number of articles. Moreover, in terms of citations, all articles were of relatively high quality.

**TABLE 1:** The top 14 contries in terms of the number of publications.

Rank	Country	Publications	Total citations	Average citation
1	CHINA	132	3,800	29
2	SOUTH KOREA	49	1,662	34
3	USA	20	759	38
4	VIETNAM	7	222	32
5	JAPAN	6	197	33
6	AUSTRALIA	5	145	29
7	CANADA	4	151	38
8	INDIA	4	19	5
9	IRAN	4	65	16
10	ENGGLAND	3	179	60
11	RUSSIA	3	103	34
12	BRAZIL	2	91	46
13	EGYPT	2	23	12
14	PAKISTAN	2	41	21

**FIGURE 4:**
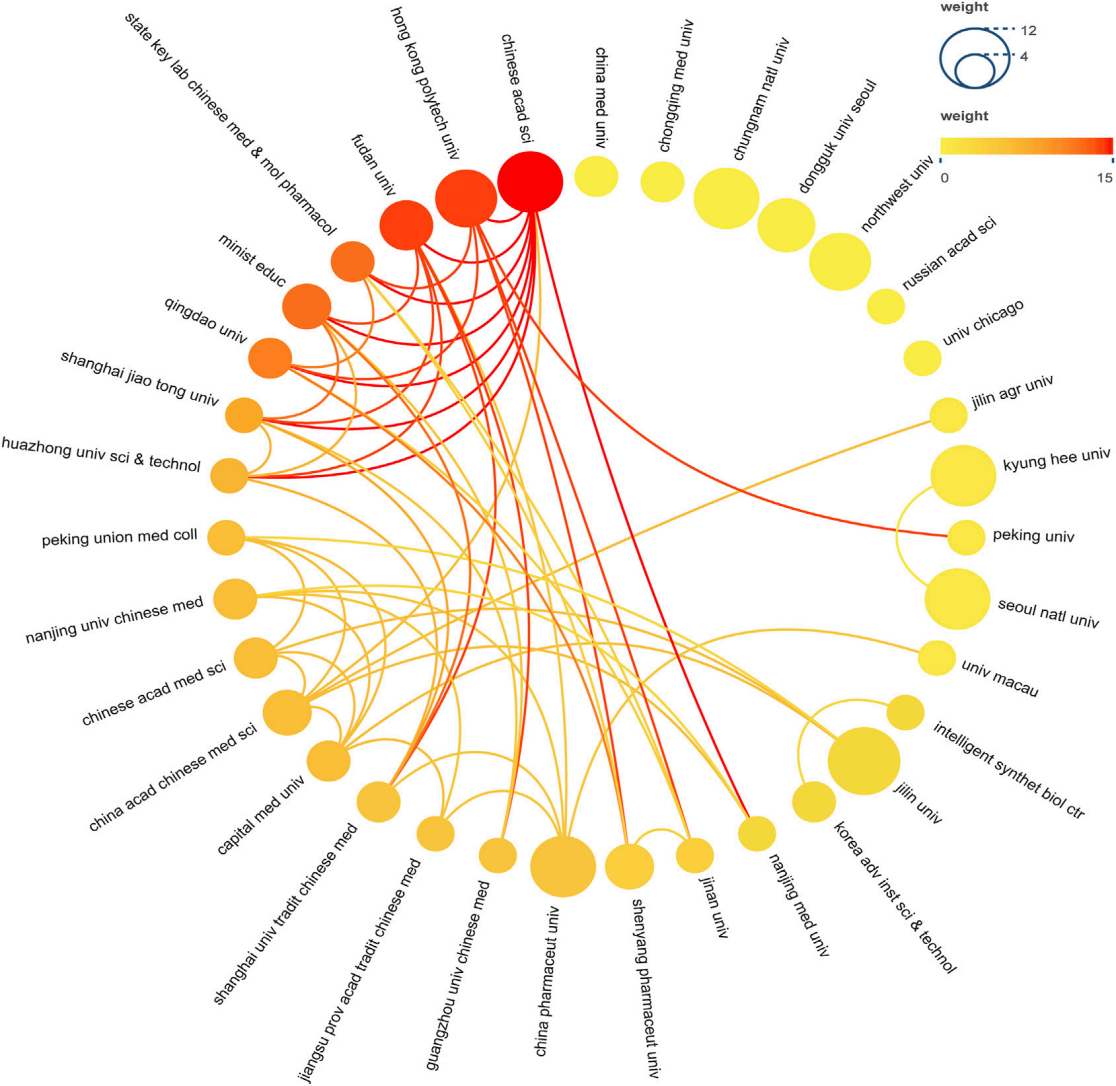
Institutional Cooperation Network Map. In the network, number of documents reflected by node size. The connection strength is reflected by the color.

A total of 302 institutions contributed to this research, Table 2 presented the top 10 institutions in terms of the number of publications. The Jilin University had the most publications but did not receive the highest number of citations. The highest number of citations and the average number of citations were obtained by Hong Kong Polytechnic University. Figure 4 demonstrated the collaborative relationships between multiple institutions. The Chinese Academy of Sciences collaborated the most with other institutions. However, in general, the institutions did not work very closely together.

**TABLE 2:** The top 10 institutes in terms of the number of publications.

Rank	Institute	Publications	Citations	Average citation
1	Jilin University	11	170	15
2	China Pharmaceutical University	9	292	32
3	Chinese Academy of Sciences	9	310	34
4	Chungnam National University	9	172	19
5	Kyung Hee University	9	311	35
6	SEOUL National University	9	510	57
7	Hong Kong Polytechnic University	8	512	64
8	northwest university	8	158	20
9	Dongguo University	7	128	18
10	Fudan University	6	119	20

### 2.4 Distribution of authors

Table 3 displayed the top 15 authors in terms of number of publications, with a minimum number of 4 publications. Among the highly productive authors, the one with the most publications was Fan Daidi, with a total of 23 publications from 2002 to 2022. Moreover, his articles received up to 158 citations, averaging 20 citations per article. Notably, an author named Wong Man-sau has received 325 citations despite having published only six relevant articles, indicating the high quality of her articles. Similar authors like Zhou Fang, Yang Deokchun, and Zhang Jingwei also gained high citations for their few articles.

**TABLE 3:** The top 15 authors in terms of number of publications.

Rank	Author	Publications	Citations	Average citation
1	fan, daidi	8	158	20
2	heo, kyung-sun	7	92	13
3	kim, sun jung	7	128	18
4	jin, yujin	6	73	12
5	wong, man-sau	6	325	54
6	kim, hyeon woo	5	58	12
7	wang, guangji	5	181	36
8	zhou, fang	5	181	27
9	jeong, dawoon	4	108	12
10	myung, chang-seon	4	46	9
11	wang, jianxin	4	37	9
12	xia, jiaxuan	4	37	9
13	yang, deok chun	4	90	23
14	zhang, jingwei	4	125	31
15	zhu, ying	4	37	9

**FIGURE 5:**
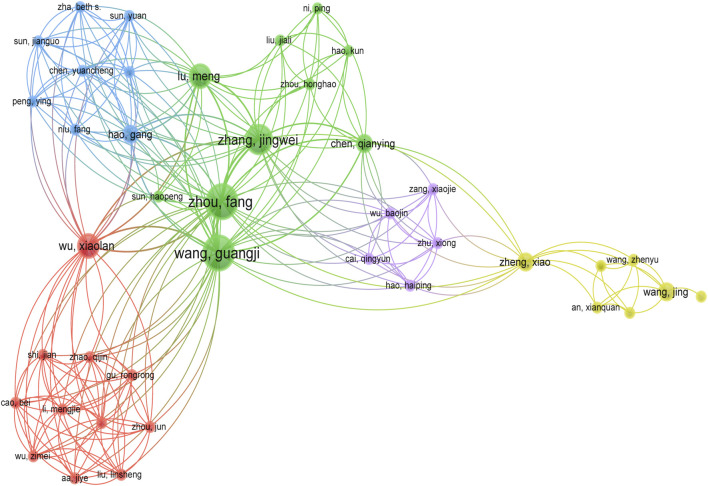
Co-authorship network map.

Figure 5 illustrated the collaborative relationships among the authors, and we found that six main teams contributed to the research on ginsenosides for breast cancer. However, there was no strong cooperative communication relationship between the different teams, and the strengths of resources were not well integrated and utilized.

### 2.5 Keyword analysis

**FIGURE 6:**
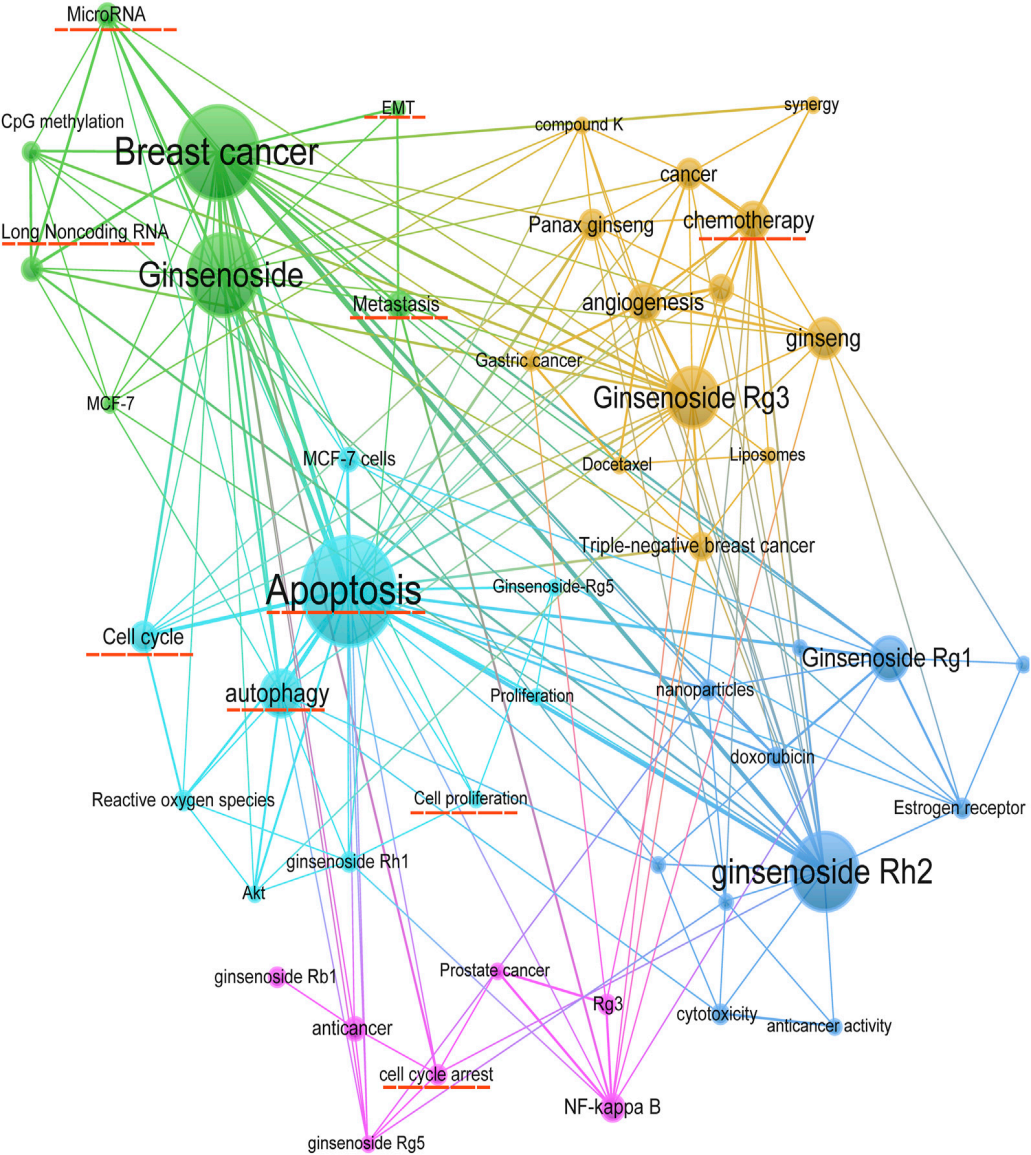
Keywords co-occurrence network.

**FIGURE 7:**
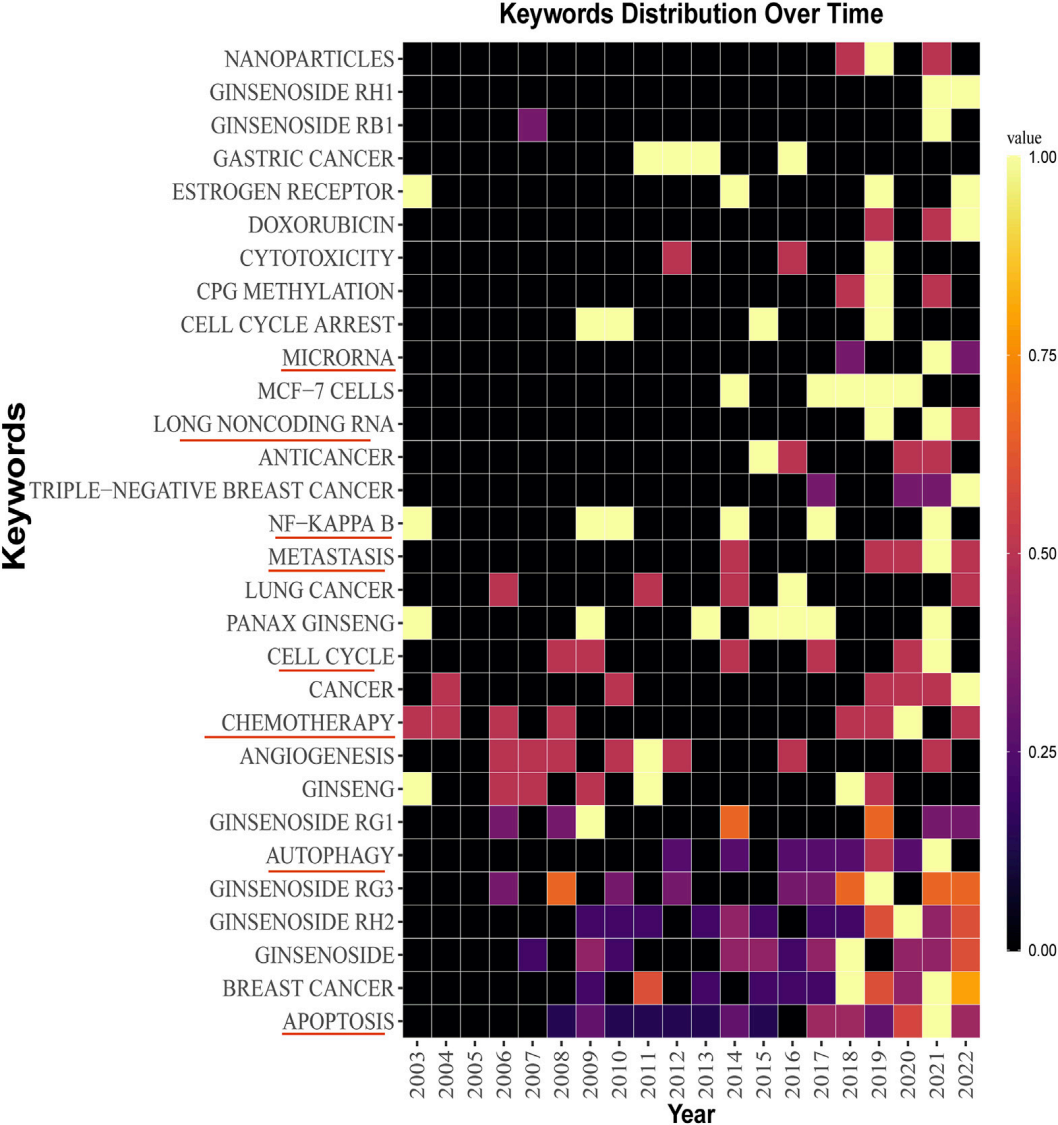
Keywords distribution over time.

The keywords co-occurrence analysis illustrates the evolution process and hot topics. The keyword visualization in this review was presented as a co-occurrence network (Figure 6) and a heat map (Figure 7). For the co-occurrence network, keywords were divided into various clusters represented by different colors based on the correlation. Notably, besides the keywords related to ginsenosides and breast cancer, “apoptosis” and “autophagy” were cross-cutting keywords in these clusters. It suggested that the way ginsenosides treat breast cancer was likely to be through the apoptotic, autophagic pathway. There were similar keywords like EMT, metastasis, cell cycle, etc. To understand the keywords evolution process, we used the keywords with high frequency to map the heat map of the distribution of the keywords over time. As shown in Figure 7, besides the keywords related to ginseng and breast cancer, the keywords like apoptosis, autophagy, metastasis, long non-coding RNA and microRNA appeared more frequently in recent years. These mechanisms may be a hot topic for future research. Preliminarily, these keywords may be the key pathway for ginsenosides in the treatment of breast cancer and these keywords appeared to be the hot topics in recent years. Therefore, we made a more in-depth study of ginsenosides for breast cancer based on these keywords.

## 3 The regulatory role of ginsenosides on breast cancer

### 3.1 Induction of apoptosis

Apoptosis, recognized as programmed cell death, represents an innate biological mechanism crucial for orchestrating equilibrium between cellular proliferation and demise within the organism. This process assumes the pivotal role of purging damaged or aberrant cells, preempting their potential deleterious impact. In breast cancer, the normal balance between cell growth and apoptosis is disrupted, leading to the uncontrolled growth and spread of cancer cells. Hence, instigating apoptosis within breast cancer cells emerges as a compelling therapeutic avenue, constituting a strategic maneuver for combating breast cancer and abrogating its propensity for metastatic expansion.

**FIGURE 8:**
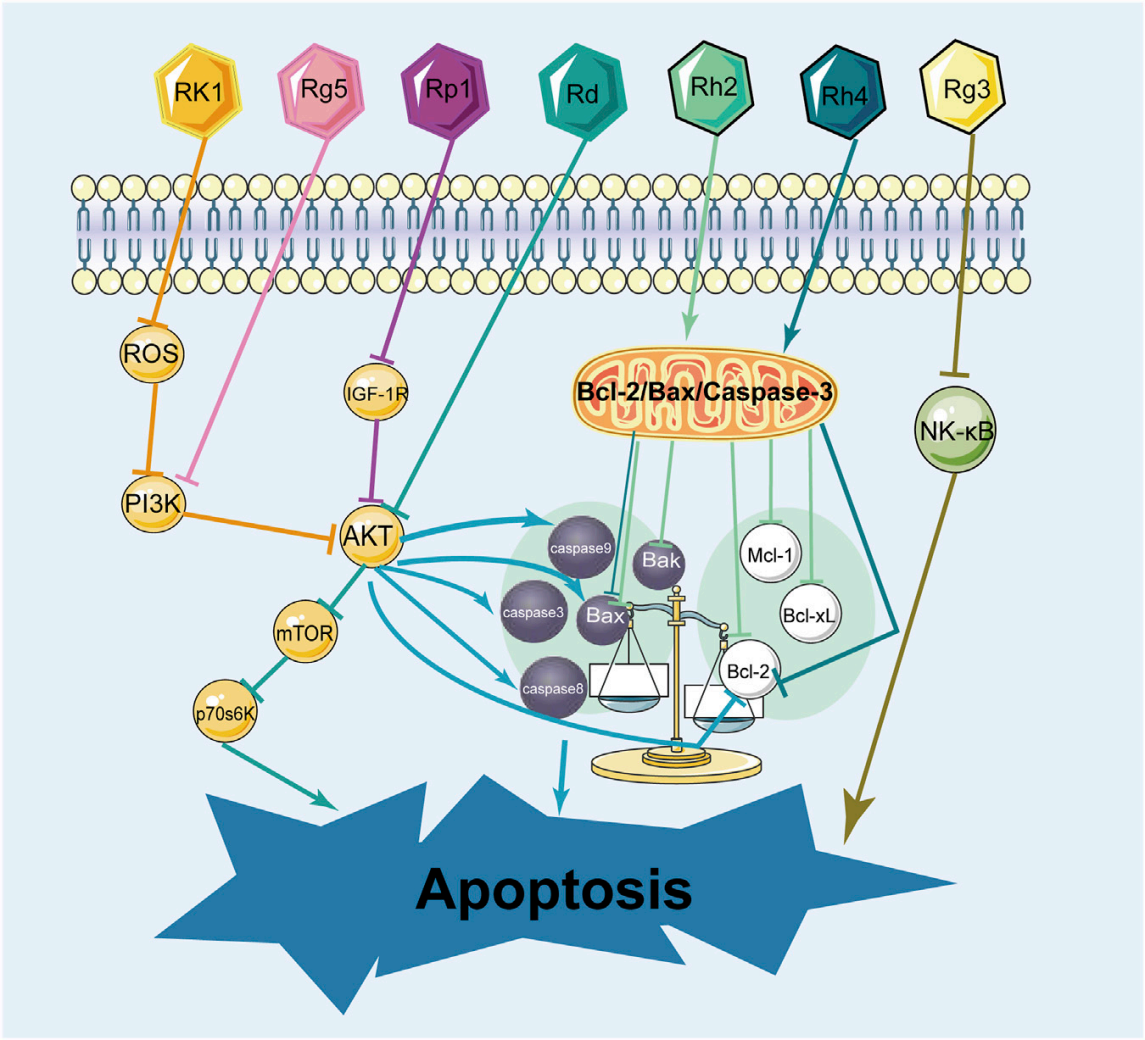
The mechanism of ginsenosides promoting apoptosis in breast cancer cells.

There are two main pathways for apoptosis: the intrinsic (mitochondrial) pathway and the extrinsic (death receptor) (Wilken et al., 2011). These pathways contribute to the activation of caspases and cause cancer cell death. The promotion of apoptosis is a common feature of anti-cancer medications and has been a theme of anticancer drug research for decades. Many experiments demonstrated that ginsenosides induced apoptosis in different types of breast cancer cells through various mechanisms. The effect of ginsenosides in promoting apoptosis on breast cancer cells was shown in Figure 8; Table 4.

**TABLE 4:** Intervention mechanism of different ginsenosides on apoptosis in breast cancer cells.

Subtype	Saponins	The mechanism of inducing apoptosis	References
C17 side-chain varied	Ginsenoside Rk1	Inhibition of the ROS/PI3K/Akt signaling pathway decreased Bcl-2 expression, upregulated Bax and cytochrome C, cleaving caspases 3, 8, and 9 expression	Hong and Fan (2019)
C17 side-chain varied	Ginsenoside Rg5	Dose-dependent inhibition of PI3K/Akt signaling pathway	Liu and Fan (2018)
C17 side-chain varied	Ginsenoside Rp1	Inhibition of the IGF-1R/Akt signaling pathway	Kang et al. (2011)
C17 side-chain varied	Ginsenoside Rh4	Reduced Bcl-2, increased Bax and caspase-8, 3 expression	Duan et al. (2018)
Protopanaxadiol	Ginsenoside Rd	Inhibition of the Akt/mTOR/p70S6K signaling pathway	Zhang et al. (2017)
Protopanaxadiol	Ginsenoside Rg3	Inhibition of NF-κb signaling pathway	Kim et al. (2014)
			Yuan et al. (2017)
Protopanaxadiol	Ginsenoside Rh2	Downregulation of Bcl-2, Bcl-xL, and Mcl-1 and increased expression of Bak, Bax, and Bim resulted in mitochondrial translocation of Bax and activation of caspases	Choi et al. (2011)

In terms of research on the induction of apoptosis in breast cancer cells, it was mainly the two types of ginsenosides, protopanaxadiol and C17 side-chain varied, that have been studied extensively. Signaling pathways play an influential role in apoptosis. As for the induction of apoptosis in breast cancer cells by ginsenosides, the PI3K/AKT signaling pathway was commonly involved. Ginsenoside Rk1 induced apoptosis in MDA-MB-231 cells by upregulating the expression of Bax and cytochrome C, cleaving caspases 3, 8, and 9, and decreasing the expression of Bcl-2 through the ROS/PI3K/Akt signaling pathway (Hong and Fan, 2019). Similarly, Ginsenoside Rg5 induced apoptosis in breast cancer cells by inhibiting the PI3K/Akt signaling pathway in a dose-dependent manner (Liu and Fan, 2018). Moreover, Ginsenoside Rp1 induced apoptosis in MCF-7, MDA-MB-231, and T-47D breast cancer cells through the insulin-like growth factor 1 receptor (IGF-1R)/Akt signaling pathway, thereby inhibiting breast the growth of breast cancer cells (Kang et al., 2011). Ginsenoside Rd inhibited the Akt/mTOR/p70S6K signaling pathway to promote apoptosis and suppressed angiogenesis in MDA-MB-231 cells (Zhang et al., 2017). In addition, the NF-κb signaling pathway also regulates apoptosis. Some experiments demonstrated that ginsenoside Rg3 promoted apoptosis in triple-negative breast cancer cells by inhibiting the NF-κb signaling pathway (Kim et al., 2014; Yuan et al., 2017).

Bcl-2/Bax/Caspase-3 is a signaling pathway closely related to apoptosis. Ginsenosides promote apoptosis in breast cancer cells by regulating the Bcl-2/Bax/Caspase-3 signaling pathway. Several experiments showed that ginsenoside Rh2 induced apoptosis in MCF-7 and MDA-MB-231 human breast cells (Choi et al., 2011), associated with mitochondria-mediated apoptosis. Besides, under the effect of ginsenoside Rh2, the expression of anti-apoptotic proteins Bcl-2, Bcl-xL, and Mcl-1 was downregulated, whereas the expression of pro-apoptotic factors Bak, Bax, and Bim was upregulated, causing mitochondrial translocation of Bax and caspases activation. It is reported that ginsenoside Rh4 induced apoptosis of breast cancer cells by reducing Bcl-2, increasing Bax, and activating caspase-8, 3 (Duan et al., 2018). These observations highlight that ginsenosides induce apoptosis in various breast cancer cells and interact with multiple pathways and targets.

The NF-κb signaling pathway and PI3K/AKT signaling pathway are signaling pathways closely related to apoptosis. Consequently, prevailing investigations into ginsenoside-induced apoptosis in breast cancer predominantly converge upon these two pathways, while other apoptosis-related pathways, such as the MAPK signaling pathway and Wnt signaling pathway, have not been studied yet. The pathway of inducing apoptosis in breast cancer cells by ginsenosides is supposed to be multi-pathway and multi-targeted, but the current study is more limited to the classical pathway, so it is necessary to explore the mechanism of inducing apoptosis by ginsenosides from various aspects. In addition, other types of ginsenosides have been less studied in this aspect of apoptosis, and more research on other types of ginsenosides should be conducted.

### 3.2 Regulation of breast cancer cell cycle and proliferation

The cell cycle is a tightly regulated process that controls the growth and division of cells in the body. It consists of several distinct phases, including G1, S, G2 and M. In breast cancer, cell cycle deregulation leads to malignant proliferation of cancer cells. Specifically, mutations or alterations in genes that regulate the cell cycle can disrupt the normal progression of cells at different stages of the cycle, leading to abnormal proliferation and tumor formation. Many current breast cancer therapies, such as chemotherapy and endocrine therapy, slow or stop the growth of cancer cells by blocking the cell cycle.

**FIGURE 9:**
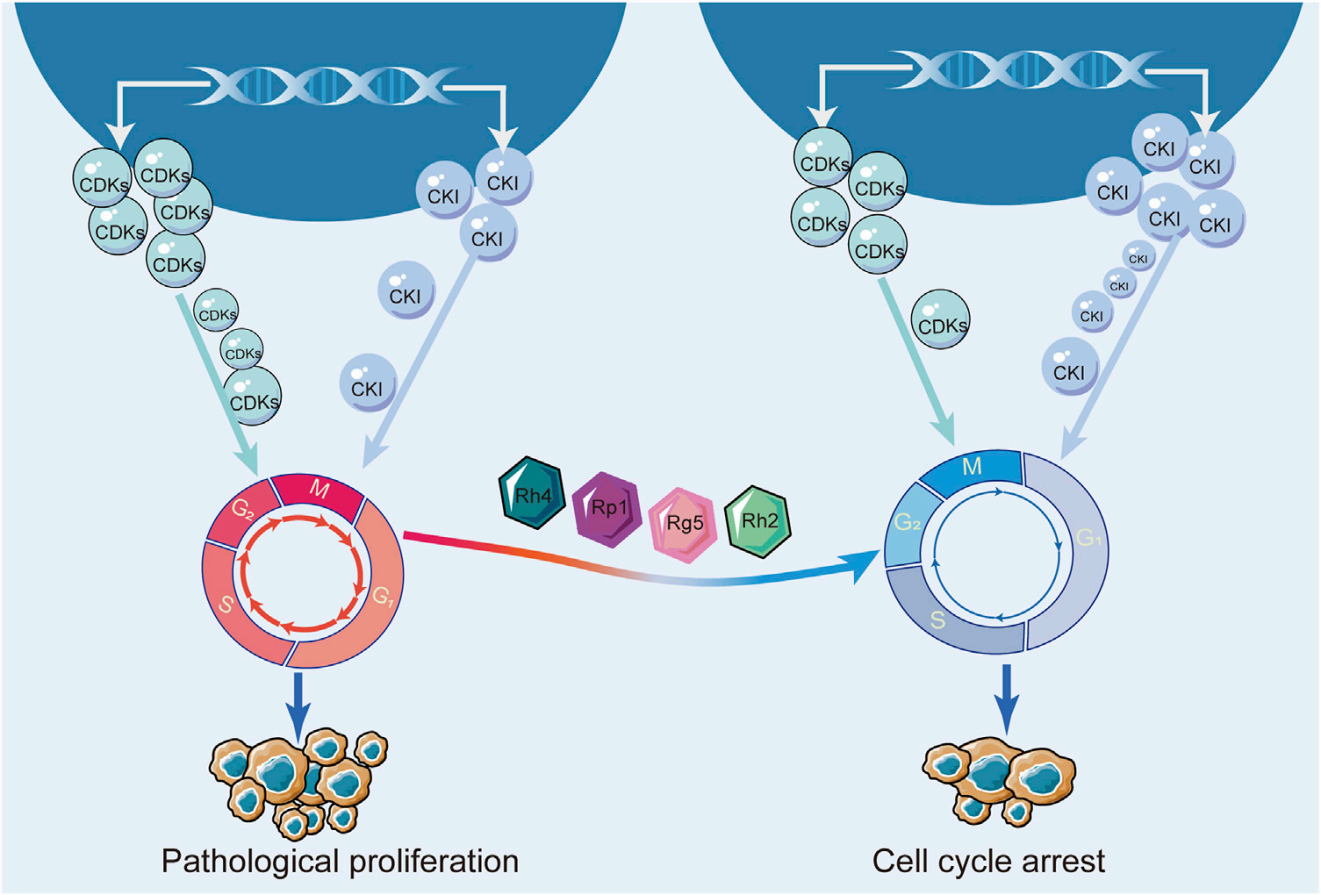
The mechanism of ginsenoside blocking breast cancer cell cycle.

Key regulators of this process are cell cycle-dependent kinases (CDKs) and cell cycle inhibitory proteins (CKIs). CDKs are proteins that promote cell cycle progression, while CKIs are proteins that inhibit cell cycle progression. When these regulatory proteins are not functioning properly, cells can begin to divide uncontrollably, leading to the growth of a tumor (Fry et al., 2004; Ding et al., 2020). Researchers envisioned blocking the cell cycle to stunt unlimited cell proliferation. Moreover, they have successfully developed CDK4/6 inhibitors to block the cell cycle and have shown promising results in the treatment of hormone receptor-positive breast cancer (Finn et al., 2015; Piezzo et al., 2020; Schettini et al., 2020). Numerous studies have demonstrated that ginsenosides inhibited various types of breast cancer cells. Ginsenosides Rk1 and Rp1 contributed to cell cycle arrest in triple-negative breast cancer cells (Kang et al., 2011; Hong and Fan, 2019). Ginsenosides Rh4, Rp1, Rg5, Rh2, 20(S)-Protopanaxadiol all induced cell cycle arrest in hormone receptor-positive breast cancer cells (Kang et al., 2011; Kim and Kim, 2015, 5; Duan et al., 2018; Zhang et al., 2018; Peng et al., 2022). More specifically, the mechanism of ginsenoside blocking the cell cycle in breast cancer affected the expression of CDKs and CKI. Ginsenoside Rg5 upregulated the expression of CKI, such as p53, p21, and p15, and downregulated the expression of cycle-dependent kinases, such as Cyclin D1, Cyclin E2, and CDK4 (Kim and Kim, 2015, 5). Therefore, these current studies revealed that ginsenosides induced cell cycle arrest by regulating cell cycle-related proteins. The effects of ginsenosides on the cell cycle of breast cancer were shown in Figure 9; Table 5.

**TABLE 5:** Various ginsenosides block the cell cycle in different types of breast cancer.

Subtype	Saponins	Types of breast cancer	References
C17 side-chain varied	Ginsenoside Rk1	Triple-negative breast cancer	Hong and Fan (2019)
C17 side-chain varied	Ginsenoside Rg5	Hormone receptor–positive breast cancer	Kim and Kim (2015)
C17 side-chain varied	Ginsenoside Rp1	Triple-negative breast cancer, Hormone receptor–positive breast cancer	Kang et al. (2011)
C17 side-chain varied	Ginsenoside Rh4	Hormone receptor–positive breast cancer	Duan et al. (2018)
Protopanaxadiol	Ginsenoside Rh2	Hormone receptor–positive breast cancer	Peng et al. (2022)
Protopanaxadiol	20(S)-Protopanaxadiol	Hormone receptor–positive breast cancer	Zhang et al. (2018)

Regulation of cell cycle to inhibit tumor growth is a fundamental property of antitumor drugs, but for the wide variety of ginsenosides, only a small number of ginsenosides with cell cycle regulation have been found in the current study. Moreover, some studies involved only one type of breast cancer. Therefore, further studies are needed to investigate the cell cycle regulatory effects of other types of ginsenosides on different types of breast cancer. In addition, the current study on the mechanism of ginsenosides regulating the cell cycle is not deep enough, and it is necessary to further elucidate the deep mechanism of ginsenosides regulating the cell cycle.

### 3.3 Induction of autophagy

Autophagy is the process of self-degradation of some intracellular components through the autophagic lysosomal pathway. On the one hand, autophagy induces apoptosis. When autophagy is overactivated, lysosomes degrade cellular components and induce cell death (Linder and Kögel, 2019). On the other hand, autophagy inhibits apoptosis. Autophagy provides nutrients to tumor cells in nutrient-deficient and low-oxygen environments and protects them from apoptosis (White, 2015; Onorati et al., 2018). The bi-nature of autophagy has attracted the attention of many scholars. Accumulating evidence showed that ginsenosides induced autophagy of breast cancer cells.

Ginsenoside Rg5 exerted a profound impact on breast cancer cell proliferation by invoking mitochondria-mediated autophagic cell death, as evidenced by a notable attenuation of cellular proliferation (Liu and Fan, 2018). Similarly, ginsenoside Rh1 and ginsenoside (20S)-protopanaxatriol induced non-protective autophagy and inhibited breast cancer cell proliferation through the PI3K/AKT pathway (Huynh et al., 2021; Li et al., 2021). Its mechanism was associated with the formation of autophagosomes and elevated expression levels of LC3BII, P62, and Atg proteins. In addition, these observations highlight that ginsenosides not only promote apoptosis in breast cancer cells through the PI3K/AKT signaling pathway, but also induce cellular autophagy through this signaling pathway. Therefore, the PI3K/AKT signaling pathway is an important pathway for ginsenosides to exert autophagic effects.

**FIGURE 10:**
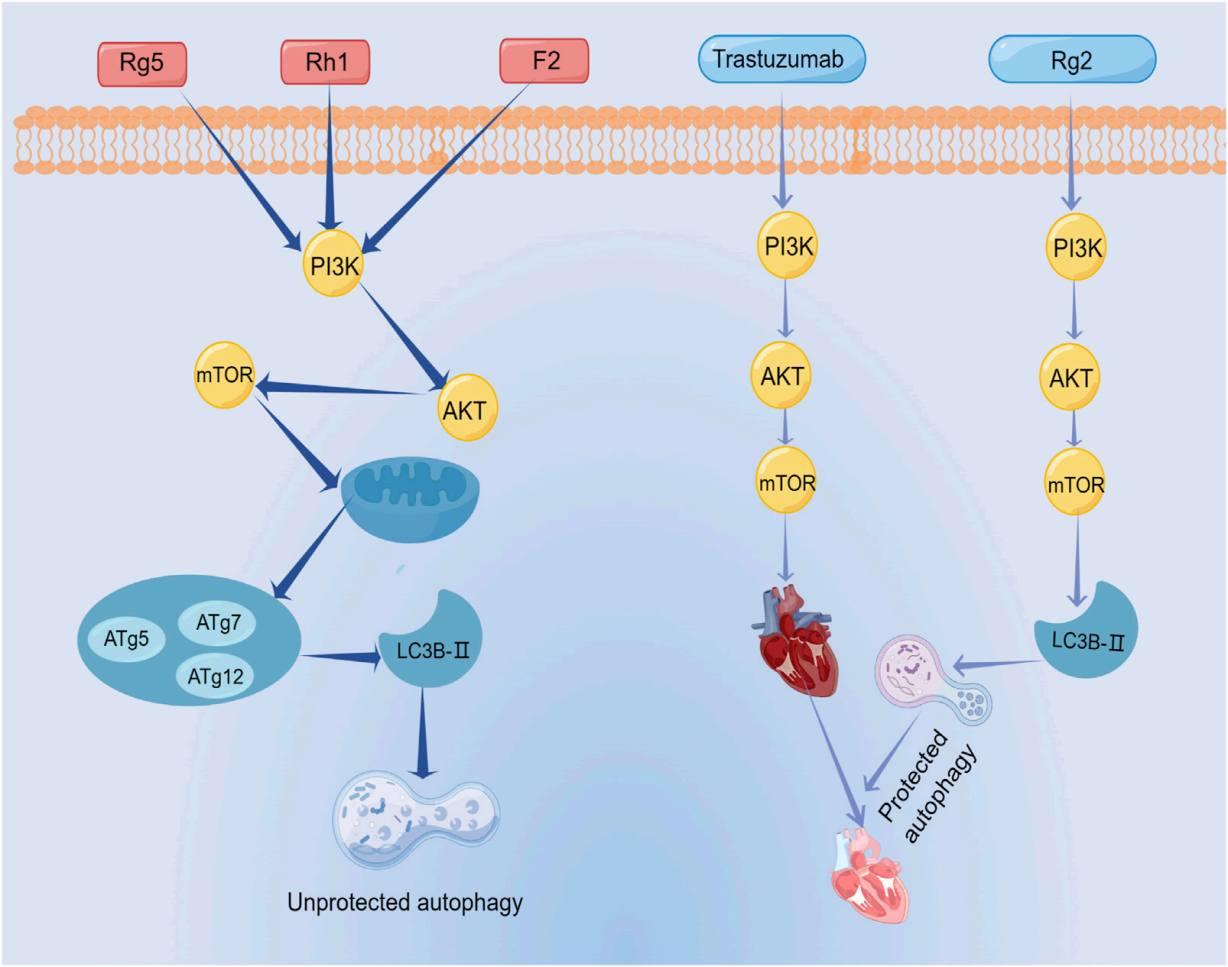
The mechanism of autophagy induced by ginsenosides.

As mentioned above, the relationship between autophagy and apoptosis is two-fold. In some cases, ginsenosides elicit autophagy that proves protective for cancer cells. An example is the case of ginsenoside F2, which both promotes apoptosis in breast cancer stem cells and induces autophagy within the same context. As mentioned above, the relationship between autophagy and apoptosis is two-fold. In some cases, ginsenosides elicit autophagy that proves protective for cancer cells. An example is the case of ginsenoside F2, which both promotes apoptosis in breast cancer stem cells and induces autophagy within the same context. (Mai et al., 2012). The evidence suggested that autophagy of breast cancer stem cells limits the effect of ginsenoside F2, which is a common problem of numerous anti-cancer drugs today. In other words, autophagy is closely related to drug resistance of tumors (Hu et al., 2022). However, some ginsenoside-induced protective autophagy is essential. The therapeutic prominence of trastuzumab in breast cancer treatment is undeniable. However, its clinical utility is constrained by its cardiotoxicity. A recent investigation unveiled the shielding potential of ginsenoside Rg2 against trastuzumab-triggered cardiotoxicity through the induction of autophagy in cardiomyocytes (Liu et al., 2021). This result illustrated ginsenoside Rg2 as a potential therapeutic agent to prevent cardiotoxicity associated with medications for treating breast cancer. The mechanism of autophagy induced by ginsenosides was shown in Figure 10; Table 6.

**TABLE 6:** Induction of autophagy in breast cancer cells by ginsenosides.

Subtype	Saponins	Effects of autophagy	References
C17 side-chain varied	Ginsenoside Rg5	Inhibited the proliferation of breast cancer cells	Liu and Fan (2018)
C17 side-chain varied	Ginsenoside F2	Induced autophagy was protective of breast cancer stem cells	Mai et al. (2012)
Protopanaxatriol	Ginsenoside Rh1	Inhibited the proliferation of breast cancer cells	Huynh et al. (2021)
Protopanaxatriol	(20S)-Protopanaxatriol	Inhibited the proliferation of breast cancer cells	Li et al. (2021)
Protopanaxatriol	Ginsenoside Rg2	Induced autophagy prevented trastuzumab-induced cardiomyocytotoxicity	Liu et al. (2021)

From the above studies, ginsenoside-induced autophagy exerted mostly anti-tumor effects on breast cancer. In addition, the protective autophagy induced by ginsenosides can also be used synergistically with anti-breast cancer drugs to reduce drug toxicity. This strategic integration holds the promise of expanding the therapeutic purview of ginsenosides within the realm of breast cancer treatment.

### 3.4 Inhibition of metastasis

Metastasis is the process by which cancer cells spread from the primary tumor to other parts of the body and is a major cause of mortality in breast cancer patients. Despite the wide range of treatments and medications available for breast cancer, it is reported that 20%–30% of breast cancer patients develop metastases after diagnosis or treatment of the primary tumor. This is a major concern, as metastases are often the cause of death in up to 90% of breast cancer cases (Yoshimaru et al., 2013). As a result, it is critical to focus on treatments that target metastases in order to improve outcomes for breast cancer patients.

There were only a few studies on ginsenosides against breast cancer metastasis and they were all focused on the category of protopanaxadiol. Circulating tumor cells, a type of tumor cell in the bloodstream, play a seed-like role in breast cancer metastasis. Metastatic niches are like soil. Multifunctional Rg3 liposomes loaded with doxorubicin was specifically targeted on circulating tumor cells and prevented the formation of metastatic niches by altering the immunosuppressive microenvironment (Xia et al., 2022). In another breast cancer study, ginsenoside Rd treatment resulted in a reduction in lung tumor lesions in a spontaneous and experimental model of metastasis. This mechanism was related to the fact that ginsenoside 2 decreased miR-18a expression and elevated Smad2 expression (Wang et al., 2016). In addition, a previous *in vivo* and *in vitro* study demonstrated that ginsenoside 20(S)-protopanaxadiol significantly inhibited the growth and lung metastasis of triple-negative breast cancer. The mechanism involved the inhibition of the EGFR-mediated MAPK signaling pathway and the reversal of EMT by ginsenoside 20(S)-protopanaxadiol.

The exploration of ginsenosides as potential suppressors of breast cancer metastasis appears to have been a less traversed avenue within the realm of scholarly inquiry. But breast cancer metastasis is very relevant to the prognosis of breast cancer patients. Therefore, we hope that more researchers would explore the inhibition of breast cancer metastasis by ginsenosides in the future.

### 3.5 Regulation of EMT

EMT is a process by which epithelial cells, which are normally tightly connected and organized in a specific tissue structure, transform into mesenchymal cells, which are more motile and invasive. EMT plays an important role in cancer progression by promoting invasion and metastasis. In breast cancer, EMT has been shown to play a critical role in the development of invasive and metastatic disease. During EMT, breast cancer cells lose their epithelial characteristics, such as cell-cell adhesion and polarity, and acquire mesenchymal characteristics, such as increased motility and invasiveness. This allows the cancer cells to break away from the primary tumor, invade surrounding tissues, and establish new tumors in distant organs (Mitra et al., 2015; Felipe Lima et al., 2016). Transcription factors such as Snail, Twist1, Slug, and ZEB1 can promote EMT by reducing or inhibiting the protein E-calmodulin. This leads to the conversion of epithelial cells into mesenchymal cells, allowing the cancer cells to spread more easily to other parts of the body (Uchikado et al., 2011; Wang et al., 2011; Deep et al., 2014). The strategic interception of EMT assumes a position of growing significance, materializing as a fulcrum for the prospect of forestalling and treating breast cancer metastasis.

**FIGURE 11:**
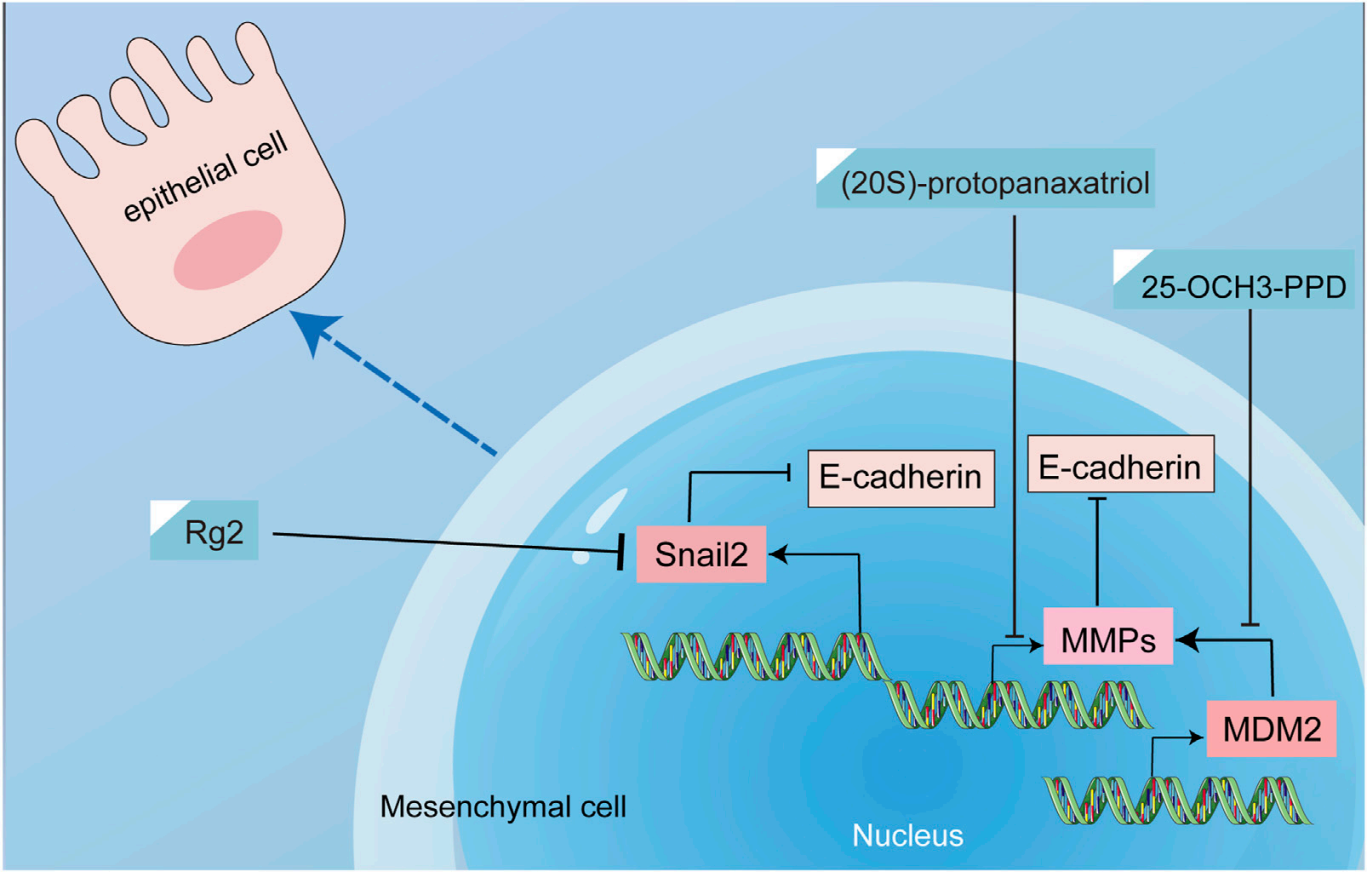
The mechanism of inhibiting EMT by ginsenosides.

Treatment with ginsenoside Rg1 has shown a notable impact on breast cancer tissues by downregulating SNAIL2 expression and significantly attenuating the expression of pivotal regulators of cell growth, including MAPK, EGFR, and TGF-β(Chu et al., 2020). This modulation is of particular importance since previous research has underscored the role of MAPK, EGFR, and TGF-β in fueling the progression of EMT, a pivotal driver of metastasis (Heldin et al., 2012; Serrano et al., 2014; Zhang et al., 2022b). A crucial driver of tumor invasion and metastasis is matrix metalloproteinases (MMPs), which facilitate the detachment of epithelial cells from their confines. Moreover, heightened MMP levels have been implicated in the degradation of E-calmodulin, a key mediator of EMT in cancer cells (Radisky and Radisky, 2010). Ginsenoside 20(S)-protopanaxadiol was reported to reduce the expression and activity of MMPs, while increasing the expression of tissue inhibitors of metalloproteinases (TIMPs), thus reinforcing its capacity to deter EMT progression (Peng et al., 2019). In addition, ginsenoside 25-OCH3-PPD inhibited EMT and prevented breast cancer metastasis by downregulating MDM2 (Wang et al., 2012, 2). MDM2 has been demonstrated to be closely associated with breast cancer invasion and metastasis. Moreover, MDM2 enhanced the invasion and migration of breast cancer cells by upregulating the expression of MMP-9. Therefore, MDM2 played a very important role in EMT (Rayburn et al., 2005; Chen et al., 2013, 9). Recognized as instrumental in fostering immune dysfunction and amplifying metastatic propensities, myeloid-derived suppressor cells (MDSCs) have emerged as a focal point of exploration in the context of tumor metastasis (Safarzadeh et al., 2018; Cole et al., 2021). Ginsenoside Rg3 has shown promise in attenuating cancer stemness and countering mesenchymal transformation by influencing MDSC modulation (Song et al., 2020). Many studies have demonstrated that ginsenosides can inhibit breast cancer EMT, but few articles have explored the deeper mechanisms of EMT inhibition. The mechanism of inhibiting EMT by ginsenosides was shown in Figure 11; Table 7.

**TABLE 7:** Interventional effects of ginsenosides on EMT in breast cancer.

Subtype	Saponins	The mechanism of inducing apoptosis	References
Protopanaxatriol	Ginsenoside Rg1	Reduced expression of SNAIL2, MAPK, EGFR and TGF-β in breast cancer tissues	Chu et al. (2020)
Protopanaxadiol	Ginsenoside 20(S)-protopanaxadiol	Decreased expression and activity of MMPs and increased expression of TIMPs	Peng et al. (2019)
Protopanaxadiol	Ginsenoside 25-OCH3-PPD	Reduced MDM2 expression and expression of EMT markers β-catenin, Twist, Vimentin, and Snail1	Wang et al. (2012)
Protopanaxadiol	Ginsenoside Rg3	Reduced MDSC expression and expression of EMT markers β-catenin and Vimentin	Song et al. (2020)

### 3.6 Regulation of miRNAs

MicroRNAs (miRNAs) are small, non-coding RNAs that play a vital role in the regulation of gene expression. They can act as either tumor suppressors or oncogenes, depending on the specific miRNA and the context in which it is expressed. In breast cancer, dysregulation of miRNAs has been implicated in the development and progression of the disease. Specifically, some miRNAs have been shown to be overexpressed in breast cancer to promote tumor growth and metastasis, while others are downregulated and may act as tumor suppressors. MiRNAs can affect various cellular processes involved in breast cancer development, such as cell proliferation, apoptosis, angiogenesis, invasion, and metastasis (Zhang et al., 2022a; Ismail et al., 2022). Overall, Targeting regulatory miRNAs has emerged as a promising strategy for the prevention and treatment of breast cancer.

Current studies only found that protopanaxadiol can interfere with miRNAs, thereby affecting apoptosis, proliferation, metastasis, and drug resistance in breast cancer cells. Ginsenoside Rh2 exhibited a role in upregulating miR-3614-3p expression, resulting in the inhibition of MCF-7 proliferation and the induction of apoptosis (Park et al., 2022). In addition, ginsenoside Rh2 downregulated miRNA-4425, inducing growth inhibition and apoptosis in various breast cancer cells (Park et al., 2021). Besides, ginsenoside Rh2 regulated miR-222, miR-34a, and miR-29a to attenuate chemoresistance in breast cancer (Wen et al., 2015). These observations highlighted the ability of ginsenoside Rh2 to target multiple miRNAs and thus contributed to therapeutic efficacy and reduced drug resistance as a potential enhancer of chemotherapy sensitivity. Several studies indicated that some oncogenes and tumor suppressor genes were under the control of miRNAs(Tong et al., 2016). Consequently, it is plausible that ginsenosides may mediate the upregulation of tumor suppressor genes and downregulation of oncogenes via miRNA modulation. EYA1, DACH1, and CHRM3 are considered as oncogenes. Ginsenoside Rg3 and Rd also showed an effect on the regulation of miRNAs. Ginsenoside Rg3 suppressed the expression of the three genes by regulating miRNA-424-5p (Kim et al., 2021). In another study, under the intervention of ginsenoside Rd, the expression of microRNA-18a decreased, whereas the expression of Smad2, an anti-oncogene, elevated significantly (Wang et al., 2016).

As seen with ginsenoside Rh2, one ginsenoside tends to modulate multiple miRNAs and thus exert multiple effects. However, there were few studies on ginsenosides regulating miRNAs, and ginsenosides regulating multiple miRNAs except Rh2 have not been found yet, but they certainly exist, and thus need to be further explored.

### 3.7 Regulation of lncRNA

Long non-coding RNAs (lncRNAs) are endogenously derived non-coding RNA molecules that exceed a length of 200 nucleotides. Operating through transcriptional and post-transcriptional regulatory mechanisms, lncRNAs actively participate in various cellular physiological processes. These mechanisms encompass interactions with microRNAs (miRNAs), leading to the modulation of target gene regulation by miRNAs, as well as engagement in the competing endogenous RNA (ceRNA) network (Yang et al., 2016). Furthermore, it has been demonstrated that a variety of lncRNAs were aberrantly expressed in breast cancer tissues and cell lines, which were involved in regulating breast cancer cell proliferation, invasion, migration, apoptosis, and drug resistance (Liu et al., 2015). Taken together, in breast cancer, dysregulation of lncRNAs is associated with the development and progression of the disease. It is interesting to note that ginsenosides regulated lncRNAs to exert therapeutic effects on breast cancer.

The landscape of studies exploring the modulation of long non-coding RNAs (lncRNAs) by ginsenosides for breast cancer treatment is relatively sparse. Specifically, among the diverse array of ginsenosides, only protopanaxadiol compounds (Rg3 and Rh2) have been identified thus far for their capacity to regulate lncRNAs in breast cancer. Notably, a series of experiments have demonstrated the attenuation of tumor tissue and cell growth through the suppression of miRNA-4425 expression (Zhang et al., 2019; Lu et al., 2020). Moreover, Ginsenoside Rh2 has been observed to elevate the expression of the lncRNA STXBP5-AS1, which targets miRNA-4425, resulting in the induction of apoptosis and the inhibition of cell proliferation in breast cancer cells (Park et al., 2021). MiRNA-424-5p was reported to enhance chemotherapy sensitivity and regulate the cell cycle, apoptosis, and proliferation, playing a role as an important therapeutic player in breast cancer (Dastmalchi et al., 2021). A previous experiment reported that lncRNA ATXN8OS suppressed miRNA-424-5p expression. However, ginsenoside Rg3 prevented the oncogenic lncRNA ATXN8OS from repressing microRNA-424-5p (Kim et al., 2021). Additionally, a recent investigation has reported that ginsenosides Rh2 downregulate the activity of lncRNA CFAP20DC-AS1 while concurrently elevating the expression of miRNA-3614-3p, consequently prompting apoptosis and inhibiting cell proliferation in breast cancer cells (Park et al., 2022). Furthermore, ginsenoside Rh2 has been demonstrated to inhibit breast cancer cell proliferation through the methylation modification of the promoter region of lncRNA C3orf67-AS1. And ginsenoside Rg3 regulated the methylation of lncRNA RFX3-AS1 and STXBP5-AS1, which affected the expression of their RFX3 and GRM1 target genes, thereby inhibiting the proliferation of breast cancer cells (Ham et al., 2019, 1).

Long non-coding RNAs (lncRNAs) represent a pivotal class of molecules governing diverse facets of breast cancer biology. The exploration of lncRNAs has emerged as a burgeoning field of research, signifying their growing significance. Presently, investigations into the interplay between ginsenosides and lncRNAs in the context of breast cancer therapeutics remain relatively limited. However, we anticipate a growing surge in future studies that delve into the interactions between ginsenosides and lncRNAs.

### 3.8 Regulation of epigenetic modifications

Epigenetic modifications refer to changes in gene expression that are not caused by changes in the underlying DNA sequence. These modifications include DNA methylation, histone modifications, ubiquitination, and so on. These mechanisms play a key role in regulating gene expression and cause genes to be either activated or silenced under different conditions (Kelly et al., 2010; Zhao and Shilatifard, 2019). Moreover, epigenetic modifications have great significance in the treatment of breast cancer (Abdel-Hafiz and Horwitz, 2015; Karami Fath et al., 2022). By regulating epigenetic modifications, it promotes the expression of oncogenes and silences pro-oncogenes, thereby effectively promoting apoptosis and inhibiting the further development of breast cancer. In conclusion, intervening in epigenetic modifications during breast cancer development can inhibit tumor growth and prevent metastasis.

Currently, studies found only one type of ginsenoside, the protopanaxadiol, to treat breast cancer through epigenetic modification. Notably, methylation stands as a pivotal factor in tumorigenesis and progression (Pouliot et al., 2015). Ginsenoside Rh2 was reported to reduce m6A RNA methylation by downregulating KIF26B expression in breast cancer cells (Hu et al., 2021). In another study, ginsenoside Rh2 induced hypomethylation of an entire chromosomal element LINE1. Besides, Rh2 downregulated highly methylated oncogenes (Lee et al., 2018). By genome-wide methylation analysis, ginsenoside Rg3 downregulated oncogenes by altering methylation levels (Ham et al., 2018, 3). These findings revealed that ginsenosides regulated the methylation levels of breast cancer-related genes, thereby inhibiting the growth of breast cancer cells. Ubiquitination degrades certain key oncoproteins, pro-metastatic proteins, and proteins associated with tumor drug resistance so that cancer patients benefit from them (Xiao et al., 2016; Tecalco-Cruz and Ramírez-Jarquín, 2018). Ginsenoside Rd was reported to increase the ubiquitination of MDR1 in MCF-7 cells, leading to a decrease in the expression of this MDR1 gene associated with multidrug resistance, which reduced resistance to chemotherapy drugs (Pokharel∗ et al., 2010).

The current findings only reported that ginsenosides can regulate breast cancer development and drug resistance through ubiquitination and methylation, two epigenetic modifications. However, other epigenetic modifications are equally important, such as acetylation, glycosylation, and phosphorylation. These modifications have not been reported, which is a blank that needs to be filled urgently in the current study of ginsenosides against breast cancer.

### 3.9 Combination with chemotherapy drugs

Chemotherapy, while a cornerstone in breast cancer treatment, is often limited by drug resistance and adverse effects that some patients encounter. Ginsenosides not only alleviated the adverse effects of chemotherapy drugs but also acted on breast cancer through multi-targeting, thus reducing the toxicity and increasing the effectiveness. Therefore, the combination of ginsenosides with chemotherapeutic agents has garnered attention as a judicious therapeutic strategy.

The only reported ginsenoside used in combination with chemotherapeutic agents is protopanaxadio. 20(S)-ginsenoside Rh2 curbed adriamycin-induced oncogene ABCB1 expression within MCF-7/Adr cells. Further effect lay in its suppression of adriamycin-amplified NF-κB’s affinity for the human multidrug resistance (MDR1) promoter, effectively abating adriamycin resistance (Zhang et al., 2012, 2). Another study unearthed the capabilities of ginsenosides Rd to quell MDR1 expression in MCF-7/ADR cells. Besides, ginsenoside Rd reversed doxorubicin resistance in MCF-7/ADR cells (Pokharel∗ et al., 2010). These results indicated that ginsenosides reversed breast cancer drug resistance, which helped chemotherapy drugs work better. In addition, the combination of ginsenosides and chemotherapeutic agents enhanced the anti-tumor activity. It has been reported that ginsenoside Rg3 activated the cytotoxicity of paclitaxel by inhibiting the NF-κB signaling pathway. Moreover, the union of ginsenoside Rg3 and paclitaxel elicited a more pronounced reduction in Bcl-2 protein expression, accompanied by an augmentation in Bax and Caspase-3 protein expression within breast cancer cells, thus fortifying the tumor-suppressive potential beyond that of paclitaxel monotherapy (Yuan et al., 2017). Similarly, ginsenoside compound K combined with cisplatin-induced apoptosis and inhibited the proliferation and EMT of breast cancer cells better than these two drugs alone (Zhang and Li, 2016). The effects of ginsenosides in combination with chemotherapeutic drugs were shown in Table 8.

**TABLE 8:** Effects of ginsenosides in combination with chemotherapeutic agents.

Subtype	Saponins	Drugs combined with Ginsenoside	Effects	References
Protopanaxadiol	ginsenoside Rh2	Adriamycin	Inhibited adriamycin-induced expression of the oncogene ABCB1 in MCF-7/Adr cells; inhibited adriamycin-enhanced NF-κB binding to the human multidrug resistance (MDR1) promoter and reduced adriamycin resistance	Zhang et al. (2012)
Protopanaxadiol	Ginsenoside Rd	Doxorubicin	Reduced MDR1 expression in MCF-7/ADR cells and reversed doxorubicin resistance in MCF-7/ADR cells	Pokharel∗ et al. (2010)
Protopanaxadiol	Ginsenoside compound K	Cisplatin	Enhanced cisplatin-induced apoptosis and inhibition of proliferation and EMT in breast cancer cells	Zhang and Li (2016)
Protopanaxadiol	Ginsenoside Rg3	Paclitaxel	Inhibited the NF-κB signaling pathway, activated the cytotoxicity of paclitaxel, and enhanced the pro-apoptotic ability of paclitaxel	Yuan et al. (2017)

These observations highlight that ginsenosides combined with chemotherapeutic agents achieved better efficacy than those used alone. These studies involved both *in vivo* and *in vitro* experiments, but the clinical application of ginsenosides must be supported by data from clinical research. Therefore, large-scale clinical trials should be conducted to provide data support for the clinical application of ginsenosides in combination with other chemotherapeutic agents for the treatment of breast cancer.

## 4 Conclusion

Breast cancer is the most prevalent cancer in women worldwide with a high rate of metastasis. With medical advances, the OS and DFS of patients with early-stage breast cancer have improved significantly. But not all breast cancer patients benefit from treatment, especially advanced breast cancer is still a very difficult problem. Ginseng is full of possibilities and interest, as it is considered to be a life-prolonging and health-enhancing medication and has received a lot of attention. Ginsenosides, as the main active ingredient of ginseng, have been widely explored for their therapeutic potential in breast cancer.

Through a combination of bibliometric analysis, this review delved into the therapeutic implications of ginsenosides in the context of breast cancer. The bibliometric investigation highlights the burgeoning prominence of ginsenoside intervention in breast cancer research. Through co-occurrence analysis of keywords, several potential mechanisms underlying ginsenoside action in breast cancer treatment have been unveiled. Subsequently, we conducted a more comprehensive exploration of these mechanisms. Numerous studies have revealed its potential mechanisms for treating breast cancer, including the promotion of apoptosis, autophagy, inhibition of cell proliferation, EMT, and metastasis, etc. Moreover, the combination of ginsenosides and chemotherapeutic drugs showed a synergistic effect. These mechanisms involve the inhibition of malignant proliferation, infiltration growth, metastasis, and drug resistance pathological processes of breast cancer. Among them, the promoting apoptosis and inhibiting proliferation mechanism of action of ginsenosides can inhibit the pathological behavior of malignant proliferation of breast cancer throughout the development of breast cancer from occurrence to progression. The mechanisms of ginsenosides inducing breast cancer cell autophagy, inhibiting EMT, metastasis, and regulating miRNAs, lncRNAs, and epigenetic modifications may be an important pathological process in preventing breast cancer cell infiltration and metastasis. These mechanisms are closely related to the clinical treatment of breast cancer, the reduction of drug resistance, the prevention of metastasis, and the prolongation of DFS and OS. We believe that with further research, ginsenosides will be clinically applied in these areas.

Despite the progress made in the pharmacology of ginsenosides against breast cancer, there are still some issues that need deeper exploration. First, while the quantity of research publications focusing on ginsenosides for breast cancer treatment exhibits an upward trend annually, the volume of papers released per year remains relatively modest. Additionally, based on the limitations of the current bibliometrics software, this paper can only present the data in the form of graphs and charts after analyzing the literature, and it cannot present specific statistical values, such as regression coefficients, significance, and so on. Second, most current studies have been conducted in animal models and at the cellular level, but there are very few large-scale randomized controlled clinical trials. Comprehensive systematic evaluations have yet to be extensively pursued. Third, several mechanism studies have focused on a single type of ginsenoside, particularly protopanaxadiol, for instance, research on the regulation of miRNAs, lncRNAs, and epigenetic mechanisms only involved protopanaxadiol, and without investigating the role of other classes of ginsenosides. Furthermore, in terms of mechanism, there is a lack of studies on other properties of ginsenosides on breast cancer, such as angiogenesis, cellular energy metabolism, and other aspects. In conclusion, a more comprehensive investigation is essential to advance the utilization of ginsenosides in clinical breast cancer treatment.
