# Supplementary material for: Updating the therapeutic role of ginsenosides in breast cancer: a bibliometrics study to an in-depth review
Source: Front Pharmacol. 2023 Sep 25;14:1226629. doi: 10.3389/fphar.2023.1226629 (PMC10560733; doi:10.3389/fphar.2023.1226629)
Supplement: Supplementary file 1 [file Table1.DOCX]

**Table 1 Details of search strategy**

| Number | Search terms |
| --- | --- |
| 1 | Topic = ginsenosides* |
| 2 | Topic = ginsenoside* |
| 3 | Topic =breast cancer* |
| 4 | Topic =breast carcinoma* |
| 5 | LA=English |
| 6 | DT=article |
| 7 | DT=review |
| 8 | 1 OR 2 |
| 9 | 3 OR 4 |
| 10 | 8 AND 9 |
| 11 | 6 OR 7 |
| 12 | 10 AND 11 AND 5 |

Topic = title, abstract, author keywords, and keywords plus

LA=language

DT=document type
