# Supplementary material for: Updating the therapeutic role of ginsenosides in breast cancer: a bibliometrics study to an in-depth review
Source: Front Pharmacol. 2023 Sep 25;14:1226629. doi: 10.3389/fphar.2023.1226629 (PMC10560733; doi:10.3389/fphar.2023.1226629)
Supplement: Supplementary file 2 [file Table2.DOCX]

install.packages("remotes")

remotes::install_github("massimoaria/bibliometrix")

remotes::install_github("massimoaria/bibliometrixData")

install.packages("ggridges")

library(bibliometrix)

biblioshiny()

#Bibliometrix" is an R package designed for bibliometric analysis, which involves the quantitative study of publications and their citations. This package provides tools and functions to analyze scientific literature, primarily focusing on bibliographic data and citation patterns. Bibliometric analysis is commonly used in academic research, especially in fields like scientometrics, informetrics, and research evaluation.
