# Supplementary material for: Updating the therapeutic role of ginsenosides in breast cancer: a bibliometrics study to an in-depth review
Source: Front Pharmacol. 2023 Sep 25;14:1226629. doi: 10.3389/fphar.2023.1226629 (PMC10560733; doi:10.3389/fphar.2023.1226629)
Supplement: Supplementary file 3 [file DataSheet1.DOCX]

FN Clarivate Analytics Web of Science

VR 1.0

PT J

AU Wu, H

Wei, GL

Luo, LX

Li, LC

Gao, YB

Tan, XB

Wang, S

Chang, HX

Liu, YX

Wei, YJ

Song, J

Zhang, ZH

Huo, JG

AF Wu, Hao

Wei, Guoli

Luo, Lixia

Li, Lingchang

Gao, Yibo

Tan, Xiaobin

Wang, Sen

Chang, Haoxiao

Liu, Yuxi

Wei, Yingjie

Song, Jie

Zhang, Zhenhai

Huo, Jiege

TI Ginsenoside Rg3 nanoparticles with permeation enhancing based chitosan

derivatives were encapsulated with doxorubicin by thermosensitive

hydrogel and anti-cancer evaluation of peritumoral hydrogel injection

combined with PD-L1 antibody

SO BIOMATERIALS RESEARCH

LA English

DT Article

DE Chitosan; Thermo-sensitive hydrogel; Immunochemotherapy; Immunogenic

cell death; Doxorubicin; Ginsenoside Rg3

ID IMMUNOGENIC CELL-DEATH; CANCER; DRUG; EXPRESSION; DELIVERY; IMPROVE

AB Background: Combination of chemotherapy and immune checkpoint inhibitor therapy has greatly improved the anticancer effect on multiple malignancies. However, the efficiency on triple-negative breast cancer (TNBC) is limited, since most patients bear "cold " tumors with low tumor immunogenicity. Doxorubicin (DOX), one of the most effective chemotherapy agents, can induce immunogenic cell death (ICD) and thus initiating immune response.Methods: In this study, to maximize the ICD effect induced by DOX, chitosan and cell-penetrating peptide (R6F3)-modified nanoparticles (PNPs) loaded with ginsenoside Rg3 (Rg3) were fabricated using the self-assembly technique, followed by co-encapsulation with DOX based on thermo-sensitive hydrogel. Orthotopic tumor model and contralateral tumor model were established to observe the antitumor efficacy of the thermo-sensitive hydrogel combined with anti-PD-L1 immunotherapy, besides, the biocompatibility was also evaluated by histopathological.Results: Rg3-PNPs strengthened the immunogenic cell death (ICD) effect induced by DOX. Moreover, the hydrogel co-loading Rg3-PNPs and DOX provoked stronger immune response in originally nonimmunogenic 4T1 tumors than DOX monotherapy. Following combination with PD-L1 blocking, substantial antitumor effect was achieved due to the recruitment of memory T cells and the decline of adaptive PD-L1 enrichment.Conclusion: The hydrogel encapsulating DOX and highly permeable Rg3-PNPs provided an efficient strategy for remodeling immunosuppressive tumor microenvironment and converting immune "cold " 4T1 into "hot " tumors.

C1 [Wu, Hao; Wei, Guoli; Luo, Lixia; Li, Lingchang; Gao, Yibo; Tan, Xiaobin; Wang, Sen; Wei, Yingjie; Song, Jie; Zhang, Zhenhai; Huo, Jiege] Nanjing Univ Chinese Med, Affiliated Hosp Integrated Tradit Chinese & Wester, Nanjing 210023, Peoples R China.

[Wu, Hao; Wei, Guoli; Luo, Lixia; Li, Lingchang; Gao, Yibo; Tan, Xiaobin; Wang, Sen; Wei, Yingjie; Song, Jie; Zhang, Zhenhai; Huo, Jiege] Jiangsu Prov Acad Tradit Chinese Med, Nanjing 210028, Peoples R China.

[Wu, Hao; Liu, Yuxi] Chuzhou Univ, Sch Mat Sci & Chem Engn, Chuzhou 239000, Peoples R China.

[Wei, Guoli] Nanjing Lishui Dist Hosp Tradit Chinese Med, Dept Oncol, Nanjing, Peoples R China.

[Chang, Haoxiao] Capital Med Univ, Beijing Tiantan Hosp, Dept Neurol, Beijing, Peoples R China.

C3 Nanjing University of Chinese Medicine; Nanjing University of Chinese

Medicine; Chuzhou University; Capital Medical University

RP Song, J; Zhang, ZH; Huo, JG (通讯作者)，Nanjing Univ Chinese Med, Affiliated Hosp Integrated Tradit Chinese & Wester, Nanjing 210023, Peoples R China.; Song, J; Zhang, ZH; Huo, JG (通讯作者)，Jiangsu Prov Acad Tradit Chinese Med, Nanjing 210028, Peoples R China.

EM songjie_pharmacy@163.com; david23932@163.com; huojiege@jsatcm.com

FU National Natural Science Foundation of China [81873055]; Six Talent Peak

Projects in Jiangsu Province [SWXY-010-2019]; Jiangsu Province TCM

Leading Talent Training Project [SLJ0211]; Project of National Clinical

Research Base of Traditional Chinese Medicine in Jiangsu Province

[JD2019SZXYB04]; Key Research and Development Program of Anhui Province

[202104b11020010]; Chuzhou University research project [2020qd50,

2022XJYB10]

FX This work was financially supported by the National Natural Science

Foundation of China (81873055), Six Talent Peak Projects in Jiangsu

Province (SWXY-010-2019), Jiangsu Province TCM Leading Talent Training

Project (SLJ0211), Project of National Clinical Research Base of

Traditional Chinese Medicine in Jiangsu Province (JD2019SZXYB04), Key

Research and Development Program of Anhui Province (202104b11020010),

and Chuzhou University research project (2020qd50, 2022XJYB10).

CR Al-Abd AM, 2015, J CONTROL RELEASE, V219, P269, DOI 10.1016/j.jconrel.2015.08.055

Alzeibak R, 2021, J IMMUNOTHER CANCER, V9, DOI 10.1136/jitc-2020-001926

Cao DLG, 2019, ARTIF CELL NANOMED B, V47, P181, DOI 10.1080/21691401.2018.1548470

Charoen KM, 2014, BIOMATERIALS, V35, P2264, DOI 10.1016/j.biomaterials.2013.11.038

Chen C, 2019, ADV MATER, V31, DOI 10.1002/adma.201904914

Chen M, 2022, J NANOBIOTECHNOL, V20, DOI 10.1186/s12951-022-01491-w

Cunningham CR, 2021, ANN DIAGN PATHOL, V55, DOI 10.1016/j.anndiagpath.2021.151823

Fabian KP, 2021, FRONT ONCOL, V11, DOI 10.3389/fonc.2021.728018

Fang HL, 2014, CELL MOL IMMUNOL, V11, P150, DOI 10.1038/cmi.2013.59

Galon J, 2019, NAT REV DRUG DISCOV, V18, P197, DOI 10.1038/s41573-018-0007-y

Guo RR, 2016, SMALL, V12, P4541, DOI 10.1002/smll.201601094

Han JO, 2022, BIOMATER RES, V26, DOI 10.1186/s40824-022-00263-9

Haratake N, 2017, ANTICANCER RES, V37, P5713, DOI 10.21873/anticanres.12009

Hayashi K, 2020, NAT COMMUN, V11, DOI 10.1038/s41467-020-19970-9

Hegde PS, 2016, CLIN CANCER RES, V22, P1865, DOI 10.1158/1078-0432.CCR-15-1507

Hernandez S, 2021, FRONT MOL BIOSCI, V8, DOI 10.3389/fmolb.2021.667067

Hirsjarvi Samuli, 2011, Curr Drug Discov Technol, V8, P188

Hossain DMS, 2018, J CLIN INVEST, V128, P644, DOI 10.1172/JCI94586

Hu YW, 2015, J CONTROL RELEASE, V206, P91, DOI 10.1016/j.jconrel.2015.03.018

Huang LP, 2019, NAT COMMUN, V10, DOI 10.1038/s41467-019-12771-9

Jiang MX, 2021, NANOSCALE, V13, P17218, DOI 10.1039/d1nr05512g

Jiang ZS, 2017, BIOMED PHARMACOTHER, V96, P378, DOI 10.1016/j.biopha.2017.09.129

Jin C, 2021, INT J PHARMACEUT, V607, DOI 10.1016/j.ijpharm.2021.121027

Jin JK, 2021, FRONT ONCOL, V11, DOI 10.3389/fonc.2021.722916

Jin X, 2020, INT J PHARMACEUT, V589, DOI 10.1016/j.ijpharm.2020.119772

Kim S, 2022, CARBOHYD POLYM, V291, DOI 10.1016/j.carbpol.2022.119559

Kroemer G, 2013, ANNU REV IMMUNOL, V31, P51, DOI 10.1146/annurev-immunol-032712-100008

Lee JH, 2016, BIOMATERIALS, V103, P160, DOI 10.1016/j.biomaterials.2016.06.059

Li JH, 2022, CARBOHYD POLYM, V280, DOI 10.1016/j.carbpol.2021.119031

Li L, 2020, ADV FUNCT MATER, V30, DOI 10.1002/adfm.201908961

Lin H, 2021, CANCER CELL, V39, P480, DOI 10.1016/j.ccell.2020.12.023

Liu YH, 2022, BIOMATER RES, V26, DOI 10.1186/s40824-022-00260-y

Liubomirski Y, 2019, FRONT IMMUNOL, V10, DOI 10.3389/fimmu.2019.00757

Luo X, 2021, AGING-US, V13, P17177, DOI 10.18632/aging.203131

McErlean EM, 2021, J CONTROL RELEASE, V330, P1288, DOI 10.1016/j.jconrel.2020.11.037

Mei L, 2018, ACS APPL MATER INTER, V10, P17582, DOI 10.1021/acsami.8b02954

Mirzavi F, 2022, INT J PHARMACEUT, V613, DOI 10.1016/j.ijpharm.2021.121396

Najafi M, 2019, J CELL BIOCHEM, V120, P2782, DOI 10.1002/jcb.27681

Pieper AA, 2021, FRONT IMMUNOL, V12, DOI 10.3389/fimmu.2021.763888

Qiu NS, 2021, BIOMATERIALS, V269, DOI 10.1016/j.biomaterials.2020.120604

Radogna F, 2018, BIOCHEM PHARMACOL, V153, P12, DOI 10.1016/j.bcp.2018.02.006

Reardon DA, 2020, JAMA ONCOL, V6, P1003, DOI 10.1001/jamaoncol.2020.1024

Rotman J, 2020, FRONT IMMUNOL, V11, DOI 10.3389/fimmu.2020.596825

Ruoslahti E, 2017, ADV DRUG DELIVER REV, V110, P3, DOI 10.1016/j.addr.2016.03.008

Scheper W, 2019, NAT MED, V25, P89, DOI 10.1038/s41591-018-0266-5

Shen Q, 2020, ADV EXP MED BIOL, V1287, P183, DOI 10.1007/978-3-030-55031-8_12

Song WT, 2018, NAT COMMUN, V9, DOI 10.1038/s41467-018-04605-x

Sun DD, 2022, ACTA PHARM SIN B, V12, P378, DOI 10.1016/j.apsb.2021.06.005

Wang XH, 2013, J MATER CHEM B, V1, P5143, DOI 10.1039/c3tb20884b

Wang YP, 2019, CARBOHYD POLYM, V225, DOI 10.1016/j.carbpol.2019.115206

Xia JX, 2022, SCI ADV, V8, DOI 10.1126/sciadv.abj1262

Yan YY, 2018, FRONT IMMUNOL, V9, DOI 10.3389/fimmu.2018.01739

Yang H, 2021, CARBOHYD POLYM, V265, DOI 10.1016/j.carbpol.2021.118071

Yaroslavov AA, 2021, INT J BIOL MACROMOL, V177, P455, DOI 10.1016/j.ijbiomac.2021.02.169

Yu MA, 2022, BIOMATERIALS, V284, DOI 10.1016/j.biomaterials.2022.121503

Yu YL, 2021, J COLLOID INTERF SCI, V586, P391, DOI 10.1016/j.jcis.2020.10.103

Zhan JM, 2020, CARBOHYD POLYM, V230, DOI 10.1016/j.carbpol.2019.115576

Zhang C, 2022, ANGEW CHEM INT EDIT, V61, DOI 10.1002/anie.202114957

Zhang JM, 2018, CARBOHYD POLYM, V198, P537, DOI 10.1016/j.carbpol.2018.06.121

Zhang P, 2022, BIOMATERIALS, V284, DOI 10.1016/j.biomaterials.2022.121518

Zheng P, 2021, NANO LETT, V21, P2088, DOI 10.1021/acs.nanolett.0c04778

Zhuang Y, 2020, NANOSCALE, V12, P1389, DOI 10.1039/c9nr09039h

NR 62

TC 1

Z9 1

U1 21

U2 21

PU SPRINGERNATURE

PI LONDON

PA CAMPUS, 4 CRINAN ST, LONDON, N1 9XW, ENGLAND

SN 1226-4601

EI 2055-7124

J9 BIOMATER RES

JI Biomater. Res.

PD DEC 9

PY 2022

VL 26

IS 1

AR 77

DI 10.1186/s40824-022-00329-8

PG 21

WC Engineering, Biomedical; Materials Science, Biomaterials

WE Science Citation Index Expanded (SCI-EXPANDED)

SC Engineering; Materials Science

GA 7Y8RA

UT WOS:000915137900003

PM 36494759

OA Green Published, gold

DA 2023-04-05

ER

PT J

AU Farhangfar, SD

Fesahat, F

Zare-Zardini, H

Dehghan-Manshadi, M

Zare, F

Miresmaeili, SM

Vajihinejad, M

Soltaninejad, H

AF Farhangfar, Shervin Dokht

Fesahat, Farzaneh

Zare-Zardini, Hadi

Dehghan-Manshadi, Mahdi

Zare, Fateme

Miresmaeili, Seyed Mohsen

Vajihinejad, Maryam

Soltaninejad, Hossein

TI In vivo study of anticancer activity of ginsenoside Rh2-containing

arginine-reduced graphene in a mouse model of breast cancer

SO IRANIAN JOURNAL OF BASIC MEDICAL SCIENCES

LA English

DT Article

DE Arginine; Breast Cancer; Genes; Ginsenoside Rh2; Graphene; Tumor

ID MEDICINAL-PLANTS; TUMOR-CELLS; OXIDE; DOXORUBICIN; GROWTH; VITRO; RH2;

NANOPARTICLE; EXPRESSION; APOPTOSIS

AB Objective(s): This study aims to evaluate the in vivo anticancer activity of arginine-reduced graphene (Gr-Arg) and ginsenoside Rh2-containing arginine-reduced graphene (Gr-Arg-Rh2).

Materials and Methods: Thirty-two mice with breast cancer were divided into four groups and treated every three days for 32 days: Group 1, PBS, Group 2, Rh2, Group 3, Gr-Arg, and Group 4, Gr-ArgRh2. The tumor size and weight, gene expression (IL10, INF-alpha, TGF beta, and FOXP3), and pathological properties of the tumor and normal tissues were assessed.

Results: Results showed a significant decrease in TGF beta expression for all drug treatment groups compared with the controls (P=0.04). There was no significant difference among the groups regarding IL10 and FOXP3 gene expression profiles (P>0.05). Gr-Arg-Rh2 significantly inhibited tumor growth (size and weight) compared with Rh2 and control groups. The highest survival rate and the highest percentage of tumor necrosis (87.5%) belonged to the Gr-Arg-Rh2 group. Lungs showed metastasis in the control group. No metastasis was observed in the Gr-Arg-Rh2 group. Gr-Arg-Rh2 showed partial degeneration of hepatocytes and acute cell infiltration in the portal spaces and around the central vein. The Gr-Arg group experienced a moderate infiltration of acute cells into the port spaces and around the central vein. The Rh2 group also showed a mild infiltration of acute and chronic cells in portal spaces.

Conclusion: Based on the results, Gr-Arg-Rh2 can reduce tumor size, weight, and growth, TGF-beta gene expression, and increase tumor necrosis and survival time in mice with cancer.

C1 [Farhangfar, Shervin Dokht; Miresmaeili, Seyed Mohsen] Sci & Arts Univ, Dept Biol, Yazd, Iran.

[Farhangfar, Shervin Dokht; Fesahat, Farzaneh; Dehghan-Manshadi, Mahdi; Zare, Fateme] Shahid Sadoughi Univ Med Sci, Reprod Immunol Res Ctr, Yazd, Iran.

[Zare-Zardini, Hadi] Shahid Sadoughi Univ Med Sci, Hematol & Oncol Res Ctr, Yazd, Iran.

[Zare-Zardini, Hadi] Meybod Univ, Dept Biomed Engn, Meybod, Iran.

[Vajihinejad, Maryam] Shahid Sadoughi Univ Med Sci, Dept Pathol, Yazd, Iran.

[Soltaninejad, Hossein] Tarbiat Modares Univ, Fac Interdisciplinary Sci & Technol, Tehran, Iran.

C3 Tarbiat Modares University

RP Zare-Zardini, H (通讯作者)，Shahid Sadoughi Univ Med Sci, Hematol & Oncol Res Ctr, Yazd, Iran.

EM hadizarezardini@gmail.com

FU Science and Arts University; Shahid Sadoughi University of Medical

Sciences, Yazd, Iran

FX The results presented in this article were part of a student thesis.

This work was supported by the Science and Arts University and Shahid

Sadoughi University of Medical Sciences, Yazd, Iran.

CR Al-Koussa H, 2020, CANCER CELL INT, V20, DOI 10.1186/s12935-020-01232-9

Albaugh VL, 2017, J SURG ONCOL, V115, P273, DOI 10.1002/jso.24490

Mendez-Garcia LA, 2019, J INTERF CYTOK RES, V39, P39, DOI 10.1089/jir.2018.0024

Chang CM, 2021, TZU CHI MED J, V33, P203, DOI 10.4103/tcmj.tcmj_162_20

Compton OC, 2010, SMALL, V6, P711, DOI 10.1002/smll.200901934

Connolly EC, 2012, INT J BIOL SCI, V8, P964, DOI 10.7150/ijbs.4564

Dehghan-Manshadi M, 2021, INT IMMUNOPHARMACOL, V93, DOI 10.1016/j.intimp.2021.107414

Desai AG, 2008, CURR DRUG METAB, V9, P581, DOI 10.2174/138920008785821657

Dillekas H, 2019, CANCER MED-US, V8, P5574, DOI 10.1002/cam4.2474

Economopoulos SP, 2010, ACS NANO, V4, P7499, DOI 10.1021/nn101735e

Esquivel-Velazquez M, 2015, J INTERF CYTOK RES, V35, P1, DOI 10.1089/jir.2014.0026

Fang Jinbo, 2014, J Pharm Sci Pharmacol, V1, P82

Foroutan T, 2018, J BIOMED MATER RES A, V106, P293, DOI 10.1002/jbm.a.36231

Gennari A, 2005, CANCER, V104, P1742, DOI 10.1002/cncr.21359

Greenwell M, 2015, INT J PHARM SCI RES, V6, P4103, DOI 10.13040/IJPSR.0975-8232.6(10).4103-12

Gu Y, 2009, FOOD CHEM TOXICOL, V47, P2257, DOI 10.1016/j.fct.2009.06.013

Gupta A, 2020, JAMA ONCOL, V6, P308, DOI 10.1001/jamaoncol.2019.5409

Hamidullah, 2012, BREAST CANCER RES TR, V133, P11, DOI 10.1007/s10549-011-1855-x

Javanbakht S, 2018, MAT SCI ENG C-MATER, V87, P50, DOI 10.1016/j.msec.2018.02.010

Jiang XG, 2015, J ZHEJIANG UNIV-SC B, V16, P44, DOI 10.1631/jzus.B1400352

Jin LT, 2018, CANCER BIOL THER, V19, P858, DOI 10.1080/15384047.2018.1456599

Jorgovanovic D, 2020, BIOMARK RES, V8, DOI 10.1186/s40364-020-00228-x

Kocic J, 2012, EUR J CANCER, V48, P1550, DOI 10.1016/j.ejca.2011.06.043

Kotredes KP, 2013, J INTERF CYTOK RES, V33, P162, DOI 10.1089/jir.2012.0110

Kretzschmar M, 2000, BREAST CANCER RES, V2, P107, DOI 10.1186/bcr42

Liaw CC, 2015, J ONCOL, V2015, DOI 10.1155/2015/210916

Lubec B, 1996, LIFE SCI, V58, P2317, DOI 10.1016/0024-3205(96)00232-9

Martin F, 2010, ONCOGENE, V29, P4121, DOI 10.1038/onc.2010.174

Maughan KL, 2010, AM FAM PHYSICIAN, V81, P1339

Moo TA, 2018, PET CLIN, V13, P339, DOI 10.1016/j.cpet.2018.02.006

Moon IK, 2010, NAT COMMUN, V1, DOI 10.1038/ncomms1067

Naghibzadeh M, 2018, BIOINTERFACE RES APP, V8, P3048

Nakata H, 1998, JPN J CANCER RES, V89, P733, DOI 10.1111/j.1349-7006.1998.tb03278.x

Ni L, 2018, CANCER MED-US, V7, P4509, DOI 10.1002/cam4.1700

Nikpoor AR, 2017, NANOMED-NANOTECHNOL, V13, P2671, DOI 10.1016/j.nano.2017.08.010

Nounou MI, 2015, BREAST CANCER-BASIC, V9, P17, DOI 10.4137/BCBCR.S29420

Oh M, 1999, INT J ONCOL, V14, P869

Ou LL, 2017, INT J NANOMED, V12, P6633, DOI 10.2147/IJN.S140526

Patel SC, 2016, THER DELIV, V7, P101, DOI 10.4155/tde.15.93

Patil MD, 2016, ONCOGENE, V35, P4957, DOI 10.1038/onc.2016.37

Patra JK, 2018, J NANOBIOTECHNOL, V16, DOI 10.1186/s12951-018-0392-8

Pearce A, 2017, PLOS ONE, V12, DOI 10.1371/journal.pone.0184360

Pusztai L, 2004, CYTOKINE, V25, P94, DOI 10.1016/j.cyto.2003.10.004

Reers S, 2013, ANTICANCER RES, V33, P2481

Sangisetty SL, 2012, WORLD J GASTRO SURG, V4, P87, DOI 10.4240/wjgs.v4.i4.87

Sharma H, 2020, INT J MOL SCI, V21, DOI 10.3390/ijms21176280

Shewach DS, 2009, CHEM REV, V109, P2859, DOI 10.1021/cr900208x

Siegel RL, 2021, CA-CANCER J CLIN, V71, P7, DOI 10.3322/caac.21654

SreeHarsha N, 2019, INT J NANOMED, V14, P7419, DOI 10.2147/IJN.S211224

Sun Q, 2018, INT J NANOMED, V13, P3713, DOI 10.2147/IJN.S162939

Takenaka M, 2013, MOL CLIN ONCOL, V1, P625, DOI 10.3892/mco.2013.107

Tao K, 2008, BMC CANCER, V8, DOI 10.1186/1471-2407-8-228

Tian MZ, 2011, CELL SIGNAL, V23, P951, DOI 10.1016/j.cellsig.2010.10.015

Wan Y, 2021, J GINSENG RES, V45, P617, DOI 10.1016/j.jgr.2021.03.001

Yang SC, 2017, MOL CANCER, V16, DOI 10.1186/s12943-017-0700-1

Yang XX, 2017, NANOSCALE, V9, P16086, DOI 10.1039/c7nr06520e

Zare-Zardini H, 2018, J Peoples R China INST CHEM E, V93, P70, DOI 10.1016/j.jtice.2018.08.010

Zare-Zardini H, 2018, SCI REP-UK, V8, DOI 10.1038/s41598-017-18938-y

Zheng H, 2022, J GRID COMPUT, V20, DOI 10.1007/s10723-021-09594-8

NR 59

TC 0

Z9 0

U1 0

U2 0

PU MASHHAD UNIV MED SCIENCES

PI MASHHAD

PA VICE-CHANCELLOR FOR RES CTR OFF IJBMS, DANESHGAH ST, PO BOX 9138813944 -

445, MASHHAD, 00000, IRAN

SN 2008-3866

EI 2008-3874

J9 IRAN J BASIC MED SCI

JI Iran. J. Basic Med. Sci.

PD DEC

PY 2022

VL 25

IS 12

BP 1442

EP 1451

DI 10.22038/IJBMS.2022.66065.14524

PG 10

WC Medicine, Research & Experimental; Pharmacology & Pharmacy

WE Science Citation Index Expanded (SCI-EXPANDED)

SC Research & Experimental Medicine; Pharmacology & Pharmacy

GA 6P9SP

UT WOS:000891264000005

PM 36544523

DA 2023-04-05

ER

PT J

AU Jin, YJ

Nguyen, TL

Myung, CS

Heo, KS

AF Jin, Yujin

Nguyen, Thuy Le Lam

Myung, Chang-Seon

Heo, Kyung-Sun

TI Ginsenoside Rh1 protects human endothelial cells against

lipopolysaccharide-induced inflammatory injury through inhibiting TLR2/

4-mediated STAT3, NF-?B, and ER stress signaling pathways

SO LIFE SCIENCES

LA English

DT Article

DE Ginsenoside Rh1; Endothelial cell dysfunction; Lipopolysaccharide; ER

stress; STAT3; NF-?B; Human umbilical vein endothelial cells

ID ENDOPLASMIC-RETICULUM STRESS; BREAST-CANCER CELLS; MAPK;

ATHEROSCLEROSIS; APOPTOSIS; ROS

AB Aim: Endothelial cell (EC) dysfunction initiates atherosclerosis by inducing inflammatory cytokines and adhesion molecules. Herein, we investigated the role of ginsenoside Rh1 (Rh1) in lipopolysaccharide (LPS)-induced EC dysfunction.Main methods: The inhibitory effect of Rh1 on LPS binding to toll-like receptor 2 (TLR2) or TLR4 was evaluated using an immunofluorescence (IF) assay. Annexin V and cleaved caspase-3-positive EC apoptosis were evaluated by flow cytometry and IF assay. Western blotting and quantitative reverse transcription-PCR were performed to clarify underlying molecular mechanisms. In vivo model, effect of Rh1 on EC dysfunction was evaluated by using en face IF assay on aortas isolated C57BL/6 mice. Key finding: LPS (500 ng/mL) activated inflammatory signaling pathways, including ERK1/2, STAT3, and NF-kappa B. Interestingly, Rh1 significantly abolished the binding of LPS to TLR2 and TLR4. Consistently, Rh1 inhibited LPS-induced NF-kappa B activation and its downstream molecules, including inflammatory cytokines and adhesion mol-ecules. Furthermore, Rh1 alleviated LPS-induced downregulation of eNOS promoter activity. Notably, inacti-vation of eNOS by 50 mu M L-NAME significantly increased NF-& UKappa;B promoter activity. In addition, Rh1 abolished LPS-mediated cell cycle arrest and EC apoptosis by inhibiting endoplasmic reticulum stress via PERK/CHOP/ ERO1-alpha signaling pathway. Consistent with in vitro experimental data, Rh1 effectively suppressed LPS-induced VCAM-1 and CHOP expression and rescuing LPS-destroyed tight junctions between ECs as indicated in ZO-1 expression on mice aorta. Significance: Rh1 suppresses LPS-induced EC inflammation and apoptosis by inhibiting STAT3/NF-& UKappa;B and endoplasmic reticulum stress signaling pathways, mediated by blocking LPS binding-to TLR2 and TLR4. Consistently, Rh1 effectively reduced EC dysfunction in vivo model.

C1 [Jin, Yujin; Nguyen, Thuy Le Lam; Myung, Chang-Seon; Heo, Kyung-Sun] Chungnam Natl Univ, Coll Pharm, Daejeon 34134, South Korea.

[Jin, Yujin; Nguyen, Thuy Le Lam; Myung, Chang-Seon; Heo, Kyung-Sun] Chungnam Natl Univ, Inst Drug Res & Dev, Daejeon 34134, South Korea.

[Heo, Kyung-Sun] Chungnam Natl Univ, Coll Pharm, 99 Daehak Ro, Daejeon, South Korea.

[Heo, Kyung-Sun] Chungnam Natl Univ, Inst Drug Res & Dev, 99 Daehak Ro, Daejeon, South Korea.

C3 Chungnam National University; Chungnam National University; Chungnam

National University; Chungnam National University

RP Heo, KS (通讯作者)，Chungnam Natl Univ, Coll Pharm, 99 Daehak Ro, Daejeon, South Korea.; Heo, KS (通讯作者)，Chungnam Natl Univ, Inst Drug Res & Dev, 99 Daehak Ro, Daejeon, South Korea.

EM kheo@cnu.ac.kr

FU NRF of Korea [KNRF- 2019R1C1C1007331, 2022R1A2C4001776]

FX This work was supported by the NRF of Korea grant (KNRF-

2019R1C1C1007331 and 2022R1A2C4001776) .

CR Abukhdeir Abde M., 2008, Expert Reviews in Molecular Medicine, V10, P1, DOI 10.1017/S1462399408000744

Ali FEM, 2021, LIFE SCI, V276, DOI 10.1016/j.lfs.2021.119433

Andreou I, 2016, CIRCULATION, V134, P1413, DOI 10.1161/CIRCULATIONAHA.116.025129

Bailey KA, 2019, FASEB J, V33, P12888, DOI 10.1096/fj.201900236R

Chen J, 2018, FOOD FUNCT, V9, P2386, DOI [10.1039/c7fo01406f, 10.1039/C7FO01406F]

Chen Q, 2019, THERANOSTICS, V9, P6424, DOI 10.7150/thno.35528

Chen TL, 2020, MEDICINE, V99, DOI 10.1097/MD.0000000000022241

Dai JP, 2018, INT IMMUNOPHARMACOL, V54, P177, DOI 10.1016/j.intimp.2017.11.009

Deyrup ST, 2021, BIOMOL THER, V29, P105, DOI 10.4062/biomolther.2020.229

Huynh DTN, 2019, ARCH PHARM RES, V42, P848, DOI 10.1007/s12272-019-01180-7

Dong YZ, 2010, CIRCULATION, V121, P792, DOI 10.1161/CIRCULATIONAHA.109.900928

Fledderus J, 2021, J PERS MED, V11, DOI 10.3390/jpm11020103

Fu ZH, 2020, AGING-US, V12, P8640, DOI 10.18632/aging.103181

Gong LL, 2019, AM J TRANSL RES, V11, P2140

Gonzalez L, 2022, PHARMACEUTICS, V14, DOI 10.3390/pharmaceutics14071395

Heo KS, 2016, ANTIOXID REDOX SIGN, V25, P435, DOI 10.1089/ars.2015.6556

Huang NL, 2009, INT J CARDIOL, V134, P169, DOI 10.1016/j.ijcard.2008.04.010

Huynh DTN, 2021, CANCERS, V13, DOI 10.3390/cancers13081892

Huynh DTN, 2020, INT J MOL SCI, V21, DOI 10.3390/ijms21186656

Huynh DTN., 2022, CARDIOMETAB SYNDR J, V2, P96, DOI [10.51789/cmsj.2022.2.e16, DOI 10.51789/CMSJ.2022.2.E16]

Hwang HJ, 2013, PLOS ONE, V8, DOI 10.1371/journal.pone.0076679

Ishii M, 2017, BIOMED PHARMACOTHER, V91, P111, DOI 10.1016/j.biopha.2017.04.052

Jeon H, 2021, ARCH PHARM RES, V44, P702, DOI 10.1007/s12272-021-01345-3

Jin MY, 2022, LIPIDS, V57, P83, DOI 10.1002/lipd.12331

Jin YJ, 2022, ARCH PHARM RES, V45, P174, DOI 10.1007/s12272-022-01377-3

Jin YJ, 2021, INT J MOL SCI, V22, DOI 10.3390/ijms221910458

Jin Y, 2020, ARCH PHARM RES, V43, P773, DOI [10.1007/s12272-020-01265-8, 10.1007/s12272-020-01255-w]

Jin Y, 2019, BMB REP, V52, P706, DOI 10.5483/BMBRep.2019.52.12.234

Jin Yujin, 2018, Journal of Bacteriology and Virology, V48, P156, DOI 10.4167/jbv.2018.48.4.156

Kim SK, 2021, BIOMOL THER, V29, P127, DOI 10.4062/biomolther.2020.093

Le YF, 2019, TOXICOL IND HEALTH, V35, DOI 10.1177/0748233719871778

Lee D, 2019, INT J MOL SCI, V20, DOI 10.3390/ijms20246309

Li X, 2020, J INFLAMM-LOND, V17, DOI 10.1186/s12950-020-0238-7

Lim Jong-Hoon, 2009, Korean Journal of Internal Medicine, V24, P113, DOI 10.3904/kjim.2009.24.2.113

Luo PC, 2021, EUR J PHARMACOL, V893, DOI 10.1016/j.ejphar.2020.173822

Mather KJ, 2001, J AM COLL CARDIOL, V37, P1344, DOI 10.1016/S0735-1097(01)01129-9

Mathiyalagan R, 2019, MOLECULES, V24, DOI 10.3390/molecules24234367

Ming XD, 2015, LIFE SCI, V136, P36, DOI 10.1016/j.lfs.2015.06.015

Mozzini C, 2017, CURR ATHEROSCLER REP, V19, DOI 10.1007/s11883-017-0669-7

Nafisa A, 2018, PHARMACOL THERAPEUT, V192, P150, DOI 10.1016/j.pharmthera.2018.07.007

Nguyen TL, 2021, ARCH PHARM RES, V44, P241, DOI 10.1007/s12272-020-01304-4

Pan CS, 2015, PLOS ONE, V10, DOI 10.1371/journal.pone.0126640

Song DQ, 2021, MOL MED REP, V23, DOI 10.3892/mmr.2021.11906

Sun JL, 2020, AM J CHINESE MED, V48, P967, DOI 10.1142/S0192415X20500469

Thalhammer C, 1999, ARTERIOSCL THROM VAS, V19, P1173, DOI 10.1161/01.ATV.19.5.1173

Tian R, 2019, BBA-MOL BASIS DIS, V1865, P1701, DOI 10.1016/j.bbadis.2019.04.009

Woodward AM, 2020, SCI REP-UK, V10, DOI 10.1038/s41598-020-59237-3

Xiao HM, 2018, BIOMED RES INT, V2018, DOI 10.1155/2018/8475463

Zhao HK, 2021, SIGNAL TRANSDUCT TAR, V6, DOI 10.1038/s41392-021-00658-5

Zhong L, 2018, FASEB J, V32, P4070, DOI 10.1096/fj.201701536R

Zhu J, 2015, INT J ONCOL, V46, P981, DOI 10.3892/ijo.2015.2819

Zhu ML, 2021, LIFE SCI, V265, DOI 10.1016/j.lfs.2020.118855

NR 52

TC 1

Z9 1

U1 7

U2 7

PU PERGAMON-ELSEVIER SCIENCE LTD

PI OXFORD

PA THE BOULEVARD, LANGFORD LANE, KIDLINGTON, OXFORD OX5 1GB, ENGLAND

SN 0024-3205

EI 1879-0631

J9 LIFE SCI

JI Life Sci.

PD NOV 15

PY 2022

VL 309

AR 120973

DI 10.1016/j.lfs.2022.120973

PG 13

WC Medicine, Research & Experimental; Pharmacology & Pharmacy

WE Science Citation Index Expanded (SCI-EXPANDED)

SC Research & Experimental Medicine; Pharmacology & Pharmacy

GA 5M5FH

UT WOS:000871120300006

PM 36150463

DA 2023-04-05

ER

PT J

AU Zuo, ST

Wang, J

An, XQ

Wang, ZY

Zheng, X

Zhang, Y

AF Zuo, Shuting

Wang, Jing

An, Xianquan

Wang, Zhenyu

Zheng, Xiao

Zhang, Yan

TI Fabrication of Ginsenoside-Based Nanodrugs for Enhanced Antitumor

Efficacy on Triple-Negative Breast Cancer

SO FRONTIERS IN BIOENGINEERING AND BIOTECHNOLOGY

LA English

DT Article

DE triple-negative breast cancer; self-assembly; nanodrug; ginsenoside;

biomaterial

ID DELIVERY-SYSTEMS; LUNG-CANCER; CHALLENGES; RESISTANCE; CISPLATIN

AB There is an urgent need to identify chemotherapeutic agents with improved efficacy and safety against triple-negative breast cancer (TNBC). Ginsenosides can reportedly induce tumor cell death, invasion, and metastasis; however, poor water solubility, low oral absorption rate, and rapid blood clearance limit their clinical application. Utilizing the amphiphilic property of ginsenosides as building blocks of biomaterials, we fabricated a carrier-free nanodrug composed of ginsenosides Rg3 and Rb1 using a nano-reprecipitation method without any additional carriers. After characterizing and demonstrating their uniform morphology and pH-sensitive drug release properties, we observed that Rg3-Rb1 nanoparticles (NPs) exhibited stronger antitumor and anti-invasive effects on TNBCs in vitro than those mediated by free ginsenosides. Consequently, Rg3-Rb1 NPs afforded superior inhibition of tumor growth and reduction of pulmonary metastasis than the Rg3 and Rb1 mixture, with no obvious systematic toxicity in vivo. Collectively, our results provide a proof-of-concept that self-assembled engineered ginsenoside nanodrugs may be efficient and safe for TNBC therapy.

C1 [Zuo, Shuting; Wang, Jing; Wang, Zhenyu; Zhang, Yan] Jilin Univ, Hosp 2, Dept Breast Surg, Changchun, Peoples R China.

[An, Xianquan] Jilin Univ, Hosp 2, Dept Anesthesiol, Changchun, Peoples R China.

[Zheng, Xiao] South China Univ Technol, Sch Biomed Sci & Engn, Guangzhou, Peoples R China.

C3 Jilin University; Jilin University; South China University of Technology

RP Zhang, Y (通讯作者)，Jilin Univ, Hosp 2, Dept Breast Surg, Changchun, Peoples R China.; Zheng, X (通讯作者)，South China Univ Technol, Sch Biomed Sci & Engn, Guangzhou, Peoples R China.

EM zhengxiao129@scut.edu.cn; zhangy01@jlu.edu.cn

FU Scientific Development Program of Jilin Province; Finance Department

Program of Jilin Province; Guangzhou Basic and Applied Basic Research

Foundation; [20180201054YY]; [2020SCZT028]; [202102020361]

FX Funding This study was supported by the Scientific Development Program

of Jilin Province (20180201054YY), the Finance Department Program of

Jilin Province (2020SCZT028), and Guangzhou Basic and Applied Basic

Research Foundation (No. 202102020361).

CR Bianchini G, 2016, NAT REV CLIN ONCOL, V13, P674, DOI 10.1038/nrclinonc.2016.66

Chen SH, 2014, EVID-BASED COMPL ALT, V2014, DOI 10.1155/2014/168940

Cleator S, 2007, LANCET ONCOL, V8, P235, DOI 10.1016/S1470-2045(07)70074-8

Collignon J, 2016, BREAST CANCER-TARGET, V8, P93, DOI 10.2147/BCTT.S69488

Dawulieti J, 2020, SCI ADV, V6, DOI 10.1126/sciadv.aay7148

Foulkes WD, 2010, NEW ENGL J MED, V363, P1938, DOI 10.1056/NEJMra1001389

Garrido-Castro AC, 2019, CANCER DISCOV, V9, P176, DOI 10.1158/2159-8290.CD-18-1177

Hong C, 2020, NANO-MICRO LETT, V12, DOI 10.1007/s40820-020-00472-8

Hu HZ, 2021, ADV SCI, V8, DOI 10.1002/advs.202002020

Huang L, 2021, BIOMATERIALS, V268, DOI 10.1016/j.biomaterials.2020.120557

Jain V, 2020, J CONTROL RELEASE, V326, P628, DOI 10.1016/j.jconrel.2020.07.003

Jiang ZS, 2017, BIOMED PHARMACOTHER, V96, P378, DOI 10.1016/j.biopha.2017.09.129

Jin Y, 2020, ARCH PHARM RES, V43, P773, DOI [10.1007/s12272-020-01265-8, 10.1007/s12272-020-01255-w]

Kim H, 2018, J GINSENG RES, V42, P361

Kim YJ, 2015, BIOTECHNOL ADV, V33, P717, DOI 10.1016/j.biotechadv.2015.03.001

Lebert JM, 2018, CURR ONCOL, V25, pS142, DOI 10.3747/co.25.3954

Leung Kar Wah, 2010, Chin Med, V5, P20, DOI 10.1186/1749-8546-5-20

Li C, 2014, CLIN TRANSL ONCOL, V16, P593, DOI 10.1007/s12094-014-1169-7

Li L, 2017, DRUG DELIV, V24, P1617, DOI 10.1080/10717544.2017.1391893

Mei H, 2022, BIOACT MATER, V8, P220, DOI 10.1016/j.bioactmat.2021.06.035

Nedeljkovic M, 2019, CELLS-BASEL, V8, DOI 10.3390/cells8090957

Pan LL, 2019, EVID-BASED COMPL ALT, V2019, DOI 10.1155/2019/2417418

Pan WL, 2018, FITOTERAPIA, V129, P272, DOI 10.1016/j.fitote.2018.06.001

Podo F, 2010, MOL ONCOL, V4, P209, DOI 10.1016/j.molonc.2010.04.006

Qin SY, 2017, BIOMATERIALS, V112, P234, DOI 10.1016/j.biomaterials.2016.10.016

Ren ZG, 2020, SMALL, V16, DOI 10.1002/smll.201905233

Shao D, 2020, ADV MATER, V32, DOI 10.1002/adma.202004385

Shi CX, 2022, SCI ADV, V8, DOI 10.1126/sciadv.abj2372

Son KJ, 2016, IMMUNE NETW, V16, P75, DOI 10.4110/in.2016.16.1.75

Sun MD, 2019, COLLOID SURFACE B, V180, P313, DOI 10.1016/j.colsurfb.2019.04.061

Tu ZX, 2022, NAT REV MATER, V7, P557, DOI 10.1038/s41578-022-00426-z

van der Meel R, 2019, NAT NANOTECHNOL, V14, P1007, DOI 10.1038/s41565-019-0567-y

Wahba HA, 2015, CANCER BIOL MED, V12, P106, DOI 10.7497/j.issn.2095-3941.2015.0030

Wang H, 2021, J NANOBIOTECHNOL, V19, DOI 10.1186/s12951-021-01062-5

Wang JJ, 2018, CANCER LETT, V415, P73, DOI 10.1016/j.canlet.2017.11.037

Wang MZ, 2021, BIOMATER SCI-UK, V9, P8373, DOI 10.1039/d1bm01353j

Xu Y, 2022, BIOMATERIALS, V280, DOI 10.1016/j.biomaterials.2021.121077

Zhao J, 2021, CHINESE J CHEM ENG, V30, P291, DOI 10.1016/j.cjche.2020.11.012

Zheng MM, 2018, BIOMED RES INT, V2018, DOI 10.1155/2018/8174345

Zheng X, 2021, BIOMATERIALS, V271, DOI 10.1016/j.biomaterials.2021.120716

Zheng X, 2018, BIOFACTORS, V44, P496, DOI 10.1002/biof.1450

Zuo ST, 2021, FRONT BIOENG BIOTECH, V9, DOI 10.3389/fbioe.2021.747637

NR 42

TC 1

Z9 1

U1 16

U2 16

PU FRONTIERS MEDIA SA

PI LAUSANNE

PA AVENUE DU TRIBUNAL FEDERAL 34, LAUSANNE, CH-1015, SWITZERLAND

SN 2296-4185

J9 FRONT BIOENG BIOTECH

JI Front. Bioeng. Biotechnol.

PD AUG 12

PY 2022

VL 10

AR 945472

DI 10.3389/fbioe.2022.945472

PG 8

WC Biotechnology & Applied Microbiology; Multidisciplinary Sciences

WE Science Citation Index Expanded (SCI-EXPANDED)

SC Biotechnology & Applied Microbiology; Science & Technology - Other

Topics

GA 4X3WF

UT WOS:000860775800001

PM 36032706

OA gold, Green Published

DA 2023-04-05

ER

PT J

AU Xia, JX

Zhang, SY

Zhang, R

Wang, AN

Zhu, Y

Dong, MC

Ma, SJ

Hong, C

Liu, SY

Wang, D

Wang, JX

AF Xia, Jiaxuan

Zhang, Shuya

Zhang, Ru

Wang, Anni

Zhu, Ying

Dong, Meichen

Ma, Shaojie

Hong, Chao

Liu, Shengyao

Wang, Dan

Wang, Jianxin

TI Targeting therapy and tumor microenvironment remodeling of

triple-negative breast cancer by ginsenoside Rg3 based liposomes

SO JOURNAL OF NANOBIOTECHNOLOGY

LA English

DT Article

DE Triple-negative breast cancer; Ginsenoside Rg3; Liposomes; Docetaxel;

Tumor active targeting; Stroma cells; Cancer-associated fibroblasts;

Tumor microenvironment

ID NF-KAPPA-B; DELIVERY-SYSTEM; FIBROBLASTS; CHOLESTEROL; BARRIER

AB The chemotherapy effect of docetaxel (DTX) against triple-negative breast cancer (TNBC) remains mediocre and limited when encapsulated in conventional cholesterol liposomes, mainly ascribed to poor penetration and immunosuppressive tumor microenvironment (TME) caused by tumor stroma cells, especially cancer-associated fibroblasts (CAFs). Many studies have attempted to address these problems but trapped into the common dilemma of excessively complicated formulation strategies at the expense of druggability as well as clinical translational feasibility. To better address the discrepancy, ginsenoside Rg3 was utilized to substitute cholesterol to develop a multifunctional DTX-loaded Rg3 liposome (Rg3-Lp/DTX). The obtained Rg3-Lp/DTX was proved to be preferentially uptake by 4T1 cells and accumulate more at tumor site via the interaction between the glycosyl moiety of Rg3 exposed on liposome surface and glucose transporter1 (Glut1) overexpressed on tumor cells. After reaching tumor site, Rg3 was shown to reverse the activated CAFs to the resting stage and attenuate the dense stroma barrier by suppressing secretion of TGF-beta from tumor cells and regulating TGF-beta/Smad signaling. Therefore, reduced levels of CAFs and collagens were found in TME after incorporation of Rg3, inducing enhanced penetration of Rg3-Lp/DTX in the tumor and reversed immune system which can detect and neutralize tumor cells. Compared with wooden cholesterol liposomes, the smart and versatile Rg3-Lp/DTX could significantly improve the anti-tumor effect of DTX, providing a promising approach for TNBC therapy with excellent therapeutic efficacy and simple preparation process.

C1 [Xia, Jiaxuan; Zhang, Shuya; Zhang, Ru; Wang, Anni; Dong, Meichen; Liu, Shengyao; Wang, Jianxin] Fudan Univ, Sch Pharm, Dept Pharmaceut, Shanghai 201203, Peoples R China.

[Xia, Jiaxuan; Zhang, Shuya; Zhang, Ru; Wang, Anni; Dong, Meichen; Liu, Shengyao; Wang, Jianxin] Minist Educ, Key Lab Smart Drug Delivery, Shanghai 201203, Peoples R China.

[Zhu, Ying] Fudan Univ, Dept Integrat Oncol, Shanghai Canc Ctr, Shanghai 200032, Peoples R China.

[Ma, Shaojie] Huazhong Univ Sci & Technol, Coll Life Sci & Technol, Key Lab Mol Biophys, Minist Educ, Wuhan 430071, Peoples R China.

[Hong, Chao] Shanghai Univ Tradit Chinese Med, Expt Ctr Teaching & Learning, Shanghai 201203, Peoples R China.

[Wang, Dan] Xiamen Ginposome Pharmatech Co Ltd, Xiamen 361026, Peoples R China.

[Wang, Jianxin] Fudan Univ, Inst Integrat Med, Shanghai 201203, Peoples R China.

C3 Fudan University; Fudan University; Huazhong University of Science &

Technology; Shanghai University of Traditional Chinese Medicine; Fudan

University

RP Wang, JX (通讯作者)，Minist Educ, Key Lab Smart Drug Delivery, Shanghai 201203, Peoples R China.; Wang, JX (通讯作者)，Fudan Univ, Inst Integrat Med, Shanghai 201203, Peoples R China.

EM jxwang@fudan.edu.cn

FU National Natural Science Foundation of China [82074277, 8177391,

81690263]; Development Project of Shanghai Peak Disciplines-Integrated

Medicine [20180101]

FX We are thankful for financial support from the National Natural Science

Foundation of China (nos. 82074277, 8177391 and 81690263) and the

Development Project of Shanghai Peak Disciplines-Integrated Medicine

(no. 20180101).

CR Balkwill F, 2004, NAT REV CANCER, V4, P540, DOI 10.1038/nrc1388

Belfiore L, 2018, J CONTROL RELEASE, V277, P1, DOI 10.1016/j.jconrel.2018.02.040

Bianchini G, 2016, NAT REV CLIN ONCOL, V13, P674, DOI 10.1038/nrclinonc.2016.66

Bourgeois-Daigneault MC, 2018, SCI TRANSL MED, V10, DOI 10.1126/scitranslmed.aao1641

Chen C, 2022, ASIAN J PHARM SCI, V17, P219, DOI 10.1016/j.ajps.2021.12.002

Chen M, 2022, J NANOBIOTECHNOL, V20, DOI 10.1186/s12951-022-01491-w

Chen RJ, 2020, ACTA PHARMACOL SIN B, V10, P2140, DOI 10.1016/j.apsb.2020.04.005

Chen XM, 2019, NAT REV DRUG DISCOV, V18, P99, DOI 10.1038/s41573-018-0004-1

Cheng YS, 2018, CELL DEATH DIS, V9, DOI 10.1038/s41419-018-0458-4

Cvjetinovic D, 2021, J CONTROL RELEASE, V332, P301, DOI 10.1016/j.jconrel.2021.03.006

Davis ME, 2008, NAT REV DRUG DISCOV, V7, P771, DOI 10.1038/nrd2614

Deng D, 2014, NATURE, V510, P121, DOI 10.1038/nature13306

Ellerhorst JA, 1999, ONCOL REP, V6, P1097

Furuta E, 2010, BBA-REV CANCER, V1805, P141, DOI 10.1016/j.bbcan.2010.01.005

GALLAY J, 1984, BIOCHIM BIOPHYS ACTA, V769, P96, DOI 10.1016/0005-2736(84)90013-0

Halling KK, 2004, BBA-BIOMEMBRANES, V1664, P161, DOI 10.1016/j.bbamem.2004.05.006

Hong C, 2020, NANO-MICRO LETT, V12, DOI 10.1007/s40820-020-00472-8

Hong C, 2019, THERANOSTICS, V9, P4437, DOI 10.7150/thno.34953

Houthuijzen JM, 2018, CANCER METAST REV, V37, P577, DOI 10.1007/s10555-018-9768-3

Hsiao YW, 2022, CLIN TRANSL MED, V12, DOI 10.1002/ctm2.724

Hua S, 2018, FRONT PHARMACOL, V9, DOI 10.3389/fphar.2018.00790

HYSLOP PA, 1990, BIOCHEMISTRY-US, V29, P1025, DOI 10.1021/bi00456a027

Kim MK, 2017, SCI REP-UK, V7, DOI 10.1038/s41598-017-17956-0

Kim SM, 2009, ARCH PHARM RES, V32, P755, DOI 10.1007/s12272-009-1515-4

Lee SW, 2011, J CONTROL RELEASE, V155, P262, DOI 10.1016/j.jconrel.2011.06.012

Li YP, 2020, J CONTROL RELEASE, V317, P232, DOI 10.1016/j.jconrel.2019.11.031

Liu HJ, 2021, ADV FUNCT MATER, V31, DOI 10.1002/adfm.202100262

Liu Jiaye, 2022, Nat Commun, V13, P4308, DOI 10.1038/s41467-022-31928-7

Massague J, 2008, CELL, V134, P215, DOI 10.1016/j.cell.2008.07.001

Meng H, 2018, ADV DRUG DELIVER REV, V130, P50, DOI 10.1016/j.addr.2018.06.014

Miao L, 2016, ACS NANO, V10, P9243, DOI 10.1021/acsnano.6b02776

Miao L, 2015, J CONTROL RELEASE, V219, P192, DOI 10.1016/j.jconrel.2015.08.017

Monteran L, 2019, FRONT IMMUNOL, V10, DOI 10.3389/fimmu.2019.01835

Moosavian SA, 2021, SEMIN CANCER BIOL, V69, P337, DOI 10.1016/j.semcancer.2019.09.025

Mortezaee K, 2021, J BIOCHEM MOL TOXIC, V35, DOI 10.1002/jbt.22708

Pan LL, 2019, EVID-BASED COMPL ALT, V2019, DOI 10.1155/2019/2417418

Pang N, 2018, INT J NANOMED, V13, P5971, DOI 10.2147/IJN.S171224

Pei YY, 2019, SMALL, V15, DOI 10.1002/smll.201900631

Pereira BA, 2019, TRENDS CANCER, V5, P724, DOI 10.1016/j.trecan.2019.09.010

Pulaski B A, 2001, Curr Protoc Immunol, VChapter 20, DOI 10.1002/0471142735.im2002s39

Salvi AM, 2021, NAT CELL BIOL, V23, P457, DOI 10.1038/s41556-021-00677-y

Savas P, 2016, NAT REV CLIN ONCOL, V13, P228, DOI 10.1038/nrclinonc.2015.215

Sercombe L, 2015, FRONT PHARMACOL, V6, DOI 10.3389/fphar.2015.00286

Shi C, 2020, BIOMATERIALS, V233, DOI 10.1016/j.biomaterials.2020.119755

Son KJ, 2016, IMMUNE NETW, V16, P75, DOI 10.4110/in.2016.16.1.75

Sun HF, 2018, BIOMATER SCI-UK, V6, P2172, DOI 10.1039/c8bm00486b

Takai K, 2016, ONCOTARGET, V7, P82889, DOI 10.18632/oncotarget.12658

Thompson AMG, 2015, SCI REP-UK, V5, DOI 10.1038/srep12804

Vulczak A, 2020, CELLS-BASEL, V9, DOI 10.3390/cells9030628

Wang XX, 2022, NANO TODAY, V44, DOI 10.1016/j.nantod.2022.101458

Wang X, 2021, SIGNAL TRANSDUCT TAR, V6, DOI 10.1038/s41392-020-00390-6

Xia JX, 2022, SCI ADV, V8, DOI 10.1126/sciadv.abj1262

Yi XL, 2021, ADV FUNCT MATER, V31, DOI 10.1002/adfm.202010283

Yin L, 2020, BREAST CANCER RES, V22, DOI 10.1186/s13058-020-01296-5

Yuan ZG, 2017, BIOMED PHARMACOTHER, V89, P227, DOI 10.1016/j.biopha.2017.02.038

Zeltz C, 2020, SEMIN CANCER BIOL, V62, P166, DOI 10.1016/j.semcancer.2019.08.004

Zhang T, 2019, J CELL MOL MED, V23, P3711, DOI 10.1111/jcmm.14276

Zhu Y, 2021, J CONTROL RELEASE, V330, P641, DOI 10.1016/j.jconrel.2020.12.036

NR 58

TC 0

Z9 0

U1 32

U2 36

PU BMC

PI LONDON

PA CAMPUS, 4 CRINAN ST, LONDON N1 9XW, ENGLAND

EI 1477-3155

J9 J NANOBIOTECHNOL

JI J. Nanobiotechnol.

PD SEP 15

PY 2022

VL 20

IS 1

AR 414

DI 10.1186/s12951-022-01623-2

PG 22

WC Biotechnology & Applied Microbiology; Nanoscience & Nanotechnology

WE Science Citation Index Expanded (SCI-EXPANDED)

SC Biotechnology & Applied Microbiology; Science & Technology - Other

Topics

GA 4O6FC

UT WOS:000854790300002

PM 36109762

OA gold, Green Published

DA 2023-04-05

ER

PT J

AU Mohammed, HA

Almahmoud, SA

Arfeen, M

Srivastava, A

El-Readi, MZ

Ragab, EA

Shehata, SM

Mohammed, SAA

Mostafa, EM

El-khawaga, HA

Khan, RA

AF Mohammed, Hamdoon A.

Almahmoud, Suliman A.

Arfeen, Minhajul

Srivastava, Ashish

El-Readi, Mahmoud Z.

Ragab, Ehab A.

Shehata, Safia M.

Mohammed, Salman A. A.

Mostafa, Ehab M.

El-khawaga, Hend A.

Khan, Riaz A.

TI Phytochemical profiling, molecular docking, and in vitro

anti-hepatocellular carcinoid bioactivity of Suaeda vermiculata extracts

SO ARABIAN JOURNAL OF CHEMISTRY

LA English

DT Article

DE Suaeda vermiculata; Anti-cancer; Liver cancer; Hepatocellular carcinoma;

Molecular modeling; Receptor docking; HepG2; HepG-2/ADR; In vitro

activity; MTT assays

ID BREAST-CANCER CELLS; MULTIDRUG-RESISTANCE; P-GLYCOPROTEIN; ENHANCES

CHEMOSENSITIVITY; ABC TRANSPORTERS; DOXORUBICIN; EXPRESSION; QUERCETIN;

FLAVONOIDS; CHEMOTHERAPY

AB The ATP-binding cassette is the major class of transporters responsible for the efflux of chemotherapeutic agents from cancer cells, resulting in treatment failures of cancer's patients. Suaeda vermiculata Forssk. ex. J. F. Gmel. is traditionally known for its liver protective activity. The LC-MS based chemical profilings of the sequentially partitioned sub-extracts obtained from the alcoholic extract of S. vermiculata using n-hexane, chloroform, ethyl acetate, and n-butanol as fractionating solvents, identified a total of thirty six compounds. These sub-extracts were evaluated for their anti-hepatocarcinoma activity against the sensitive HepG2 and doxorubicin (DOX)-resistant, HepG-2/ADR cell lines. A mixture of doxorubicin and sub-extracts at 20 mu g/ml doses were also tested for their anti-hepatocarcinoma activity. The exhibited IC50 values for the chloroform, ethyl acetate, n-hexane, and n-butanol sub-extracts, and the doxorubicin against HepG2, and HepG-2/ADR cell lines were found at 64.5, 66.8, 81.25, 125, 1.3 mu g/ml, and 110.1, 91.82, 138.2, 265.7, 4.77 mu g/ml levels, respectively. However, the treatment of resistant cells with 20 mu g/ml of different sub-extracts in combination with the doxorubicin showed significant improvements in the doxorubicin activity against the resistant cells, and the IC50 values for DOX + chloroform, DOX + ethyl acetate, DOX + n-hexane, and DOX + n-butanol against resistant cells, were at 1.77, 2.05, 2.66, and 2.71 mu g/ml levels, respectively. The IC50 values exhibited 2.69x, 2.33x, 1.79x and 1.76x-folds reversal of the sensitivity in the resistant cancer cell lines. The molecular docking studies of the compounds identified in the LC-MS chemical profilings, against three ATP-binding cassette proteins i.e., ABCB1, ABCC1, and ABCG2, showed that flavonoids as the major class of compounds responsible for reversal of the resistant cells sensitivities. The predicted binding affinity for the flavonoids against the above mentioned three ATP-binding cassette proteins' are in the ranges of similar to-8 to -11 kcal/mol. Our results clearly indicate that the presence of flavonoids, as the major class of compounds in the S. vermiculatta is responsible for the chemosensitization of the resistant HCC-cell lines. Moreover, the structures, 21 (5-O-methyl visamminol), 22 (N-transferuloyl tyramine), 27 (atractylenolide-III), and 32 (ginsenoside-Rh2) were also identified among the potential ATP-binding cassette's modulators during the current study. These observations put the S. vermiculata in perspective with the traditionally claimed liver protective efficacy of the plant. (C) 2022 The Author(s). Published by Elsevier B.V. on behalf of King Saud University.

C1 [Mohammed, Hamdoon A.; Almahmoud, Suliman A.; Arfeen, Minhajul; Khan, Riaz A.] Qassim Univ, Coll Pharm, Dept Med Chem & Pharmacognosy, Qasim 51452, Saudi Arabia.

[Mohammed, Hamdoon A.; Ragab, Ehab A.; Mostafa, Ehab M.] Al Azhar Univ, Fac Pharm Boys, Dept Pharmacognosy & Med Plants, Cairo 11884, Egypt.

[Srivastava, Ashish] Pranveer Singh Inst Technol PSIT, Dept Pharm, Kanpur 209305, Uttar Pradesh, India.

[El-Readi, Mahmoud Z.] Umm Al Qura Univ, Fac Med, Dept Clin Biochem, Mecca 21955, Saudi Arabia.

[El-Readi, Mahmoud Z.] Al Azhar Univ, Fac Pharm, Dept Biochem, Assiut 71524, Egypt.

[Shehata, Safia M.] Ain Shams Univ Hosp, Clin Pathol Dept, Cairo, Egypt.

[Mohammed, Salman A. A.] Qassim Univ, Coll Pharm, Dept Pharmacol & Toxicol, Qasim 51452, Saudi Arabia.

[Mostafa, Ehab M.] Jouf Univ, Coll Pharm, Dept Pharmacognosy, Sakaka 72341, Aljouf, Saudi Arabia.

[El-khawaga, Hend A.] Al Azhar Univ, Fac Sci, Bot & Microbiol Dept, Girls Branch, Cairo, Egypt.

C3 Qassim University; Egyptian Knowledge Bank (EKB); Al Azhar University;

Umm Al Qura University; Egyptian Knowledge Bank (EKB); Al Azhar

University; Egyptian Knowledge Bank (EKB); Ain Shams University; Qassim

University; Al Jouf University; Egyptian Knowledge Bank (EKB); Al Azhar

University

RP Mohammed, HA; Khan, RA (通讯作者)，Qassim Univ, Coll Pharm, Dept Med Chem & Pharmacognosy, Qasim 51452, Saudi Arabia.

EM ham.mohammed@qu.edu.sa; ri.khan@qu.edu.sa

OI Srivastava, Dr. Ashish/0000-0001-9159-9466

FU Deputyship for Research & Innovation, Ministry of Education and, Saudi

Arabia [QU-IF-1-2-2]

FX The authors extend their appreciation to the Deputyship for Research &

Innovation, Ministry of Education and, Saudi Arabia for funding this

research work through the projectnumber (QU-IF-1-2-2). The authors also

thank the technical support of Qassim University.

CR Al-Omar MS, 2021, PAK J BOT, V53, P351, DOI 10.30848/PJB2021-1(9)

Al-Tohamy R, 2018, J APPL BIOMED, V16, P289, DOI 10.1016/j.jab.2018.08.001

Alam A, 2019, SCIENCE, V363, P753, DOI 10.1126/science.aav7102

Borska S, 2010, MOLECULES, V15, P857, DOI 10.3390/molecules15020857

Buchner N, 2006, RAPID COMMUN MASS SP, V20, P3229, DOI 10.1002/rcm.2720

Bugde P, 2017, EXPERT OPIN THER TAR, V21, P511, DOI 10.1080/14728222.2017.1310841

Chen C, 2010, LIFE SCI, V87, P333, DOI 10.1016/j.lfs.2010.07.004

Chen ZL, 2018, PHYTOMEDICINE, V43, P37, DOI 10.1016/j.phymed.2018.03.040

Choi HS, 2016, PHYTOTHER RES, V30, P2020, DOI 10.1002/ptr.5708

Cox J, 2016, HEPAT ONCOL, V3, DOI 10.2217/hep.15.41

Dallakyan S, 2015, METHODS MOL BIOL, V1263, P243, DOI 10.1007/978-1-4939-2269-7_19

Dohse M, 2010, DRUG METAB DISPOS, V38, P1371, DOI 10.1124/dmd.109.031302

El-Awady Raafat, 2016, Front Pharmacol, V7, P535, DOI 10.3389/fphar.2016.00535

Frion-Herrera Y, 2019, FITOTERAPIA, V136, DOI 10.1016/j.fitote.2019.104173

Jang MH, 2008, PHYTOTHER RES, V22, P544, DOI 10.1002/ptr.2406

Kalinowsky L, 2018, ACS OMEGA, V3, P5704, DOI 10.1021/acsomega.7b01194

Kim S, 2019, NUCLEIC ACIDS RES, V47, pD1102, DOI 10.1093/nar/gky1033

Kim TH, 2014, BBA-GEN SUBJECTS, V1840, P615, DOI 10.1016/j.bbagen.2013.10.023

Kumar A, 2019, EUR J MED CHEM, V176, P268, DOI 10.1016/j.ejmech.2019.05.027

Le Grazie M, 2017, WORLD J HEPATOL, V9, P907, DOI 10.4254/wjh.v9.i21.907

Lee WYW, 2010, J NAT PROD, V73, P854, DOI 10.1021/np900792p

Li K, 2017, BIOMED PHARMACOTHER, V96, P371, DOI 10.1016/j.biopha.2017.10.016

Li SZ, 2018, BIOMED PHARMACOTHER, V100, P441, DOI 10.1016/j.biopha.2018.02.055

Li Y., 2011, ALTERN MED, V2011

Li Y, 2013, EXPERT OPIN DRUG MET, V9, P267, DOI 10.1517/17425255.2013.749858

Li ZH, 2016, J CHROMATOGR SCI, V54, P805, DOI 10.1093/chromsci/bmw016

Longley DB, 2005, J PATHOL, V205, P275, DOI 10.1002/path.1706

March RE, 2004, INT J MASS SPECTROM, V231, P157, DOI 10.1016/j.ijms.2003.10.008

Mohammed HA, 2021, PLANTS-BASEL, V10, DOI 10.3390/plants10091811

Mohammed Hamdoon A., 2020, Central Nervous System Agents in Medicinal Chemistry, V20, P122, DOI 10.2174/1871524920666200319142536

Mohammed HA, 2019, J ESSENT OIL BEAR PL, V22, P82, DOI [10.1080/0972060X.2019.1574611, 10.1080/0972060x.2019.1574611]

Mohammed HA, 2019, MOLECULES, V24, DOI 10.3390/molecules24081501

Mohammed SAA, 2020, PLANTS-BASEL, V9, DOI 10.3390/plants9101291

Mohana S, 2016, MOL BIOSYST, V12, P2458, DOI 10.1039/c6mb00187d

Morris GM, 2009, J COMPUT CHEM, V30, P2785, DOI 10.1002/jcc.21256

Morris ME, 2006, LIFE SCI, V78, P2116, DOI 10.1016/j.lfs.2005.12.003

Ng IOL, 2000, AM J CLIN PATHOL, V113, P355, DOI 10.1309/AC1M-4TY4-U0TN-EN7T

Nies AT, 2001, INT J CANCER, V94, P492, DOI 10.1002/ijc.1498

O'Boyle NM, 2011, J CHEMINFORMATICS, V3, DOI 10.1186/1758-2946-3-33

Orlando BJ, 2020, NAT COMMUN, V11, DOI 10.1038/s41467-020-16155-2

Shen D, 2021, ACS OMEGA, V6, P32005, DOI 10.1021/acsomega.1c04779

Silverman J A, 1995, Prog Liver Dis, V13, P101

Szakacs G, 2006, NAT REV DRUG DISCOV, V5, P219, DOI 10.1038/nrd1984

Trott O, 2010, J COMPUT CHEM, V31, P455, DOI 10.1002/jcc.21334

Wang C, 2021, INT J PEPT RES THER, V27, P2291, DOI 10.1007/s10989-021-10253-5

Wang X., 2022, PHARMACOL RES

Wink M, 2012, FRONT MICROBIOL, V3, DOI 10.3389/fmicb.2012.00130

Wu CP, 2021, J NAT PROD, V84, P2544, DOI 10.1021/acs.jnatprod.1c00584

Xing Y, 2014, J DIGEST DIS, V15, P246, DOI 10.1111/1751-2980.12131

Yuan ZH, 2015, NUTR CANCER, V67, P126, DOI 10.1080/01635581.2015.965334

NR 50

TC 5

Z9 5

U1 3

U2 4

PU ELSEVIER

PI AMSTERDAM

PA RADARWEG 29, 1043 NX AMSTERDAM, NETHERLANDS

SN 1878-5352

EI 1878-5379

J9 ARAB J CHEM

JI Arab. J. Chem.

PD JUL

PY 2022

VL 15

IS 7

AR 103950

DI 10.1016/j.arabjc.2022.103950

EA MAY 2022

PG 13

WC Chemistry, Multidisciplinary

WE Science Citation Index Expanded (SCI-EXPANDED)

SC Chemistry

GA 4K9AC

UT WOS:000852231900009

OA gold

DA 2023-04-05

ER

PT J

AU Langeh, U

Kumar, V

Singh, C

Singh, A

AF Langeh, Urvashi

Kumar, Vishal

Singh, Charan

Singh, Arti

TI Drug-herb combination therapy in cancer management

SO MOLECULAR BIOLOGY REPORTS

LA English

DT Review

DE Cancer; Chemotherapy; Clinical; Herbal drugs; Phytochemistry; Side

effects

ID HUMAN BREAST-CANCER; FACTOR-KAPPA-B; GINSENOSIDE RG3; P-GLYCOPROTEIN;

ORAL BIOAVAILABILITY; CELL APOPTOSIS; CURCUMIN; CHEMOTHERAPY;

PACLITAXEL; CHEMISTRY

AB Cancer is the second leading cause of fatality all over the world. Various unwanted side effects are being reported with the use of conventional chemotherapy. The plant derived bioactive compounds are the prominent alternative medicinal approach for reduction of chemotherapy associated side effects. The data is collected from Pubmed, Sci-hub, Google scholar, and Research gate were systematically searched up to year 2020. Several herbal drugs have been investigated and found with grateful anti-cancer potentials hence, it can be used in combination with chemotherapy for the depletion of associated side-effects. Herbal drugs and their extracts contain a mixture of active ingredients, which show interactions within themselves and along with chemotherapeutic agents to show either synergistic or antagonistic therapeutic effects. Therefore, it is necessary to develop alternative treatment to control chemotherapy associated side-effects. In this review, we discussed some of the significant chemical compounds, which could be efficient against cancer. This review focuses on the different herbal drugs that play an important role in the treatment of cancer and its associated side-effects. This study aimed to evaluate the efficacy of herbal treatment in combination with chemotherapy for cancer treatment.

C1 [Langeh, Urvashi; Kumar, Vishal; Singh, Charan; Singh, Arti] ISF Coll Pharm, Dept Pharmacol, Moga 142001, Punjab, India.

[Langeh, Urvashi; Kumar, Vishal; Singh, Charan; Singh, Arti] IK Gujral Punjab Tech Univ, Jalandhar 144603, Punjab, India.

C3 ISF College of Pharmacy; I. K. Gujral Punjab Technical University

RP Singh, A (通讯作者)，ISF Coll Pharm, Dept Pharmacol, Moga 142001, Punjab, India.; Singh, A (通讯作者)，IK Gujral Punjab Tech Univ, Jalandhar 144603, Punjab, India.

EM artisingh@isfcp.org

RI Singh, Arti/HLX-4417-2023; Singh, Charan/GZL-3630-2022; Kumar,

Vishal/AFU-8315-2022

OI Singh, Charan/0000-0002-1630-3183; Kumar, Vishal/0000-0003-3615-9374; ,

Arti/0000-0001-5168-4930

FU DST SERB [EEQ/2019/000690]

FX This work was supported by DST SERB under EEQ grant with File No.

EEQ/2019/000690.

CR Abotaleb M, 2019, CANCERS, V11, DOI 10.3390/cancers11010028

Abu Samaan TM, 2019, BIOMOLECULES, V9, DOI 10.3390/biom9120789

Aggarwal BB, 2005, CLIN CANCER RES, V11, P7490, DOI 10.1158/1078-0432.CCR-05-1192

Ali BH, 2006, NAT PROD COMMUN, V1, P509

Allemani C, 2017, CANCER-AM CANCER SOC, V123, P4977, DOI 10.1002/cncr.30854

[Anonymous], 2008, CHIN J INTEGR MED, V14, P33, DOI [10.1007/s11655-007-9002-6, 10.1007/s11655-007-9002]

Bae JW, 2011, INFLAMM RES, V60, P751, DOI 10.1007/s00011-011-0330-9

Balasubramanyam K, 2004, J BIOL CHEM, V279, P33716, DOI 10.1074/jbc.M402839200

Barton DL, 2010, SUPPORT CARE CANCER, V18, P179, DOI 10.1007/s00520-009-0642-2

Batra P, 2013, 3 BIOTECH, V3, P439, DOI 10.1007/s13205-013-0117-5

Ben-Shabat S, 2020, DRUG DELIV TRANSL RE, V10, P354, DOI 10.1007/s13346-019-00691-6

Bimonte S, 2016, NUTRIENTS, V8, DOI 10.3390/nu8070433

Bonam SR, 2018, CHEMMEDCHEM, V13, P1854, DOI 10.1002/cmdc.201800343

Brusselmans K, 2005, J BIOL CHEM, V280, P5636, DOI 10.1074/jbc.M408177200

Chan JY, 2008, CANCER BIOL THER, V7, P1305, DOI 10.4161/cbt.7.8.6302

Chatfield K, 2018, EVID-BASED COMPL ALT, V2018, DOI 10.1155/2018/1903629

Chen S, 2014, EVID-BASED COMPL ALT, V2014, DOI DOI 10.1155/2014/168940

Cheng YY, 2018, J FOOD DRUG ANAL, V26, pS88, DOI 10.1016/j.jfda.2018.01.003

Chiu TL, 2009, INT J MOL MED, V23, P469, DOI 10.3892/ijmm_00000153

Choi CH, 2003, PLANTA MED, V69, P235, DOI 10.1055/s-2003-38483

Christensen LP, 2009, ADV FOOD NUTR RES, V55, P1, DOI 10.1016/S1043-4526(08)00401-4

Delmas D, 2020, MOLECULES, V25, DOI 10.3390/molecules25092009

Desai AG, 2008, CURR DRUG METAB, V9, P581, DOI 10.2174/138920008785821657

DEVITA VT, 1975, CANCER, V35, P98, DOI 10.1002/1097-0142(197501)35:1<98::AID-CNCR2820350115>3.0.CO;2-B

Farsad-Naeimi A, 2018, FOOD FUNCT, V9, P2025, DOI [10.1039/C7FO01898C, 10.1039/c7fo01898c]

Fasinu PS, 2019, FRONT ONCOL, V9, DOI 10.3389/fonc.2019.01356

Fu BW, 2018, FRONT PHARMACOL, V9, DOI 10.3389/fphar.2018.01394

Ganji-Harsini S, 2016, CELL J, V18

George VC, 2017, J NUTR BIOCHEM, V45, P1, DOI 10.1016/j.jnutbio.2016.11.007

Ghafari Fereshteh, 2017, Asian Pac J Cancer Prev, V18, P795

Ghalaut Veena S, 2012, J Oncol Pharm Pract, V18, P186, DOI 10.1177/1078155211416530

Giovannucci E, 1999, HORM RES, V51, P34, DOI 10.1159/000053160

Goey AKL, 2013, BRIT J CLIN PHARMACO, V76, P467, DOI 10.1111/bcp.12159

Hamed AR, 2019, B NATL RES CENT, V43, P8, DOI [10.1186/s42269-019-0043-8, DOI 10.1186/S42269-019-0043-8]

Hassan, 2020, MED PLANTS USE PREVE

Heim KE, 2002, J NUTR BIOCHEM, V13, P572, DOI 10.1016/S0955-2863(02)00208-5

Hemalswarya S, 2006, PHYTOTHER RES, V20, P239, DOI 10.1002/ptr.1841

Hoensch H, 2008, WORLD J GASTROENTERO, V14, P2187, DOI 10.3748/wjg.14.2187

Huang CS, 2007, J NUTR BIOCHEM, V18, P449, DOI 10.1016/j.jnutbio.2006.08.007

INVERNIZZI R, 1993, HAEMATOLOGICA, V78, P340

Ireson C, 2001, CANCER RES, V61, P1058

Jakubowicz-Gil J, 2013, TUMOR BIOL, V34, P2367, DOI 10.1007/s13277-013-0785-0

Jeong JS., 2018, SCI REP, V8, P1, DOI DOI 10.1038/s41598-017-17765-5

Kahraman C., 2020, MED TOXICOL, P1

Kale A, 2008, PHYTOTHER RES, V22, P567, DOI 10.1002/ptr.2283

Khan Arif, 2019, Asian Pac J Cancer Prev, V20, P1153, DOI 10.31557/APJCP.2019.20.4.1153

Kim Mi Jung, 2015, J Cancer Prev, V20, P92, DOI 10.15430/JCP.2015.20.2.92

Kim SM, 2010, EUR J PHARMACOL, V631, P1, DOI 10.1016/j.ejphar.2009.12.018

Kim SM, 2009, ARCH PHARM RES, V32, P755, DOI 10.1007/s12272-009-1515-4

Koohpar ZK, 2015, IRAN J CANCER PREV, V8, DOI 10.17795/ijcp2331

Kotb MG, 2019, EGYPT J NEUROL PSYCH, V55, DOI 10.1186/s41983-019-0104-9

Kumar S, 2013, SCI WORLD J, DOI 10.1155/2013/162750

Laskar Y.B., 2020, MED PLANTS USE PREVE

Lee CK, 2010, PHARMACOLOGY, V85, P350, DOI 10.1159/000312690

Lephart Edwin D, 2015, Enzyme Res, V2015, P594656, DOI 10.1155/2015/594656

Li SZ, 2013, ANTI-CANCER AGENT ME, V13, P352, DOI 10.2174/1871520611313020020

Lin SR, 2020, BRIT J PHARMACOL, V177, P1409, DOI 10.1111/bph.14816

Liu CQ, 2015, CANCER LETT, V362, P8, DOI 10.1016/j.canlet.2015.03.019

Liu DW, 2013, J BREAST CANCER, V16, P133, DOI 10.4048/jbc.2013.16.2.133

Liu JJ, 2011, CANCER LETT, V300, P105, DOI 10.1016/j.canlet.2010.10.001

Lu JM, 2009, CURR VASC PHARMACOL, V7, P293, DOI 10.2174/157016109788340767

Mahmoodnia Leila, 2017, J Nephropathol, V6, P144, DOI 10.15171/jnp.2017.25

Mangla B., 2018, INT J DRUG DEV RES, V10, P22

Mansouri K, 2020, BMC CANCER, V20, DOI 10.1186/s12885-020-07256-8

Mason JK, 2015, J NUTR BIOCHEM, V26, P16, DOI 10.1016/j.jnutbio.2014.08.001

Mekhail Tarek M, 2002, Expert Opin Pharmacother, V3, P755

Muldoon LL, 2007, J CLIN ONCOL, V25, P2295, DOI 10.1200/JCO.2006.09.9861

Nelson KM, 2017, J MED CHEM, V60, P1620, DOI 10.1021/acs.jmedchem.6b00975

Nishida N, 2006, VASC HEALTH RISK MAN, V2, P213, DOI 10.2147/vhrm.2006.2.3.213

Ono Misaki, 2015, Enzymes, V37, P139, DOI 10.1016/bs.enz.2015.06.002

Padhye S, 2009, J HEMATOL ONCOL, V2, DOI 10.1186/1756-8722-2-38

Panche AN, 2016, J NUTR SCI, V5, DOI 10.1017/jns.2016.41

Peng DC, 2012, CHIN MED-UK, V7, DOI [10.1186/1749-8546-7-23, 10.1186/1749-8546-7-2]

Piantino CB, 2009, INT BRAZ J UROL, V35, P354, DOI 10.1590/S1677-55382009000300012

Pourmohamadi K, 2018, INT J HEMATOL ONCOL, V12, P313, DOI DOI 10.18502/IJHOSCR.V12I4.110

Roy M, 2011, THER DELIV, V2, P1275, DOI 10.4155/TDE.11.97

Saadat N, 2012, J ONCOL, V2012, DOI 10.1155/2012/647206

Santandreu FM, 2011, CELL PHYSIOL BIOCHEM, V28, P219, DOI 10.1159/000331733

Schroder L, 2019, ONCOL REP, V41, P387, DOI 10.3892/or.2018.6789

Seretny M, 2014, PAIN, V155, P2461, DOI 10.1016/j.pain.2014.09.020

Shahid U., 2013, OMICS GROUP EBOOKS, P1

Shan X, 2014, PLOS ONE, V9, DOI 10.1371/journal.pone.0115401

Shi J, 2000, CRIT REV FOOD SCI, V40, P1, DOI 10.1080/10408690091189275

Sparreboom A, 1997, P NATL ACAD SCI USA, V94, P2031, DOI 10.1073/pnas.94.5.2031

Srivastava S, 2015, J FOOD SCI TECH MYS, V52, P41, DOI 10.1007/s13197-012-0918-2

Sun SY, 2004, JNCI-J NATL CANCER I, V96, P662, DOI 10.1093/jnci/djh123

Tan BL, 2019, MOLECULES, V24, DOI 10.3390/molecules24142527

Tang WP, 2013, ACS SYM SER, V1129, P133

Thakur VS, 2014, AAPS J, V16, P151, DOI 10.1208/s12248-013-9548-5

Tu SH, 2017, FOOD FUNCT, V8, P1067, DOI 10.1039/c6fo01588c

Vinod BS, 2013, CELL DEATH DIS, V4, DOI 10.1038/cddis.2013.26

Wang L, 2015, MOL MED REP, V12, P609, DOI 10.3892/mmr.2015.3397

Wang ZJ, 2014, PHYTOCHEM REV, V13, P323, DOI 10.1007/s11101-013-9327-z

Wilken R, 2011, MOL CANCER, V10, DOI 10.1186/1476-4598-10-12

Wong AST, 2015, NAT PROD REP, V32, P256, DOI 10.1039/c4np00080c

Yang LQ, 2012, BIOPHARM DRUG DISPOS, V33, P425, DOI 10.1002/bdd.1806

Yu Y, 2007, CANCER-AM CANCER SOC, V109, P2374, DOI 10.1002/cncr.22659

Zhan YZ, 2014, ARCH PHARM RES, V37, P1086, DOI 10.1007/s12272-013-0311-3

Zhang HC, 2017, SCI REP-UK, V7, DOI [10.1038/srep39911, 10.1038/srep40819, 10.1038/srep43163]

Zhang J, 2020, DOSE-RESPONSE, V18, DOI 10.1177/1559325820926732

NR 100

TC 0

Z9 0

U1 7

U2 8

PU SPRINGER

PI DORDRECHT

PA VAN GODEWIJCKSTRAAT 30, 3311 GZ DORDRECHT, NETHERLANDS

SN 0301-4851

EI 1573-4978

J9 MOL BIOL REP

JI Mol. Biol. Rep.

PD NOV

PY 2022

VL 49

IS 11

BP 11009

EP 11024

DI 10.1007/s11033-022-07861-9

EA SEP 2022

PG 16

WC Biochemistry & Molecular Biology

WE Science Citation Index Expanded (SCI-EXPANDED)

SC Biochemistry & Molecular Biology

GA 5U5OK

UT WOS:000852118000001

PM 36083521

DA 2023-04-05

ER

PT J

AU Park, JE

Ji, HW

Kim, HW

Baek, M

Jung, S

Kim, SJ

AF Park, Jae Eun

Ji, Hwee Won

Kim, Hyeon Woo

Baek, Minjae

Jung, Sanghyun

Kim, Sun Jung

TI Ginsenoside Rh2 Regulates the CFAP20DC-AS1/MicroRNA-3614-3p/BBX and

TNFAIP3 Axis to Induce Apoptosis in Breast Cancer Cells

SO AMERICAN JOURNAL OF CHINESE MEDICINE

LA English

DT Article

DE Breast Cancer; Competitive Endogenous RNA; Ginsenoside; Long Noncoding

RNA; MicroRNA

ID ROLES

AB While a number of coding genes have explained the anticancer activity of ginsenoside Rh2, little is known about noncoding RNAs. This study was performed to elucidate the regulatory activity of long noncoding RNA (lncRNA) CFAP20DC-AS1, which is known to be downregulated by Rh2. MiR-3614-3p, which potentially binds CFAP20DC-AS1, was screened using the LncBase Predicted program, and the binding was verified by assaying the luciferase activity of a luciferase/lncRNA recombinant plasmid construct. The competitive endogenous RNA (ceRNA) relationship of the two RNAs was further validated by quantitative PCR after deregulation of each RNA using siRNA. The effect of miRNA and target genes on the MCF-7 cancer cell growth was determined by monitoring proliferation and apoptosis in the presence of Rh2 after deregulating the corresponding gene. The miRNA decreased the luciferase activity of the luciferase/CFAP20DC-AS1 fusion vector, confirming the binding. SiRNA-based deregulation of CFAP20DC-AS1 attenuated the expression of miR-3614-3p and vice versa. In contrast to CFAP20DC-AS1, miR-3614-3p was upregulated by Rh2, inhibiting proliferation but stimulating apoptosis of the MCF-7 cells. Target genes of miR-3614-3p, BBX and TNFAIP3, were downregulated by Rh2 and the miRNA but upregulated by the lncRNA. Rh2 inhibits CFAP20DC-AS1, which obscures the association of the lncRNA with miR-3614-3p, resulting in the suppression of oncogenic BBX and TNFAIP3. Taken together, the Rh2/CFAP20DC-AS1/miR-3614-3p/target gene axis contributes to the antiproliferation activity of Rh2 in cancer cells.

C1 [Park, Jae Eun; Ji, Hwee Won; Kim, Hyeon Woo; Baek, Minjae; Jung, Sanghyun; Kim, Sun Jung] Dongguk Univ Seoul, Dept Life Sci, 32 Dongguk Ro, Goyang 10326, South Korea.

C3 Dongguk University

RP Kim, SJ (通讯作者)，Dongguk Univ Seoul, Dept Life Sci, 32 Dongguk Ro, Goyang 10326, South Korea.

EM sunjungk@dongguk.edu

RI kim, sun jung/T-1013-2019

FU Korean Society of Ginseng; National Research Foundation of Korea -

Ministry of Education, Science, and Technology [NRF-2016R1D1A1B01009235]

FX This work was supported by a grant from the Korean Society of Ginseng

(2018); and by the Basic Science Research Program

(NRF-2016R1D1A1B01009235) of the National Research Foundation of Korea,

funded by the Ministry of Education, Science, and Technology.

CR Chan JJ, 2018, INT J MOL SCI, V19, DOI 10.3390/ijms19051310

Chen C, 2021, MOLECULES, V26, DOI 10.3390/molecules26133926

Chen TL, 2014, DEV GENES EVOL, V224, P261, DOI 10.1007/s00427-014-0476-x

Chen WW, 2019, J ASIAN NAT PROD RES, V21, P742, DOI 10.1080/10286020.2018.1490273

Chen WW, 2018, ONCOL LETT, V16, P5367, DOI 10.3892/ol.2018.9235

Chen YY, 2019, ANTI-CANCER AGENT ME, V19, P1633, DOI 10.2174/1871520619666190704165205

Choi S, 2011, J CELL BIOCHEM, V112, P330, DOI 10.1002/jcb.22932

Dong B, 2017, CELL MOL BIOL, V63, P1, DOI 10.14715/cmb/2017.63.8.1

Gao QR, 2018, CELL PROLIFERAT, V51, DOI 10.1111/cpr.12438

Hayes EL, 2015, BREAST CANCER RES, V17, DOI 10.1186/s13058-015-0542-y

Hou J, 2020, PHYTOTHER RES, V34, P1659, DOI 10.1002/ptr.6636

Jeong D, 2021, AM J CANCER RES, V11, P2568

Jeong D, 2019, AM J CHINESE MED, V47, P1643, DOI 10.1142/S0192415X19500848

Jin X, 2016, J GINSENG RES, V40, P269, DOI 10.1016/j.jgr.2015.08.007

Kim H, 2021, BIOMOLECULES, V11, DOI 10.3390/biom11010118

Klinge Carolyn M., 2018, Non-Coding RNA, V4, P40, DOI 10.3390/ncrna4040040

Lee H, 2018, J GINSENG RES, V42, P455, DOI 10.1016/j.jgr.2017.05.003

Lee S, 2017, FREE RADICAL BIO MED, V110, P280, DOI 10.1016/j.freeradbiomed.2017.06.017

Li M, 2019, EXP MOL PATHOL, V108, P17, DOI 10.1016/j.yexmp.2019.03.004

Oh M, 1999, INT J ONCOL, V14, P869

Rashid F, 2016, GENOM PROTEOM BIOINF, V14, P73, DOI 10.1016/j.gpb.2016.03.005

Shi YY, 2021, CANCER LETT, V511, P26, DOI 10.1016/j.canlet.2021.04.017

Sun XD, 2021, J ONCOL, V2021, DOI 10.1155/2021/9715154

Wang YS, 2021, INT J MOL SCI, V22, DOI 10.3390/ijms22179289

Wang ZZ, 2019, EBIOMEDICINE, V41, P357, DOI 10.1016/j.ebiom.2018.12.061

Yun M, 2020, J GINSENG RES, V44, P373, DOI 10.1016/j.jgr.2019.12.006

Zhu SR, 2021, J GINSENG RES, V45, P295, DOI 10.1016/j.jgr.2020.05.001

NR 27

TC 2

Z9 2

U1 5

U2 5

PU WORLD SCIENTIFIC PUBL CO PTE LTD

PI SINGAPORE

PA 5 TOH TUCK LINK, SINGAPORE 596224, SINGAPORE

SN 0192-415X

EI 1793-6853

J9 AM J CHINESE MED

JI Am. J. Chin. Med.

PY 2022

VL 50

IS 06

BP 1703

EP 1717

DI 10.1142/S0192415X22500720

PG 15

WC Integrative & Complementary Medicine; Medicine, General & Internal

WE Science Citation Index Expanded (SCI-EXPANDED)

SC Integrative & Complementary Medicine; General & Internal Medicine

GA 3U6CT

UT WOS:000841057700013

PM 35787669

DA 2023-04-05

ER

PT J

AU Peng, KJ

Luo, T

Li, JJ

Huang, JJ

Dong, ZZ

Liu, J

Pi, CQ

Zou, ZZ

Gu, Q

Liu, OS

Zhang, JT

Luo, ZY

AF Peng, Kunjian

Luo, Tiao

Li, Jijia

Huang, Jingjia

Dong, Zizeng

Liu, Jia

Pi, Chaoqiong

Zou, Zizeng

Gu, Qin

Liu, Ousheng

Zhang, Jian-Ting

Luo, Zhi-Yong

TI Ginsenoside Rh2 inhibits breast cancer cell growth via ER beta-TNF alpha

pathway

SO ACTA BIOCHIMICA ET BIOPHYSICA SINICA

LA English

DT Article

DE ginsenoside Rh2; estrogen receptor; TNF alpha; breast cancer; apoptosis

ID ESTROGEN-RECEPTOR-BETA; 20(S)-GINSENOSIDE RH2; CARCINOMA CELLS;

PROLIFERATION; APOPTOSIS; ANTAGONIST; ACTIVATION; MECHANISM; HEALTH;

SIGNAL

AB Ginsenoside Rh2 is one of rare panaxidiols extracted from Panax ginseng and a potential estrogen receptor ligand that exhibits moderate estrogenic activity. However, the effect of Rh2 on growth inhibition and its underlying molecular mechanism in human breast cells are not fully understood. In this study, we tested cell viability by MTT and colony formation assays. Cell growth and cell cycle were determined to investigate the effect of ginsenoside Rh2 by flow cytometry. The expressions of estrogen receptors (ERs), TNF alpha, and apoptosis-related proteins were detected by qPCR and western blot analysis. The mechanisms of ER alpha and ER beta action were determined using transfection and inhibitors. Antitumor effect of ginsenoside Rh2 against MCF-7 cells was investigated in xenograft mice. Our results showed that ginsenoside Rh2 induced apoptosis and G1/S phase arrest in MCF-7 cells. Treatment of cells with ginsenoside Rh2 down-regulated protein levels of ER alpha, and up-regulated mRNA and protein levels of ER beta and TNF alpha. We also found that ginsenoside Rh2-induced TNF alpha over-expression is through up-regulation of ER beta initiated by ginsenoside Rh2. Furthermore, ginsenoside Rh2 induced MCF-7 cell apoptosis via estrogen receptor beta-TNF alpha pathway in vivo. These results demonstrate that ginsenoside Rh2 promotes TNF alpha-induced apoptosis and G1/S phase arrest via regulation of ER beta.

C1 [Peng, Kunjian; Huang, Jingjia; Liu, Jia; Pi, Chaoqiong; Zou, Zizeng; Gu, Qin; Luo, Zhi-Yong] Cent South Univ, Dept Biochem & Mol Biol, Hunan Prov Key Lab Bas & Appl Hematol, Hunan Key Lab Anim Models Human Dis,Sch Life Sci, Changsha 410008, Peoples R China.

[Dong, Zizeng; Zhang, Jian-Ting] Univ Toledo, Coll Med & Life Sci, Dept Cell & Canc Biol, Toledo, OH 43614 USA.

[Luo, Tiao; Liu, Ousheng] Cent South Univ, Hunan Key Lab Oral Hlth Res, Changsha 410008, Peoples R China.

[Luo, Tiao; Liu, Ousheng] Cent South Univ, Xiangya Stomatol Hosp, Changsha 410008, Peoples R China.

[Luo, Tiao; Liu, Ousheng] Cent South Univ, Xiangya Sch Stomatol, Changsha 410008, Peoples R China.

[Li, Jijia] Cent South Univ, Ctr Stomatol, Xiangya Hosp, Changsha 410008, Peoples R China.

C3 Central South University; University System of Ohio; University of

Toledo; Central South University; Central South University; Central

South University; Central South University

RP Luo, ZY (通讯作者)，Cent South Univ, Dept Biochem & Mol Biol, Hunan Prov Key Lab Bas & Appl Hematol, Hunan Key Lab Anim Models Human Dis,Sch Life Sci, Changsha 410008, Peoples R China.; Zhang, JT (通讯作者)，Univ Toledo, Coll Med & Life Sci, Dept Cell & Canc Biol, Toledo, OH 43614 USA.; Liu, OS (通讯作者)，Cent South Univ, Hunan Key Lab Oral Hlth Res, Changsha 410008, Peoples R China.; Liu, OS (通讯作者)，Cent South Univ, Xiangya Stomatol Hosp, Changsha 410008, Peoples R China.; Liu, OS (通讯作者)，Cent South Univ, Xiangya Sch Stomatol, Changsha 410008, Peoples R China.

EM liuousheng@163.com; JianTing.Zhang@UToledo.edu; luozhiyong@csu.edu.cn

FU National Natural Science Foundation of China [81973710, 81903107,

81874332, 81673544]; Hunan Provincial Natural Science Foundation of

China [2021JJ30902, 2020JJ8052]; Health Commission Key Program of Hunan

Province [202102021395]; Hunan Provincial Key Program for Research and

Development [2018SK21310]; Graduate research innovation project of

Central South University [2018zzts118, 1053320192015]

FX This work was supported by the grants from the National Natural Science

Foundation of China (Nos. 81973710, 81903107, 81874332 and 81673544),

the Hunan Provincial Natural Science Foundation of China (Nos.

2021JJ30902 and 2020JJ8052), the Health Commission Key Program of Hunan

Province (No. 202102021395), the Hunan Provincial Key Program for

Research and Development (No. 2018SK21310), and the Graduate research

innovation project of Central South University (Nos. 2018zzts118 and

1053320192015).

CR Aggarwal BB, 2003, NAT REV IMMUNOL, V3, P745, DOI 10.1038/nri1184

Bianchini G, 2016, NAT REV CLIN ONCOL, V13, P674, DOI 10.1038/nrclinonc.2016.66

Biskobing DM, 2007, CLIN INTERV AGING, V2, P299

Biswas Debajit K, 2005, Sci STKE, V2005, ppe27, DOI 10.1126/stke.2882005pe27

Chen Y, 2016, ONCOL REP, V36, P137, DOI 10.3892/or.2016.4774

Choi S, 2011, J CELL BIOCHEM, V112, P330, DOI 10.1002/jcb.22932

Christensen LP, 2009, ADV FOOD NUTR RES, V55, P1, DOI 10.1016/S1043-4526(08)00401-4

Compton DR, 2004, J MED CHEM, V47, P5872, DOI 10.1021/jm049631k

Demain AL, 2011, MICROB BIOTECHNOL, V4, P687, DOI 10.1111/j.1751-7915.2010.00221.x

Gierut JJ, 2015, SCI SIGNAL, V8, DOI 10.1126/scisignal.aac7235

Gyorffy B, 2021, COMPUT STRUCT BIOTEC, V19, P4101, DOI 10.1016/j.csbj.2021.07.014

Heldring N, 2007, PHYSIOL REV, V87, P905, DOI 10.1152/physrev.00026.2006

Hsu I, 2014, CARCINOGENESIS, V35, P651, DOI 10.1093/carcin/bgt348

Huang B, 2014, P NATL ACAD SCI USA, V111, P1933, DOI 10.1073/pnas.1323719111

Huang JJ, 2016, ACTA BIOCH BIOPH SIN, V48, P750, DOI 10.1093/abbs/gmw049

Jia M, 2015, BEST PRACT RES CL EN, V29, P557, DOI 10.1016/j.beem.2015.04.008

Josephs SF, 2018, J TRANSL MED, V16, DOI 10.1186/s12967-018-1611-7

Kim JK, 2009, J CELL PHYSIOL, V220, P292, DOI 10.1002/jcp.21791

Kovalchuk SN, 2006, J STEROID BIOCHEM, V101, P226, DOI 10.1016/j.jsbmb.2006.06.022

Kushner PJ, 2000, J STEROID BIOCHEM, V74, P311, DOI 10.1016/S0960-0760(00)00108-4

Lai AC, 2017, NAT REV DRUG DISCOV, V16, P101, DOI 10.1038/nrd.2016.211

Lee Y, 2003, J STEROID BIOCHEM, V84, P463, DOI 10.1016/S0960-0760(03)00067-0

Lee YJ, 2003, ARCH PHARM RES, V26, P58, DOI 10.1007/BF03179933

Li BH, 2011, CANCER LETT, V301, P185, DOI 10.1016/j.canlet.2010.11.015

Li Q, 2017, EUR J PHARMACOL, V815, P173, DOI 10.1016/j.ejphar.2017.09.023

Li SY, 2016, TUMOR BIOL, V37, P15477, DOI 10.1007/s13277-015-3759-6

Liu Y, 2021, MOL MED REP, V24, DOI 10.3892/mmr.2021.12513

Liu ZQ, 2012, CHEM REV, V112, P3329, DOI 10.1021/cr100174k

Liu ZG, 2005, CELL RES, V15, P24, DOI 10.1038/sj.cr.7290259

Locksley RM, 2001, CELL, V104, P487, DOI 10.1016/S0092-8674(01)00237-9

Mann J, 2002, NAT REV CANCER, V2, P143, DOI 10.1038/nrc723

Matthews J, 2006, MOL ENDOCRINOL, V20, P534, DOI 10.1210/me.2005-0140

Mishra AK, 2016, ONCOTARGET, V7, P56876, DOI 10.18632/oncotarget.10871

Osborne CK, 2004, BRIT J CANCER, V90, pS2, DOI 10.1038/sj.bjc.6601629

Park JE, 2021, J GINSENG RES, V45, P754, DOI 10.1016/j.jgr.2021.08.006

Pettersson K, 1997, MOL ENDOCRINOL, V11, P1486, DOI 10.1210/me.11.10.1486

Pike ACW, 1999, EMBO J, V18, P4608, DOI 10.1093/emboj/18.17.4608

Powell E, 2008, P NATL ACAD SCI USA, V105, P19012, DOI 10.1073/pnas.0807274105

Qian Jun, 2016, Asian Pac J Cancer Prev, V17, P1817

Schneider BP, 2008, CLIN CANCER RES, V14, P8010, DOI 10.1158/1078-0432.CCR-08-1208

Shanle EK, 2011, CHEM RES TOXICOL, V24, P6, DOI 10.1021/tx100231n

Shi QQ, 2016, ONCOL REP, V36, P2059, DOI 10.3892/or.2016.5033

Tong-Lin Wu Tony, 2018, Oncotarget, V9, P11109, DOI 10.18632/oncotarget.24326

Wagner EF, 2009, NAT REV CANCER, V9, P537, DOI 10.1038/nrc2694

Wang L, 2008, CELL, V133, P693, DOI 10.1016/j.cell.2008.03.036

Warner M, 2017, TRENDS PHARMACOL SCI, V38, P92, DOI 10.1016/j.tips.2016.10.006

Xia T, 2017, MOL MED REP, V15, P3591, DOI 10.3892/mmr.2017.6459

Yun TK, 2001, LANCET ONCOL, V2, P49, DOI 10.1016/S1470-2045(00)00196-0

Zeng MN, 2018, MOLECULES, V23, DOI 10.3390/molecules23092293

Zheng K, 2016, AUTOPHAGY, V12, P1593, DOI 10.1080/15548627.2016.1192751

Zhuang JJ, 2018, NUTRIENTS, V10, DOI 10.3390/nu10030328

NR 51

TC 1

Z9 1

U1 5

U2 14

PU SCIENCE PRESS

PI BEIJING

PA 16 DONGHUANGCHENGGEN NORTH ST, Building 5, Room 411, BEIJING, 100009,

PEOPLES R CHINA

SN 1672-9145

EI 1745-7270

J9 ACTA BIOCH BIOPH SIN

JI Acta Biochim. Biophys. Sin.

PD MAY

PY 2022

VL 54

IS 5

BP 647

EP 656

DI 10.3724/abbs.2022039

PG 10

WC Biochemistry & Molecular Biology; Biophysics

WE Science Citation Index Expanded (SCI-EXPANDED)

SC Biochemistry & Molecular Biology; Biophysics

GA 3N2SU

UT WOS:000836003000006

PM 35593465

OA Green Published

DA 2023-04-05

ER

PT J

AU Hou, JX

Zhong, LP

Liu, JM

Liu, FL

Xia, CH

AF Hou, Jinxia

Zhong, Lanping

Liu, Jianming

Liu, Fanglan

Xia, Chunhua

TI Interaction of the main active components in Shengmai formula mediated

by organic anion transporter 1 (OAT1)

SO JOURNAL OF ETHNOPHARMACOLOGY

LA English

DT Article

DE Shengmai formula; OAT1; Interactions; Pharmacokinetics

ID TRADITIONAL CHINESE MEDICINE; GINSENOSIDE RB1; COMPATIBILITY; INJECTION;

FAMILY

AB Ethnopharmacological relevance: Shengmai formula (SMF) is a classical traditional Chinese medicine prescription, which is widely used in the treatment of cardiovascular and cerebrovascular diseases. Our previous studies have demonstrated that some components in SMF can interact with each other through breast cancer resistance protein, sodium taurocholate co-transporting polypeptide, organic anion transporting polypeptide 1B1 and 1B3. Organic anion transporter 1 (OAT1) is highly expressed in kidney, mediating the elimination of many endogenous and exogenous substances. However, the interaction between the main active components in SMF and OAT1 is not clear.

Aim of the study: This study aimed to investigate the interactions of the major bioactive components in SMF mediated by OAT1.

Materials and methods: Four main fractions, namely, ginseng total saponins (GTS), ophiopogon total saponins (OTS), ophiopogon total flavonoids (OTF), fructus schisandrae total lignans (STL), and 12 active components, namely, ginsenoside Rg1, Re, Rd and Rb1, ophiopogonin D and D', methylophiopogonanone A and B, schizandrol A and B, schizandrin A and B, were selected to explore the interactions of SMF with OAT1 using cell and rat models.

Results: The above four main fractions in SMF all exhibited inhibitory effects on the uptake of 6-carboxyfluorescein (6-CF), a classic substrate of OAT1. Among the 12 main effective components, only ginsenoside Re, Rd, and methylophiopogonanone A showed inhibition of 6-CF uptake. Additionally, we found that schizandrin B was transported by HEK293-OAT1 cells, and schizandrin B uptake was markedly inhibited by GTS, OTS, OTF, ginsenoside Re, Rd, and methylophiopogonanone A. In rats, ginsenoside Re, Rd, and methylophiopogonanone A jointly increased the AUC((0-t)), AUC((0-infinity)), and C-max of schizandrin B, but they decreased its clearance in plasma and excretion in urine.

Conclusions: Ginsenoside Re, Rd, and methylophiopogonanone A were the potential inhibitors of OAT1, and may interact with some drugs serving as OAT1 substrates clinically. Schizandrin B was a potential OAT1 substrate, and its OAT1-mediated transport was inhibited by ginsenoside Re, Rd, and methylophiopogonanone A. OAT1-mediated interactions of the main active components in SMF can be regarded as one of the important compatibility mechanisms of traditional Chinese medicine preparations.

C1 [Hou, Jinxia; Zhong, Lanping; Liu, Jianming; Liu, Fanglan; Xia, Chunhua] Nanchang Univ, Clin Pharmacol Inst, Xuefu Rd 1299, Nanchang 330031, Peoples R China.

[Hou, Jinxia] Jiangxi Prov Peoples Hosp, Pharm Dept, Nanchang 330006, Peoples R China.

C3 Nanchang University

RP Xia, CH (通讯作者)，Nanchang Univ, Clin Pharmacol Inst, Xuefu Rd 1299, Nanchang 330031, Peoples R China.

EM xch720917@ncu.edu.cn

FU National Natural Science Foundation of China [81760672, 82160708];

Natural Science Foundation of Jiangxi Province [20202ACB206013]; Key

Research and Development Program of Jiangxi Province [20212BBG73035]

FX Acknowledgments This research was supported by National Natural Science

Foundation of China (No. 81760672 and 82160708) , Natural Science

Foundation of Jiangxi Province (No. 20202ACB206013) and Key Research and

Development Program of Jiangxi Province (No. 20212BBG73035) .

CR Ahn SY, 2008, CURR OPIN NEPHROL HY, V17, P499, DOI 10.1097/MNH.0b013e32830b5d5d

Burckhardt BC, 2003, REV PHYSIOL BIOCH P, V146, P95, DOI 10.1007/s10254-002-0003-8

Chen L, 2019, XENOBIOTICA, V49, P1221, DOI 10.1080/00498254.2018.1493757

Huang XY, 2019, COMPLEMENT THER MED, V43, P140, DOI 10.1016/j.ctim.2019.01.020

Huo XK, 2020, PHYTOTHER RES, V34, P2998, DOI 10.1002/ptr.6727

Jiang Y, 2020, BIOSCIENCE REP, V40, DOI 10.1042/BSR20200286

Konig J, 2013, PHARMACOL REV, V65, P944, DOI 10.1124/pr.113.007518

Lao YZ, 2014, J ETHNOPHARMACOL, V155, P1, DOI 10.1016/j.jep.2014.05.022

Li CY, 2020, TOXICOL SCI, V175, P279, DOI 10.1093/toxsci/kfaa033

Liu XP, 2018, FRONT PHARMACOL, V9, DOI 10.3389/fphar.2018.00957

Lu H, 2017, PEERJ, V5, DOI 10.7717/peerj.3333

Luan X, 2020, J ETHNOPHARMACOL, V254, DOI 10.1016/j.jep.2020.112687

Newman DJ, 2020, ADV PHARMACOL, V87, P113, DOI 10.1016/bs.apha.2019.07.001

Nigam AK, 2020, J BIOL CHEM, V295, P1829, DOI 10.1074/jbc.RA119.010729

Nigam SK, 2018, ANNU REV PHARMACOL, V58, P663, DOI 10.1146/annurev-pharmtox-010617-052713

Nigam SK, 2015, PHYSIOL REV, V95, P83, DOI 10.1152/physrev.00025.2013

Taniguchi T, 2019, J PHARMACOL EXP THER, V371, P162, DOI 10.1124/jpet.119.259341

Tian J, 2018, CELL PHYSIOL BIOCHEM, V50, P1726, DOI 10.1159/000494791

Wang SP, 2012, J ETHNOPHARMACOL, V143, P412, DOI 10.1016/j.jep.2012.07.033

Wu LN, 2021, BIOMED PHARMACOTHER, V133, DOI 10.1016/j.biopha.2020.110939

Wu X, 2016, J CHROMATOGR B, V1026, P236, DOI 10.1016/j.jchromb.2015.11.024

Xiong XJ, 2020, PHARMACOL RES, V160, DOI 10.1016/j.phrs.2020.105056

Yang P, 2018, ACTA BIOCH BIOPH SIN, V50, P144, DOI 10.1093/abbs/gmx136

You Y, 2020, CHIN MED-UK, V15, DOI 10.1186/s13020-020-00394-y

Zhan T, 2019, PHYTOMEDICINE, V59, DOI 10.1016/j.phymed.2019.152916

Zhang C.Q., 2020, J PRAC TRADIT CHIN M, V36, P409

Zhang JH, 2015, ACTA PHARMACOL SIN, V36, P654, DOI 10.1038/aps.2015.8

Zhang X, 2018, J CELL MOL MED, V22, P409, DOI 10.1111/jcmm.13329

Zhang YQ, 2020, J ETHNOPHARMACOL, V250, DOI 10.1016/j.jep.2019.112528

Zhou MM, 2017, J ETHNOPHARMACOL, V206, P363, DOI 10.1016/j.jep.2017.06.007

Zhou Q, 2014, COCHRANE DB SYST REV, DOI 10.1002/14651858.CD005052.pub5

NR 31

TC 0

Z9 0

U1 5

U2 6

PU ELSEVIER IRELAND LTD

PI CLARE

PA ELSEVIER HOUSE, BROOKVALE PLAZA, EAST PARK SHANNON, CO, CLARE, 00000,

IRELAND

SN 0378-8741

EI 1872-7573

J9 J ETHNOPHARMACOL

JI J. Ethnopharmacol.

PD OCT 5

PY 2022

VL 296

AR 115515

DI 10.1016/j.jep.2022.115515

EA JUL 2022

PG 8

WC Plant Sciences; Chemistry, Medicinal; Integrative & Complementary

Medicine; Pharmacology & Pharmacy

WE Science Citation Index Expanded (SCI-EXPANDED)

SC Plant Sciences; Pharmacology & Pharmacy; Integrative & Complementary

Medicine

GA 3A4NC

UT WOS:000827237600005

PM 35777609

DA 2023-04-05

ER

PT J

AU Shan, KZ

Deng, YJ

Du, ZQ

Yue, PY

Yang, SF

AF Shan, Kuizhong

Deng, Yujiang

Du, Zhiquan

Yue, Peiyu

Yang, Sufang

TI Examination of Combined Treatment of Ginsenoside Rg3 and 5-Fluorouracil

in Lung Adenocarcinoma Cells

SO COMPUTATIONAL AND MATHEMATICAL METHODS IN MEDICINE

LA English

DT Article

ID NF-KAPPA-B; CANCER CELLS; DOWN-REGULATION; CISPLATIN; ANGIOGENESIS;

CHEMOTHERAPY; EXPRESSION; VEGF; 5-FU

AB Chemotherapy is a commonly used strategy for advanced lung cancer patients. However, its clinical application is restrained due to its toxicity and drug resistance. Ginsenoside Rg3 (Rg3) has a strong anticancer influence on colon cancer, breast cancer, lung cancer, and other malignant tumors. However, it is still unclear whether Rg3 can cooperate with 5-FU to inhibit the tumor growth and angiogenesis of lung adenocarcinoma (LUAD). This study examined the combined treatment of Rg3 and 5-FU in LUAD. It was revealed that the combined treatment could notably enhance the suppression on proliferative, invasive, and migratory abilities and angiogenesis in LUAD cells A549 and SPC-A-1. On the other hand, we also discovered that Rg3 or 5-FU could suppress the activity of the NF-kappa B signaling pathway and downregulate VEGFA expression in LUAD cells. Collectively, this study suggested that Rg3 combined chemotherapy may perform a more powerful drug efficiency in LUAD cells.

C1 [Shan, Kuizhong; Deng, Yujiang; Du, Zhiquan; Yue, Peiyu; Yang, Sufang] Second Peoples Hosp Kunshan, Dept Pulm Nodule Ctr, Suzhou 215300, Peoples R China.

RP Shan, KZ (通讯作者)，Second Peoples Hosp Kunshan, Dept Pulm Nodule Ctr, Suzhou 215300, Peoples R China.

EM shankz11@163.com; toughrafbl@outlook.com; deategvnh@outlook.com;

theleelpf@outlook.com; kuslithdajqh@outlook.com

FU Kunshan Science and Technology Bureau Project [ks1905]

FX This study was supported by the funds from the project of Kunshan

Science and Technology Bureau Project (fund No.: ks1905).

CR Abeyama K, 2000, J CLIN INVEST, V105, P1751, DOI 10.1172/JCI9745

Cancer Genome Atlas Research Network, 2014, Nature, V511, P543, DOI [10.1038/s41586-018-0228-6, 10.1038/nature13385]

Cao Y, 2017, PLOS ONE, V12, DOI 10.1371/journal.pone.0186520

Carmeliet P, 2005, ONCOLOGY-BASEL, V69, P4, DOI 10.1159/000088478

Chung HW, 2017, CANCER SCI, V108, P1594, DOI 10.1111/cas.13288

Collisson EA, 2014, NATURE, V511, P543, DOI 10.1038/nature13385

Gao Y, 2017, J PINEAL RES, V62, DOI 10.1111/jpi.12380

Garg A, 2002, LEUKEMIA, V16, P1053, DOI 10.1038/sj.leu.2402482

Greten FR, 2004, CELL, V118, P285, DOI 10.1016/j.cell.2004.07.013

Hong SZ, 2020, ONCOL REP, V44, P1333, DOI 10.3892/or.2020.7728

Hu S, 2019, ANAL CELL PATHOL, V2019, DOI 10.1155/2019/3815786

Kiriakidis S, 2003, J CELL SCI, V116, P665, DOI 10.1242/jcs.00286

Li S, 2019, ANGIOGENESIS, V22, P15, DOI 10.1007/s10456-018-9645-2

Lim TG, 2015, ARCH DERMATOL RES, V307, P397, DOI 10.1007/s00403-015-1569-8

Meng LB, 2019, INT J ONCOL, V54, P2069, DOI 10.3892/ijo.2019.4787

Nakhjavani M, 2020, MOLECULES, V25, DOI 10.3390/molecules25214905

Noro R, 2010, CANCER SCI, V101, P1424, DOI 10.1111/j.1349-7006.2010.01559.x

Qi FH, 2015, BIOSCI TRENDS, V9, P16, DOI 10.5582/bst.2015.01019

RICHARDS F, 1991, CANCER, V67, P2974

Sethy C, 2021, BIOMED PHARMACOTHER, V137, DOI 10.1016/j.biopha.2021.111285

Smith I, 2014, NUTR REV, V72, P319, DOI 10.1111/nure.12099

Sun CX, 2016, J EXP CLIN CANC RES, V35, DOI 10.1186/s13046-015-0274-y

Sun X, 2022, J GINSENG RES, V46, DOI 10.1016/j.jgr.2021.06.009

Tabruyn SP, 2008, ANGIOGENESIS, V11, P101, DOI 10.1007/s10456-008-9094-4

TAPAZOGLOU E, 1988, INVEST NEW DRUG, V6, P259

Vodenkova S, 2020, PHARMACOL THERAPEUT, V206, DOI 10.1016/j.pharmthera.2019.107447

Wang JH, 2014, TUMOR BIOL, V35, P11985, DOI 10.1007/s13277-014-2497-5

Wang JJ, 2018, CANCER LETT, V415, P73, DOI 10.1016/j.canlet.2017.11.037

Wigmore PM, 2010, ADV EXP MED BIOL, V678, P157

Wu RH, 2014, J FOOD SCI, V79, pH1430, DOI 10.1111/1750-3841.12518

Yang LQ, 2012, BIOPHARM DRUG DISPOS, V33, P425, DOI 10.1002/bdd.1806

Yang XL, 2017, BIOMED PHARMACOTHER, V96, P1240, DOI 10.1016/j.biopha.2017.11.092

Zaric B, 2013, J THORAC DIS, V5, pS371, DOI 10.3978/j.issn.2072-1439.2013.05.16

Zhao JG, 2014, TUMOR BIOL, V35, P12305, DOI 10.1007/s13277-014-2543-3

Zheng Q, 2021, MOL MED REP, V24, DOI 10.3892/mmr.2021.12222

NR 36

TC 2

Z9 2

U1 0

U2 0

PU HINDAWI LTD

PI LONDON

PA ADAM HOUSE, 3RD FLR, 1 FITZROY SQ, LONDON, W1T 5HF, ENGLAND

SN 1748-670X

EI 1748-6718

J9 COMPUT MATH METHOD M

JI Comput. Math. Method Med.

PD JUN 28

PY 2022

VL 2022

AR 2813142

DI 10.1155/2022/2813142

PG 8

WC Mathematical & Computational Biology

WE Science Citation Index Expanded (SCI-EXPANDED)

SC Mathematical & Computational Biology

GA 3B1KQ

UT WOS:000827707600003

PM 35799655

OA gold, Green Published

DA 2023-04-05

ER

PT J

AU Zhang, B

Fu, RZ

Duan, ZG

Shen, SH

Zhu, CH

Fan, DD

AF Zhang, Bo

Fu, Rongzhan

Duan, Zhiguang

Shen, Shihong

Zhu, Chenhui

Fan, Daidi

TI Ginsenoside CK induces apoptosis in triple-negative breast cancer cells

by targeting glutamine metabolism

SO BIOCHEMICAL PHARMACOLOGY

LA English

DT Article

DE Ginsenoside CK; Glutamine metabolism; GLS1; Apoptosis; Triple-negative

breast cancer

ID COMPOUND K; GROWTH; CHEMOTHERAPY; ABSORPTION; EXCRETION; CARCINOMA;

SAPONINS; PATHWAY; BIOLOGY; DEATH

AB Breast cancer (BC) has replaced lung cancer as the most common cancer worldwide. Ginsenoside CK (CK) can effectively inhibit triple-negative breast cancer (TNBC), the occurrence and development of which are associated with glutamine addiction. However, the connection between CK and glutamine metabolism in TNBC proliferation and the mechanism of cell death induction remains unclear. Here, we found that high glutamine-addicted TNBC cells were particularly sensitive to CK treatment. CK exerted antitumour activity against TNBC by suppressing glutamine consumption and glutamate production via downregulation of glutaminase 1 (GLS1) expression. CK treatment further decreased cellular ATP production, reduced the utilisation of amino acids associated with glutamine metabolism, and induced glutathione (GSH) depletion and reactive oxygen species (ROS) accumulation, consequently triggering apoptosis in TNBC. Furthermore, CK decreased GLS1 expression in SUM159 xenograft mouse mammary tumours and significantly inhibited tumour growth with few side effects. Together, our data provide a powerful theoretical basis for the application of CK as a glutamine metabolic inhibitor in TNBC treatment.

C1 [Zhu, Chenhui; Fan, Daidi] Northwest Univ, Shaanxi Key Lab Degradable Biomed Mat, Sch Chem Engn, 229 North Taibai Rd, Xian 710069, Shaanxi, Peoples R China.

Northwest Univ, Shaanxi R&D Ctr Biomat & Fermentat Engn, Sch Chem Engn, 229 North Taibai Rd, Xian 710069, Shaanxi, Peoples R China.

Northwest Univ, Biotech & Biomed Res Inst, 229 North Taibai Rd, Xian 710069, Shaanxi, Peoples R China.

C3 Northwest University Xi'an; Northwest University Xi'an; Northwest

University Xi'an

RP Zhu, CH; Fan, DD (通讯作者)，Northwest Univ, Shaanxi Key Lab Degradable Biomed Mat, Sch Chem Engn, 229 North Taibai Rd, Xian 710069, Shaanxi, Peoples R China.

EM zch2005@nwu.edu.cn; fandaidi@nwu.edu.cn

FU National Key R&D Pro-gram of China [2021YFC2103900, 2021YFC2101500];

National Natural Science Foundation of China [22178287, 22108224];

Nat-ural Science Foundation of Shaanxi Province, China [2020JQ-570];

Xi?an Science and Technology Project [20191422315KYPT014JC016]

FX Acknowledgments This study was financially supported by the National Key

R&D Pro-gram of China (2021YFC2103900, 2021YFC2101500) , the National

Natural Science Foundation of China (22178287, 22108224) , the Nat-ural

Science Foundation of Shaanxi Province, China (No. 2020JQ-570) , the

Xi?an Science and Technology Project (20191422315KYPT014JC016) .

CR Anderson FH, 2000, HEPATOL RES, V18, P63, DOI 10.1016/S1386-6346(99)00085-6

Bansal A, 2018, J CELL BIOL, V217, P2291, DOI 10.1083/jcb.201804161

CATANE R, 1979, CANCER TREAT REP, V63, P1033

Chae S, 2009, J AGR FOOD CHEM, V57, P5777, DOI 10.1021/jf900331g

Chen K, 2020, ONCOL REP, V43, P886, DOI 10.3892/or.2020.7460

Choi BH, 2019, CANCERS, V11, DOI 10.3390/cancers11050675

Coley HM, 2009, EJC SUPPL, V7, P3, DOI 10.1016/S1359-6349(09)70003-5

Counihan JL, 2018, CHEM REV, V118, P6893, DOI 10.1021/acs.chemrev.7b00775

DeBerardinis RJ, 2010, ONCOGENE, V29, P313, DOI 10.1038/onc.2009.358

DeBerardinis RJ, 2008, CURR OPIN GENET DEV, V18, P54, DOI 10.1016/j.gde.2008.02.003

DeBerardinis RJ, 2008, CELL METAB, V7, P11, DOI 10.1016/j.cmet.2007.10.002

DeBerardinis RJ, 2007, P NATL ACAD SCI USA, V104, P19345, DOI 10.1073/pnas.0709747104

DeMichele A, 2016, J CLIN ONCOL, V34, DOI 10.1200/JCO.2016.34.15_suppl.1011

DEMPLE B, 1994, ANNU REV BIOCHEM, V63, P915, DOI 10.1146/annurev.biochem.63.1.915

Deng XQ, 2020, BIOCHEM PHARMACOL, V178, DOI 10.1016/j.bcp.2020.114038

Dent R, 2007, CLIN CANCER RES, V13, P4429, DOI 10.1158/1078-0432.CCR-06-3045

Glunde K, 2004, CANCER RES, V64, P4270, DOI 10.1158/0008-5472.CAN-03-3829

Gross MI, 2014, MOL CANCER THER, V13, P890, DOI 10.1158/1535-7163.MCT-13-0870

Gwangwa MV, 2019, BIOL RES, V52, DOI 10.1186/s40659-019-0224-9

Hanahan D, 2011, CELL, V144, P646, DOI 10.1016/j.cell.2011.02.013

Hong YN, 2019, J FUNCT FOODS, V57, P255, DOI 10.1016/j.jff.2019.04.019

Hsu PP, 2008, CELL, V134, P703, DOI 10.1016/j.cell.2008.08.021

Jacobs MA, 2004, J MAGN RESON IMAGING, V19, P68, DOI 10.1002/jmri.10427

KARIKURA M, 1991, CHEM PHARM BULL, V39, P2357

KARIKURA M, 1991, CHEM PHARM BULL, V39, P400

Khan A, 2021, J AGR FOOD CHEM, V69, P6897, DOI 10.1021/acs.jafc.1c01173

Kim AD, 2010, ENVIRON TOXICOL PHAR, V30, P134, DOI 10.1016/j.etap.2010.04.008

Korangath P, 2015, CLIN CANCER RES, V21, P3263, DOI 10.1158/1078-0432.CCR-14-1200

Kung HN, 2011, PLOS GENET, V7, DOI 10.1371/journal.pgen.1002229

Lee SJ, 2018, J FUNCT FOODS, V46, P159, DOI 10.1016/j.jff.2018.04.050

Li BH, 2019, EBIOMEDICINE, V39, P239, DOI 10.1016/j.ebiom.2018.11.063

Liu JL, 2021, J FUNCT FOODS, V76, DOI 10.1016/j.jff.2020.104325

Mates JM, 2018, BBA-REV CANCER, V1870, P158, DOI 10.1016/j.bbcan.2018.07.007

MATSUNO T, 1987, INT J BIOCHEM, V19, P303, DOI 10.1016/0020-711X(87)90002-4

Ming YL, 2011, PLANTA MED, V77, P428, DOI 10.1055/s-0030-1250454

Nasimian A, 2020, BIOCHEM PHARMACOL, V177, DOI 10.1016/j.bcp.2020.113999

Oh JM, 2019, INT J MOL SCI, V20, DOI 10.3390/ijms20174279

Shiman R, 1998, J BIOL CHEM, V273, P34760, DOI 10.1074/jbc.273.52.34760

Tao JJ, 2015, BREAST, V24, pS149, DOI 10.1016/j.breast.2015.07.035

van Geldermalsen M, 2016, ONCOGENE, V35, P3201, DOI 10.1038/onc.2015.381

World Health Organization, 2020, LAT GLOB CANC DAT CA

Wu GY, 2004, J NUTR, V134, P489, DOI 10.1093/jn/134.10.2783S

Wu Q, 2018, BIOCHEM PHARMACOL, V148, P64, DOI 10.1016/j.bcp.2017.12.004

Yang WH, 2021, TRENDS CANCER, V7, P790, DOI 10.1016/j.trecan.2021.04.003

Yang XD, 2015, FITOTERAPIA, V100, P208, DOI 10.1016/j.fitote.2014.11.019

Zhang X, 2018, MOLECULES, V23, DOI 10.3390/molecules23061482

Zhao Y, 2010, CANCER LETT, V288, P42, DOI 10.1016/j.canlet.2009.06.021

Zou ZZ, 2017, APOPTOSIS, V22, P1321, DOI 10.1007/s10495-017-1424-9

NR 48

TC 3

Z9 3

U1 15

U2 26

PU PERGAMON-ELSEVIER SCIENCE LTD

PI OXFORD

PA THE BOULEVARD, LANGFORD LANE, KIDLINGTON, OXFORD OX5 1GB, ENGLAND

SN 0006-2952

EI 1873-2968

J9 BIOCHEM PHARMACOL

JI Biochem. Pharmacol.

PD AUG

PY 2022

VL 202

AR 115101

DI 10.1016/j.bcp.2022.115101

EA JUN 2022

PG 19

WC Pharmacology & Pharmacy

WE Science Citation Index Expanded (SCI-EXPANDED)

SC Pharmacology & Pharmacy

GA 2M9NK

UT WOS:000818018100002

PM 35618001

DA 2023-04-05

ER

PT J

AU Xue, XD

Liu, YN

Qu, LL

Fan, CY

Ma, XX

Ouyang, PK

Fan, DD

AF Xue, Xiaodan

Liu, Yannan

Qu, Linlin

Fan, Cuiying

Ma, Xiaoxuan

Ouyang, Pingkai

Fan, Daidi

TI Ginsenoside Rh3 Inhibits Lung Cancer Metastasis by Targeting

Extracellular Signal-Regulated Kinase: A Network Pharmacology Study

SO PHARMACEUTICALS

LA English

DT Article

DE ginsenoside Rh3; network pharmacology; human lung cancer; metastasis;

ERK

ID KOREAN RED GINSENG; BREAST-CANCER; PANAX-GINSENG; CELLS; ACTIVATION;

HYPOXIA; ANGIOGENESIS; PROGRESSION; MECHANISMS; EXPRESSION

AB Lung cancer has a high mortality rate and is very common. One of the main reasons for the poor prognosis of patients with lung cancer is the high incidence of metastasis. Ginsenoside Rh3, a rare ginsenoside extracted from Panax notoginseng, exhibits excellent anti-inflammatory and anti-tumor effects. Nonetheless, the inhibitory potential of Rh3 against lung cancer remains unknown. The target genes of Rh3 were screened by the PharmMapper database; the proliferation of lung cancer cells was detected by MTT assay; the migration and invasion of cells were detected by the Transwell method; and the expression of extracellular signal-regulated kinase (ERK) and EMT-related proteins in vivo and in vitro were detected by Western blotting. In addition, we established a lung metastasis model in nude mice using A549 cells to assess the effect of Rh3 on NSCLC tumor metastasis in vivo. Our findings suggest that Rh3 significantly inhibited lung cancer metastasis both in vivo and in vitro. It was determined by flow cytometry analysis that Rh3 notably inhibited cell proliferation by blocking the G1 phase. In addition, Rh3 inhibited metastasis in lung cancer cells and regulated the expression of metastasis-related proteins under hypoxia. Mechanistic studies suggested that Rh3 targeted ERK to inhibit lung cancer metastasis. The ERK inhibitor U0126 or siRNA-mediated knockdown of ERK had an enhanced effect on Rh3's ability to inhibit lung cancer metastasis. The studies revealed that the inhibitory effect of Rh3 on the metastatic ability of lung cancer cells may be supported by ERK-related signaling pathways.

C1 [Xue, Xiaodan; Liu, Yannan; Qu, Linlin; Ma, Xiaoxuan; Fan, Daidi] Northwest Univ, Sch Chem Engn, Shaanxi Key Lab Degradable Biomed Mat, Taibai North Rd 229, Xian 710069, Peoples R China.

[Xue, Xiaodan; Liu, Yannan; Qu, Linlin; Ma, Xiaoxuan; Fan, Daidi] Northwest Univ, Shaanxi R&D Ctr Biomat & Fermentat Engn, Sch Chem Engn, Taibai North Rd 229, Xian 710069, Peoples R China.

[Xue, Xiaodan; Liu, Yannan; Qu, Linlin; Ma, Xiaoxuan; Fan, Daidi] Northwest Univ, Biotech & Biomed Res Inst, Taibai North Rd 229, Xian 710069, Peoples R China.

[Fan, Cuiying] Xian Giant Biotechnol Co Ltd, Xian 710076, Peoples R China.

[Ouyang, Pingkai] Nanjing Tech Univ, Coll Biotechnol & Pharmaceut Engn, Nanjing 211816, Peoples R China.

C3 Northwest University Xi'an; Northwest University Xi'an; Northwest

University Xi'an; Nanjing Tech University

RP Fan, DD (通讯作者)，Northwest Univ, Sch Chem Engn, Shaanxi Key Lab Degradable Biomed Mat, Taibai North Rd 229, Xian 710069, Peoples R China.; Fan, DD (通讯作者)，Northwest Univ, Shaanxi R&D Ctr Biomat & Fermentat Engn, Sch Chem Engn, Taibai North Rd 229, Xian 710069, Peoples R China.; Fan, DD (通讯作者)，Northwest Univ, Biotech & Biomed Res Inst, Taibai North Rd 229, Xian 710069, Peoples R China.

EM xuexiaodan@stumail.nwu.edu.cn; liuyannan@nwu.edu.cn;

linlinqu1994@163.com; wangyoucao526@163.com; xiaoxuanma@nwu.edu.cn;

ouyangpk@njtech.edu.cn; fandaidi@nwu.edu.cn

FU National Key R&D Program of China [2021YFC2101500]; National Natural

Science Foundation of China [22078264, 22108224]; Scientific Research

Projects of Shaanxi Provincial Department of Education [21JK0940]; Xian

Association for Science and Technology Youth Talent Support Plan

[095920211301]; Natural Science Foundation of Shaanxi Province

[2020JQ-570]

FX This research was funded by the National Key R&D Program of China (grant

number 2021YFC2101500); the National Natural Science Foundation of China

(grant number 22078264 and 22108224); the Scientific Research Projects

of Shaanxi Provincial Department of Education (grant number 21JK0940);

the Xian Association for Science and Technology Youth Talent Support

Plan (grant number 095920211301); and the Natural Science Foundation of

Shaanxi Province (grant number 2020JQ-570).

CR Ahn GO, 2014, P NATL ACAD SCI USA, V111, P2698, DOI 10.1073/pnas.1320243111

Ando N, 2012, ANN SURG ONCOL, V19, P68, DOI 10.1245/s10434-011-2049-9

Chang L, 2014, CANCER METAST REV, V33, P469, DOI 10.1007/s10555-014-9493-5

Chung I, 2015, J GINSENG RES, V39, P322, DOI 10.1016/j.jgr.2015.03.001

Coon JT, 2002, DRUG SAFETY, V25, P323, DOI 10.2165/00002018-200225050-00003

de Araujo RSA, 2022, PHARMACEUTICALS-BASE, V15, DOI 10.3390/ph15010104

Feng WW, 2021, SCI REP-UK, V11, DOI 10.1038/s41598-021-02167-5

Fu HC, 2018, CLIN CANCER RES, V24, P3069, DOI 10.1158/1078-0432.CCR-17-2687

Fukuoka S, 2002, BBA-MOL BASIS DIS, V1588, P106, DOI 10.1016/S0925-4439(02)00153-9

Gonzalez-Moreno O, 2010, EXP CELL RES, V316, P554, DOI 10.1016/j.yexcr.2009.11.020

Hagen T, 2003, SCIENCE, V302, P1975, DOI 10.1126/science.1088805

Huang LH, 2015, ACTA PHARM SIN B, V5, P390, DOI 10.1016/j.apsb.2015.07.001

Inamura K, 2017, FRONT ONCOL, V7, DOI 10.3389/fonc.2017.00193

Kang KS, 2013, J GINSENG RES, V37, P379, DOI 10.5142/jgr.2013.37.379

Lee HL, 2017, J GINSENG RES, V41, P227, DOI 10.1016/j.jgr.2017.01.011

Li BH, 2011, CANCER LETT, V301, P185, DOI 10.1016/j.canlet.2010.11.015

Liu XK, 2018, EVID-BASED COMPL ALT, V2018, DOI 10.1155/2018/7802639

Luo MN, 2016, CANCER LETT, V373, P1, DOI 10.1016/j.canlet.2016.01.010

Meng YB, 2017, ONCOL RES, V25, P1207, DOI 10.3727/096504017X14886679715637

Mylonis I, 2006, J BIOL CHEM, V281, P33095, DOI 10.1074/jbc.M605058200

NIGG EA, 1995, BIOESSAYS, V17, P471, DOI 10.1002/bies.950170603

Panossian AG, 2021, MED RES REV, V41, P630, DOI 10.1002/med.21743

Pastushenko I, 2019, TRENDS CELL BIOL, V29, P212, DOI 10.1016/j.tcb.2018.12.001

Quail DF, 2013, NAT MED, V19, P1423, DOI 10.1038/nm.3394

Ralph SJ, 2015, PHARMACEUTICALS-BASE, V8, P62, DOI 10.3390/ph8010062

Rengarajan T, 2014, ASIAN PAC J CANCER P, V15, P1757, DOI 10.7314/APJCP.2014.15.4.1757

Samal SK, 2015, SCI REP-UK, V5, DOI 10.1038/srep09982

Savoia P, 2019, INT J MOL SCI, V20, DOI 10.3390/ijms20061483

Semenza GL, 2003, NAT REV CANCER, V3, P721, DOI 10.1038/nrc1187

Shikov AN, 2021, J ETHNOPHARMACOL, V268, DOI 10.1016/j.jep.2020.113685

Shin YW, 2006, ARCH PHARM RES, V29, P685, DOI 10.1007/BF02968253

Singh A, 2017, INT REV NEUROBIOL, V135, P197, DOI 10.1016/bs.irn.2017.02.010

Sipos F, 2012, WORLD J GASTROENTERO, V18, P601, DOI 10.3748/wjg.v18.i7.601

Sulzmaier FJ, 2013, CANCER RES, V73, P6099, DOI 10.1158/0008-5472.CAN-13-1087

Tang C, 2016, ACTA ONCOL, V55, P1022, DOI 10.3109/0284186X.2016.1154602

Tang CZ, 2018, FREE RADICAL BIO MED, V117, P238, DOI 10.1016/j.freeradbiomed.2018.02.001

Tsuji T, 2009, CANCER RES, V69, P7135, DOI 10.1158/0008-5472.CAN-09-1618

Umezu T, 2014, BLOOD, V124, P3748, DOI 10.1182/blood-2014-05-576116

Wang T, 2018, BIOMED ENVIRON SCI, V31, P855, DOI 10.3967/bes2018.114

Xie SJ, 2021, CELL DEATH DISCOV, V7, DOI 10.1038/s41420-021-00497-x

Yang GM, 2022, J GINSENG RES, V46, P426, DOI 10.1016/j.jgr.2021.07.005

Yang MH, 2008, CELL CYCLE, V7, P2090, DOI 10.4161/cc.7.14.6324

Yang MH, 2008, NAT CELL BIOL, V10, P295, DOI 10.1038/ncb1691

Yang XB, 2021, ONCOL REP, V45, P459, DOI 10.3892/or.2020.7881

Yang YCSH, 2021, J BIOMED SCI, V28, DOI 10.1186/s12929-021-00719-5

Yayeh T, 2012, J GINSENG RES, V36, P263, DOI 10.5142/jgr.2012.36.3.263

Yi YS, 2019, J GINSENG RES, V43, P172, DOI 10.1016/j.jgr.2017.11.005

Ying Y, 2019, J AGR FOOD CHEM, V67, P8348, DOI 10.1021/acs.jafc.9b02954

Zheng YC, 2021, CELL DEATH DISCOV, V7, DOI 10.1038/s41420-021-00764-x

Zhu L, 2021, INT J BIOL SCI, V17, P926, DOI 10.7150/ijbs.57445

Zhu NQ, 2019, MED SCI MONITOR, V25, P6051, DOI 10.12659/MSM.915821

NR 51

TC 0

Z9 0

U1 9

U2 12

PU MDPI

PI BASEL

PA ST ALBAN-ANLAGE 66, CH-4052 BASEL, SWITZERLAND

EI 1424-8247

J9 PHARMACEUTICALS-BASE

JI Pharmaceuticals

PD JUN

PY 2022

VL 15

IS 6

AR 758

DI 10.3390/ph15060758

PG 20

WC Chemistry, Medicinal; Pharmacology & Pharmacy

WE Science Citation Index Expanded (SCI-EXPANDED)

SC Pharmacology & Pharmacy

GA 2N2TT

UT WOS:000818238800001

PM 35745677

OA Green Published, gold

DA 2023-04-05

ER

PT J

AU Chen, C

Xia, JX

Ren, HW

Wang, AN

Zhu, Y

Zhang, R

Gan, ZC

Wang, JX

AF Chen, Chen

Xia, Jiaxuan

Ren, Hongwei

Wang, Anni

Zhu, Ying

Zhang, Ru

Gan, Zicheng

Wang, Jianxin

TI Effect of the structure of ginsenosides on the in vivo fate of their

liposomes

SO ASIAN JOURNAL OF PHARMACEUTICAL SCIENCES

LA English

DT Article

DE Ginsenosides; Liposomes; Structure activity relationship; Rg3 liposomes;

Long circulation; Tumor targeting; Glut 1

ID GLUT-1 EXPRESSION; BREAST-CANCER; MECHANISM; CHOLESTEROL; ABSORPTION;

STABILITY; GINSENG

AB To utilize the multiple functions and give full play of ginsenosides, a variety of ginsenosides with different structures were prepared into liposomes and evaluated for their effect on the stability, pharmacokinetics and tumor targeting capability of liposomes. The results showed that the position and number of glycosyl groups of ginsenosides have significant effect on the in vitro and in vivo properties of their liposomes. The pharmacokinetics of ginsenosides liposomes indicated that the C-3 sugar group of ginsenosides is beneficial to their liposomes for longer circulation in vivo. The C-3 and C-6 glycosyls can enhance the uptake of their liposomes by 4T1 cells, and the glycosyls at C-3 position can enhance the tumor active targeting ability significantly, based on the specific binding capacity to Glut 1 expressed on the surface of 4T1 cells. According to the results in the study, ginsenoside Rg3 and ginsenoside Rh2 are potential for exploiting novel liposomes because of their cholesterol substitution, long blood circulation and tumor targeting capabilities. The results provide a theoretical basis for further development of ginsenoside based liposome delivery systems. (C) 2022 Shenyang Pharmaceutical University. Published by Elsevier B.V.

C1 [Chen, Chen; Xia, Jiaxuan; Ren, Hongwei; Wang, Anni; Zhu, Ying; Zhang, Ru; Gan, Zicheng; Wang, Jianxin] Fudan Univ, Sch Pharm, Dept Pharmaceut, Shanghai 201203, Peoples R China.

[Chen, Chen; Xia, Jiaxuan; Ren, Hongwei; Wang, Anni; Zhu, Ying; Zhang, Ru; Gan, Zicheng; Wang, Jianxin] Minist Educ, Key Lab Smart Drug Delivery, Shanghai 201203, Peoples R China.

[Zhu, Ying] Guangzhou Univ Chinese Med, Inst Trop Med, Guangzhou 510006, Peoples R China.

[Wang, Jianxin] Fudan Univ, Inst Integrated Chinese & Western Med, Shanghai 200040, Peoples R China.

C3 Fudan University; Guangzhou University of Chinese Medicine; Fudan

University

RP Wang, JX (通讯作者)，Fudan Univ, Sch Pharm, Dept Pharmaceut, Shanghai 201203, Peoples R China.; Wang, JX (通讯作者)，Minist Educ, Key Lab Smart Drug Delivery, Shanghai 201203, Peoples R China.; Wang, JX (通讯作者)，Fudan Univ, Inst Integrated Chinese & Western Med, Shanghai 200040, Peoples R China.

EM jxwang@fudan.edu.cn

FU National Natural Science Foundation of China [82074277, 81773911];

Development Project of Shanghai Peak Disciplines-Integrated Medicine

[20180101]

FX This work was supported by the National Natural Science Foundation of

China (No. 82074277 and 81773911) , and the Development Project of

Shanghai Peak Disciplines-Integrated Medicine (No. 20180101) .

CR Avril N, 2004, J NUCL MED, V45, P930

Briuglia ML, 2015, DRUG DELIV TRANSL RE, V5, P231, DOI 10.1007/s13346-015-0220-8

BROWN RS, 1993, CANCER, V72, P2979, DOI 10.1002/1097-0142(19931115)72:10<2979::AID-CNCR2820721020>3.0.CO;2-X

Cantuaria G, 2001, CANCER-AM CANCER SOC, V92, P1144, DOI 10.1002/1097-0142(20010901)92:5<1144::AID-CNCR1432>3.0.CO;2-T

Dong H, 2011, MOLECULES, V16, P10619, DOI 10.3390/molecules161210619

Duan ZG, 2018, BIOCHEM BIOPH RES CO, V499, P482, DOI 10.1016/j.bbrc.2018.03.174

Guo XW, 2018, CHIN J INTEGR MED, V24, P227, DOI 10.1007/s11655-017-2951-5

Hong C, 2020, NANO-MICRO LETT, V12, DOI 10.1007/s40820-020-00472-8

Hong C, 2019, THERANOSTICS, V9, P4437, DOI 10.7150/thno.34953

Hu CMJ, 2015, NATURE, V526, P118, DOI 10.1038/nature15373

Hussein YR, 2011, TRANSL ONCOL, V4, P321, DOI 10.1593/tlo.11256

Iancu CV, 2013, P NATL ACAD SCI USA, V110, P17862, DOI 10.1073/pnas.1311485110

Jiang J, 2017, BIOMED PHARMACOTHER, V96, P619, DOI 10.1016/j.biopha.2017.10.043

Kaiser RD, 1998, BIOCHEMISTRY-US, V37, P8180, DOI 10.1021/bi980064a

Krzeslak A, 2012, PATHOL ONCOL RES, V18, P721, DOI 10.1007/s12253-012-9500-5

Phi LTH, 2019, ONCOTARGETS THER, V12, P10885, DOI 10.2147/OTT.S219063

Phi LTH, 2018, CANCER MED-US, V7, P5621, DOI 10.1002/cam4.1800

LHEUREUX GP, 1988, BIOPHYS CHEM, V30, P293, DOI 10.1016/0301-4622(88)85024-5

Li Jing, 2014, Chinese Journal of Biologicals, V27, P1633

Li R, 2020, J AGR FOOD CHEM, V68, P642, DOI 10.1021/acs.jafc.9b06460

Li W, 2009, ARCH PHARM RES, V32, P49, DOI 10.1007/s12272-009-1117-1

[刘继华 LIU Jihua], 2007, [中国药学杂志, Chinese Pharmaceutical Journal], V42, P1087

MURAMATSU K, 1994, INT J PHARM, V107, P1, DOI 10.1016/0378-5173(94)90296-8

Nelson ER, 2014, TRENDS ENDOCRIN MET, V25, P649, DOI 10.1016/j.tem.2014.10.001

Pan LL, 2019, EVID-BASED COMPL ALT, V2019, DOI 10.1155/2019/2417418

Park HJ, 2012, J GINSENG RES, V36, P225, DOI 10.5142/jgr.2012.36.3.225

Qi LW, 2010, BIOCHEM PHARMACOL, V80, P947, DOI 10.1016/j.bcp.2010.06.023

Quan K, 2015, SCI REP-UK, V5, DOI 10.1038/srep08598

Szebeni J, 2000, AM J PHYSIOL-HEART C, V279, pH1319, DOI 10.1152/ajpheart.2000.279.3.H1319

Thompson AMG, 2015, SCI REP-UK, V5, DOI 10.1038/srep12804

Zhu Y, 2021, J CONTROL RELEASE, V330, P641, DOI 10.1016/j.jconrel.2020.12.036

NR 31

TC 2

Z9 2

U1 11

U2 22

PU SHENYANG PHARMACEUTICAL UNIV

PI SHENYANG

PA SHENYANG PHARMACEUTICAL UNIV, NO 103, WENHUA RD, SHENYANG, 110016,

PEOPLES R CHINA

SN 1818-0876

J9 ASIAN J PHARM SCI

JI Asian J. Pharm. Sci.

PD MAR

PY 2022

VL 17

IS 2

BP 219

EP 229

DI 10.1016/j.ajps.2021.12.002

PG 11

WC Pharmacology & Pharmacy

WE Science Citation Index Expanded (SCI-EXPANDED)

SC Pharmacology & Pharmacy

GA 1K2IX

UT WOS:000798432200005

PM 35582640

OA gold, Green Published

DA 2023-04-05

ER

PT J

AU Song, C

Yuan, Y

Zhou, J

He, ZL

Hu, YY

Xie, Y

Liu, N

Wu, L

Zhang, J

AF Song, Chao

Yuan, Yue

Zhou, Jing

He, Ziliang

Hu, Yeye

Xie, Yuan

Liu, Nan

Wu, Lei

Zhang, Ji

TI Network Pharmacology-Based Prediction and Verification of Ginsenoside

Rh2-Induced Apoptosis of A549 Cells via the PI3K/Akt Pathway

SO FRONTIERS IN PHARMACOLOGY

LA English

DT Article

DE ginsenoside Rh2; network pharmacology; lung cancer; A549 cells; PI3K-Akt

signaling pathway

ID LUNG-CANCER; WEB SERVER; SIGNALING PATHWAY; PIK3CA MUTATIONS; RH2;

MECHANISMS; IDENTIFICATION; BINDING; ARREST; BREAST

AB Ginsenoside Rh2 (G-Rh2), a rare protopanaxadiol (PPD)-type triterpene saponin, from Panax ginseng has anti-proliferation, anti-invasion, and anti-metastatic activity. However, the mechanisms by which G-Rh2 induces apoptosis of lung cancer cells are unclear. In the present work, a G-Rh2 target-lung cancer network was constructed and analyzed by the network pharmacology approach. A total of 91 compound-targets of G-Rh2 was obtained based on the compound-target network analysis, and 217 targets were identified for G-Rh2 against lung cancer by PPI network analysis. The 217 targets were significantly enriched in 103 GO terms with FDR <0.05 as threshold in the GO enrichment analysis. In KEGG pathway enrichment analysis, all the candidate targets were significantly enriched in 143 pathways, among of which PI3K-Akt signaling pathway was identified as one of the top enriched pathway. Besides, G-Rh2 induced apoptosis in human lung epithelial (A549) cells was verified in this work. G-Rh2 significantly inhibited the proliferation of A549 cells in a dose-dependent manner, and the apoptosis rate significantly increased from 4.4% to 78.7% using flow cytometry. Western blot analysis revealed that the phosphorylation levels of p85, PDK1, Akt and I kappa B alpha were significantly suppressed by G-Rh2. All the experimental findings were consistent with the network pharmacology results. Research findings in this work will provide potential therapeutic value for further mechanism investigations.

C1 [Song, Chao; Zhou, Jing; He, Ziliang; Hu, Yeye; Xie, Yuan; Zhang, Ji] Huaiyin Normal Univ, Jiangsu Collaborat Innovat Ctr Reg Modern Agr & En, Sch Life Sci, Huaian, Peoples R China.

[Yuan, Yue] Tsinghua Univ, Inst Chinese Mat Med, Sch Pharmaceut Sci, Beijing, Peoples R China.

[Liu, Nan] Beijing Increasepharm Safety & Efficacy Co Ltd, Beijing, Peoples R China.

[Wu, Lei] Acad Sci, Inst Appl Chem, Nanchang, Peoples R China.

C3 Huaiyin Normal University; Institute of Chinese Materia Medica, CACMS;

Tsinghua University; Chinese Academy of Sciences

RP Zhang, J (通讯作者)，Huaiyin Normal Univ, Jiangsu Collaborat Innovat Ctr Reg Modern Agr & En, Sch Life Sci, Huaian, Peoples R China.; Liu, N (通讯作者)，Beijing Increasepharm Safety & Efficacy Co Ltd, Beijing, Peoples R China.; Wu, L (通讯作者)，Acad Sci, Inst Appl Chem, Nanchang, Peoples R China.

EM nanliu0304@163.com; wulei858196@163.com; zhangji@hytc.edu.cn

CR Albano D, 2021, INSIGHTS IMAGING, V12, DOI 10.1186/s13244-021-01017-2

An IS, 2013, ONCOL REP, V29, P523, DOI 10.3892/or.2012.2136

Bradner JE, 2017, CELL, V168, P629, DOI 10.1016/j.cell.2016.12.013

Brown JM, 2005, NAT REV CANCER, V5, P231, DOI 10.1038/nrc1560

Chen QY, 2017, INT IMMUNOPHARMACOL, V53, P149, DOI 10.1016/j.intimp.2017.10.025

Cheng CC, 2005, CANCER CHEMOTH PHARM, V55, P531, DOI 10.1007/s00280-004-0919-6

Cho WCS, 2013, EXPERT OPIN THER TAR, V17, P107, DOI 10.1517/14728222.2013.729043

Fei XF, 2002, ACTA PHARMACOL SIN, V23, P315

Ge GQ, 2017, BIOL PHARM BULL, V40, P2117, DOI 10.1248/bpb.b17-00463

Gfeller D, 2014, NUCLEIC ACIDS RES, V42, pW32, DOI 10.1093/nar/gku293

Han S, 2016, J ETHNOPHARMACOL, V194, P83, DOI 10.1016/j.jep.2016.08.039

Hanna Jennifer M, 2013, J Carcinog, V12, P6, DOI 10.4103/1477-3163.109033

Hayashi H, 2011, CLIN MED INSIGHTS-ON, V5, P177, DOI 10.4137/CMO.S6252

Hennessy BT, 2005, NAT REV DRUG DISCOV, V4, P988, DOI 10.1038/nrd1902

Houghton AM, 2010, NAT MED, V16, P219, DOI 10.1038/nm.2084

Huang DW, 2009, NAT PROTOC, V4, P44, DOI 10.1038/nprot.2008.211

Huang Q.M., 2021, J MOD ONCOL, V29, P4271, DOI [10.3969/j.issn.1672-4992.2021.24.002, DOI 10.3969/J.ISSN.1672-4992.2021.24.002]

Ito C, 2017, J SURG RES, V212, P195, DOI 10.1016/j.jss.2017.01.018

Jelovac D, 2014, HUM PATHOL, V45, P880, DOI 10.1016/j.humpath.2013.10.016

Jere D, 2009, INT J PHARMACEUT, V378, P194, DOI 10.1016/j.ijpharm.2009.05.046

Keiser MJ, 2007, NAT BIOTECHNOL, V25, P197, DOI 10.1038/nbt1284

Li Y, 2015, J ETHNOPHARMACOL, V175, P301, DOI 10.1016/j.jep.2015.09.016

Lim SM, 2020, IMMUNE NETW, V20, DOI 10.4110/in.2020.20.e10

Liu PX, 2009, NAT REV DRUG DISCOV, V8, P627, DOI 10.1038/nrd2926

Liu Y, 2021, MOL MED REP, V24, DOI 10.3892/mmr.2021.12513

LoRusso PM, 2016, J CLIN ONCOL, V34, P3803, DOI 10.1200/JCO.2014.59.0018

Martin A, 2010, BMC BIOINFORMATICS, V11, DOI 10.1186/1471-2105-11-91

Park EK, 2010, BRIT J PHARMACOL, V160, P1212, DOI 10.1111/j.1476-5381.2010.00768.x

Park SY, 2018, J GINSENG RES, V42, P98, DOI 10.1016/j.jgr.2017.09.001

Porter HA, 2013, CANCER LETT, V338, P239, DOI 10.1016/j.canlet.2013.03.030

Portugal J, 2018, BIOCHEM PHARMACOL, V155, P336, DOI 10.1016/j.bcp.2018.07.030

Sawa K, 2017, LUNG CANCER, V112, P96, DOI 10.1016/j.lungcan.2017.07.039

Scott DE, 2016, NAT REV DRUG DISCOV, V15, P533, DOI 10.1038/nrd.2016.29

Shi Xue-ping, 2017, Chinese Pharmacological Bulletin, V33, P114, DOI 10.3969/j.issn.1001-1978.2017.01.020

Solary E, 2000, LEUKEMIA, V14, P1833, DOI 10.1038/sj.leu.2401902

Song XQ, 2019, INT IMMUNOPHARMACOL, V74, DOI 10.1016/j.intimp.2019.105725

Sun PG, 2018, INFORM SCIENCES, V454, P229, DOI 10.1016/j.ins.2018.04.078

Szklarczyk D, 2016, NUCLEIC ACIDS RES, V44, pD380, DOI 10.1093/nar/gkv1277

Thirunavukkarasu T, 2018, INORG CHIM ACTA, V482, P229, DOI 10.1016/j.ica.2018.06.003

Tong-Lin Wu Tony, 2018, Oncotarget, V9, P11109, DOI 10.18632/oncotarget.24326

Wang X, 2017, NUCLEIC ACIDS RES, V45, pW356, DOI 10.1093/nar/gkx374

Wang YS, 2017, SCI REP-UK, V7, DOI 10.1038/s41598-017-12572-4

Wang YC, 2018, EXP THER MED, V15, P4916, DOI 10.3892/etm.2018.6067

Wild C.P., 2020, WORLD CANC REPORT CA

Wong AST, 2015, NAT PROD REP, V32, P256, DOI 10.1039/c4np00080c

Xie C, 2011, NUCLEIC ACIDS RES, V39, pW316, DOI 10.1093/nar/gkr483

Xie QP, 2017, BIOMED PHARMACOTHER, V85, P16, DOI 10.1016/j.biopha.2016.11.096

Yamamoto H, 2008, CANCER RES, V68, P6913, DOI 10.1158/0008-5472.CAN-07-5084

Yu T., 2021, CHIN J CLIN RES, V34, P248, DOI [10.13429/j.cnki.cjcr.2021.02.026, DOI 10.13429/J.CNKI.CJCR.2021.02.026]

Zhang Chunjing, 2011, Zhongguo Zhong Yao Za Zhi, V36, P1670

Zhang HB, 2021, ONCOL REP, V45, DOI 10.3892/or.2021.7984

Zhang J, 2019, FRONT PHARMACOL, V10, DOI 10.3389/fphar.2019.01331

Zhang YQ, 2017, CANCER CELL, V31, P820, DOI 10.1016/j.ccell.2017.04.013

Zhao RL, 2018, J ETHNOPHARMACOL, V210, P287, DOI 10.1016/j.jep.2017.08.041

Zhou ZC, 2020, EVID-BASED COMPL ALT, V2020, DOI 10.1155/2020/1646905

Zhu XB, 2018, BIOMED PHARMACOTHER, V102, P502, DOI 10.1016/j.biopha.2018.03.106

桑崇铃, 2007, [中国实用内科杂志, Chinese Journal of Practical Internal Medicine], V27, P1313

NR 57

TC 2

Z9 2

U1 12

U2 27

PU FRONTIERS MEDIA SA

PI LAUSANNE

PA AVENUE DU TRIBUNAL FEDERAL 34, LAUSANNE, CH-1015, SWITZERLAND

EI 1663-9812

J9 FRONT PHARMACOL

JI Front. Pharmacol.

PD MAY 4

PY 2022

VL 13

AR 878937

DI 10.3389/fphar.2022.878937

PG 10

WC Pharmacology & Pharmacy

WE Science Citation Index Expanded (SCI-EXPANDED)

SC Pharmacology & Pharmacy

GA 1J4YL

UT WOS:000797925500001

PM 35600856

OA gold, Green Published

DA 2023-04-05

ER

PT J

AU Hou, JG

Yun, Y

Cui, CH

Kim, S

AF Hou, Jingang

Yun, Yeejin

Cui, Changhao

Kim, Sunchang

TI Ginsenoside Rh2 mitigates doxorubicin-induced cardiotoxicity by

inhibiting apoptotic and inflammatory damage and weakening pathological

remodelling in breast cancer-bearing mice

SO CELL PROLIFERATION

LA English

DT Article

ID SUDDEN CARDIAC DEATH; SENESCENCE; FIBROSIS; CELLS

AB Objectives There are presently a few viable ways to reduce cardiotoxicity of doxorubicin (Dox). The combination of chemotherapy agents with natural compounds delivers greater efficacy and reduces adverse effects in recent researches for cancer treatment. Here, we examined the potential effect of ginsenoside Rh2 on a Dox-based regimen in chemotherapy treatment. Materials and Methods Human breast tumour (MDA-MB-231) xenograft nude mice, human cardiac ventricle fibroblasts, and human umbilical vein endothelial cells (HUVEC) were employed in the present study. Histology, immunohistochemistry, immunofluorescence, western blot, antibody array, and RNA-sequencing analyses were utilized to assess the protective effect of Rh2 on cardiotoxicity induced by Dox and the underlying mechanisms. Results Rh2-reduced cardiotoxicity by inhibiting the cardiac histopathological changes, apoptosis and necrosis, and consequent inflammation. Pathological remodelling was attenuated by reducing fibroblast to myofibroblast transition (FMT) and endothelial-mesenchymal transition (EndMT) in hearts. RNA-sequencing analysis showed that Dox treatment predominantly targets cell cycle and attachment of microtubules and boosted tumour necrosis, chemokine and interferon-gamma production, response to cytokine and chemokine, and T cell activation, whereas Rh2 regulated these effects. Intriguingly, Rh2 also attenuated fibrosis via promoting senescence in myofibroblasts and reversing established myofibroblast differentiation in EndMT. Conclusions Rh2 regulates multiple pathways in the Dox-provoked heart, proposing a potential candidate for cancer supplement and therapy-associated cardiotoxicity.

C1 [Hou, Jingang; Kim, Sunchang] Intelligent Synthet Biol Ctr, Daejeon, South Korea.

[Yun, Yeejin; Kim, Sunchang] Korea Adv Inst Sci & Technol, Dept Biol Sci, 291 Daehak Ro, Daejeon 34141, South Korea.

[Cui, Changhao] Sempio Foods Co, Res & Dev Team 4, Cheongju, South Korea.

C3 Korea Advanced Institute of Science & Technology (KAIST)

RP Kim, S (通讯作者)，Korea Adv Inst Sci & Technol, Dept Biol Sci, 291 Daehak Ro, Daejeon 34141, South Korea.

EM sunckim@kaist.ac.kr

FU Advancement of Active Biomedical material Project - Ministry of Trade

[P0014633]; Development of industrialization technology for crop virus

and pest Project - Ministry of Agriculture, Food and Rural Affairs

[321109041SB010]; Intelligent Synthetic Biology Center of the Global

Frontier Project - Ministry of Education, Science and Technology

[2011-0031955]; KAIST CrossGeneration Collaborative Lab project;

MultiDepartment Research and Business Development Program - Sejong city

[2021-153-0028-0019-002B]; Bio-Synergy Research Project of the Ministry

of Science, ICT and Future Planning through the National Research

Foundation [NRF-2021M3A9C4001028]

FX Advancement of Active Biomedical material Project, funded by the

Ministry of Trade, Grant/Award Number: P0014633; Development of

industrialization technology for crop virus and pest Project, funded by

the Ministry of Agriculture, Food and Rural Affairs, Grant/Award Number:

321109041SB010; Intelligent Synthetic Biology Center of the Global

Frontier Project, funded by the Ministry of Education, Science and

Technology, Grant/Award Number: 2011-0031955; KAIST CrossGeneration

Collaborative Lab project; MultiDepartment Research and Business

Development Program, funded by Sejong city, Grant/Award Number:

2021-153-0028-0019-002B; The Bio-Synergy Research Project of the

Ministry of Science, ICT and Future Planning through the National

Research Foundation, Grant/Award Number: NRF-2021M3A9C4001028

CR Baci D, 2020, INT J MOL SCI, V21, DOI 10.3390/ijms21197165

Bisogno G, 2018, LANCET ONCOL, V19, P1061, DOI 10.1016/S1470-2045(18)30337-1

Blagosklonny MV, 2004, CELL CYCLE, V3, P1035

Cardinale D, 2010, J AM COLL CARDIOL, V55, P213, DOI 10.1016/j.jacc.2009.03.095

Christidi E, 2021, CELL DEATH DIS, V12, DOI 10.1038/s41419-021-03614-x

Desai VD, 2014, PLOS ONE, V9, DOI 10.1371/journal.pone.0086865

Foucquier J, 2015, PHARMACOL RES PERSPE, V3, DOI 10.1002/prp2.149

Garrison G, 2013, AM J RESP CELL MOL, V48, P550, DOI 10.1165/rcmb.2012-0262OC

Gong T, 2020, NAT REV IMMUNOL, V20, P95, DOI 10.1038/s41577-019-0215-7

Gulati A, 2013, JAMA-J AM MED ASSOC, V309, P896, DOI 10.1001/jama.2013.1363

Gyongyosi M, 2020, CARDIOVASC RES, V116, P970, DOI 10.1093/cvr/cvz192

Hu C, 2019, ACTA PHARM SIN B, V9, P690, DOI 10.1016/j.apsb.2019.03.003

Ichikawa Y, 2014, J CLIN INVEST, V124, P617, DOI 10.1172/JCI72931

Khongorzul P, 2020, MOL CANCER RES, V18, P3, DOI 10.1158/1541-7786.MCR-19-0582

Levitt G, 2009, EUR J CANCER, V45, P3027, DOI 10.1016/j.ejca.2009.08.006

Lin SR, 2020, BRIT J PHARMACOL, V177, P1409, DOI 10.1111/bph.14816

Mamounas EP, 2005, J CLIN ONCOL, V23, P3686, DOI 10.1200/JCO.2005.10.517

Meyer K, 2016, J AM COLL CARDIOL, V67, P2018, DOI 10.1016/j.jacc.2016.02.047

Mohajeri M, 2018, CRIT REV ONCOL HEMAT, V122, P30, DOI 10.1016/j.critrevonc.2017.12.005

Munch J, 2016, J CARD FAIL, V22, P845, DOI 10.1016/j.cardfail.2016.03.010

Rocca A, 2021, THER ADV MED ONCOL, V13, DOI 10.1177/1758835920985632

Sachinidis A, 2020, CELLS-BASEL, V9, DOI 10.3390/cells9041001

Shin HJ, 2015, SCI REP-UK, V5, DOI 10.1038/srep15798

Siegel R.L., 2018, CA-CANCER J CLIN, V68, P7, DOI DOI 10.3322/caac.21442

Sonowal H, 2017, SCI REP-UK, V7, DOI 10.1038/s41598-017-03284-w

Spinale FG, 2013, CIRC RES, V112, P195, DOI 10.1161/CIRCRESAHA.112.266882

Sritharan S, 2021, LIFE SCI, V278, DOI 10.1016/j.lfs.2021.119527

Sweeney M, 2020, EMBO MOL MED, V12, DOI 10.15252/emmm.201910865

Wang DY, 2004, MOL ENDOCRINOL, V18, P402, DOI 10.1210/me.2003-0202

Wang YY, 2020, FRONT PHARMACOL, V11, DOI 10.3389/fphar.2020.00186

Yan C, 2021, AGING DIS, V12, P552, DOI [10.14336/AD.2021.0811, 10.14336/AD.2020.0811]

Zhang WL, 2015, J AM HEART ASSOC, V4, DOI 10.1161/JAHA.115.001993

NR 32

TC 6

Z9 6

U1 0

U2 4

PU WILEY

PI HOBOKEN

PA 111 RIVER ST, HOBOKEN 07030-5774, NJ USA

SN 0960-7722

EI 1365-2184

J9 CELL PROLIFERAT

JI Cell Prolif.

PD JUN

PY 2022

VL 55

IS 6

AR e13246

DI 10.1111/cpr.13246

EA MAY 2022

PG 15

WC Cell Biology

WE Science Citation Index Expanded (SCI-EXPANDED)

SC Cell Biology

GA 2G3RA

UT WOS:000792511700001

PM 35534947

OA Green Published

DA 2023-04-05

ER

PT J

AU Liu, G

Zhang, JL

Sun, FY

Ma, JT

Qi, XY

AF Liu, Guang

Zhang, Jinli

Sun, Fangyi

Ma, Jingtao

Qi, Xiaoyong

TI Ginsenoside Rg2 Attenuated Trastuzumab-Induced Cardiotoxicity in Rats

SO BIOMED RESEARCH INTERNATIONAL

LA English

DT Article

ID MEDIATED AMPK ACTIVATION; APOPTOSIS; PROTECTS; AUTOPHAGY; INJURY; DAMAGE

AB Aim. Trastuzumab (TZM) is a monoclonal antibody drug for HER2-positive breast cancer by targeting epidermal growth factor 2, but it has significant cardiotoxicity. Ginsenoside Rg2 has shown a variety of biological activities. This study was aimed at investigating whether Rg2 attenuates TZM-induced cardiotoxicity. Methods. A model of TZM-induced cardiotoxicity was established in Wistar rats, and the rats were pretreated with Rg2. After echocardiography analysis, the rats were killed and the hearts were dissected for RNAseq analysis. Primary human cardiomyocytes (HCMs) were treated with TZM with or without pretreatment with Rg2 and then subjected to a colony formation assay, flow cytometry analysis, and Western blot analysis for the detection of caspase-3, caspase-9, and BAX. Results. TZM induced LV dysfunction in rats, but Rg2 could attenuate TZM-induced LV dysfunction. The mRNA levels of caspase-3, caspase-9, and BAX were significantly higher in TZM-treated rats. The colony formation ability of HCMs was significantly lower in TZM-treated cells but was recovered after pretreatment with Rg2. The apoptosis rate of HCMs was significantly higher in TZM-treated cells but was significantly lower after pretreatment with Rg2. Moreover, protein levels of caspase-3, caspase-9, and BAX were significantly higher in TZM-treated cells but were significantly lower after pretreatment with Rg2. Conclusion. Ginsenoside Rg2 inhibited TZM-induced cardiotoxicity, and the mechanism may be related to the downregulation of the expression of proapoptotic proteins caspase-3, caspase-9, and BAX and the inhibition of TZM-induced apoptosis in cardiomyocytes. Ginsenoside Rg2 has a potential to be applied in patients with breast cancer to prevent TZM-induced cardiotoxicity.

C1 [Liu, Guang; Qi, Xiaoyong] Hebei Med Univ, Grad Sch, 361 East Zhongshan Rd, Shijiazhuang 050000, Hebei, Peoples R China.

[Liu, Guang; Zhang, Jinli; Sun, Fangyi; Ma, Jingtao] Hebei Med Univ, Cardiovasc Dept, Hosp 4, 12 Jiankang Rd, Shijiazhuang 050000, Hebei, Peoples R China.

[Qi, Xiaoyong] Hebei Prov Peoples Hosp, Ctr Cardiol, 348 West Heping Rd, Shijiazhuang 050000, Hebei, Peoples R China.

C3 Hebei Medical University; Hebei Medical University

RP Qi, XY (通讯作者)，Hebei Med Univ, Grad Sch, 361 East Zhongshan Rd, Shijiazhuang 050000, Hebei, Peoples R China.; Qi, XY (通讯作者)，Hebei Prov Peoples Hosp, Ctr Cardiol, 348 West Heping Rd, Shijiazhuang 050000, Hebei, Peoples R China.

EM 47126458@qq.com; 365130121@qq.com; ydsysfy2012@163.com;

jingtaom0502@163.com; qxi101010@gmail.com

FU Key Science and Technology Research Plan of Department of Health of

Hebei Province [20210481]

FX AcknowledgmentsThis study was supported by the fund from the Key Science

and Technology Research Plan of Department of Health of Hebei Province

(No. 20210481).

CR Abdollahpour-Alitappeh M, 2019, J CELL PHYSIOL, V234, P2693, DOI 10.1002/jcp.27085

Ahmed OM, 2020, BIOCELL, V44, P41, DOI 10.32604/biocell.2020.08157

Barile L, 2018, CARDIOVASC RES, V114, P992, DOI 10.1093/cvr/cvy055

Chen XJ, 2016, EVID-BASED COMPL ALT, V2016, DOI 10.1155/2016/5738694

Cheng B, 2020, J AGR FOOD CHEM, V68, P4215, DOI 10.1021/acs.jafc.0c00833

Chung Y, 2018, ANIM CELLS SYST, V22, P382, DOI 10.1080/19768354.2018.1545696

Cui J, 2021, J ETHNOPHARMACOL, V266, DOI 10.1016/j.jep.2020.113466

Cui J, 2017, CHEM-BIOL INTERACT, V275, P152, DOI 10.1016/j.cbi.2017.07.021

Fan YY, 2017, AUTOPHAGY, V13, P41, DOI 10.1080/15548627.2016.1240855

Fu WW, 2015, INT J CLIN EXP MED, V8, P19938

Hu ZH, 2021, BIOCELL, V45, P745, DOI 10.32604/biocell.2021.013293

Jeon H, 2021, ARCH PHARM RES, V44, P702, DOI 10.1007/s12272-021-01345-3

Jeon H, 2021, PHYTOMEDICINE, V85, DOI 10.1016/j.phymed.2021.153549

Liu G, 2021, EXP THER MED, V21, DOI 10.3892/etm.2021.9904

Mazzotta M, 2019, J CLIN MED, V8, DOI 10.3390/jcm8020254

Sato A, 2019, MOL CLIN ONCOL, V10, P37, DOI 10.3892/mco.2018.1764

Slamon D, 2011, NEW ENGL J MED, V365, P1273, DOI 10.1056/NEJMoa0910383

Xu QM, 2013, J FUNCT FOODS, V5, P2012, DOI 10.1016/j.jff.2013.08.005

Yaghoubi S, 2021, BREAST CANCER-TOKYO, V28, P216, DOI 10.1007/s12282-020-01153-5

Zhang GZ, 2008, J ETHNOPHARMACOL, V115, P441, DOI 10.1016/j.jep.2007.10.026

NR 20

TC 5

Z9 5

U1 0

U2 1

PU HINDAWI LTD

PI LONDON

PA ADAM HOUSE, 3RD FLR, 1 FITZROY SQ, LONDON, W1T 5HF, ENGLAND

SN 2314-6133

EI 2314-6141

J9 BIOMED RES INT

JI Biomed Res. Int.

PD JAN 12

PY 2022

VL 2022

AR 8866660

DI 10.1155/2022/8866660

PG 6

WC Biotechnology & Applied Microbiology; Medicine, Research & Experimental

WE Science Citation Index Expanded (SCI-EXPANDED)

SC Biotechnology & Applied Microbiology; Research & Experimental Medicine

GA 0B8UO

UT WOS:000774903300001

PM 35071601

OA gold, Green Published

DA 2023-04-05

ER

PT J

AU Zhao, MY

Liu, X

Hou, YZ

Yang, TT

Xu, JQ

Su, R

AF Zhao, Mingyue

Liu, Xin

Hou, Yuzhu

Yang, Tongtong

Xu, Jiaquan

Su, Rui

TI Combination of Electrochemistry and Mass Spectrometry to Study Nitric

Oxide Metabolism and Its Modulation by Compound K in Breast Cancer Cells

SO ANALYTICAL CHEMISTRY

LA English

DT Article

ID METABOLOMICS; ARGININE; GROWTH; SENSOR

AB The levels of L-arginine and asymmetric dimethylarginine(ADMA) and the amount of the nitric oxide (NO) production have recently been linked to breast cancer and pharmaceutical effect evaluation. Herein, a method combining electrochemistry and high-resolution mass spectrometry(HRMS) was established and used to study NO metabolism and its modulation by ginsenoside compound K (CK) in breast cancer cells. Platinum nanoparticles-decorated fluorine tin oxide was employed as an electrochemical sensor for in situ detection of NO release, while HRMS was used for the analysis of the NO-related metabolites. Through the combination of the electrochemical and HRMS results, decreases in arginine and NO and increases in ADMA andornithine were observed after modulation by CK, and two highly correlated metabolic pathways including arginine and proline metabolism and vascular smooth muscle contraction were found. This method offers a new strategy for fast evaluation of pharmaceutical efficacy based on NO metabolism.

C1 [Zhao, Mingyue; Liu, Xin; Hou, Yuzhu; Yang, Tongtong] Changchun Univ Chinese Med, Jilin Ginseng Acad, Changchun 130017, Peoples R China.

[Xu, Jiaquan] East China Univ Technol, Jiangxi Key Lab Mass Spectrometry & Instrumentat, Nanchang 330013, Jiangxi, Peoples R China.

[Su, Rui] Jilin Univ, Coll Chem, State Key Lab Inorgan Synth & Preparat Chem, Changchun 130012, Peoples R China.

C3 Changchun University of Chinese Medicine; East China University of

Technology; Jilin University

RP Xu, JQ (通讯作者)，East China Univ Technol, Jiangxi Key Lab Mass Spectrometry & Instrumentat, Nanchang 330013, Jiangxi, Peoples R China.; Su, R (通讯作者)，Jilin Univ, Coll Chem, State Key Lab Inorgan Synth & Preparat Chem, Changchun 130012, Peoples R China.

EM jiaquan_xu@foxmail.com; rsu@jlu.edu.cn

FU National Natural Science Foundation of China (NSFC) [82004005,

21727812]; Jilin Province Science and Technology Development Plan

[202002056JC]; Jilin Province Education Department Science and

Technology Research Project [JJKH20210942KJ]

FX This work was supported by the National Natural Science Foundation of

China (NSFC; nos. 82004005 and 21727812), the Jilin Province Science and

Technology Development Plan (no. 202002056JC), and the Jilin Province

Education Department Science and Technology Research Project (no.

JJKH20210942KJ).

CR Aerts JT, 2014, ANAL CHEM, V86, P3203, DOI 10.1021/ac500168d

Alshamleh I, 2020, ANGEW CHEM INT EDIT, V59, P2304, DOI 10.1002/anie.201912919

Baira E, 2018, FOOD CHEM, V269, P276, DOI 10.1016/j.foodchem.2018.06.146

Basudhar D, 2017, P NATL ACAD SCI USA, V114, P13030, DOI 10.1073/pnas.1709119114

Boger RH, 2003, VASC MED, V8, P149, DOI 10.1191/1358863x03vm501ed

Choi E, 2019, J GINSENG RES, V43, P692, DOI 10.1016/j.jgr.2019.07.001

Cooke JP, 2006, EUR J CLIN PHARMACOL, V62, P115, DOI 10.1007/s00228-005-0005-y

Dayoub H, 2003, CIRCULATION, V108, P3042, DOI 10.1161/01.CIR.0000101924.04515.2E

Evans MA, 2018, CHEM SCI, V9, P3729, DOI 10.1039/c8sc00015h

Finkelman BS, 2017, J AM COLL CARDIOL, V70, P152, DOI 10.1016/j.jacc.2017.05.019

Flaherty RL, 2019, CANCER LETT, V459, P59, DOI 10.1016/j.canlet.2019.05.027

Fujita M, 2019, REDOX BIOL, V22, DOI 10.1016/j.redox.2019.101158

Fukumura D, 2006, NAT REV CANCER, V6, P521, DOI 10.1038/nrc1910

Gacche RN, 2018, DRUG RESIST UPDATE, V36, P47, DOI 10.1016/j.drup.2018.01.002

Garg SK, 2020, FRONT IMMUNOL, V11, DOI 10.3389/fimmu.2020.00164

Huang HL, 2015, ONCOL REP, V34, P3131, DOI 10.3892/or.2015.4280

Huang JQ, 2019, J GERONTOL A-BIOL, V74, P853, DOI 10.1093/gerona/gly128

IGLESIAS A, 2016, ANALYST, V141, P3776, DOI DOI 10.1039/C6AN00170J

Jacobi J, 2005, CIRCULATION, V111, P1431, DOI 10.1161/01.CIR.0000158487.80483.09

Kang FH, 2018, CHEM SCI, V9, DOI 10.1039/c8sc00167g

Khan KA, 2018, NAT REV CLIN ONCOL, V15, P310, DOI 10.1038/nrclinonc.2018.9

Lee Y, 2004, ANAL CHEM, V76, P545, DOI 10.1021/ac035065+

Li CM, 2006, ELECTROANAL, V18, P713, DOI 10.1002/elan.200503457

Li RF, 2020, NAT COMMUN, V11, DOI 10.1038/s41467-020-17008-8

Li Y, 2017, J AM CHEM SOC, V139, P13055, DOI 10.1021/jacs.7b06476

Manna SK, 2014, GASTROENTEROLOGY, V146, P1313, DOI 10.1053/j.gastro.2014.01.017

Morris SM, 2016, J NUTR, V146, P2579, DOI 10.3945/jn.115.226621

Muller C, 2019, NAT PROTOC, V14, P2546, DOI 10.1038/s41596-019-0193-z

PALMER RMJ, 1988, NATURE, V333, P664, DOI 10.1038/333664a0

Peng H, 2020, ONCOGENE, V39, P6747, DOI 10.1038/s41388-020-01480-z

Phang JM, 2019, ANTIOXID REDOX SIGN, V30, P635, DOI 10.1089/ars.2017.7350

Ryk Charlotta, 2015, Redox Biol, V5, P419, DOI 10.1016/j.redox.2015.09.029

Schmidt HHHW, 2019, CIRCULATION, V139, P2664, DOI 10.1161/CIRCULATIONAHA.119.040423

Shao W, 2014, MOL CANCER, V13, DOI 10.1186/1476-4598-13-197

Shestakova KM, 2020, MOLECULES, V25, DOI 10.3390/molecules25245896

Shibasaki M, 2008, J APPL PHYSIOL, V105, P1504, DOI 10.1152/japplphysiol.91017.2008

Shukla SK, 2017, CANCER CELL, V32, P71, DOI 10.1016/j.ccell.2017.06.004

Somasundaram V, 2019, ANTIOXID REDOX SIGN, V30, P1124, DOI 10.1089/ars.2018.7527

Song YY, 2019, SCI TOTAL ENVIRON, V651, P3139, DOI 10.1016/j.scitotenv.2018.10.171

Sung H, 2021, CA-CANCER J CLIN, V71, P209, DOI [10.3322/caac.21492, 10.3322/caac.21660, 10.3322/caac.21442]

Sung YC, 2019, NAT NANOTECHNOL, V14, P1160, DOI 10.1038/s41565-019-0570-3

Wang CY, 2020, AM J CANCER RES, V10, P95

Wang L, 2019, NANO LETT, V19, P6800, DOI 10.1021/acs.nanolett.9b01869

Wells SM, 2007, AM J RESP CELL MOL, V36, P520, DOI 10.1165/rcmb.2006-0302SM

Xu JQ, 2015, ANGEW CHEM INT EDIT, V54, P14402, DOI 10.1002/anie.201507354

Yan GL, 2013, PLOS ONE, V8, DOI 10.1371/journal.pone.0073839

Yan YT, 2018, J IMMUNOTHER CANCER, V6, DOI 10.1186/s40425-018-0466-z

Zhan JM, 2020, CARBOHYD POLYM, V230, DOI 10.1016/j.carbpol.2019.115576

Zhang XW, 2017, ANGEW CHEM INT EDIT, V56, P12997, DOI 10.1002/anie.201707187

Zheng FJ, 2020, NAT PROTOC, V15, P2519, DOI 10.1038/s41596-020-0341-5

Zhong ZH, 2019, TALANTA, V204, P6, DOI 10.1016/j.talanta.2019.05.088

Zhu Z, 2016, ONCOGENE, V35, P1399, DOI 10.1038/onc.2015.197

NR 52

TC 0

Z9 0

U1 18

U2 36

PU AMER CHEMICAL SOC

PI WASHINGTON

PA 1155 16TH ST, NW, WASHINGTON, DC 20036 USA

SN 0003-2700

EI 1520-6882

J9 ANAL CHEM

JI Anal. Chem.

PD MAR 29

PY 2022

VL 94

IS 12

BP 5122

EP 5131

DI 10.1021/acs.analchem.1c05492

PG 10

WC Chemistry, Analytical

WE Science Citation Index Expanded (SCI-EXPANDED)

SC Chemistry

GA 0I3EH

UT WOS:000779305600031

PM 35306816

DA 2023-04-05

ER

PT J

AU Jin, YJ

Huynh, DTN

Heo, KS

AF Jin, Yujin

Diem Thi Ngoc Huynh

Heo, Kyung-Sun

TI Ginsenoside Rh1 inhibits tumor growth in MDA-MB-231 breast cancer cells

via mitochondrial ROS and ER stress-mediated signaling pathway

SO ARCHIVES OF PHARMACAL RESEARCH

LA English

DT Article

DE Cell proliferation; ER stress; Ginsenoside Rh1; Mitochondrial ROS;

Triple negative breast cancer cells

ID ACTIVATION; 5-FLUOROURACIL; MIGRATION

AB Ginsenoside-Rh1 (Rh1) is a ginseng-derived compound that has been reported to exert anticancer effects by regulating cell cycle arrest and apoptosis according to reactive oxygen species (ROS) production. However, the effects of Rh1 on mitochondrial dysfunction are involved in triple negative breast cancer (TNBC) cell apoptosis, and the related molecular mechanisms remain unknown. Rh1 treatment induced cell toxicity less than 50% at 50 mu M. In addition, Rh1 induced apoptosis in TNBC cells through cleaved caspase-3 activation and G1/S arrest. The Rh1-treated TNBC cells showed a significant increase in mitochondrial ROS (mtROS), which in turn increased protein expression of mitochondrial molecules, such as Bak and cytochrome C, and caused the loss of mitochondrial membrane potential. Pretreatment with mitochondria-targeted antioxidant Mito-TEMPO alters the Rh1-reduced rate of mito- and glycol-ATP. Furthermore, Rh1 induces ER stress-mediated calcium accumulation via PERK/eIF2 alpha/ATF4/CHOP pathway. Inhibition of ATF4 by siRNA transfection significantly inhibited Rh1-mediated apoptosis and calcium production. Interestingly, Mito-TEMPO treatment significantly reduced apoptosis and ER stress induced by Rh1. Finally, Rh1 at 5 mg/kg suppressed tumor growth through increased levels of ROS production, cleaved caspase-3, and ATF4 more than 5-fluorouracil treated group. Overall, our results suggest that Rh1 has potential for use in TNBC treatment.

C1 [Jin, Yujin; Diem Thi Ngoc Huynh; Heo, Kyung-Sun] Chungnam Natl Univ, Coll Pharm, 99 Daehak Ro, Daejeon, South Korea.

C3 Chungnam National University

RP Heo, KS (通讯作者)，Chungnam Natl Univ, Coll Pharm, 99 Daehak Ro, Daejeon, South Korea.

EM kheo@cnu.ac.kr

OI Heo, Kyung-Sun/0000-0003-3800-7665

FU National Research Foundation of Korea (NRF) - Ministry of Science, ICT

and Future Planning [2017R1A4A1015860, 2019R1C1C100733112]

FX This research was supported by National Research Foundation of Korea

(NRF) funded by the Ministry of Science, ICT and Future Planning

(2017R1A4A1015860 and 2019R1C1C100733112).

CR Ahn J, 2020, ARCH PHARM RES, V43, P735, DOI 10.1007/s12272-020-01254-x

Chen W, 2018, J EXP CLIN CANC RES, V37, DOI 10.1186/s13046-018-0987-9

Deng WJ, 2018, J CELL MOL MED, V22, P3108, DOI 10.1111/jcmm.13588

Huynh DTN, 2019, ARCH PHARM RES, V42, P848, DOI 10.1007/s12272-019-01180-7

Duan ZG, 2018, BIOCHEM BIOPH RES CO, V499, P482, DOI 10.1016/j.bbrc.2018.03.174

Ghosh S, 2019, BIOMED PHARMACOTHER, V114, DOI 10.1016/j.biopha.2019.108855

Huynh DTN, 2021, CANCERS, V13, DOI 10.3390/cancers13081892

Jeon H, 2021, ARCH PHARM RES, V44, P702, DOI 10.1007/s12272-021-01345-3

Jeon H, 2021, PHYTOMEDICINE, V85, DOI 10.1016/j.phymed.2021.153549

Jiao YL, 2019, ARCH PHARM RES, V42, P1092, DOI 10.1007/s12272-019-01197-y

Jin YJ, 2021, INT J MOL SCI, V22, DOI 10.3390/ijms221910458

Jin Y, 2020, ARCH PHARM RES, V43, P773, DOI [10.1007/s12272-020-01265-8, 10.1007/s12272-020-01255-w]

Jin Y, 2019, BMB REP, V52, P706, DOI 10.5483/BMBRep.2019.52.12.234

Kim C, 2018, NUTRIENTS, V10, DOI 10.3390/nu10081021

Kim J, 2016, EXP MOL MED, V48, DOI 10.1038/emm.2016.119

Kumar P, 2016, ARCH GYNECOL OBSTET, V293, P247, DOI 10.1007/s00404-015-3859-y

Lastraioli E, 2020, FRONT PHARMACOL, V11, DOI 10.3389/fphar.2020.00725

Lee Y, 2003, J STEROID BIOCHEM, V84, P463, DOI 10.1016/S0960-0760(03)00067-0

Lee YJ, 2019, ARCH PHARM RES, V42, P531, DOI 10.1007/s12272-019-01158-5

Li YL, 2019, FRONT PHARMACOL, V10, DOI 10.3389/fphar.2019.01195

Longley DB, 2003, NAT REV CANCER, V3, P330, DOI 10.1038/nrc1074

Lyu X, 2019, ONCOL LETT, V18, P4160, DOI 10.3892/ol.2019.10742

Ma CB, 2019, MOL CELLS, V42, P628, DOI 10.14348/molcells.2019.0038

Mathiyalagan R, 2019, MOLECULES, V24, DOI 10.3390/molecules24234367

Murphy MP, 2009, BIOCHEM J, V417, P1, DOI 10.1042/BJ20081386

Nguyen TL, 2021, ARCH PHARM RES, V44, P241, DOI 10.1007/s12272-020-01304-4

Park JH, 2019, CANCER SCI, V110, P2834, DOI 10.1111/cas.14124

Rozpedek W, 2016, CURR MOL MED, V16, P533, DOI 10.2174/1566524016666160523143937

Su PY, 2020, ONCOTARGETS THER, V13, P5207, DOI 10.2147/OTT.S242820

Tripathi SK, 2020, ARCH PHARM RES, V43, P242, DOI 10.1007/s12272-020-01221-6

Yang YH, 2016, J CELL PHYSIOL, V231, P2570, DOI 10.1002/jcp.25349

Yoon JH, 2012, EUR J PHARMACOL, V679, P24, DOI 10.1016/j.ejphar.2012.01.020

Zhu J, 2015, INT J ONCOL, V46, P981, DOI 10.3892/ijo.2015.2819

Zorov DB, 2014, PHYSIOL REV, V94, P909, DOI 10.1152/physrev.00026.2013

NR 34

TC 4

Z9 4

U1 7

U2 11

PU PHARMACEUTICAL SOC KOREA

PI SEOUL

PA 1489-3 SUHCHO-DONG, SUHCHO-KU, SEOUL 137-071, SOUTH KOREA

SN 0253-6269

EI 1976-3786

J9 ARCH PHARM RES

JI Arch. Pharm. Res.

PD MAR

PY 2022

VL 45

IS 3

BP 174

EP 184

DI 10.1007/s12272-022-01377-3

EA MAR 2022

PG 11

WC Chemistry, Medicinal; Pharmacology & Pharmacy

WE Science Citation Index Expanded (SCI-EXPANDED)

SC Pharmacology & Pharmacy

GA 0D5VH

UT WOS:000772718700001

PM 35325393

DA 2023-04-05

ER

PT J

AU Li, YW

He, F

Zhang, Y

Pan, ZY

AF Li, YanWei

He, Feng

Zhang, Yu

Pan, ZhanYu

TI Apatinib and Ginsenoside-Rb1 Synergetically Control the Growth of

Hypopharyngeal Carcinoma Cells

SO DISEASE MARKERS

LA English

DT Article

ID BREAST-CANCER; EFFICACY; THERAPY; GINSENG; SAFETY; HEAD

AB Background. Apatinib is an anticancer drug known to inhibit the vascular endothelial growth factor receptor-2 (VEGFR-2) through regulating tyrosine kinases. Drug resistance and reduced activity in various cancers is the matter of great concern; thus, researchers opt to use combination of the two or more drugs. So far, its gynergetic anticancer role with a traditional Chinese drug Ginsenoside-Rb1 (G-Rb1) has not been studied in cancers including hypopharyngeal carcinoma. Objective. The current study is aimed at investigating the anticancer synergetic effects of G-Rb1 and apatinib in hypopharyngeal carcinoma. Methods. The synergetic effects of both drugs on cell proliferation, wound healing and cell migration, and cell apoptosis were studied in hypopharyngeal carcinoma cells. Furthermore, the xenograft rat model was generated, and tumor inhibition was monitored after treating rats with both drugs as mono- and combination therapy. In addition, protein expression and localization were performed by western blotting and immunofluorescent staining, respectively. Results. The analyses of the data showed that combination therapy of apatinib and G-Rb1 significantly inhibited the proliferation, migration, and wound healing capability of hypopharyngeal carcinoma cells. Moreover, the glycolysis rate of the cells in the combination therapy (apatinib and G-Rb1) group was significantly decreased as compared to that in the monotherapy group or no treatment group, suggesting that the glycolysis inhibition led to the inhibition of tumor growth. Moreover, the combination therapy on xenograft rats dramatically reduced the tumor size. Furthermore, combination therapy also exhibited an increased count of CD3(+) and CD4(+) T cells, as well as the ratio between CD4(+) and CD8(+) T cells. Conclusion. Interestingly, a combination of apatinib and G-Rb1 induced more tumor cell apoptosis and reduced cell proliferation than the individual drug treatment and promote antitumor immunity by enhancing immunomodulatory molecules. Thus, we believe that this study could serve as a valuable platform to assess the synergetic anticancer effects of the herbal as well as synthetic medicines.

C1 [Li, YanWei; He, Feng] Tianjin Univ, Acad Med Engn & Translat Med, Tianjin 300192, Peoples R China.

[Li, YanWei; He, Feng] Tianjin Univ, Tianjin Key Lab Brain Sci & Neural Engn, Tianjin 300192, Peoples R China.

[Li, YanWei; Zhang, Yu; Pan, ZhanYu] Tianjin Med Univ, Dept Integrat Oncol, Canc Inst & Hosp, Tianjin 300060, Peoples R China.

[Li, YanWei; Zhang, Yu; Pan, ZhanYu] Key Lab Canc Prevent & Therapy, Tianjin 300060, Peoples R China.

C3 Tianjin University; Tianjin University; Tianjin Medical University

RP Li, YW (通讯作者)，Tianjin Univ, Acad Med Engn & Translat Med, Tianjin 300192, Peoples R China.; Li, YW (通讯作者)，Tianjin Univ, Tianjin Key Lab Brain Sci & Neural Engn, Tianjin 300192, Peoples R China.; Li, YW (通讯作者)，Tianjin Med Univ, Dept Integrat Oncol, Canc Inst & Hosp, Tianjin 300060, Peoples R China.; Li, YW (通讯作者)，Key Lab Canc Prevent & Therapy, Tianjin 300060, Peoples R China.

EM liyanwei127@hotmail.com; 498919592@qq.com; zhangyu1441@sina.om;

13920434704@139.com

RI he, feng/HOF-1989-2023; li, yan/GTI-4638-2022; He, Feng/HHS-6457-2022

OI He, Feng/0000-0002-1996-0744

FU Youth Fund of National Natural Science Foundation of China [81503622]

FX AcknowledgmentsThe current study was supported by the Youth Fund of

National Natural Science Foundation of China (grant number 81503622). We

highly acknowledge the support of the colleagues and institutes

participated in the study.

CR An MY, 2021, ANTIOXIDANTS-BASEL, V10, DOI 10.3390/antiox10010062

Chen LH, 2019, CELL ONCOL, V42, P679, DOI 10.1007/s13402-019-00455-x

Deng XQ, 2020, BIOCHEM PHARMACOL, V178, DOI 10.1016/j.bcp.2020.114038

Derakhshan S, 2021, MOL BIOL REP, V48, P3223, DOI 10.1007/s11033-021-06341-w

Ding Xinjing, 2021, Mol Clin Oncol, V15, P151, DOI 10.3892/mco.2021.2313

Fan MH, 2014, BREAST CANCER RES TR, V143, P141, DOI 10.1007/s10549-013-2793-6

Feng YL, 2021, J PAK MED ASSOC, V71, P1025, DOI 10.47391/JPMA.1442

Geng RX, 2018, EXPERT OPIN DRUG SAF, V17, P1145, DOI 10.1080/14740338.2018.1535592

Irfan M, 2020, J GINSENG RES, V44, P538, DOI 10.1016/j.jgr.2020.03.001

Kim J, 2018, FOOD SCI BIOTECHNOL, V27, P227, DOI 10.1007/s10068-017-0255-3

Kumar Dhruv, 2017, Postdoc J, V5, P14

Li YW, 2018, EVID-BASED COMPL ALT, V2018, DOI 10.1155/2018/5291517

Liao X, 2020, GENES DIS, V7, P370, DOI 10.1016/j.gendis.2019.10.016

Liu KS, 2017, CELL DEATH DIS, V8, DOI 10.1038/cddis.2017.422

Liu ZY, 2021, FRONT ONCOL, V11, DOI 10.3389/fonc.2021.643654

Lu HX, 2017, FRONT PHARMACOL, V8, DOI 10.3389/fphar.2017.00783

Lu S, 2020, BMC COMPLEMENT MED, V20, DOI 10.1186/s12906-019-2797-9

Lv XX, 2021, ANTI-CANCER DRUG, V32, P773, DOI 10.1097/CAD.0000000000001102

Meng M, 2019, J CANCER RES THER, V15, P442, DOI 10.4103/jcrt.JCRT_894_18

Peng QX, 2017, ONCOTARGET, V8, P52813, DOI 10.18632/oncotarget.17264

Rajput SA, 2021, ECOTOX ENVIRON SAFE, V220, DOI 10.1016/j.ecoenv.2021.112333

Santoro R, 2008, HEAD NECK-J SCI SPEC, V30, P1483, DOI 10.1002/hed.20907

Sato K, 2015, EUR ARCH OTO-RHINO-L, V272, P2001, DOI 10.1007/s00405-014-3132-1

Scott AJ, 2015, DRUG TODAY, V51, P223, DOI 10.1358/dot.2015.51.4.2320599

Scott LJ, 2018, DRUGS, V78, P747, DOI 10.1007/s40265-018-0903-9

Shoemaker M, 2005, PHYTOTHER RES, V19, P649, DOI 10.1002/ptr.1702

Varghese BT, 2009, ACTA OTO-LARYNGOL, V129, P1480, DOI 10.3109/00016480902748520

Wang B, 2021, MICROB PATHOGENESIS, V157, DOI 10.1016/j.micpath.2021.105002

Wang L, 2021, AM J TRANSL RES, V13, P4704

Wang MT, 2020, J DRUG TARGET, V28, P961, DOI 10.1080/1061186X.2020.1764963

Wang YM, 2021, CELL BIOSCI, V11, DOI 10.1186/s13578-021-00640-2

Xu JM, 2019, CLIN CANCER RES, V25, P515, DOI 10.1158/1078-0432.CCR-18-2484

Yin Q, 2021, FOOD FUNCT, V12, P5301, DOI [10.1039/D1FO00348H, 10.1039/d1fo00348h]

Zhang HM, 2015, CYTOKINE, V76, P549, DOI 10.1016/j.cyto.2015.06.019

Zhang JH, 2021, AM J CHINESE MED, V49, P1195, DOI 10.1142/S0192415X21500579

Zhao DZ, 2018, ONCOTARGETS THER, V11, P4137, DOI 10.2147/OTT.S172305

Zhao H, 2020, J OVARIAN RES, V13, DOI 10.1186/s13048-020-00719-3

Zhao XG, 2015, PLOS ONE, V10, DOI 10.1371/journal.pone.0126147

Zhu GX, 2021, PHARMACOL RES, V169, DOI 10.1016/j.phrs.2021.105647

Zhu H, 2021, J ETHNOPHARMACOL, V265, DOI 10.1016/j.jep.2020.113271

NR 40

TC 4

Z9 4

U1 8

U2 12

PU HINDAWI LTD

PI LONDON

PA ADAM HOUSE, 3RD FLR, 1 FITZROY SQ, LONDON, W1T 5HF, ENGLAND

SN 0278-0240

EI 1875-8630

J9 DIS MARKERS

JI Dis. Markers

PD JAN 13

PY 2022

VL 2022

AR 3833489

DI 10.1155/2022/3833489

PG 14

WC Biotechnology & Applied Microbiology; Genetics & Heredity; Medicine,

Research & Experimental; Pathology

WE Science Citation Index Expanded (SCI-EXPANDED)

SC Biotechnology & Applied Microbiology; Genetics & Heredity; Research &

Experimental Medicine; Pathology

GA ZR1FD

UT WOS:000767537200001

PM 35069931

OA Green Published, gold

DA 2023-04-05

ER

PT J

AU Xia, JX

Ma, SJ

Zhu, X

Chen, C

Zhang, R

Cao, ZL

Chen, X

Zhang, LL

Zhu, Y

Zhang, SY

Li, SY

Gu, GL

Wei, XB

Yu, KQ

Wang, JX

AF Xia, Jiaxuan

Ma, Shaojie

Zhu, Xi

Chen, Chen

Zhang, Ru

Cao, Zhonglian

Chen, Xing

Zhang, Longlong

Zhu, Ying

Zhang, Shuya

Li, Shiyi

Gu, Guolong

Wei, Xunbin

Yu, Kunqian

Wang, Jianxin

TI Versatile ginsenoside Rg3 liposomes inhibit tumor metastasis by

capturing circulating tumor cells and destroying metastatic niches

SO SCIENCE ADVANCES

LA English

DT Article

ID NF-KAPPA-B; RESISTANT; COMBINATION; PACLITAXEL; STRATEGIES; THERAPY

AB Limited circulating tumor cells (CTCs) capturing efficiency and lack of regulation capability on CTC-supportive metastatic niches (MNs) are two main obstacles hampering the clinical translation of conventional liposomes for the treatment of metastatic breast cancers. Traditional delivery strategies, such as ligand modification and immune modulator co-encapsulation for nanocarriers, are inefficient and laborious. Here, a multifunctional Rg3 liposome loading with docetaxel (Rg3-Lp/DTX) was developed, in which Rg3 was proved to intersperse in the phospholipid bilayer and exposed its glycosyl on the liposome surface. Therefore, it exhibited much higher CTC- capturing efficiency via interaction with glucose transporter 1 (Glut1) overexpressed on CTCs. After reaching the lungs with CTCs, Rg3 inhibited the formation of MNs by reversing the immunosuppressive microenvironment. Together, Rg3-Lp/DTX exhibited excellent metastasis inhibition capacity by CTC ("seeds") neutralization and MN ("soil") inhibition. The strategy has great clinical translation prospects for antimetastasis treatment with enhanced therapeutic efficacy and simple preparation process.

C1 [Xia, Jiaxuan; Chen, Chen; Zhang, Ru; Cao, Zhonglian; Chen, Xing; Zhang, Longlong; Zhu, Ying; Zhang, Shuya; Li, Shiyi; Gu, Guolong; Wang, Jianxin] Fudan Univ, Sch Pharm, Dept Pharmaceut, Shanghai 201203, Peoples R China.

[Xia, Jiaxuan; Chen, Chen; Zhang, Ru; Cao, Zhonglian; Chen, Xing; Zhang, Longlong; Zhu, Ying; Zhang, Shuya; Li, Shiyi; Gu, Guolong; Wang, Jianxin] Minist Educ, Key Lab Smart Drug Delivery, Shanghai 201203, Peoples R China.

[Ma, Shaojie; Yu, Kunqian] Chinese Acad Sci, Drug Discovery & Design Ctr, Shanghai Inst Mat Med, State Key Lab Drug Res, Shanghai 201203, Peoples R China.

[Ma, Shaojie] Huazhong Univ Sci & Technol, Coll Life Sci & Technol, Key Lab Mol Biophys, Minist Educ, Wuhan 430071, Peoples R China.

[Zhu, Xi; Wei, Xunbin] Shanghai Jiao Tong Univ, Med X Res Inst, Shanghai Canc Inst, State Key Lab Oncogenes & Related Genes, Shanghai 200030, Peoples R China.

[Zhu, Xi; Wei, Xunbin] Shanghai Jiao Tong Univ, Sch Biomed Engn, Shanghai 200030, Peoples R China.

[Wang, Jianxin] Fudan Univ, Inst Integrat Med, Shanghai 201203, Peoples R China.

C3 Fudan University; Chinese Academy of Sciences; Shanghai Institute of

Materia Medica, CAS; Huazhong University of Science & Technology;

Shanghai Jiao Tong University; Shanghai Jiao Tong University; Fudan

University

RP Wang, JX (通讯作者)，Fudan Univ, Sch Pharm, Dept Pharmaceut, Shanghai 201203, Peoples R China.; Wang, JX (通讯作者)，Minist Educ, Key Lab Smart Drug Delivery, Shanghai 201203, Peoples R China.; Wang, JX (通讯作者)，Fudan Univ, Inst Integrat Med, Shanghai 201203, Peoples R China.

EM jxwang@fudan.edu.cn

OI Ma, Shaojie/0000-0002-7951-0104; Xia, jiaxuan/0000-0002-5969-3812

FU National Natural Science Foundation of China [82074277, 8177391,

81690263]; Development Project of Shanghai Peak Disciplines-Integrated

Medicine [20180101]; National Key R&D Program of China [2020YFC0841400];

Strategic Priority Research Program of the Chinese Academy of Sciences

[XDPB2505]

FX We are thankful for financial support from the National Natural Science

Foundation of China (nos. 82074277, 8177391, and 81690263), the

Development Project of Shanghai Peak Disciplines-Integrated Medicine

(no. 20180101), National Key R&D Program of China (2020YFC0841400), and

the Strategic Priority Research Program of the Chinese Academy of

Sciences (grant XDPB2505).

CR Arrieta O, 2020, JAMA ONCOL, V6, P856, DOI 10.1001/jamaoncol.2020.0409

Balayla J, 2020, J ASSIST REPROD GEN, V37, P913, DOI 10.1007/s10815-020-01730-9

Baud V, 2009, NAT REV DRUG DISCOV, V8, P33, DOI 10.1038/nrd2781

Bianchini G, 2016, NAT REV CLIN ONCOL, V13, P674, DOI 10.1038/nrclinonc.2016.66

Commander R, 2020, NAT COMMUN, V11, DOI 10.1038/s41467-020-15219-7

Crabb SJ, 2020, J CLIN ONCOL, V38

Echeverria GV, 2019, SCI TRANSL MED, V11, DOI 10.1126/scitranslmed.aav0936

Eckhardt BL, 2012, NAT REV DRUG DISCOV, V11, P479, DOI 10.1038/nrd2372

Eloy JO, 2014, COLLOID SURFACE B, V123, P345, DOI 10.1016/j.colsurfb.2014.09.029

Feng HR, 2020, CELL DEATH DIS, V11, DOI 10.1038/s41419-020-03111-7

GALLAY J, 1984, BIOCHIM BIOPHYS ACTA, V769, P96, DOI 10.1016/0005-2736(84)90013-0

Guo P, 2019, SCI ADV, V5, DOI 10.1126/sciadv.aav5010

Halling KK, 2004, BBA-BIOMEMBRANES, V1664, P161, DOI 10.1016/j.bbamem.2004.05.006

Hiruta Y, 2006, J CONTROL RELEASE, V113, P146, DOI 10.1016/j.jconrel.2006.04.016

Hong C, 2020, NANO-MICRO LETT, V12, DOI 10.1007/s40820-020-00472-8

Hong C, 2019, THERANOSTICS, V9, P4437, DOI 10.7150/thno.34953

Isakoff SJ, 2010, CANCER J, V16, P53, DOI 10.1097/PPO.0b013e3181d24ff7

Kaplan RN, 2005, NATURE, V438, P820, DOI 10.1038/nature04186

Kim Bo-Min, 2013, J Cancer Prev, V18, P177

Kim K, 2014, P NATL ACAD SCI USA, V111, P11774, DOI 10.1073/pnas.1410626111

Li YF, 2018, NANOMATERIALS-BASEL, V8, DOI 10.3390/nano8060361

Li YF, 2014, BMC CANCER, V14, DOI 10.1186/1471-2407-14-329

Liu S, 2017, J DRUG TARGET, V25, P541, DOI 10.1080/1061186X.2017.1298602

Liu Y, 2016, CANCER CELL, V30, P668, DOI 10.1016/j.ccell.2016.09.011

Mantovani A, 2009, NATURE, V457, P36, DOI 10.1038/457036b

Martinez-Usatorre A, 2020, CELL REP, V30, P599, DOI 10.1016/j.celrep.2020.01.027

Massague J, 2016, NATURE, V529, P298, DOI 10.1038/nature17038

Nguyen DX, 2009, NAT REV CANCER, V9, P274, DOI 10.1038/nrc2622

Ozturk-Atar K, 2019, J NANOSCI NANOTECHNO, V19, P3686, DOI 10.1166/jnn.2019.16764

Park CG, 2018, SCI TRANSL MED, V10, DOI 10.1126/scitranslmed.aar1916

Pauli G, 2019, PHARMACEUTICS, V11, DOI 10.3390/pharmaceutics11090465

Peinado H, 2017, NAT REV CANCER, V17, P302, DOI 10.1038/nrc.2017.6

Schust J, 2006, CHEM BIOL, V13, P1235, DOI 10.1016/j.chembiol.2006.09.018

Shah S, 2020, ADV DRUG DELIVER REV, V154, P102, DOI 10.1016/j.addr.2020.07.002

Shi JJ, 2011, ACCOUNTS CHEM RES, V44, P1123, DOI 10.1021/ar200054n

Song LJ, 2020, CANCER SCI, V111, P4242, DOI 10.1111/cas.14648

Sun MY, 2017, INT J MOL MED, V39, P507, DOI 10.3892/ijmm.2017.2857

Thompson AMG, 2015, SCI REP-UK, V5, DOI 10.1038/srep12804

Umansky V, 2013, CANCER MICROENVIRON, V6, P169, DOI 10.1007/s12307-012-0126-7

Volk LD, 2011, NEOPLASIA, V13, P327, DOI 10.1593/neo.101490

Wang XS, 2012, PRACT J CLIN MED, V9, P192

Yuan ZG, 2017, BIOMED PHARMACOTHER, V89, P227, DOI 10.1016/j.biopha.2017.02.038

Zhang YJ, 2019, ADV FUNCT MATER, V29, DOI 10.1002/adfm.201806620

Zhou H., 2006, ACTA ACAD MED ZUNYI, V29, P364

Zhu Y, 2021, J CONTROL RELEASE, V330, P641, DOI 10.1016/j.jconrel.2020.12.036

NR 45

TC 10

Z9 11

U1 35

U2 93

PU AMER ASSOC ADVANCEMENT SCIENCE

PI WASHINGTON

PA 1200 NEW YORK AVE, NW, WASHINGTON, DC 20005 USA

SN 2375-2548

J9 SCI ADV

JI Sci. Adv.

PD FEB

PY 2022

VL 8

IS 6

AR eabj1262

DI 10.1126/sciadv.abj1262

PG 19

WC Multidisciplinary Sciences

WE Science Citation Index Expanded (SCI-EXPANDED)

SC Science & Technology - Other Topics

GA ZC2VH

UT WOS:000757383500003

PM 35148178

OA Green Published

DA 2023-04-05

ER

PT J

AU Jin, YJ

Huynh, DTN

Myung, CS

Heo, KS

AF Jin, Yujin

Diem Thi Ngoc Huynh

Myung, Chang-Seon

Heo, Kyung-Sun

TI Ginsenoside Rh1 Prevents Migration and Invasion through Mitochondrial

ROS-Mediated Inhibition of STAT3/NF-kappa B Signaling in MDA-MB-231

Cells

SO INTERNATIONAL JOURNAL OF MOLECULAR SCIENCES

LA English

DT Article

DE metastasis; ginsenoside Rh1; mitochondrial ROS; STAT3; NF-kappa B;

triple-negative breast cancer cells

ID BREAST-CANCER CELLS; KAPPA-B ACTIVITY; STAT3; METASTASIS; ACTIVATION;

EXPRESSION; PATHWAY; GROWTH

AB Breast cancer (BC) a very common cancer in women worldwide. Triple negative breast cancer (TNBC) has been shown to have a poor prognosis with a high level of tumor metastatic spread. Here, the inhibitory effects of ginsenoside-Rh1 (Rh1) on BC metastasis, and its underlying signaling pathway in TNBC were investigated. Rh1-treated MDA-MB-231 cells were analyzed for metastasis using a wound healing assay, transwell migration and invasion assay, western blotting, and qRT-PCR. Rh1 treatment significantly inhibited BC metastasis by inhibiting the both protein and mRNA levels of MMP2, MMP9, and VEGF-A. Further, Rh1-mediated inhibitory effect on BC migration was associated with mitochondrial ROS generation. Rh1 treatment significantly eliminated STAT3 phosphorylation and NF-kappa B transactivation to downregulate metastatic factors, such as MMP2, MMP9, and VEGF-A. In addition, Mito-TEMPO treatment reversed Rh1 effects on the activation of STAT3, NF-kappa B, and their transcriptional targets. Rh1 further enhanced the inhibitory effects of STAT3 or NF-kappa B specific inhibitor, stattic or BAY 11-7082 on MMP2, MMP9, and VEGF-A expression, respectively. In summary, our results revealed the potent anticancer effect of Rh1 on TNBC migration and invasion through mtROS-mediated inhibition of STAT3 and NF-kappa B signaling.

C1 [Heo, Kyung-Sun] Chungnam Natl Univ, Coll Pharm, Daejeon 34134, South Korea.

Chungnam Natl Univ, Inst Drug Res & Dev, Daejeon 34134, South Korea.

C3 Chungnam National University; Chungnam National University

RP Heo, KS (通讯作者)，Chungnam Natl Univ, Coll Pharm, Daejeon 34134, South Korea.

EM 201850535@o.cnu.ac.kr; ngocdiemphar@gmail.com; cm8r@cnu.ac.kr;

kheo@cnu.ac.kr

OI Huynh, Diem Thi Ngoc/0000-0003-1822-5518

FU National Research Foundation of Korea (NRF) - Ministry of Science, ICT

and Future Planning [2017R1A4A1015860, 2019R1C1C100733112]

FX This research was supported by National Research Foundation of Korea

(NRF) funded by the Ministry of Science, ICT and Future Planning (and

2019R1C1C100733112).2017R1A4A1015860 and

CR Al-Mahmood S, 2018, DRUG DELIV TRANSL RE, V8, P1483, DOI 10.1007/s13346-018-0551-3

Alsamri H, 2019, FRONT ONCOL, V9, DOI 10.3389/fonc.2019.00743

Anastasiadi Z, 2017, UPDATES SURG, V69, P313, DOI 10.1007/s13304-017-0424-1

Banerjee K, 2016, INT J CANCER, V138, P2570, DOI 10.1002/ijc.29923

Boonrao M, 2010, ARCH PHARM RES, V33, P989, DOI 10.1007/s12272-010-0703-6

Chen W, 2018, J EXP CLIN CANC RES, V37, DOI 10.1186/s13046-018-0987-9

Chen XJ, 2016, EVID-BASED COMPL ALT, V2016, DOI 10.1155/2016/5738694

Dai XF, 2016, J CANCER, V7, P1281, DOI 10.7150/jca.13141

Esparza-Lopez J, 2019, BMC CANCER, V19, DOI 10.1186/s12885-019-5945-1

Huang Q, 2019, FRONT ONCOL, V9, DOI 10.3389/fonc.2019.00461

Huynh DTN, 2021, CANCERS, V13, DOI 10.3390/cancers13081892

Huynh DTN, 2020, INT J MOL SCI, V21, DOI 10.3390/ijms21186656

Jeon H, 2021, PHYTOMEDICINE, V85, DOI 10.1016/j.phymed.2021.153549

Jin FJ, 2019, CANCER BIOL MED, V16, P38, DOI 10.20892/j.issn.2095-3941.2018.0253

Jin S, 2019, MOLECULES, V24, DOI 10.3390/molecules24142618

Jin Y, 2020, ARCH PHARM RES, V43, P773, DOI [10.1007/s12272-020-01265-8, 10.1007/s12272-020-01255-w]

Jin Y, 2019, BMB REP, V52, P706, DOI 10.5483/BMBRep.2019.52.12.234

Jung JS, 2013, NEUROCHEM INT, V63, P80, DOI 10.1016/j.neuint.2013.05.002

Lee H, 2009, CANCER CELL, V15, P283, DOI 10.1016/j.ccr.2009.02.015

Lee KL, 2019, CANCERS, V11, DOI 10.3390/cancers11091334

Lee SY, 2015, EUR J PHARMACOL, V762, P333, DOI 10.1016/j.ejphar.2015.06.011

Li YL, 2019, FRONT PHARMACOL, V10, DOI 10.3389/fphar.2019.01195

Lin YZ, 2018, INT J MOL SCI, V19, DOI 10.3390/ijms19030729

Lyu X, 2019, ONCOL LETT, V18, P4160, DOI 10.3892/ol.2019.10742

Ma CB, 2019, MOL CELLS, V42, P628, DOI 10.14348/molcells.2019.0038

Martincuks A, 2020, FRONT ONCOL, V10, DOI 10.3389/fonc.2020.589601

Menke K, 2018, COMPLEMENT THER MED, V40, P158, DOI 10.1016/j.ctim.2018.03.005

Nguyen TL, 2021, ARCH PHARM RES, V44, P241, DOI 10.1007/s12272-020-01304-4

Peng B, 2019, PHARMACOL RES, V142, P1, DOI 10.1016/j.phrs.2019.02.003

Qin JJ, 2019, J EXP CLIN CANC RES, V38, DOI 10.1186/s13046-019-1206-z

Ryu D, 2020, ARCH PHARM RES, V43, P1297, DOI 10.1007/s12272-020-01298-z

Saleem MZ, 2020, ONCOTARGETS THER, V13, P435, DOI 10.2147/OTT.S228702

Shin KO, 2014, ARCH PHARM RES, V37, P1183, DOI 10.1007/s12272-014-0340-6

Shin SA, 2019, ARCH PHARM RES, V42, P658, DOI 10.1007/s12272-019-01169-2

Wang LH, 2019, J EXP CLIN CANC RES, V38, DOI 10.1186/s13046-019-1424-4

Yang DW, 2020, ARCH TOXICOL, V94, P3433, DOI 10.1007/s00204-020-02842-y

Yang YF, 2020, CANCER LETT, V485, P27, DOI 10.1016/j.canlet.2020.04.025

Yang YH, 2016, J CELL PHYSIOL, V231, P2570, DOI 10.1002/jcp.25349

Yeo IJ, 2019, ARCH PHARM RES, V42, P274, DOI 10.1007/s12272-018-1088-1

Yoon JH, 2012, EUR J PHARMACOL, V679, P24, DOI 10.1016/j.ejphar.2012.01.020

Zhang X, 2018, MOLECULES, V23, DOI 10.3390/molecules23061482

NR 41

TC 15

Z9 15

U1 3

U2 8

PU MDPI

PI BASEL

PA ST ALBAN-ANLAGE 66, CH-4052 BASEL, SWITZERLAND

EI 1422-0067

J9 INT J MOL SCI

JI Int. J. Mol. Sci.

PD OCT

PY 2021

VL 22

IS 19

AR 10458

DI 10.3390/ijms221910458

PG 16

WC Biochemistry & Molecular Biology; Chemistry, Multidisciplinary

WE Science Citation Index Expanded (SCI-EXPANDED)

SC Biochemistry & Molecular Biology; Chemistry

GA ZA6WZ

UT WOS:000756302900001

PM 34638797

OA gold, Green Published

DA 2023-04-05

ER

PT J

AU Park, JE

Kim, HW

Yun, SH

Kim, SJ

AF Park, Jae Eun

Kim, Hyeon Woo

Yun, Sung Hwan

Kim, Sun Jung

TI Ginsenoside Rh2 upregulates long noncoding RNA STXBP5-AS1 to sponge

microRNA-4425 in suppressing breast cancer cell proliferation

SO JOURNAL OF GINSENG RESEARCH

LA English

DT Article

DE Breast cancer; ceRNA; Ginsenoside Rh2; Long noncoding RNA; microRNA

ID APOPTOSIS; CERNA

AB Background: Ginsenoside Rh2, a major saponin derivative in ginseng extract, is recognized for its anti-cancer activities. Compared to coding genes, studies on long noncoding RNAs (lncRNAs) and microRNAs (miRNAs) that are regulated by Rh2 in cancer cells, especially on competitive endogenous RNA (ceRNA) are sparse.

Methods: LncRNAs whose promoter DNA methylation level was significantly altered by Rh2 were screened from methylation array data. The effect of STXBP5-AS1, miR-4425, and RNF217 on the proliferation and apoptosis of MCF-7 breast cancer cells was monitored in the presence of Rh2 after deregulating the corresponding gene. The ceRNA relationship between STXBP5-AS1 and miR-4425 was examined by measuring the luciferase activity of a recombinant luciferase/STXBP5-AS1 plasmid construct in the presence of mimic miR-4425.

Results: Inhibition of STXBP5-AS1 decreased apoptosis but stimulated growth of the MCF-7 cells, suggesting tumor-suppressive activity of the lncRNA. MiR-4425 was identified to have a binding site on STXBP5-AS1 and proven to be downregulated by STXBP5-AS1 as well as by Rh2. In contrast to STXBP5-AS1, miR-4425 showed pro-proliferation activity by inducing a decrease in apoptosis but increased growth of the MCF-7 cells. MiR-4425 decreased luciferase activity from the luciferase/STXBP5-AS1 construct by 26%. Screening the target genes of miR-4425 and Rh2 revealed that Rh2, STXBP5-AS1, and miR-4425 consistently regulated tumor suppressor RNF217 at both the RNA and protein level.

Conclusion: LncRNA STXBP5-AS1 is upregulated by Rh2 via promoter hypomethylation and acts as a ceRNA, sponging the oncogenic miR-4425. Therefore, Rh2 controls the STXBP5-AS1/miR-4425/RNF217 axis to suppress breast cancer cell growth. (C) 2021 The Korean Society of Ginseng. Publishing services by Elsevier B.V.

C1 [Park, Jae Eun; Kim, Hyeon Woo; Yun, Sung Hwan; Kim, Sun Jung] Dongguk Univ Seoul, Dept Life Sci, Goyang 10326, South Korea.

C3 Dongguk University

RP Kim, SJ (通讯作者)，Dongguk Univ Seoul, Dept Life Sci, Goyang 10326, South Korea.

EM sunjungk@dongguk.edu

RI kim, sun jung/T-1013-2019

FU Korean Society of Ginseng; Basic Science Research Program of the

National Research Foundation of Korea - Ministry of Education, Science

and Technology [NRF2016R1D1A1B01009235]

FX This work was supported by a grant from the Korean Society of Ginseng

(2018); and by the Basic Science Research Program

(NRF2016R1D1A1B01009235) of the National Research Foundation of Korea,

funded by the Ministry of Education, Science and Technology.

CR Cen DZ, 2019, ONCOTARGETS THER, V12, P1929, DOI 10.2147/OTT.S194463

Chen S, 2020, CLIN EPIGENETICS, V12, DOI 10.1186/s13148-020-00961-y

Chen WW, 2019, J ASIAN NAT PROD RES, V21, P742, DOI 10.1080/10286020.2018.1490273

Chen Y, 2018, J CELL BIOCHEM, V119, P6527, DOI 10.1002/jcb.26716

Chen Y, 2016, ONCOL REP, V36, P137, DOI 10.3892/or.2016.4774

Chen YY, 2019, ANTI-CANCER AGENT ME, V19, P1633, DOI 10.2174/1871520619666190704165205

Dong B, 2017, CELL MOL BIOL, V63, P1, DOI 10.14715/cmb/2017.63.8.1

Gao QR, 2018, CELL PROLIFERAT, V51, DOI 10.1111/cpr.12438

Ham J, 2019, J GINSENG RES, V43, P625, DOI 10.1016/j.jgr.2019.02.004

Huang JJ, 2016, ACTA BIOCH BIOPH SIN, V48, P750, DOI 10.1093/abbs/gmw049

Jeong D, 2019, AM J CHINESE MED, V47, P1643, DOI 10.1142/S0192415X19500848

Ji HW, 2020, CANCERS, V12, DOI 10.3390/cancers12092640

Jia WWG, 2004, CAN J PHYSIOL PHARM, V82, P431, DOI [10.1139/y04-049, 10.1139/Y04-049]

Jiang YS, 2010, INT J MOL MED, V26, P787, DOI 10.3892/ijmm_00000526

Karreth FA, 2013, CANCER DISCOV, V3, P1113, DOI 10.1158/2159-8290.CD-13-0202

Kim H, 2021, BIOMOLECULES, V11

Kim HW, 2020, OXID MED CELL LONGEV, V2020, DOI 10.1155/2020/9490567

Krause LMF, 2014, SCI REP-UK, V4, DOI 10.1038/srep06565

Lee H, 2018, J GINSENG RES, V42, P455, DOI 10.1016/j.jgr.2017.05.003

Li M, 2019, EXP MOL PATHOL, V108, P17, DOI 10.1016/j.yexmp.2019.03.004

Li X, 2020, EUR J MED CHEM, V203, DOI 10.1016/j.ejmech.2020.112627

Lu JJ, 2020, ARCH BIOCHEM BIOPHYS, V693, DOI 10.1016/j.abb.2020.108569

OTA T, 1987, CANCER RES, V47, P3863

Qi XL, 2015, J MED GENET, V52, P710, DOI 10.1136/jmedgenet-2015-103334

Shao SQ, 2019, BIOMED PHARMACOTHER, V117, DOI 10.1016/j.biopha.2019.109082

Volovat SR, 2020, FRONT ONCOL, V10, DOI 10.3389/fonc.2020.526850

Wang YS, 2017, SCI REP-UK, V7, DOI 10.1038/s41598-017-12572-4

Wang YC, 2018, EXP THER MED, V15, P4916, DOI 10.3892/etm.2018.6067

Wen Xu, 2015, Asian Pac J Cancer Prev, V16, P1105

Zhang HB, 2021, ONCOL REP, V45, DOI 10.3892/or.2021.7984

Zhang LN, 2019, J GENE MED, V21, DOI 10.1002/jgm.3074

Zhao Y, 2009, PLANTA MED, V75, P1124, DOI 10.1055/s-0029-1185477

Zheng X, 2018, CELL PHYSIOL BIOCHEM, V51, P1340, DOI 10.1159/000495552

NR 33

TC 7

Z9 9

U1 2

U2 8

PU KOREAN SOC GINSENG

PI SEOCHO-GU

PA 1807 SEOCHO WORLD OFFICETEL, 1355-3 SEOCHO-DONG, SEOCHO-GU, SEOUL

137-862, SOUTH KOREA

SN 1226-8453

EI 2093-4947

J9 J GINSENG RES

JI J. Ginseng Res.

PD NOV

PY 2021

VL 45

IS 6

BP 754

EP 762

DI 10.1016/j.jgr.2021.08.006

EA OCT 2021

PG 9

WC Plant Sciences; Chemistry, Medicinal; Integrative & Complementary

Medicine

WE Science Citation Index Expanded (SCI-EXPANDED)

SC Plant Sciences; Pharmacology & Pharmacy; Integrative & Complementary

Medicine

GA ZA9JH

UT WOS:000756471100014

PM 34764730

OA gold, Green Published

DA 2023-04-05

ER

PT J

AU Sekar, P

Ravitchandirane, R

Khanam, S

Muniraj, N

Cassinadane, AV

AF Sekar, Priyanka

Ravitchandirane, Raashmi

Khanam, Sofia

Muniraj, Nethaji

Cassinadane, Ananda Vayaravel

TI Novel molecules as the emerging trends in cancer treatment: an update

SO MEDICAL ONCOLOGY

LA English

DT Review

DE Novel molecules; Cancer; Combretastatin; Ginsenoside; Plitidepsin;

Eribulin; Mycobacterium bovis

ID CLOSTRIDIUM-NOVYI-NT; NF-KAPPA-B; CELL-CYCLE ARREST; HUMAN

LEUKEMIA-CELLS; KOREAN RED GINSENG; GROWTH IN-VITRO; BREAST-CANCER;

LUNG-CANCER; DEPENDENT APOPTOSIS; CALMETTE-GUERIN

AB As per World Health Organization cancer remains as a leading killer disease causing nearly 10 million deaths in 2020. Since the burden of cancer increases worldwide, warranting an urgent search for anti-cancer compounds from natural sources. Secondary metabolites from plants, marine organisms exhibit a novel chemical and structural diversity holding a great promise as therapeutics in cancer treatment. These natural metabolites target only the cancer cells and the normal healthy cells are left unharmed. In the emerging trends of cancer treatment, the natural bioactive compounds have long become a part of cancer chemotherapy. In this review, we have tried to compile about eight bioactive compounds from plant origin viz. combretastatin, ginsenoside, lycopene, quercetin, resveratrol, silymarin, sulforaphane and withaferin A, four marine-derived compounds viz. bryostatins, dolastatins, eribulin, plitidepsin and three microorganisms viz. Clostridium, Mycobacterium bovis and Streptococcus pyogenes with their well-established anticancer potential, mechanism of action and clinical establishments are presented.

C1 [Sekar, Priyanka; Ravitchandirane, Raashmi] Sri Venkateshwaraa Med Coll Hosp & Res Ctr, Pondicherry 605102, India.

[Khanam, Sofia] Calcutta Inst Pharmaceut Technol & Allied Hlth Sc, Howrah 711316, WB, India.

[Muniraj, Nethaji] Natl Childrens Hosp, Childrens Natl Res Inst, Ctr Canc Immunol Res, 111 Michigan Ave NW, Washington, DC 20010 USA.

[Cassinadane, Ananda Vayaravel] Sri Venkateshwaraa Coll Paramed Sci, Pondicherry 605102, India.

C3 Children's National Health System

RP Muniraj, N (通讯作者)，Natl Childrens Hosp, Childrens Natl Res Inst, Ctr Canc Immunol Res, 111 Michigan Ave NW, Washington, DC 20010 USA.; Cassinadane, AV (通讯作者)，Sri Venkateshwaraa Coll Paramed Sci, Pondicherry 605102, India.

EM nmuniraj@childrensnational.org; camvayaravel@gmail.com

RI CASSINADANE, ANANDA VAYARAVEL/ABE-3820-2021; KHANAM,

SOFIA/ABY-1397-2022; MUNIRAJ, NETHAJI/I-6017-2019

OI CASSINADANE, ANANDA VAYARAVEL/0000-0002-2103-8417; KHANAM,

SOFIA/0000-0002-5201-7387; MUNIRAJ, NETHAJI/0000-0002-3820-2148;

RAVITCHANDIRANE, RAASHMI/0000-0001-9267-8887

CR Agarwal R, 2006, ANTICANCER RES, V26, P4457

Agrawal N, 2004, P NATL ACAD SCI USA, V101, P15172, DOI 10.1073/pnas.0406242101

Aizawa K, 2016, INT J CANCER, V139, P1171, DOI 10.1002/ijc.30161

Al-Malki AL, 2012, TOXICOL IND HEALTH, V28, P542, DOI 10.1177/0748233711416948

Alonso-Alvarez S, 2017, DRUG DES DEV THER, V11, P253, DOI 10.2147/DDDT.S94165

Atwell LL, 2015, MOL NUTR FOOD RES, V59, P424, DOI 10.1002/mnfr.201400674

BAI R, 1990, BIOCHEM PHARMACOL, V39, P1941, DOI 10.1016/0006-2952(90)90613-P

Banerjee S, 2008, J NAT PROD, V71, P492, DOI 10.1021/np0705716

Baskar R, 2012, INT J MED SCI, V9, P193, DOI 10.7150/ijms.3635

Berretta M, 2020, INT J MOL SCI, V21, DOI 10.3390/ijms21082945

Bettegowda C, 2006, NAT BIOTECHNOL, V24, P1573, DOI 10.1038/nbt1256

Bevers RFM, 2004, BRIT J CANCER, V91, P607, DOI 10.1038/sj.bjc.6602026

Bhuvaneswari V., 2005, Current Medicinal Chemistry - Anti-Cancer Agents, V5, P627, DOI 10.2174/156801105774574667

Biberacher V, 2012, HAEMATOL-HEMATOL J, V97, P771, DOI 10.3324/haematol.2011.049155

Biscardi M, 2005, ANN ONCOL, V16, P1667, DOI 10.1093/annonc/mdi311

Bishayee A, 2010, PHARM RES-DORDR, V27, P1080, DOI 10.1007/s11095-010-0144-4

Biteau K, 2016, AM J CANCER RES, V6, P677

Boily G, 2009, ONCOGENE, V28, P2882, DOI 10.1038/onc.2009.147

Boly R, 2011, INT J ONCOL, V38, P833, DOI 10.3892/ijo.2010.890

Bricker GV, 2014, MOL NUTR FOOD RES, V58, P1991, DOI 10.1002/mnfr.201400104

Brock KE, 2012, BRIT J NUTR, V108, P1077, DOI 10.1017/S0007114511006489

Broggini M, 2003, LEUKEMIA, V17, P52, DOI 10.1038/sj.leu.2402788

Brown VA, 2010, CANCER RES, V70, P9003, DOI 10.1158/0008-5472.CAN-10-2364

Buettner R, 2002, CLIN CANCER RES, V8, P945

Bukhari SNA, 2017, BIOORG CHEM, V72, P130, DOI 10.1016/j.bioorg.2017.04.007

Burns J, 2002, J AGR FOOD CHEM, V50, P3337, DOI 10.1021/jf0112973

Camara M, 2013, STUDIES NATURAL PROD, V40, P383

Careri M, 2003, J AGR FOOD CHEM, V51, P5226, DOI 10.1021/jf034149g

Carter LG, 2014, ENDOCR-RELAT CANCER, V21, pR209, DOI 10.1530/ERC-13-0171

Catalgol B, 2012, FRONT PHARMACOL, V3, DOI 10.3389/fphar.2012.00141

Chae YC, 2018, BMB REP, V51, P319, DOI 10.5483/BMBRep.2018.51.7.112

Chambers CS, 2017, FOOD RES INT, V100, P339, DOI 10.1016/j.foodres.2017.07.017

Chang JH, 2017, BBA-MOL CELL RES, V1864, P1746, DOI 10.1016/j.bbamcr.2017.06.017

Chen H., 2020, EFOOD, V1, P226

Chen PN, 2006, J DENT RES, V85, P220, DOI 10.1177/154405910608500303

Chen SH, 2014, EVID-BASED COMPL ALT, V2014, DOI 10.1155/2014/168940

Choi CH, 2003, PLANTA MED, V69, P235, DOI 10.1055/s-2003-38483

Choi S, 2007, CARCINOGENESIS, V28, P151, DOI 10.1093/carcin/bgl144

Chu SC, 2004, MOL CARCINOGEN, V40, P143, DOI 10.1002/mc.20018

Chung MA, 2003, CANCER RES, V63, P1280

Cragg GM, 2005, J ETHNOPHARMACOL, V100, P72, DOI 10.1016/j.jep.2005.05.011

Cross D, 2006, CLIN MED RES, V4, P218, DOI 10.3121/cmr.4.3.218

Cuadrado A, 2003, J BIOL CHEM, V278, P241, DOI 10.1074/jbc.M201010200

Cui Y, 2008, AM J CLIN NUTR, V87, P1009, DOI 10.1093/ajcn/87.4.1009

D'Andrea G, 2015, FITOTERAPIA, V106, P256, DOI 10.1016/j.fitote.2015.09.018

Dang LH, 2001, P NATL ACAD SCI USA, V98, P15155, DOI 10.1073/pnas.251543698

Dangles O, 2000, J CHEM SOC PERK T 2, V6, P1215

Das TP, 2016, CELL DEATH DIS, V7, DOI 10.1038/cddis.2015.403

Davidson SK, 1999, BIOL BULL, V196, P273, DOI 10.2307/1542952

Deep G, 2007, INTEGR CANCER THER, V6, P130, DOI 10.1177/1534735407301441

Deli T, 2020, PATHOL ONCOL RES, V26, P63, DOI 10.1007/s12253-018-00569-x

Delmas D, 2020, MOLECULES, V25, DOI 10.3390/molecules25092009

Deng XH, 2013, EXP THER MED, V6, P1155, DOI 10.3892/etm.2013.1285

DEVI P U, 1992, Indian Journal of Experimental Biology, V30, P169

DeWeerdt S, 2013, NATURE, V504, pS4, DOI 10.1038/504S4a

Diaz LA, 2005, TOXICOL SCI, V88, P562, DOI 10.1093/toxsci/kfi316

Dillon BJ, 2004, CANCER-AM CANCER SOC, V100, P826, DOI 10.1002/cncr.20057

Donoghue M, 2012, CLIN CANCER RES, V18, P1496, DOI 10.1158/1078-0432.CCR-11-2149

Dutta R, 2019, INT J MOL SCI, V20, DOI 10.3390/ijms20215310

Dzubak P, 2006, BIOORGAN MED CHEM, V14, P3793, DOI 10.1016/j.bmc.2006.01.035

Fahey JW, 2001, PHYTOCHEMISTRY, V56, P5, DOI 10.1016/S0031-9422(00)00316-2

Feher J, 2012, CURR PHARM BIOTECHNO, V13, P210, DOI 10.2174/138920112798868818

Fiedler T, 2015, CANCER BIOL THER, V16, P1047, DOI 10.1080/15384047.2015.1026478

Fishbein AB, 2009, ARCH PHARM RES, V32, P505, DOI 10.1007/s12272-009-1405-9

Fisher B, 2008, CANCER RES, V68, P10007, DOI 10.1158/0008-5472.CAN-08-0186

Flahive E, 2012, ANTICANCER AGENTS FROM NATURAL PRODUCTS, 2ND EDITION, P263

Fox ME, 1996, GENE THER, V3, P173

FREI E, 1985, CANCER RES, V45, P6523

Garcia-Fernandez LF, 2002, ONCOGENE, V21, P7533, DOI 10.1038/sj.onc.1205972

Gelman AE, 2003, NAT MED, V9, P1465, DOI 10.1038/nm1203-1465

Geoerger B, 2012, EUR J CANCER, V48, P289, DOI 10.1016/j.ejca.2011.10.036

Ghosh K, 2016, PLOS ONE, V11, DOI 10.1371/journal.pone.0168488

Gong XM, 2016, MOL CANCER RES, V14, P966, DOI 10.1158/1541-7786.MCR-16-0075

Gonzalez M, 2019, MOLECULES, V24, DOI 10.3390/molecules24234319

Gonzalez-Santiago L, 2006, CELL DEATH DIFFER, V13, P1968, DOI 10.1038/sj.cdd.4401898

Granato M, 2017, J NUTR BIOCHEM, V41, P124, DOI 10.1016/j.jnutbio.2016.12.011

Gruber C, 2011, MOL CANCER THER, V10, P233, DOI 10.1158/1535-7163.MCT-10-0669

Guallar-Garrido S, 2020, IMMUNOTARGETS THER, V9, P1, DOI 10.2147/ITT.S202006

Gupta M., 2018, GMJ MED, V2, P45, DOI [10.29088/GMJM.2018.45, DOI 10.29088/GMJM.2018.45]

Hahm ER, 2019, MOL CARCINOGEN, V58, P2139, DOI 10.1002/mc.23104

Hahm ER, 2013, CANCER LETT, V334, P101, DOI 10.1016/j.canlet.2012.08.026

Hahm ER, 2011, PLOS ONE, V6, DOI 10.1371/journal.pone.0023354

Hahm ER, 2011, MOL CARCINOGEN, V50, P614, DOI 10.1002/mc.20760

Han JS, 2020, BIOMED PHARMACOTHER, V129, DOI 10.1016/j.biopha.2020.110393

Harikumar KB, 2010, INT J CANCER, V127, P257, DOI 10.1002/ijc.25041

He Y, 2006, VACCINE, V24, P2575, DOI 10.1016/j.vaccine.2005.12.030

Hosseinzade A, 2019, FRONT IMMUNOL, V10, DOI 10.3389/fimmu.2019.00051

Hsieh YS, 2007, CARCINOGENESIS, V28, P977, DOI 10.1093/carcin/bgl221

Huo Y, 2007, REGUL TOXICOL PHARM, V49, P63, DOI 10.1016/j.yrtph.2007.05.005

Hura N, 2018, ACS OMEGA, V3, P9754, DOI 10.1021/acsomega.8b00996

Ip BC, 2014, CANCER PREV RES, V7, P1219, DOI 10.1158/1940-6207.CAPR-14-0154

Ip BC, 2014, NUTRIENTS, V6, P124, DOI 10.3390/nu6010124

Iqbal J, 2017, ASIAN PAC J TROP BIO, V7, P1129, DOI 10.1016/j.apjtb.2017.10.016

Jang SH, 2012, FREE RADICAL BIO MED, V52, P607, DOI 10.1016/j.freeradbiomed.2011.11.010

Janku F, 2020, J IMMUNOTHER CANCER, V8, pA233, DOI 10.1136/jitc-2020-SITC2020.0383

Jeong JH, 2009, J CELL BIOCHEM, V106, P73, DOI 10.1002/jcb.21977

Jiang C, 2000, BIOCHEM BIOPH RES CO, V276, P371, DOI 10.1006/bbrc.2000.3474

Jin XX, 2019, CANCER MED-US, V8, P1246, DOI 10.1002/cam4.1993

Johary A., 2012, INT J NUTR PHARM NEU, V2, P167

Johnson TM, 2017, PHARMACOTHERAPY, V37, P988, DOI 10.1002/phar.1975

Jordan MA, 2005, MOL CANCER THER, V4, P1086, DOI 10.1158/1535-7163.MCT-04-0345

Jordan MA, 2004, NAT REV CANCER, V4, P253, DOI 10.1038/nrc1317

Jung JH, 2013, J GINSENG RES, V37, P167, DOI 10.5142/jgr.2013.37.167

Kalra N, 2008, LIFE SCI, V82, P348, DOI 10.1016/j.lfs.2007.11.006

Karatoprak GS, 2020, MOLECULES, V25, DOI 10.3390/molecules25112560

Kaufman-Szymczyk A, 2015, INT J MOL SCI, V16, P29732, DOI 10.3390/ijms161226195

KAVOUSSI LR, 1990, J CLIN INVEST, V85, P62, DOI 10.1172/JCI114434

Khalifa SAM, 2019, MAR DRUGS, V17, DOI 10.3390/md17090491

Khan F, 2016, NUTRIENTS, V8, DOI 10.3390/nu8090529

Kim DJ, 1997, CANCER LETT, V120, P15, DOI 10.1016/S0304-3835(97)00281-4

Kim Mi Jung, 2015, J Cancer Prev, V20, P92, DOI 10.15430/JCP.2015.20.2.92

Kim SM, 2010, EUR J PHARMACOL, V631, P1, DOI 10.1016/j.ejphar.2009.12.018

Kim SM, 2009, ARCH PHARM RES, V32, P755, DOI 10.1007/s12272-009-1515-4

Kiruthiga PV, 2007, BASIC CLIN PHARMACOL, V100, P414, DOI 10.1111/j.1742-7843.2007.00069.x

Ko JH, 2017, INT J MOL SCI, V18, DOI 10.3390/ijms18122589

Koduru S, 2010, MOL CANCER THER, V9, P202, DOI 10.1158/1535-7163.MCT-09-0771

Koya T, 2017, SCI REP-UK, V7, DOI 10.1038/srep42145

Kundu JK, 2008, CANCER LETT, V269, P243, DOI 10.1016/j.canlet.2008.03.057

Lah JJ, 2007, WORLD J GASTROENTERO, V13, P5299, DOI 10.3748/wjg.v13.i40.5299

Lee SO, 2007, BIOCHEM BIOPH RES CO, V354, P165, DOI 10.1016/j.bbrc.2006.12.181

Lee YT, 2018, EUR J PHARMACOL, V834, P188, DOI 10.1016/j.ejphar.2018.07.034

Li D, 2019, INT J MOL MED, V43, P630, DOI 10.3892/ijmm.2018.3969

Li DB, 2016, MOL MED REP, V13, P2708, DOI 10.3892/mmr.2016.4841

Li JL, 2021, NPJ VACCINES, V6, DOI 10.1038/s41541-020-00278-0

Li LH, 2007, J ASIAN NAT PROD RES, V9, P593, DOI 10.1080/10286020600882502

Li YY, 2018, CANCER PREV RES, V11, P451, DOI 10.1158/1940-6207.CAPR-17-0423

Li YY, 2010, MOL CANCER, V9, DOI 10.1186/1476-4598-9-274

Lian FZ, 2007, CARCINOGENESIS, V28, P1567, DOI 10.1093/carcin/bgm076

LIN CM, 1988, MOL PHARMACOL, V34, P200

Liu Y, 2017, APOPTOSIS, V22, P544, DOI 10.1007/s10495-016-1334-2

Losada A, 2014, CANCER RES, V74, DOI 10.1158/1538-7445.AM2014-5467

LOU Y, 1994, J PHARM PHARMACOL, V46, P863, DOI 10.1111/j.2042-7158.1994.tb05703.x

Luo H, 2019, CHIN MED-UK, V14, DOI 10.1186/s13020-019-0270-9

Luo Y, 2010, CLIN DEV IMMUNOL, DOI 10.1155/2010/357591

Maalik A, 2014, TROP J PHARM RES, V13, P1561, DOI 10.4314/tjpr.v13i9.26

MALMGREN RA, 1955, CANCER RES, V15, P473

Marks PA, 2009, J CELL BIOCHEM, V107, P600, DOI 10.1002/jcb.22185

Masuda H, 2018, MOL PHARMACEUT, V15, P5762, DOI 10.1021/acs.molpharmaceut.8b00919

Matusheski NV, 2001, J AGR FOOD CHEM, V49, P5743, DOI 10.1021/jf010809a

McCarthy Edward F, 2006, Iowa Orthop J, V26, P154

Meeran SM, 2012, PLOS ONE, V7, DOI 10.1371/journal.pone.0037748

Meeran SM, 2010, PLOS ONE, V5, DOI 10.1371/journal.pone.0011457

Meyer JP, 2002, POSTGRAD MED J, V78, P449, DOI 10.1136/pmj.78.922.449

Middleton E, 1998, ADV EXP MED BIOL, V439, P175

Mikstacka R, 2013, CELL MOL BIOL LETT, V18, P368, DOI 10.2478/s11658-013-0094-z

Mirahmadi M, 2020, BIOMED PHARMACOTHER, V129, DOI 10.1016/j.biopha.2020.110459

MOCHIZUKI M, 1995, BIOL PHARM BULL, V18, P1197, DOI 10.1248/bpb.18.1197

Mohan R, 2016, METHOD ENZYMOL, V568, P187, DOI 10.1016/bs.mie.2015.09.025

Morande PE, 2012, INVEST NEW DRUG, V30, P1830, DOI 10.1007/s10637-011-9740-3

Moreno DA, 2006, J PHARMACEUT BIOMED, V41, P1508, DOI 10.1016/j.jpba.2006.04.003

MOSE JR, 1964, CANCER RES, V24, P212

Moselhy J, 2017, NEOPLASIA, V19, P451, DOI 10.1016/j.neo.2017.04.005

Muniraj N, 2019, CARCINOGENESIS, V40, P1110, DOI 10.1093/carcin/bgz015

Munoz MJ, 2007, P 2007 AACR ANN M LO

Murthy SH., 2015, J VASC INTERV RADIOL, V26, P151

Mutter R, 2000, BIOORGAN MED CHEM, V8, P1841, DOI 10.1016/S0968-0896(00)00150-4

Myzak MC, 2006, CARCINOGENESIS, V27, P811, DOI 10.1093/carcin/bgi265

Naimi A, 2019, J CELL PHYSIOL, V234, P13233, DOI 10.1002/jcp.27995

Nakajima H, 2004, CANCER IMMUNOL IMMUN, V53, P617, DOI 10.1007/s00262-003-0498-0

Nandini D B, 2020, J Oral Maxillofac Pathol, V24, P405, DOI 10.4103/jomfp.JOMFP_126_19

Nobili S, 2009, PHARMACOL RES, V59, P365, DOI 10.1016/j.phrs.2009.01.017

Noguera-Ortega E, 2020, CANCERS, V12, DOI 10.3390/cancers12071802

NONOMURA S, 1963, Yakugaku Zasshi, V83, P988

Ocio EM, 2016, J CLIN ONCOL, V34, DOI 10.1200/JCO.2016.34.15_suppl.8006

Ohta N, 2010, ACTA OTO-LARYNGOL, V130, P1287, DOI 10.3109/00016489.2010.483480

Okajima E, 1997, JPN J CANCER RES, V88, P543, DOI 10.1111/j.1349-7006.1997.tb00417.x

Oliveira J, 2020, STREPTOCOCCUS PYOGEN

Olivieri C, 2016, PEDIATR NEONATOL, V57, P240, DOI 10.1016/j.pedneo.2013.06.011

PARKER RC, 1947, P SOC EXP BIOL MED, V66, P461

Philip, 1999, Expert Opin Investig Drugs, V8, P2189

Pinz S, 2014, PLOS ONE, V9, DOI 10.1371/journal.pone.0099391

Pitot HC, 1999, CLIN CANCER RES, V5, P525

Polyak K, 2009, TRENDS GENET, V25, P30, DOI 10.1016/j.tig.2008.10.012

Popat R, 2013, BRIT J HAEMATOL, V160, P714, DOI 10.1111/bjh.12154

Popiela Tadeusz, 2004, Gastric Cancer, V7, P240, DOI 10.1007/s10120-004-0299-y

Popovich DG., 2012, INT J BIOMED PHARM S, V6, P56

Prakash J, 2001, PHYTOTHER RES, V15, P240, DOI 10.1002/ptr.779

Pratheeshkumar P., 2017, PLOS ONE, V7

Primikyri A, 2014, ACS CHEM BIOL, V9, P2737, DOI 10.1021/cb500259e

Qi LW, 2011, NAT PROD REP, V28, P467, DOI 10.1039/c0np00057d

Qi WJ, 2021, BIOMED PHARMACOTHER, V138, DOI 10.1016/j.biopha.2021.111546

Quail DF, 2013, NAT MED, V19, P1423, DOI 10.1038/nm.3394

Ramasamy K, 2008, CANCER LETT, V269, P352, DOI 10.1016/j.canlet.2008.03.053

Ravichandra VD., 2018, ANTICANCER PLANTS PR, DOI [10.1007/978-981-10-8548-2_21, DOI 10.1007/978-981-10-8548-2_21]

Ray PS, 1999, FREE RADICAL BIO MED, V27, P160, DOI 10.1016/S0891-5849(99)00063-5

Rebuffini Elena, 2012, Dent Res J (Isfahan), V9, pS192, DOI 10.4103/1735-3327.109752

RENAUD S, 1992, LANCET, V339, P1523, DOI 10.1016/0140-6736(92)91277-F

Reyes-Farias M, 2019, INT J MOL SCI, V20, DOI 10.3390/ijms20133177

Rhee MY, 2011, J ALTERN COMPLEM MED, V17, P45, DOI 10.1089/acm.2010.0065

ROODI N, 1995, JNCI-J NATL CANCER I, V87, P446, DOI 10.1093/jnci/87.6.446

Roy RV, 2013, J NAT PROD, V76, P1909, DOI 10.1021/np400441f

Benitez MLR, 2019, APPL MICROBIOL BIOT, V103, P7903, DOI 10.1007/s00253-019-10057-0

Ryoma Y, 2004, ANTICANCER RES, V24, P3295

Sahin K., 2018, CHAPTER 5 LYCOPENE M

Samantha SK., 2017, J NATL CANC I, V109, P6

Schoffski P, 2009, MAR DRUGS, V7, P57, DOI 10.3390/md7010057

Schwartsmann G, 2001, LANCET ONCOL, V2, P221, DOI 10.1016/S1470-2045(00)00292-8

Setola E, 2017, EXPERT REV ANTICANC, V17, P717, DOI 10.1080/14737140.2017.1344098

Shan Y, 2011, CURR MED CHEM, V18, P523, DOI 10.2174/092986711794480221

Shankar S, 2007, FRONT BIOSCI-LANDMRK, V12, P4839, DOI 10.2741/2432

Shetty N, 2014, SOUTH ASIAN J CANCER, V3, P57, DOI 10.4103/2278-330X.126527

Siegel R, 2013, CA-CANCER J CLIN, V63, P11, DOI 10.3322/caac.21166

Sin S, 2012, INT J ONCOL, V41, P1669, DOI 10.3892/ijo.2012.1604

Singh AP, 2019, MED RES REV, V39, P1851, DOI 10.1002/med.21565

Singh RP, 2006, MOL CARCINOGEN, V45, P436, DOI 10.1002/mc.20223

Singh SV, 2005, J BIOL CHEM, V280, P19911, DOI 10.1074/jbc.M412443200

Sirerol JA, 2015, FREE RADICAL BIO MED, V85, P1, DOI 10.1016/j.freeradbiomed.2015.03.027

Smith AB, 2005, BIOORG MED CHEM LETT, V15, P3623, DOI 10.1016/j.bmcl.2005.05.068

Sodrul IMD, 2018, ONCOTARGET, V9, P2931, DOI 10.18632/oncotarget.23407

Spicka I, 2019, ANN HEMATOL, V98, P2139, DOI 10.1007/s00277-019-03739-2

Staedtke V, 2016, GENES DIS, V3, P144, DOI 10.1016/j.gendis.2016.01.003

Staedtke V, 2015, ONCOTARGET, V6, P5536, DOI 10.18632/oncotarget.3627

Stan SD, 2008, CANCER RES, V68, P7661, DOI 10.1158/0008-5472.CAN-08-1510

Stan SD, 2008, NUTR CANCER, V60, P51, DOI 10.1080/01635580802381477

Subbaraju GV, 2006, J NAT PROD, V69, P1790, DOI 10.1021/np060147p

Sun CX, 2016, J EXP CLIN CANC RES, V35, DOI 10.1186/s13046-015-0274-y

Sung HH, 2022, CLIN EXP HYPERTENS, V44, P146, DOI 10.1080/10641963.2021.2007943

Swami U, 2017, EXPERT OPIN INV DRUG, V26, P495, DOI 10.1080/13543784.2017.1292250

Synakiewicz A, 2014, EXPERT OPIN INV DRUG, V23, P1517, DOI 10.1517/13543784.2014.934808

Takaoka M., 1939, NIPPON KAGAKU KAISHI, V60, P1090, DOI [10.1246/nikkashi1921.60.1090, DOI 10.1246/NIKKASHI1921.60.1090]

Tang SM, 2020, BIOMED PHARMACOTHER, V121, DOI 10.1016/j.biopha.2019.109604

Taraboletti G, 2004, BRIT J CANCER, V90, P2418, DOI 10.1038/sj.bjc.6601864

Tome-Carneiro J, 2013, CURR PHARM DESIGN, V19, P6064, DOI 10.2174/13816128113199990407

TORREY JOHN C., 1927, JOUR CANCER RES, V11, P334

Trejo-Solis C, 2013, EVID-BASED COMPL ALT, V2013, DOI 10.1155/2013/705121

Tseng SH, 2004, CLIN CANCER RES, V10, P2190, DOI 10.1158/1078-0432.CCR-03-0105

Tyagi A, 2006, CARCINOGENESIS, V27, P2269, DOI 10.1093/carcin/bgl098

Ullah Mohammad Fahad, 2015, Medicines (Basel), V2, P141, DOI 10.3390/medicines2030141

Urdiales JL, 1996, CANCER LETT, V102, P31, DOI 10.1016/0304-3835(96)04151-1

Vaiopoulos AG, 2014, BBA-MOL BASIS DIS, V1842, P971, DOI 10.1016/j.bbadis.2014.02.006

Valkova V, 2020, J MICROB BIOTEC FOOD, V9, P836, DOI 10.15414/jmbfs.2020.9.4.836-843

Verdier-Pinard P, 2000, MOL PHARMACOL, V57, P180

Vrana JA, 1998, DIFFERENTIATION, V63, P33, DOI 10.1046/j.1432-0436.1998.6310033.x

Wadsworth TL, 1999, BIOCHEM PHARMACOL, V57, P941, DOI 10.1016/S0006-2952(99)00002-7

Wang DK, 2019, BIOMED RES INT, V2019, DOI 10.1155/2019/8407683

Wang FP, 2018, INT J PHARMACOL, V14, P369, DOI 10.3923/ijp.2018.369.376

Wang JF, 2019, BIOCHEM BIOPH RES CO, V512, P473, DOI 10.1016/j.bbrc.2019.03.014

Wang K, 2011, AUTOPHAGY, V7, P966, DOI 10.4161/auto.7.9.15863

Wang L, 2018, J CANCER RES THER, V14, pS1, DOI 10.4103/0973-1482.204841

Wang M, 2012, CHINESE J PHYSIOL, V55, P134, DOI 10.4077/CJP.2012.BAA085

Wang XJ, 2010, CANCER IMMUNOL IMMUN, V59, P1859, DOI 10.1007/s00262-010-0911-4

Wei Ming Q, 2008, Genet Vaccines Ther, V6, P8, DOI 10.1186/1479-0556-6-8

Wender PA, 1999, MED RES REV, V19, P388

Whisner CM, 2019, CURR NUTR REP, V8, P42, DOI 10.1007/s13668-019-0257-2

Widodo N, 2007, CLIN CANCER RES, V13, P2298, DOI 10.1158/1078-0432.CCR-06-0948

Widodo N, 2010, PLOS ONE, V5, DOI 10.1371/journal.pone.0013536

Wright J J, 1999, Oncology (Williston Park), V13, P68

Xia SX, 2018, BIOCHEM BIOPH RES CO, V503, P2363, DOI 10.1016/j.bbrc.2018.06.162

Xia T, 2017, MOL MED REP, V15, P3591, DOI 10.3892/mmr.2017.6459

Xia T, 2016, ONCOTARGET, V7, P27336, DOI 10.18632/oncotarget.8285

Xu CJ, 2005, ONCOGENE, V24, P4486, DOI 10.1038/sj.onc.1208656

Yang CM, 2011, MOL NUTR FOOD RES, V55, P606, DOI 10.1002/mnfr.201000308

Yang WS, 2006, MOL CANCER THER, V5, P1610, DOI 10.1158/1535-7163.MCT-05-0515

Yang WZ, 2014, PHYTOCHEMISTRY, V106, P7, DOI 10.1016/j.phytochem.2014.07.012

Yang Z, 2013, PLOS ONE, V8, DOI 10.1371/journal.pone.0075069

Yokosaka S, 2018, BIOORGAN MED CHEM, V26, P1643, DOI 10.1016/j.bmc.2018.02.011

Yu DL, 2017, ONCOTARGETS THER, V10, P4719, DOI 10.2147/OTT.S136840

Yuan ZG, 2017, BIOMED PHARMACOTHER, V89, P227, DOI 10.1016/j.biopha.2017.02.038

YUN TK, 1990, INT J EPIDEMIOL, V19, P871, DOI 10.1093/ije/19.4.871

Zeng HW, 2011, NUTR CANCER, V63, P248, DOI 10.1080/01635581.2011.523500

Zeng N, 2018, MOL MED REP, V17, P1077, DOI 10.3892/mmr.2017.7993

Zhang Y, 2007, VACCINE, V25, P6911, DOI 10.1016/j.vaccine.2007.07.034

Zheng YQ, 2015, EXPERT REV VACCINES, V14, P1255, DOI 10.1586/14760584.2015.1068124

Zhong X, 2006, TOXICOLOGY, V227, P211, DOI 10.1016/j.tox.2006.07.021

Zhou B, 2016, RADIOLOGY, V280, P630, DOI 10.1148/radiol.2016150719

Zi XL, 1998, CLIN CANCER RES, V4, P1055

Zong JB, 2009, ONCOL REP, V22, P953, DOI 10.3892/or_00000522

NR 267

TC 5

Z9 5

U1 5

U2 25

PU HUMANA PRESS INC

PI TOTOWA

PA 999 RIVERVIEW DRIVE SUITE 208, TOTOWA, NJ 07512 USA

SN 1357-0560

EI 1559-131X

J9 MED ONCOL

JI Med. Oncol.

PD FEB

PY 2022

VL 39

IS 2

AR 20

DI 10.1007/s12032-021-01615-6

PG 24

WC Oncology

WE Science Citation Index Expanded (SCI-EXPANDED)

SC Oncology

GA YA9TA

UT WOS:000738666100005

PM 34982273

DA 2023-04-05

ER

PT J

AU Hashemi, F

Zarrabi, A

Zabolian, A

Saleki, H

Farahani, MV

Sharifzadeh, SO

Ghahremaniyeh, Z

Bejandi, AK

Hushmandi, K

Ashrafizadeh, M

Khan, H

AF Hashemi, Farid

Zarrabi, Ali

Zabolian, Amirhossein

Saleki, Hossein

Farahani, Mahdi Vasheghani

Sharifzadeh, Seyed Omid

Ghahremaniyeh, Zahra

Bejandi, Atefe Kazemzade

Hushmandi, Kiavash

Ashrafizadeh, Milad

Khan, Haroon

TI Novel Strategy in Breast Cancer Therapy: Revealing The Bright Side of

Ginsenosides

SO CURRENT MOLECULAR PHARMACOLOGY

LA English

DT Review

DE Breast cancer; ginsenoside; apoptosis; cancer therapy; MicroRNA;

autophagy

ID EPITHELIAL-MESENCHYMAL TRANSITION; COLORECTAL-CANCER; LUNG-CANCER;

IN-VITRO; INHIBITS PROLIFERATION; MULTIDRUG-RESISTANCE; PROMOTER

METHYLATION; ANTITUMOR-ACTIVITY; CARCINOMA CELLS; GASTRIC-CANCER

AB Breast cancer is one of the leading causes of death worldwide. Breast cancer cells demonstrate uncontrolled proliferation and high metastatic capacity. They can obtain resistance to chemotherapy and radiotherapy. This has resulted in troublesome treatment of breast cancer. Nature as a rich source of plant derived-natural products with anti-tumor activity can be of interest in breast cancer therapy. Ginsenosides are triterpenoid saponins and considered as secondary metabolites exclusively found in Panax species. From immemorial times, ginsenosides have been applied in the treatment of various disorders such as diabetes, inflammatory diseases, neurological disorders, and particularly, cancer. In the present review, we highlight the anti-tumor activity of ginsenosides against breast cancer cells. Ginsenosides are able to induce apoptosis and cell cycle arrest. They interfere with breast cancer metastasis via inhibiting epithelial-to-mesenchymal transition, matrix metalloproteinase proteins and angiogenesis. Ginsenosides can promote the efficacy of chemotherapy via suppressing migration and proliferation. Molecular pathways such as phosphatidylinositol 3-kinase (PI3K)/protein kinase B (Akt), insulin-like growth factor-1, Wnt, microRNAs and long non-coding RNAs are affected by ginsenosides in suppressing breast cancer malignancy. Consequently, ginsenosides are versatile compounds in breast cancer therapy by suppressing the growth and invasion, as well as promoting their sensitivity to chemotherapy.

C1 [Hashemi, Farid] Univ Tehran, Fac Vet Med, Dept Comparat Biosci, Tehran, Iran.

[Zarrabi, Ali; Ashrafizadeh, Milad] Sabanci Univ, Nanotechnol Res & Applicat Ctr SUNUM, TR-34956 Istanbul, Turkey.

[Zabolian, Amirhossein; Saleki, Hossein; Farahani, Mahdi Vasheghani; Sharifzadeh, Seyed Omid; Ghahremaniyeh, Zahra; Bejandi, Atefe Kazemzade] Islamic Azad Univ, Tehran Med Sci, Young Researchers & Elite Club, Tehran, Iran.

[Hushmandi, Kiavash] Univ Tehran, Fac Vet Med, Dept Food Hyg & Qual Control, Div Epidemiol & Zoonoses, Tehran, Iran.

[Ashrafizadeh, Milad] Sabanci Univ, Fac Engn & Nat Sci, Univ Caddesi 27, TR-34956 Istanbul, Turkey.

[Khan, Haroon] Abdul Wali Khan Univ, Dept Pharm, Mardan 23200, Pakistan.

C3 University of Tehran; Sabanci University; Islamic Azad University;

University of Tehran; Sabanci University; Abdul Wali Khan University

RP Khan, H (通讯作者)，Abdul Wali Khan Univ, Dept Pharm, Mardan 23200, Pakistan.

EM hkdr2006@gmail.com

RI Khan, Haroon/AAY-1785-2020; Zarrabi, Ali/U-2602-2019

OI Khan, Haroon/0000-0002-1736-4404; Zarrabi, Ali/0000-0003-0391-1769

CR Abu Samaan TM, 2019, BIOMOLECULES, V9, DOI 10.3390/biom9120789

Adams RH, 2007, NAT REV MOL CELL BIO, V8, P464, DOI 10.1038/nrm2183

Aggarwal V, 2020, BIOMEDICINES, V8, DOI 10.3390/biomedicines8050103

Anand P, 2008, PHARM RES-DORDR, V25, P2097, DOI 10.1007/s11095-008-9661-9

Ashrafizadeh M, 2020, J CELL PHYSIOL, V235, P9241, DOI 10.1002/jcp.29819

Attia YM, 2020, CHEM-BIOL INTERACT, V315, DOI 10.1016/j.cbi.2019.108865

Balasubramanian R, 2019, BRIT J HOSP MED, V80, P720, DOI 10.12968/hmed.2019.80.12.720

Banik K, 2020, PHARMACOL RES, V153, DOI 10.1016/j.phrs.2020.104635

Bashmail HA, 2020, MOLECULES, V25, DOI 10.3390/molecules25020426

Bedada TL, 2020, BIOMED PHARMACOTHER, V129, DOI 10.1016/j.biopha.2020.110409

Borah A, 2020, NANOTECHNOLOGY, V31, DOI 10.1088/1361-6528/ab6d20

Boutas I, 2019, ARCH GYNECOL OBSTET, V300, P1113, DOI 10.1007/s00404-019-05292-9

Chen Dafu, 2003, Sichuan Da Xue Xue Bao Yi Xue Ban, V34, P546

Chen W, 2019, BIOCHEM BIOPH RES CO, V520, P263, DOI 10.1016/j.bbrc.2019.09.133

Chen XP, 2011, INT J CLIN ONCOL, V16, P519, DOI 10.1007/s10147-011-0222-6

Chen XJ, 2019, MOL BIOL REP, V46, P5323, DOI 10.1007/s11033-019-04988-0

Chen X, 2019, BIOMED PHARMACOTHER, V120, DOI 10.1016/j.biopha.2019.109487

Chene P, 2003, NAT REV CANCER, V3, P102, DOI 10.1038/nrc991

Cheng JT, 2019, CELLS-BASEL, V8, DOI 10.3390/cells8101178

Chi YY, 2019, THERANOSTICS, V9, P6840, DOI 10.7150/thno.36338

Choi HJ, 2019, EMBO REP, V20, DOI 10.15252/embr.201948058

Choi S, 2011, J CELL BIOCHEM, V112, P330, DOI 10.1002/jcb.22932

Choi S, 2009, PHARM RES-DORDR, V26, P2280, DOI 10.1007/s11095-009-9944-9

Christensen LP, 2009, ADV FOOD NUTR RES, V55, P1, DOI 10.1016/S1043-4526(08)00401-4

Cong ZY, 2020, PHARMACOLOGY, V105, P329, DOI 10.1159/000503821

Deng L., 2015, SELF RENEWAL PATHWAY, P155

Deng XQ, 2020, BIOCHEM PHARMACOL, V178, DOI 10.1016/j.bcp.2020.114038

Di Vinci A, 2005, INT J CANCER, V114, P414, DOI 10.1002/ijc.20771

Dong YN, 2019, INT J NANOMED, V14, P6971, DOI 10.2147/IJN.S210882

Dorward HS, 2016, J EXP CLIN CANC RES, V35, DOI 10.1186/s13046-016-0310-6

Duan ZG, 2018, BIOCHEM BIOPH RES CO, V499, P482, DOI 10.1016/j.bbrc.2018.03.174

Endo M, 1999, MICROVASC RES, V58, P89, DOI 10.1006/mvre.1999.2158

Esteller M, 2000, JNCI-J NATL CANCER I, V92, P564, DOI 10.1093/jnci/92.7.564

Esteva-Font C, 2014, FASEB J, V28, P1446, DOI 10.1096/fj.13-245621

Feng FF, 2019, ONCOL LETT, V18, P5428, DOI 10.3892/ol.2019.10895

Feng SL, 2020, J GINSENG RES, V44, P247, DOI 10.1016/j.jgr.2018.10.007

Mendes JMF, 2020, INT J MOL SCI, V21, DOI 10.3390/ijms21186487

Fox DB, 2020, NAT METAB, V2, P318, DOI 10.1038/s42255-020-0191-z

Fultang N, 2020, SCI REP-UK, V10, DOI 10.1038/s41598-020-58864-0

Fuzzati N, 2004, J CHROMATOGR B, V812, P119, DOI 10.1016/j.jchromb.2004.07.039

Gao H, 2020, BIOMED PHARMACOTHER, V124, DOI 10.1016/j.biopha.2020.109891

Gao QG, 2014, J STEROID BIOCHEM, V141, P104, DOI 10.1016/j.jsbmb.2014.01.014

Gao Y, 2020, EUR J PHARMACOL, V866, DOI 10.1016/j.ejphar.2019.172801

Garcia M, 2007, GLOBAL CANC FACTS FI, P1

Guan F, 2016, PLOS ONE, V11, DOI 10.1371/journal.pone.0146553

Guilbert A, 2009, AM J PHYSIOL-CELL PH, V297, pC493, DOI 10.1152/ajpcell.00624.2008

Guo Q, 2020, AGING-US, V12, P80, DOI 10.18632/aging.102598

Guo XJ, 2019, ARTIF CELL NANOMED B, V47, P2972, DOI 10.1080/21691401.2019.1640712

Ham J, 2019, J GINSENG RES, V43, P625, DOI 10.1016/j.jgr.2019.02.004

Ham J, 2018, AM J CHINESE MED, V46, P1333, DOI 10.1142/S0192415X18500702

Han JY, 2013, PLANT CELL PHYSIOL, V54, P2034, DOI 10.1093/pcp/pct141

Han JY, 2011, PLANT CELL PHYSIOL, V52, P2062, DOI 10.1093/pcp/pcr150

Han S, 2012, MOL CELLS, V33, P243, DOI 10.1007/s10059-012-2204-6

Hanahan D, 2011, CELL, V144, P646, DOI 10.1016/j.cell.2011.02.013

Haralampidis K, 2002, ADV BIOCHEM ENG BIOT, V75, P31

Heiden MGV, 2009, SCIENCE, V324, P1029, DOI 10.1126/science.1160809

Henamayee S, 2020, MOLECULES, V25, DOI 10.3390/molecules25102278

Hoeben A, 2004, PHARMACOL REV, V56, P549, DOI 10.1124/pr.56.4.3

Hong YN, 2019, TOXICOLOGY, V418, P22, DOI 10.1016/j.tox.2019.02.010

Hu ML, 2020, FOOD FUNCT, V11, P456, DOI [10.1039/c9fo02166c, 10.1039/C9FO02166C]

Huang L, 2020, EXP THER MED, V19, P400, DOI 10.3892/etm.2019.8186

Hwang ST, 2020, MOLECULES, V25, DOI 10.3390/molecules25061320

Iwakuma T, 2003, MOL CANCER RES, V1, P993

Jenner H, 2005, PLANTA, V220, P503, DOI 10.1007/s00425-004-1434-z

Jeong D, 2019, AM J CHINESE MED, V47, P1643, DOI 10.1142/S0192415X19500848

Jiang BH, 2008, CURR CANCER DRUG TAR, V8, P19, DOI 10.2174/156800908783497122

Jiang BH, 2013, CURR CANCER DRUG TAR, V13, P233, DOI 10.2174/1568009611313030001

Jiang J, 2007, CANCER RES, V67, P10929, DOI 10.1158/0008-5472.CAN-07-1121

Jiang N, 2020, INT J BIOCHEM CELL B, V120, DOI 10.1016/j.biocel.2019.105681

Jiang ZS, 2017, BIOMED PHARMACOTHER, V96, P378, DOI 10.1016/j.biopha.2017.09.129

Kang JH, 2011, PLANT FOOD HUM NUTR, V66, P298, DOI 10.1007/s11130-011-0242-4

Kashyap D, 2021, SEMIN CANCER BIOL, V69, P5, DOI 10.1016/j.semcancer.2019.08.014

Kasiri N, 2020, INFLAMMOPHARMACOLOGY, V28, P39, DOI 10.1007/s10787-019-00660-y

Khan MM, 2020, INT J NANOMED, V15, P2207, DOI 10.2147/IJN.S247893

Kim Bo-Min, 2014, J Cancer Prev, V19, P23

Kim Bo-Min, 2013, J Cancer Prev, V18, P177

Kim BJ, 2013, J GINSENG RES, V37, P201, DOI 10.5142/jgr.2013.37.201

Kim BJ, 2011, BASIC CLIN PHARMACOL, V109, P233, DOI 10.1111/j.1742-7843.2011.00706.x

Kim SJ, 2015, J GINSENG RES, V39, P125, DOI 10.1016/j.jgr.2014.09.003

Kim YJ, 2019, INT J NANOMED, V14, P8195, DOI 10.2147/IJN.S221328

Kim YJ, 2015, BIOTECHNOL ADV, V33, P717, DOI 10.1016/j.biotechadv.2015.03.001

Ko JH, 2019, PHYTOTHER RES, V33, P1934, DOI 10.1002/ptr.6389

Kong FH, 2020, INT J BIOCHEM CELL B, V122, DOI 10.1016/j.biocel.2020.105731

Koroth J, 2019, BMC COMPLEM ALTERN M, V19, DOI 10.1186/s12906-019-2685-3

Krstic M, 2020, LAB INVEST, V100, P400, DOI 10.1038/s41374-019-0326-6

Kutuk SG, 2019, SCI REP-UK, V9, DOI 10.1038/s41598-019-54284-x

Lai HN, 2020, SCI CHINA LIFE SCI, V63, P419, DOI 10.1007/s11427-019-9581-8

Lai KC, 2019, TOXICOL APPL PHARM, V382, DOI 10.1016/j.taap.2019.114734

Phi LTH, 2019, ONCOTARGETS THER, V12, P10885, DOI 10.2147/OTT.S219063

Lee H, 2018, J GINSENG RES, V42, P455, DOI 10.1016/j.jgr.2017.05.003

Lee H, 2019, DRUG METAB DISPOS, V47, P1372, DOI 10.1124/dmd.119.087965

Lee JH, 2020, J ADV RES, V26, P83, DOI [10.1016/j.jare.2020.07.004, 10.7851/Ksrp.2020.26.2.083]

Lee JH, 2020, BIOCHIMIE, V175, P58, DOI 10.1016/j.biochi.2020.05.006

Lee JH, 2019, PHARMACOL RES, V150, DOI 10.1016/j.phrs.2019.104504

Lee MH, 2004, PLANT CELL PHYSIOL, V45, P976, DOI 10.1093/pcp/pch126

Lee SY, 2020, EVID-BASED COMPL ALT, V2020, DOI 10.1155/2020/8980124

Li J, 2019, SCI REP-UK, V9, DOI 10.1038/s41598-019-54177-z

Li K, 2019, PATHOL RES PRACT, V215, DOI 10.1016/j.prp.2019.152575

Li K, 2020, PHARMACOL THERAPEUT, V207, DOI 10.1016/j.pharmthera.2019.107465

Li KK, 2019, BIOORG CHEM, V90, DOI 10.1016/j.bioorg.2019.103061

Li L, 2020, J CONTROL RELEASE, V317, P259, DOI 10.1016/j.jconrel.2019.11.032

Li L, 2014, ONCOL REP, V32, P1779, DOI 10.3892/or.2014.3422

Li MS, 2019, DRUG DELIV, V26, P481, DOI 10.1080/10717544.2019.1600077

Li XT, 2014, PHYTOTHER RES, V28, P1553, DOI 10.1002/ptr.5167

Liang L, 2020, ACTA PHARMACOL SIN B, V10, P1036, DOI 10.1016/j.apsb.2019.11.009

Liskova A, 2020, CANCERS, V12, DOI 10.3390/cancers12061498

Liu HH, 2019, PLOS ONE, V14, DOI 10.1371/journal.pone.0216759

Liu JZ, 2019, EXP CELL RES, V385, DOI 10.1016/j.yexcr.2019.111691

Liu L, 2020, PHYTOMEDICINE, V78, DOI 10.1016/j.phymed.2020.153312

Liu TG, 2018, EVID-BASED COMPL ALT, V2018, DOI 10.1155/2018/1580427

Liu WG, 2019, CANCER CELL INT, V19, DOI 10.1186/s12935-019-0961-4

Liu XY, 2019, PHOTOCHEM PHOTOBIOL, V95, P1412, DOI 10.1111/php.13116

Liu YN, 2020, NUTRIENTS, V12, DOI 10.3390/nu12010246

Liu YN, 2019, BIOCHEM PHARMACOL, V168, P285, DOI 10.1016/j.bcp.2019.07.008

Liu YN, 2018, FOOD FUNCT, V9, DOI 10.1039/c8fo01122b

Liu Y, 2020, ARTIF CELL NANOMED B, V48, P777, DOI 10.1080/21691401.2020.1748639

Loh CY, 2019, CELLS-BASEL, V8, DOI 10.3390/cells8101118

Lu JJ, 2020, CLIN EXP PHARMACOL P, V47, P1455, DOI 10.1111/1440-1681.13321

Lu Y, 2019, PHYTOMEDICINE, V64, DOI 10.1016/j.phymed.2019.153054

Luengo-Gil G, 2019, CELL ONCOL, V42, P627, DOI 10.1007/s13402-019-00450-2

Luo J, 2010, INT J BIOL SCI, V6, P784

Luo LH, 2020, MICROVASC RES, V129, DOI 10.1016/j.mvr.2019.103968

Luo M, 2020, J CELL BIOCHEM, V121, P2994, DOI 10.1002/jcb.29556

Lyu X, 2019, ONCOL LETT, V18, P4160, DOI 10.3892/ol.2019.10742

Man SL, 2020, FOOD FUNCT, V11, P6422, DOI [10.1039/C9FO01901D, 10.1039/c9fo01901d]

Mao XH, 2020, EVID-BASED COMPL ALT, V2020, DOI 10.1155/2020/6065124

Mao XY, 2019, ONCOTARGETS THER, V12, P9817, DOI 10.2147/OTT.S214133

Marsh T, 2020, AUTOPHAGY, V16, P1164, DOI 10.1080/15548627.2020.1753001

Moon YJ, 2008, PHARM RES-DORDR, V25, P2158, DOI 10.1007/s11095-008-9583-6

Nair MG, 2020, CANCER MED-US, V9, P5587, DOI 10.1002/cam4.3183

Nakai K, 2016, AM J CANCER RES, V6, P1609

Nakhjavani M, 2019, PHARMACEUTICALS-BASE, V12, DOI 10.3390/ph12030117

Nakhjavani Maryam, 2019, Medicines (Basel), V6, DOI 10.3390/medicines6010017

Nakopoulou L, 2003, BREAST CANCER RES TR, V77, P145, DOI 10.1023/A:1021371028777

Nanao-Hamai M, 2019, EUR J PHARMACOL, V859, DOI 10.1016/j.ejphar.2019.172546

Nazir SU, 2019, GENE, V711, DOI 10.1016/j.gene.2019.143952

Oh J, 2019, J GINSENG RES, V43, P421, DOI 10.1016/j.jgr.2018.05.004

Oh M, 1999, INT J ONCOL, V14, P869

Onel K, 2004, MOL CANCER RES, V2, P1

Oshimo Y, 2003, INT J ONCOL, V23, P1663

Ostendorf BN, 2020, DEV CELL, V52, P542, DOI 10.1016/j.devcel.2020.02.005

Otterbach F, 2010, BREAST CANCER RES TR, V120, P67, DOI 10.1007/s10549-009-0370-9

Pandya G, 2020, BBA-REV CANCER, V1874, DOI 10.1016/j.bbcan.2020.188423

Papadopoulos MC, 2008, PFLUG ARCH EUR J PHY, V456, P693, DOI 10.1007/s00424-007-0357-5

Patel G, 2020, NANOMED-NANOTECHNOL, V24, DOI 10.1016/j.nano.2019.102147

Peng B, 2019, PHARMACOL RES, V142, P1, DOI 10.1016/j.phrs.2019.02.003

Peng CW, 2019, J GASTROEN HEPATOL, V34, P1711, DOI 10.1111/jgh.14649

Perez-Lloret S, 2017, EXPERT OPIN INV DRUG, V26, P1163, DOI 10.1080/13543784.2017.1371133

Phillips DR, 2006, CURR OPIN PLANT BIOL, V9, P305, DOI 10.1016/j.pbi.2006.03.004

Piva M, 2014, EMBO MOL MED, V6, P66, DOI 10.1002/emmm.201303411

Pokharel YR, 2010, NUTR CANCER, V62, P252, DOI 10.1080/01635580903407171

Prieto-Vila M, 2020, MOLECULES, V25, DOI 10.3390/molecules25112576

Qiao JH, 2020, BIOCHEM BIOPH RES CO, V530, P402, DOI 10.1016/j.bbrc.2020.05.069

Qin CX, 2019, EUR REV MED PHARMACO, V23, P8440, DOI 10.26355/eurrev_201910_19156

Qin LH, 2019, J AGR FOOD CHEM, V67, P14074, DOI 10.1021/acs.jafc.9b05706

Rahman MM, 2019, CELLS-BASEL, V8, DOI 10.3390/cells8101214

Ramchandani S, 2020, MOLECULES, V25, DOI 10.3390/molecules25061287

Rayburn E, 2005, CURR CANCER DRUG TAR, V5, P27, DOI 10.2174/1568009053332636

Ren GX, 2018, MOLECULES, V23, DOI 10.3390/molecules23112908

Ren ZG, 2020, SMALL, V16, DOI 10.1002/smll.201905233

Rodrigues-Ferreira S, 2019, P NATL ACAD SCI USA, V116, P23691, DOI 10.1073/pnas.1910824116

Runnels LW, 2001, SCIENCE, V291, P1043, DOI 10.1126/science.1058519

Samec M, 2020, J CANCER RES CLIN, V146, P3137, DOI 10.1007/s00432-020-03424-2

Samec M, 2020, EPMA J, V11, P377, DOI 10.1007/s13167-020-00217-y

Schmitz C, 2003, CELL, V114, P191, DOI 10.1016/S0092-8674(03)00556-7

Schonorf T, 2004, CANCER LETT, V207, P215, DOI 10.1016/j.canlet.2003.10.028

Sehdev V, 2009, J ONCOL, V2009, DOI 10.1155/2009/121458

Shaheen M, 2011, BLOOD, V117, P6074, DOI 10.1182/blood-2011-01-313734

Shahzadi I, 2020, BIOMEDICINES, V8, DOI 10.3390/biomedicines8050126

Shi YH, 2020, FASEB J, V34, P208, DOI 10.1096/fj.201901537R

Shi ZH, 2012, J SURG ONCOL, V106, P267, DOI 10.1002/jso.22155

Slepicka PF, 2019, TRENDS MOL MED, V25, P866, DOI 10.1016/j.molmed.2019.06.003

Soes Signe, 2014, Oncoscience, V1, P367

Sun MY, 2019, ONCOL LETT, V17, P965, DOI 10.3892/ol.2018.9701

Sun MH, 2020, DRUG DELIV, V27, P632, DOI 10.1080/10717544.2020.1756985

Sun Y, 2019, MOLECULES, V24, DOI 10.3390/molecules24061131

Tahara T, 2010, INT J MOL MED, V25, P471, DOI 10.3892/ijmm_00000367

Tan GZ, 2019, EUR REV MED PHARMACO, V23, P9996, DOI 10.26355/eurrev_201911_19566

Tan QQ, 2020, ENVIRON TOXICOL, V35, P643, DOI 10.1002/tox.22899

Tang H, 2020, INT J PHARMACEUT, V573, DOI 10.1016/j.ijpharm.2019.118806

Tang TT, 2019, THERANOSTICS, V9, P7384, DOI 10.7150/thno.37892

Tang YC, 2018, INT J ONCOL, V52, P127, DOI 10.3892/ijo.2017.4183

Thimmappa R, 2014, ANNU REV PLANT BIOL, V65, P225, DOI 10.1146/annurev-arplant-050312-120229

Tian YZ, 2020, PHARMAZIE, V75, P147, DOI 10.1691/ph.2020.9931

Mai TT, 2012, CANCER LETT, V321, P144, DOI 10.1016/j.canlet.2012.01.045

Tyagi M, 2019, TOXICOL IN VITRO, V60, P125, DOI 10.1016/j.tiv.2019.05.004

Tyutyunyk-Massey L, 2020, SEMIN CANCER BIOL, V66, P155, DOI 10.1016/j.semcancer.2019.11.008

Verigos J, 2019, CANCERS, V11, DOI 10.3390/cancers11101585

Vogelstein B, 2000, NATURE, V408, P307, DOI 10.1038/35042675

Wang B, 2020, CLIN TRANSL ONCOL, V22, P1491, DOI 10.1007/s12094-019-02283-9

Wang JJ, 2018, CANCER LETT, V415, P73, DOI 10.1016/j.canlet.2017.11.037

Wang LH, 2019, J EXP CLIN CANC RES, V38, DOI 10.1186/s13046-019-1424-4

Wang MC, 2015, ONCOL LETT, V10, P583, DOI 10.3892/ol.2015.3361

Wang N, 2019, BIOMED PHARMACOTHER, V120, DOI 10.1016/j.biopha.2019.109519

Wang PP, 2020, PEERJ, V8, DOI 10.7717/peerj.9281

Wang PW, 2016, SCI REP-UK, V6, DOI 10.1038/srep33709

Wang W, 2012, PLOS ONE, V7, DOI 10.1371/journal.pone.0041586

Wang X, 2019, MOL CELL BIOCHEM, V462, P115, DOI 10.1007/s11010-019-03615-7

Wee ZN, 2015, NAT COMMUN, V6, DOI 10.1038/ncomms9746

Weyer-Czernilofsky U, 2020, MOL CANCER THER, V19, P1059, DOI 10.1158/1535-7163.MCT-19-0378

Williams CB, 2015, ADV CANCER RES, V127, P253, DOI 10.1016/bs.acr.2015.04.008

Wu Q, 2018, BIOCHEM PHARMACOL, V148, P64, DOI 10.1016/j.bcp.2017.12.004

Wu ZJ, 2019, CANCER CELL INT, V19, DOI 10.1186/s12935-019-1050-4

Xu ZM, 2019, J CELL BIOCHEM, V120, P18388, DOI 10.1002/jcb.29150

Xu ZX, 2020, ENVIRON TOXICOL, V35, P1179, DOI 10.1002/tox.22983

Yim NH, 2020, MOLECULES, V25, DOI 10.3390/molecules25092068

Yool AJ, 2007, CURR PHARM DESIGN, V13, P3212, DOI 10.2174/138161207782341349

Yool AJ, 2010, CLIN EXP PHARMACOL P, V37, P403, DOI 10.1111/j.1440-1681.2009.05244.x

Yu ZQ, 2019, VIRUSES-BASEL, V11, DOI 10.3390/v11111045

Yuan ZG, 2017, BIOMED PHARMACOTHER, V89, P227, DOI 10.1016/j.biopha.2017.02.038

Yun UJ, 2020, CANCERS, V12, DOI 10.3390/cancers12030605

Zare-Zardini H, 2018, SCI REP-UK, V8, DOI 10.1038/s41598-017-18938-y

Zhai K, 2020, BIOMOLECULES, V10, DOI 10.3390/biom10111469

Zhan JM, 2020, CARBOHYD POLYM, V230, DOI 10.1016/j.carbpol.2019.115576

Zhang EY, 2017, ONCOL REP, V38, P359, DOI 10.3892/or.2017.5652

Zhang GD, 2020, EXP THER MED, V19, P2913, DOI 10.3892/etm.2020.8543

Zhang JW, 2012, BRIT J PHARMACOL, V165, P120, DOI 10.1111/j.1476-5381.2011.01505.x

Zhang KQ, 2016, PHARM BIOL, V54, P561, DOI 10.3109/13880209.2015.1101142

Zhang QY, 2008, CANCER BIOTHER RADIO, V23, P647, DOI 10.1089/cbr.2008.0532

Zhang RW, 2000, CURR PHARM DESIGN, V6, P393, DOI 10.2174/1381612003400911

Zhang XM, 2020, ONCOTARGETS THER, V13, P5819, DOI 10.2147/OTT.S250766

Zhang Y, 2020, J AGR FOOD CHEM, V68, P4893, DOI 10.1021/acs.jafc.0c01473

Zhang Y, 2016, ASIAN PAC J TROP MED, V9, P178, DOI 10.1016/j.apjtm.2016.01.010

Zhao BY, 2020, LIFE SCI, V244, DOI 10.1016/j.lfs.2019.117179

Zhao L, 2019, ANATOL J CARDIOL, V22, P232, DOI 10.14744/AnatolJCardiol.2019.83710

Zhou B, 2012, TALANTA, V88, P345, DOI 10.1016/j.talanta.2011.10.051

Zhou P, 2019, J CELL MOL MED, V23, P7088, DOI 10.1111/jcmm.14611

Zou JF, 2020, J BIOCHEM MOL TOXIC, V34, DOI 10.1002/jbt.22480

Zou J, 2018, CELL BIOCHEM FUNCT, V36, P303, DOI 10.1002/cbf.3349

Zou MJ, 2018, ONCOL LETT, V15, P2889, DOI 10.3892/ol.2017.7654

NR 230

TC 5

Z9 6

U1 4

U2 14

PU BENTHAM SCIENCE PUBL LTD

PI SHARJAH

PA EXECUTIVE STE Y-2, PO BOX 7917, SAIF ZONE, 1200 BR SHARJAH, U ARAB

EMIRATES

SN 1874-4672

EI 1874-4702

J9 CURR MOL PHARMACOL

JI Curr. Molec. Pharmacol.

PY 2021

VL 14

IS 6

BP 1093

EP 1111

DI 10.2174/1874467214666210120153348

PG 19

WC Biochemistry & Molecular Biology; Pharmacology & Pharmacy

WE Science Citation Index Expanded (SCI-EXPANDED)

SC Biochemistry & Molecular Biology; Pharmacology & Pharmacy

GA XU3QP

UT WOS:000734184200017

PM 33494691

DA 2023-04-05

ER

PT J

AU Li, Y

Wang, PP

Zou, ZL

Pan, Q

Li, XY

Liang, ZE

Li, LY

Lin, YB

Peng, XY

Zhang, RH

Tian, HQ

Han, L

AF Li, Yan

Wang, Panpan

Zou, Zhuoling

Pan, Qi

Li, Xiaoyun

Liang, Zien

Li, Lingyu

Lin, Yingbing

Peng, Xueyu

Zhang, Ronghua

Tian, Huaqin

Han, Li

TI Ginsenoside (20S)-protopanaxatriol induces non-protective autophagy and

apoptosis by inhibiting Akt/mTOR signaling pathway in triple-negative

breast cancer cells

SO BIOCHEMICAL AND BIOPHYSICAL RESEARCH COMMUNICATIONS

LA English

DT Article

DE Triple negative breast cancer (TNBC); (20S)-Protopanaxatriol; Autophagy;

Apoptosis; Akt; mTOR signaling pathway

ID 20(S)-PROTOPANAXATRIOL; BINDING

AB Triple-negative breast cancer (TNBC) lacks a recognized therapeutic molecular target and has an unfavorable prognosis. (20S)-Protopanaxatriol (g-PPT, PPT) is an active metabolite extracted from ginseng. Accumulating evidence suggests that it has good anti-cancer activity in vivo and in vitro. In this study, we aimed to elucidate the anti-tumor effects of PPT in TNBC cells and tumor-bearing mice, as well as the relevant molecular mechanisms of autophagy and apoptosis. In vitro, we have found that PPT is capable of inducing non-protective autophagy and apoptosis, thus exerting some anti-proliferative and anti migration activity in TNBC cells. And in vivo, the therapeutic effects of PPT were evaluated by xenograft mouse models. The potential binding mode of PPT and Akt was predicted by molecular docking. Our findings indicated that PPT treatment induced non-protective autophagy in TNBC cells by inhibiting the Akt/mTOR signaling pathway. Therefore, PPT may be a potential treatment for TNBC in the future. (c) 2021 The Authors. Published by Elsevier Inc. This is an open access article under the CC BY-NC-ND license (http://creativecommons.org/licenses/by-nc-nd/4.0/).

C1 [Li, Yan; Zou, Zhuoling; Pan, Qi; Liang, Zien; Lin, Yingbing; Peng, Xueyu] Jinan Univ, Coll Tradit Chinese Med, Guangzhou 510630, Guangdong, Peoples R China.

[Wang, Panpan; Han, Li] Jinan Univ, Affiliated Hosp 1, Guangzhou 510630, Guangdong, Peoples R China.

[Wang, Panpan; Li, Xiaoyun; Li, Lingyu; Zhang, Ronghua] Jinan Univ, Guangdong Prov Key Lab Tradit Chinese Med Informa, Guangzhou 510630, Guangdong, Peoples R China.

[Li, Xiaoyun; Zhang, Ronghua] Jinan Univ, Coll Pharm, Guangzhou 510630, Guangdong, Peoples R China.

[Li, Lingyu] Jinan Univ, Canc Res Inst, Guangzhou 510630, Guangdong, Peoples R China.

[Tian, Huaqin] Foshan Hosp Tradit Chinese Med, Foshan 528000, Guangdong, Peoples R China.

C3 Jinan University; Jinan University; Jinan University; Jinan University;

Jinan University

RP Han, L (通讯作者)，Jinan Univ, Affiliated Hosp 1, Guangzhou 510630, Guangdong, Peoples R China.; Zhang, RH (通讯作者)，Jinan Univ, Guangdong Prov Key Lab Tradit Chinese Med Informa, Guangzhou 510630, Guangdong, Peoples R China.; Tian, HQ (通讯作者)，Foshan Hosp Tradit Chinese Med, Foshan 528000, Guangdong, Peoples R China.

EM tzrh@jnu.edu.cn; 13929969262@139.com; hanli@jnu.edu.cn

FU Guangdong Provincial Key Laboratory of Traditional Chinese Medicine

Informatization [2021B1212040007]; National Natural Science Foundation

of China [81603342]; National Key R&D Program of China [2018YFC2002500];

Administration of Traditional Chinese Medicine of Guangdong Province

[20171074]

FX Our work was supported by Guangdong Provincial Key Laboratory of

Traditional Chinese Medicine Informatization (2021B1212040007), the

National Natural Science Foundation of China (81603342), National Key

R&D Program of China 2018YFC2002500, Foshan "Summit Plan" of building

high-level hospitals, and the Administration of Traditional Chinese

Medicine of Guangdong Province (No. 20171074).

CR Basho RK, 2017, JAMA ONCOL, V3, P509, DOI 10.1001/jamaoncol.2016.5281

Chen GT, 2015, BIOTECHNOL LETT, V37, P2005, DOI 10.1007/s10529-015-1877-2

Dey N, 2017, PHARMACOL THERAPEUT, V175, P91, DOI 10.1016/j.pharmthera.2017.02.037

Espert L, 2006, J CLIN INVEST, V116, P2161, DOI 10.1172/JCI26185

Galluzzi L, 2015, EMBO J, V34, P856, DOI 10.15252/embj.201490784

Garrido-Castro AC, 2019, CANCER DISCOV, V9, P176, DOI 10.1158/2159-8290.CD-18-1177

Harbeck N, 2017, LANCET, V389, P1134, DOI [10.1016/s0140-6736(16)31891-8, 10.1016/S0140-6736(16)31891-8]

Huang Q.F., 2019, THESIS HUAZHONG U SC

Huang QF, 2019, J EXP CLIN CANC RES, V38, DOI 10.1186/s13046-019-1120-4

Janin M, 2020, CANCER DISCOV, V10, P1258, DOI 10.1158/2159-8290.CD-20-0947

Jiang YZ, 2019, CANCER CELL, V35, P428, DOI 10.1016/j.ccell.2019.02.001

Kabeya Y, 2000, EMBO J, V19, P5720, DOI 10.1093/emboj/19.21.5720

Kim C, 2018, CELL, V173, P879, DOI 10.1016/j.cell.2018.03.041

Klionsky DJ, 2016, AUTOPHAGY, V12, P1, DOI 10.1080/15548627.2015.1100356

Kwak JH, 2014, BIOORG MED CHEM LETT, V24, P5409, DOI 10.1016/j.bmcl.2014.10.041

Levy JMM, 2017, NAT REV CANCER, V17, P528, DOI 10.1038/nrc.2017.53

Maiuri MC, 2007, NAT REV MOL CELL BIO, V8, P741, DOI 10.1038/nrm2239

Massihnia D, 2016, ONCOTARGET, V7, P60712, DOI 10.18632/oncotarget.10858

Pascual J, 2019, ANN ONCOL, V30, P1051, DOI 10.1093/annonc/mdz133

Salehi S, 2021, NATURE, V595, P585, DOI 10.1038/s41586-021-03648-3

Saw CLL, 2012, CHEM RES TOXICOL, V25, P1574, DOI 10.1021/tx2005025

Shu Ting, 2016, Zhongguo Shengwu Huaxue yu Fenzi Shengwu Xuebao, V32, P1192, DOI 10.13865/j.cnki.cjbmb.2016.11.03

Terrasse V., 2020, IARC

Thorburn A, 2014, MOL PHARMACOL, V85, P830, DOI 10.1124/mol.114.091850

Waks AG, 2019, JAMA-J AM MED ASSOC, V321, P288, DOI 10.1001/jama.2018.19323

Wang Q.G, 2019, THESIS HUAZHONG U SC

Wang ZH, 2020, ACTA PHARMACOL SIN B, V10, P1020, DOI 10.1016/j.apsb.2020.01.017

Yin L, 2020, BREAST CANCER RES, V22, DOI 10.1186/s13058-020-01296-5

Zhen YQ, 2020, THERANOSTICS, V10, P8080, DOI 10.7150/thno.43473

Zheng XY, 2020, MOL CANCER, V19, DOI 10.1186/s12943-020-01183-9

NR 30

TC 3

Z9 4

U1 4

U2 22

PU ACADEMIC PRESS INC ELSEVIER SCIENCE

PI SAN DIEGO

PA 525 B ST, STE 1900, SAN DIEGO, CA 92101-4495 USA

SN 0006-291X

EI 1090-2104

J9 BIOCHEM BIOPH RES CO

JI Biochem. Biophys. Res. Commun.

PD DEC 17

PY 2021

VL 583

BP 184

EP 191

DI 10.1016/j.bbrc.2021.10.067

EA NOV 2021

PG 8

WC Biochemistry & Molecular Biology; Biophysics

WE Science Citation Index Expanded (SCI-EXPANDED)

SC Biochemistry & Molecular Biology; Biophysics

GA XO1WI

UT WOS:000729982900005

PM 34749235

OA hybrid

DA 2023-04-05

ER

PT J

AU Song, M

Cui, YL

Wang, Q

Zhang, XL

Zhang, J

Liu, ML

Li, YF

AF Song, Miao

Cui, Yilong

Wang, Qi

Zhang, Xuliang

Zhang, Jian

Liu, Menglin

Li, Yanfei

TI Ginsenoside Rg3 Alleviates Aluminum Chloride-Induced Bone Impairment in

Rats by Activating the TGF-beta 1/Smad Signaling Pathway

SO JOURNAL OF AGRICULTURAL AND FOOD CHEMISTRY

LA English

DT Article

DE aluminum; ginsenosides Rg3; bone impairment; Rat; MC3T3-E1; TGF-beta

1/Smad3 signaling pathway

ID BREAST-CANCER; DIFFERENTIATION; EXPRESSION; INDUCTION; APOPTOSIS;

BEHAVIOR; CELLS

AB Aluminum (Al)-induced bone formation and metabolism disorder through inhibition of the TGF-beta 1/Smad signaling pathway is one of the important mechanisms of bone impairment. Ginsenoside Rg3 (Rg3), a specific biological effector molecule, can provide protection to bones. Previously, we demonstrated that Rg3 can reverse aluminum chloride (AlCl3)-induced oxidative stress and metabolic disorder of bones; however, whether the TGF-beta 1/Smad signaling pathway is involved in it remains unclear. First, we found that Rg3 attenuated AI-induced bone impairment in vivo and in vitro by relieving structural damage to the femur, increasing MC3T3-E1 cell activity, differentiation, mineralization, inhibition of cell apoptosis, and upregulating the extracellular matrix (ECM) synthesis and the expression of TGF-beta 1/Smad signaling pathway key factors. Subsequently, in the signal pathway intervention experiment, the protective effect of Rg3 on bone impairment induced by Al was weakened; these results indicate that activating the TGF-beta 1/Smad signaling pathway is one of the mechanisms of Rg3-attenuated AI-induced bone impairment.

C1 [Song, Miao; Cui, Yilong; Wang, Qi; Zhang, Xuliang; Zhang, Jian; Liu, Menglin; Li, Yanfei] Northeast Agr Univ, Coll Vet Med, Dept Heilongjiang Common Anim Dis Prevent & Treat, Key Lab Prov Educ, Harbin 150030, Peoples R China.

C3 Northeast Agricultural University - China

RP Li, YF (通讯作者)，Northeast Agr Univ, Coll Vet Med, Dept Heilongjiang Common Anim Dis Prevent & Treat, Key Lab Prov Educ, Harbin 150030, Peoples R China.

EM liyanfei@neau.edu.cn

RI Cui, Yilong/GLR-8473-2022

OI Cui, Yilong/0000-0001-8852-7461

FU National Natural Science Foundation of China [31872530]

FX This study was supported by a grant from the National Natural Science

Foundation of China (No. 31872530).

CR Borrirukwanit K, 2007, MATRIX BIOL, V26, P291, DOI 10.1016/j.matbio.2006.10.014

Cao Z, 2020, ENVIRON POLLUT, V264, DOI 10.1016/j.envpol.2020.114748

Chauhan DK, 2021, CRIT REV BIOTECHNOL, V41, P715, DOI 10.1080/07388551.2021.1874282

Cho M, 2019, J GINSENG RES, V43, P49, DOI 10.1016/j.jgr.2017.08.003

Choi RJ, 2021, FRONT PHARMACOL, V12, DOI 10.3389/fphar.2021.618773

Cui QB, 2011, EUR J APPL PHYSIOL, V111, P1457, DOI 10.1007/s00421-010-1764-4

Farias JS, 2020, ARCH BIOCHEM BIOPHYS, V684, DOI 10.1016/j.abb.2020.108306

Fathima SN., 2019, BIOMED PHARMACOL J, V12, P267, DOI DOI 10.13005/bpj/1637

Gu TT, 2019, EUR J PHARMACOL, V842, P70, DOI 10.1016/j.ejphar.2018.10.008

Hellstrom HO, 2005, OSTEOPOROSIS INT, V16, P1982, DOI 10.1007/s00198-005-1981-6

Hethey C, 2021, ARCH TOXICOL, V95, P2977, DOI 10.1007/s00204-021-03107-y

Hong T, 2020, STEM CELL RES THER, V11, DOI 10.1186/s13287-020-01974-3

Im W, 2012, J GINSENG RES, V36, P78, DOI 10.5142/jgr.2012.36.1.78

Ioachim E, 2002, EUR J CANCER, V38, P2362, DOI 10.1016/S0959-8049(02)00210-1

Justin-Thenmozhi A, 2018, NEUROTOX RES, V34, P463, DOI 10.1007/s12640-018-9904-4

Lee H, 2019, J GINSENG RES, V43, P431, DOI 10.1016/j.jgr.2018.07.003

Mailloux RJ, 2011, EXP CELL RES, V317, P2231, DOI 10.1016/j.yexcr.2011.07.009

Matsunobu T, 2009, DEV BIOL, V332, P325, DOI 10.1016/j.ydbio.2009.06.002

Carbonara CEM, 2020, J BRAS NEFROL, V42, P138, DOI [10.1590/2175-8239-JBN-2019-0045, 10.1590/2175-8239-jbn-2019-0045]

Nie XJ, 2020, REGEN BIOMATER, V7, P53, DOI 10.1093/rb/rbz033

Peng L, 2020, J ETHNOPHARMACOL, V251, DOI 10.1016/j.jep.2019.112503

Safadi FF, 2003, J CELL PHYSIOL, V196, P51, DOI 10.1002/jcp.10319

Sathishkumar N, 2012, J ENZYM INHIB MED CH, V27, P685, DOI 10.3109/14756366.2011.608663

Song M, 2020, BIOL TRACE ELEM RES, V198, P557, DOI 10.1007/s12011-020-02089-9

Sun XD, 2017, CHEMOSPHERE, V176, P1, DOI 10.1016/j.chemosphere.2017.02.086

Sun XD, 2016, TOXICOLOGY, V371, P49, DOI 10.1016/j.tox.2016.10.002

Sun XD, 2016, CHEM-BIOL INTERACT, V244, P9, DOI 10.1016/j.cbi.2015.11.027

Taubenberger AV, 2010, BIOMATERIALS, V31, P2827, DOI 10.1016/j.biomaterials.2009.12.051

Wang Jun-ping, 2006, J Zhejiang Univ Sci B, V7, P769, DOI 10.1631/jzus.2006.B0769

Wang P., 2021, HORTIC RES-JAPAN, V8

Won HJ, 2019, J GINSENG RES, V43, P354

Yan H, 2016, BRAIN RES, V1630, P241, DOI 10.1016/j.brainres.2015.11.004

Yang X, 2018, FOOD CHEM TOXICOL, V116, P307, DOI 10.1016/j.fct.2018.04.057

Yang Y, 2017, J ETHNOPHARMACOL, V201, P91, DOI 10.1016/j.jep.2017.02.033

Yao XL, 2021, MOL THER-NUCL ACIDS, V24, P223, DOI 10.1016/j.omtn.2021.02.030

Yap L, 2019, TRENDS CELL BIOL, V29, P987, DOI 10.1016/j.tcb.2019.10.001

Yoshimoto T, 2015, CYTOKINE, V75, P165, DOI 10.1016/j.cyto.2015.03.011

Zhang MN, 2019, VET MICROBIOL, V237, DOI 10.1016/j.vetmic.2019.108420

Zhang X, 2010, J CELL PHYSIOL, V224, P691, DOI 10.1002/jcp.22173

Zhang XN, 2016, CHEM-BIOL INTERACT, V256, P188, DOI 10.1016/j.cbi.2016.07.003

Zhao MD, 2021, POULTRY SCI, V100, P527, DOI 10.1016/j.psj.2020.10.053

Zheng XM, 2019, BIOORGAN MED CHEM, V27, P4211, DOI 10.1016/j.bmc.2019.07.054

NR 42

TC 3

Z9 3

U1 5

U2 10

PU AMER CHEMICAL SOC

PI WASHINGTON

PA 1155 16TH ST, NW, WASHINGTON, DC 20036 USA

SN 0021-8561

EI 1520-5118

J9 J AGR FOOD CHEM

JI J. Agric. Food Chem.

PD NOV 3

PY 2021

VL 69

IS 43

BP 12634

EP 12644

DI 10.1021/acs.jafc.1c04695

EA OCT 2021

PG 11

WC Agriculture, Multidisciplinary; Chemistry, Applied; Food Science &

Technology

WE Science Citation Index Expanded (SCI-EXPANDED)

SC Agriculture; Chemistry; Food Science & Technology

GA WT4PL

UT WOS:000715847700005

PM 34694773

DA 2023-04-05

ER

PT J

AU Liu, SC

Huang, JH

Gao, FC

Yin, ZP

Zhang, RK

AF Liu, Shengcui

Huang, Junhua

Gao, Fucun

Yin, Zhiping

Zhang, Ruikui

TI Ginsenoside RG1 augments doxorubicin-induced apoptotic cell death in

MDA-MB-231 breast cancer cell lines

SO JOURNAL OF BIOCHEMICAL AND MOLECULAR TOXICOLOGY

LA English

DT Article

DE apoptosis; breast cancer; chemosensitization; doxorubicin; ginsenoside

Rg1

ID INHIBITION; RESISTANCE

AB This study determined the chemosensitizing potential of ginsenoside Rg1 in triple-negative MDA-MB-231 breast cancer cell lines. Ginsenoside Rg1 (10 mu M) treated breast cancer cells were exposed to 8 nM of doxorubicin, and the chemosensitizing potential was measured by cell-based assays. Ginsenoside Rg1 (10 mu M) treatment lowered the doxorubicin IC50 value to 0.01 nM. Furthermore, the ginsenoside pretreatment augments doxorubicin-mediated reactive oxygen species (ROS) generation and subsequent alterations of mitochondrial membrane potential in MDA-MB-231 cell lines. The alkaline comet assay results illustrated an increased % tail DNA during ginsenoside Rg1 plus doxorubicin treatment than doxorubicin alone treatment. In addition, the number of apoptotic cells was also increased in ginsenoside Rg1 plus doxorubicin-treated cells. Furthermore, the polymerase chain reaction array results illustrate activation of mitogen-activated protein kinase (MAPK) gene expression (AKT, ERK, and MAPK) during doxorubicin alone treatment and it has been attenuated by ginsenoside Rg1 pretreatment. Moreover, ginsenoside Rg1 treatment before doxorubicin activates the DNA damage response elements (ATM, H2AX, RAD51, and XRCC1) and subsequent apoptosis-related gene expression (p21, TP53. APAF1, Bax, CASP3, and CASP9) patterns in MDA-MB-231 cell lines. The ginsenoside Rg1 plus doxorubicin combination shows less cytotoxicity and ROS generation in MDA10A normal breast cancer cell lines. Therefore, the present results support the chemosensitizing property of ginsenoside Rg1 in triple-negative breast cancer cell lines.

C1 [Liu, Shengcui; Gao, Fucun] Linyi Cent Hosp, Dept Galactophore, Linyi, Shandong, Peoples R China.

[Huang, Junhua] Chengdu Fifth Peoples Hosp, Thyroid & Breast Surg Dept, Chengdu, Sichuan, Peoples R China.

[Yin, Zhiping] Yunnan Univ Tradit Chinese Med, Dept Lab Med, Affiliated Hosp 1, Kunming, Yunnan, Peoples R China.

[Zhang, Ruikui] Special Med Ctr Chinese Peoples Armed Police Forc, Dept Surg, Special Serv Emergency, 220 Chenglin Rd, Tianjin 300162, Peoples R China.

C3 Yunnan University of Chinese Medicine

RP Zhang, RK (通讯作者)，Special Med Ctr Chinese Peoples Armed Police Forc, Dept Surg, Special Serv Emergency, 220 Chenglin Rd, Tianjin 300162, Peoples R China.

EM zhangruikuilove@sina.com

CR Ahn B.C., 2015, PLOS ONE, V10

Attele AS, 1999, BIOCHEM PHARMACOL, V58, P1685, DOI 10.1016/S0006-2952(99)00212-9

BREDEHORST R, 1987, J BIOL CHEM, V262, P2034

Cheng Y, 2020, BIOORG MED CHEM LETT, V30, DOI 10.1016/j.bmcl.2020.127364

Christowitz C, 2019, BMC CANCER, V19, DOI 10.1186/s12885-019-5939-z

Chu Y, 2020, EVID-BASED COMPL ALT, V2020, DOI 10.1155/2020/8886955

CUMMINGS J, 1992, BIOCHEM PHARMACOL, V44, P2165, DOI 10.1016/0006-2952(92)90343-H

Dang YP, 2015, EXP THER MED, V9, P1470, DOI 10.3892/etm.2015.2240

Davis T, 2018, BIOCHEM PHARMACOL, V148, P174, DOI 10.1016/j.bcp.2018.01.012

Deng Y, 2018, MOL CARCINOGEN, V57, P807, DOI 10.1002/mc.22795

Di Sotto A, 2020, INT J MOL SCI, V21, DOI 10.3390/ijms21020633

Flick MB, 2004, J SOC GYNECOL INVEST, V11, P252, DOI 10.1016/j.jsgi.2003.11.003

Frion-Herrera Y, 2019, FITOTERAPIA, V136, DOI 10.1016/j.fitote.2019.104173

George BP, 2019, OXID MED CELL LONGEV, V2019, DOI 10.1155/2019/6797921

Gupta, 2016, NAT PROD CHEM RES, V4, P114

He F, 2020, FRONT PHARMACOL, V10, DOI 10.3389/fphar.2019.01565

Huang, 2017, NUTRIENTS, V9, P118

Huang JF, 2018, CANCER CHEMOTH PHARM, V82, P199, DOI 10.1007/s00280-018-3603-y

Jing SY, 2020, J FUNCT FOODS, V72, DOI 10.1016/j.jff.2020.104042

Kumar P, 2016, ARCH GYNECOL OBSTET, V293, P247, DOI 10.1007/s00404-015-3859-y

Kumar Priti, 2018, Cold Spring Harb Protoc, V2018, DOI 10.1101/pdb.prot095505

L?pez, 2021, MEDICINE, V13, P1506

Lee S.Y., 2020, EVID-BASED COMPL ALT, V10

Li GX, 2008, FOOD CHEM TOXICOL, V46, P886, DOI 10.1016/j.fct.2007.10.020

Li W, 2019, J BUON, V24, P2056

Loibl S, 2018, LANCET ONCOL, V19, P497, DOI 10.1016/S1470-2045(18)30111-6

Ma, 2020, J PHARM BIOTECHNOL, V27, P122

Siegel RL, 2016, CA-CANCER J CLIN, V66, P7, DOI [10.3322/caac.21332, 10.3322/caac.21590, 10.3322/caac.21708]

Swift LP, 2006, CANCER RES, V66, P4863, DOI 10.1158/0008-5472.CAN-05-3410

Tacar O, 2013, J PHARM PHARMACOL, V65, P157, DOI 10.1111/j.2042-7158.2012.01567.x

Tan W, 2014, MOL MED REP, V10, P3275, DOI 10.3892/mmr.2014.2598

Telli M.L., 2016, MOL PATHOLOGY BREAST

Towfida Jahan, 2020, FUNCTIONAL FOODS CAN, V405

Wang Chong-Zhi, 2015, Diseases, V3, P193

Wen CJ, 2019, MOL MED REP, V19, P5162, DOI 10.3892/mmr.2019.10180

Wen SH, 2018, CANCER CELL INT, V18, DOI 10.1186/s12935-018-0625-9

Xu R., 2019, EUR J PHARMACOL, V856

Xu ZM, 2018, INT J MOL SCI, V19, DOI 10.3390/ijms19113658

Yin L, 2020, BREAST CANCER RES, V22, DOI 10.1186/s13058-020-01296-5

Yu HL, 2018, CELL PHYSIOL BIOCHEM, V48, P2470, DOI 10.1159/000492684

Yu ML, 2015, MOL MED REP, V11, P3167, DOI 10.3892/mmr.2014.3098

Zhang ZR, 2021, MOL NEUROBIOL, V58, P1550, DOI 10.1007/s12035-020-02213-9

Zhao YD, 2020, CANCER MED-US, V9, P6281, DOI 10.1002/cam4.3284

Zhu C, 2017, ONCOTARGET, V8, P83792, DOI 10.18632/oncotarget.19698

Zhu H, 2021, J ETHNOPHARMACOL, V265, DOI 10.1016/j.jep.2020.113271

NR 45

TC 2

Z9 3

U1 10

U2 19

PU WILEY

PI HOBOKEN

PA 111 RIVER ST, HOBOKEN 07030-5774, NJ USA

SN 1095-6670

EI 1099-0461

J9 J BIOCHEM MOL TOXIC

JI J. Biochem. Mol. Toxicol.

PD JAN

PY 2022

VL 36

IS 1

AR e22945

DI 10.1002/jbt.22945

EA NOV 2021

PG 10

WC Biochemistry & Molecular Biology; Toxicology

WE Science Citation Index Expanded (SCI-EXPANDED)

SC Biochemistry & Molecular Biology; Toxicology

GA YI4VY

UT WOS:000718795600001

PM 34783124

DA 2023-04-05

ER

PT J

AU Li, CQ

Gou, XB

Gao, H

AF Li, Chaoqi

Gou, Xiangbo

Gao, Hui

TI Doxorubicin nanomedicine based on ginsenoside Rg1 with alleviated

cardiotoxicity and enhanced antitumor activity

SO NANOMEDICINE

LA English

DT Article

DE apoptosis; breast cancer; cardiac protective effect; cardiotoxicity;

cardiovascular; doxorubicin; ginsenoside Rg1; nanomedicine delivery;

nanoparticles; tumor-targeting therapy

ID INDUCED APOPTOSIS; OXIDATIVE STRESS; DRUG-DELIVERY; TOXICITY; INJURY;

NANOPARTICLES; MECHANISMS; PATHWAY; IMPACT; CELLS

AB Lay abstract Doxorubicin (Dox) is a drug used to treat cancer; however, it can be toxic to the heart. In this study, researchers made nanoparticles containing Dox and a component of ginseng, a root similar to ginger. They tested the nanoparticles in mice with tumors. The nanoparticles appeared to gather at the tumor site in greater amounts than free Dox. In healthy mice, the nanoparticles gathered less in the heart than free Dox. This means that putting Dox into nanoparticles such as these could improve their anticancer effect and decrease harm to the heart.

Aim: The authors aimed to develop Dox@Rg1 nanoparticles with decreased cardiotoxicity to expand their application in cancer. Materials & methods: Dox@Rg1 nanoparticles were developed by encapsulating doxorubicin (Dox) in a self-assembled Rg1. The antitumor effect of the nanoparticles was estimated using 4T1 tumor-bearing mice and the protective effect on the heart was investigated in vitro and in vivo. Results: Different from Dox, the Dox@Rg1 nanoparticles induced increased cytotoxicity to tumor cells, which was decreased in cardiomyocytes by the inhibition of apoptosis. The study in vivo revealed that the Dox@Rg1 nanoparticles presented a perfect tumor-targeting ability and improved antitumor effects. Conclusion: Dox@Rg1 nanoparticles could enhance the antitumor effects and decrease the cardiotoxicity of Dox.

C1 [Li, Chaoqi; Gou, Xiangbo; Gao, Hui] Tianjin Univ Technol, Sch Chem & Chem Engn, Tianjin Enterprise Key Lab Applicat Res Hyaluron, Tianjin Key Lab Drug Targeting & Bioimaging, Tianjin, Peoples R China.

[Gao, Hui] Tiangong Univ, Sch Mat Sci & Engn, State Key Lab Separat Membranes & Membrane Proc, Tianjin 300384, Peoples R China.

C3 Tianjin University of Technology; Tiangong University

RP Gou, XB; Gao, H (通讯作者)，Tianjin Univ Technol, Sch Chem & Chem Engn, Tianjin Enterprise Key Lab Applicat Res Hyaluron, Tianjin Key Lab Drug Targeting & Bioimaging, Tianjin, Peoples R China.; Gao, H (通讯作者)，Tiangong Univ, Sch Mat Sci & Engn, State Key Lab Separat Membranes & Membrane Proc, Tianjin 300384, Peoples R China.

EM gouxiangbo@163.com; huigao@tiangong.edu.cn

OI li, chaoqi/0000-0001-9263-6783

FU National Natural Science Foundation of China [U20A20260, 22075209]

FX This work was supported by the National Natural Science Foundation of

China (U20A20260, 22075209). The authors have no other relevant

affiliations or financial involvement with any organization or entity

with a financial interest in or financial conflict with the subject

matter or materials discussed in the manuscript apart from those

disclosed.

CR Alyane M, 2016, SAUDI PHARM J, V24, P165, DOI 10.1016/j.jsps.2015.02.014

Angsutararux P, 2015, OXID MED CELL LONGEV, V2015, DOI 10.1155/2015/795602

Argenziano M, 2020, CANCERS, V12, DOI 10.3390/cancers12010162

Bae YH, 2011, J CONTROL RELEASE, V153, P198, DOI 10.1016/j.jconrel.2011.06.001

Cai YL, 2012, MOL BIOL REP, V39, P10705, DOI 10.1007/s11033-012-1961-9

Cao Y, 2020, J EXP CLIN CANC RES, V39, DOI 10.1186/s13046-020-01621-y

Chatterjee K, 2010, CARDIOLOGY, V115, P155, DOI 10.1159/000265166

Cheng CJ, 2015, NAT REV DRUG DISCOV, V14, P239, DOI 10.1038/nrd4503

Deng Y, 2015, PLOS ONE, V10, DOI 10.1371/journal.pone.0116682

Desale JP, 2018, NANOMEDICINE-UK, V13, P2759, DOI 10.2217/nnm-2018-0206

Dong GT, 2016, MITOCHONDRION, V26, P7, DOI 10.1016/j.mito.2015.11.003

Fang JT, 2019, CHEM-BIOL INTERACT, V304, P186, DOI 10.1016/j.cbi.2019.03.012

Farokhzad OC, 2009, ACS NANO, V3, P16, DOI 10.1021/nn900002m

Gao YW, 2015, INT J CLIN EXP MED, V8, P6794

Guo LT, 2020, BIOMED PHARMACOTHER, V125, DOI 10.1016/j.biopha.2019.109784

Hu YH, 2020, ACTA PHARMACOL SIN, V41, P1150, DOI 10.1038/s41401-020-0364-z

Jiang Y, 2021, OXID MED CELL LONGEV, V2021, DOI 10.1155/2021/5896931

Jin X, 2020, NANOMEDICINE-UK, V15, P41, DOI 10.2217/nnm-2018-0479

Kalyanaraman B, 2002, MOL CELL BIOCHEM, V234, P119, DOI 10.1023/A:1015976430790

Kmecova J, 2010, EUR J PHARMACOL, V641, P187, DOI 10.1016/j.ejphar.2010.05.038

Koleini N, 2019, CHEM-BIOL INTERACT, V303, P35, DOI 10.1016/j.cbi.2019.01.032

Lebda MA, 2017, METAB BRAIN DIS, V32, P1639, DOI 10.1007/s11011-017-0052-y

Li MQ, 2014, BIOMATERIALS, V35, P3851, DOI 10.1016/j.biomaterials.2014.01.018

Li PC, 2016, BMC NEUROSCI, V17, DOI 10.1186/s12868-016-0272-9

Lim KH, 2013, J GINSENG RES, V37, P283, DOI 10.5142/jgr.2013.37.283

Lindsey ML, 2018, AM J PHYSIOL-HEART C, V314, pH733, DOI 10.1152/ajpheart.00339.2017

Liu JH, 2008, AM J PHYSIOL-HEART C, V295, pH1956, DOI 10.1152/ajpheart.00407.2008

Ma YY, 2017, BBA-MOL BASIS DIS, V1863, P1904, DOI 10.1016/j.bbadis.2016.12.021

Manaia EB, 2017, INT J NANOMED, V12, P4991, DOI 10.2147/IJN.S133832

Mei SB, 2019, TOXICOL LETT, V307, P41, DOI 10.1016/j.toxlet.2019.02.013

Mozaffari S, 2021, EUR J MED CHEM, V226, DOI 10.1016/j.ejmech.2021.113836

Nagasawa Y, 2020, J PHARMACOL SCI, V143, P39, DOI 10.1016/j.jphs.2020.02.005

Nicol A, 2017, J AM CHEM SOC, V139, P14792, DOI 10.1021/jacs.7b08710

Nousiainen T, 1999, J INTERN MED, V245, P359, DOI 10.1046/j.1365-2796.1999.00480.x

Octavia Y, 2012, J MOL CELL CARDIOL, V52, P1213, DOI 10.1016/j.yjmcc.2012.03.006

Pryor WA, 2006, AM J PHYSIOL-REG I, V291, pR491, DOI 10.1152/ajpregu.00614.2005

Razavi-Azarkhiavi K, 2016, DRUG RES, V66, P330, DOI 10.1055/s-0035-1569447

Sangweni NF, 2020, FRONT PHARMACOL, V11, DOI 10.3389/fphar.2020.01172

Sawyer DB, 2010, PROG CARDIOVASC DIS, V53, P105, DOI 10.1016/j.pcad.2010.06.007

Shabalala SC, 2019, TOXICOL IN VITRO, V55, P134, DOI 10.1016/j.tiv.2018.12.012

Shim MK, 2019, J CONTROL RELEASE, V294, P376, DOI 10.1016/j.jconrel.2018.11.032

Silva JD, 2019, BIOMED PHARMACOTHER, V118, DOI 10.1016/j.biopha.2019.109323

Sivapackiam J, 2019, PLOS ONE, V14, DOI 10.1371/journal.pone.0215579

Sun JM, 2013, PLOS ONE, V8, DOI 10.1371/journal.pone.0064816

Sykes EA, 2014, ACS NANO, V8, P5696, DOI 10.1021/nn500299p

Tacar O, 2013, J PHARM PHARMACOL, V65, P157, DOI 10.1111/j.2042-7158.2012.01567.x

Tadokoro T, 2020, JCI INSIGHT, V5, DOI 10.1172/jci.insight.132747

Tang FT, 2016, J CARDIOVASC PHARM, V68, P257, DOI 10.1097/FJC.0000000000000410

Tao JS, 2019, ACTA PHARM SIN B, V9, P4, DOI 10.1016/j.apsb.2018.11.001

Wang Q, 2020, BIOMED PHARMACOTHER, V122, DOI 10.1016/j.biopha.2019.109547

Warhol A, 2021, PHYSIOL REP, V9, DOI 10.14814/phy2.14987

Wei HX, 2019, INT J NANOMED, V14, P8603, DOI 10.2147/IJN.S218988

Wen JX, 2019, FRONT PHARMACOL, V10, DOI 10.3389/fphar.2019.01135

Xiao J, 2012, TOXICOLOGY, V292, P53, DOI 10.1016/j.tox.2011.11.018

Xiong J, 2008, INT J PHARMACEUT, V360, P191, DOI 10.1016/j.ijpharm.2008.04.016

Xu WG, 2015, BIOMATERIALS, V54, P72, DOI 10.1016/j.biomaterials.2015.03.021

Xu ZM, 2018, INT J MOL SCI, V19, DOI 10.3390/ijms19113658

Yang F, 2014, BBA-REV CANCER, V1845, P84, DOI 10.1016/j.bbcan.2013.12.002

Yoshida M, 2009, J MOL CELL CARDIOL, V47, P698, DOI 10.1016/j.yjmcc.2009.07.024

Yuan J, 2003, BBA-MOL CELL RES, V1641, P35, DOI 10.1016/S0167-4889(03)00047-8

Zhang L, 2021, INT J MOL MED, V47, DOI 10.3892/ijmm.2021.4896

Zhang X, 2019, INT J BIOL SCI, V15, P556, DOI 10.7150/ijbs.29907

Zhang YJ, 2013, J CARDIOVASC PHARM, V62, P50, DOI 10.1097/FJC.0b013e31828f8d45

Zhang ZH, 2016, J CONTROL RELEASE, V225, P96, DOI 10.1016/j.jconrel.2016.01.035

Zhou WN, 2021, MOLECULES, V26, DOI 10.3390/molecules26071946

Zhu C, 2017, ONCOTARGET, V8, P83792, DOI 10.18632/oncotarget.19698

NR 66

TC 3

Z9 3

U1 5

U2 23

PU FUTURE MEDICINE LTD

PI LONDON

PA UNITEC HOUSE, 3RD FLOOR, 2 ALBERT PLACE, FINCHLEY CENTRAL, LONDON, N3

1QB, ENGLAND

SN 1743-5889

EI 1748-6963

J9 NANOMEDICINE-UK

JI Nanomedicine

PD DEC

PY 2021

VL 16

IS 29

BP 2587

EP 2604

DI 10.2217/nnm-2021-0329

EA NOV 2021

PG 18

WC Biotechnology & Applied Microbiology; Nanoscience & Nanotechnology

WE Science Citation Index Expanded (SCI-EXPANDED)

SC Biotechnology & Applied Microbiology; Science & Technology - Other

Topics

GA XJ6AK

UT WOS:000713155000001

PM 34719938

DA 2023-04-05

ER

PT J

AU Wei, HM

Guo, CX

Zhu, RL

Zhang, CG

Han, NN

Liu, R

Hua, BJ

Li, YF

Lin, H

Yu, J

AF Wei, Huamin

Guo, Chunxiu

Zhu, Ruili

Zhang, Congen

Han, Nina

Liu, Rui

Hua, Baojin

Li, Yangfan

Lin, Hai

Yu, Jing

TI Shuangshen granules attenuate lung metastasis by modulating bone marrow

differentiation through mTOR signalling inhibition

SO JOURNAL OF ETHNOPHARMACOLOGY

LA English

DT Article

DE Lung metastasis; MDSC; mTOR signalling pathway; Shuangshen granules;

Traditional Chinese medicine

ID PANAX-NOTOGINSENG; SUPPRESSOR-CELLS; WATER EXTRACT; MACROPHAGE;

POLARIZATION; MECHANISM; PATHWAY; GINSENG

AB Ethnopharmacological relevance: Traditional Chinese medicine Shuangshen granules (SSG) have been used to treat lung cancer patients with Qi deficiency and blood stasis for decades. According to clinical experience, SSG indeed improve the quality of life and prolong the survival time of patients with lung cancer after surgery. Each of the components herbs was proved to be effective in anti-cancer therapy. Both the American ginseng and notoginseng belong to genus Panax of the family Araliaceae. Preclinical and clinical studies demonstrated that ginsenosides of them have anti- or preventive activities to various tumors, including cancers of gastric, breast, liver, lung, ovarian, colon, melanoma and leukemia. PDS, such as ginsenoside Rb1, and PTS, such as ginsenoside Rg1 are the main anticancer compositions. Cordyceps sinensis had also been found effective in inhibiting tumour growth and metastasis, especially on tumour associated immune cells, such as macrophages. However, limited information is available regarding potential mechanisms of SSG. Myeloid-derived suppressor cell (MDSC)-mediated immunosuppression, which is closely associated with poor clinical outcomes in cancer patients, may be the target of SSG, which regulate immune function.

Aim of the study: The present study aimed to explore whether SSG attenuate the differentiation of bone marrow cells (BMCs) into MDSCs by blocking the mTOR signalling, leading to the suppression of lung metastasis.

Materials and methods: First, we observed the differentiation of BMCs into MDSCs in vitro and in vivo. BMCs were cultured alone or co-cultured with Lewis lung carcinoma (LLC) cell supernatant in vitro. The effects of different concentrations of SSG, or LLC cell supernatant as a control, on BMC differentiation were detected by flow cytometry and western blotting. Male C57BL/6J mice were subcutaneously implanted with LLC cells, and SSG were administered by gavage twice daily before and after implantation for 7 or 14 days, respectively. The tumour weight, proportion of MDSCs, presence of CD11b(+)Ly6C(+)Ly6G- and CD11b(+)Ly6C(+)Ly6G(+) cells in the bone marrow, blood, and lungs, as well as the expression levels of differentiation-related proteins in the bone marrow and lungs were evaluated.

Results: SSG attenuated the differentiation of BMCs into MDSCs, and reduced the fraction of CD11b(+)Ly6C(+)Ly6G(+) cells by inhibiting the mTOR/S6K1/Myc signalling pathway. In vivo, SSG attenuated differentiation-associated protein markers and reduced the fractions of MDSCs and CD11b(+)Ly6C(+)Ly6G(+) cells in the bone marrow, blood, and lungs. In addition, SSG administration reduced the tumour weight and inhibited lung metastasis.

Conclusions: SSG may reduce lung metastasis by attenuating BMC differentiation into CD11b(+)Ly6C(+)Ly6G(+) cells by inhibiting mTOR signalling in vitro and in vivo.

C1 [Wei, Huamin; Zhang, Congen; Li, Yangfan; Lin, Hai; Yu, Jing] Capital Med Univ, Beijing Friendship Hosp, 95 Yong Rd, Beijing 100053, Peoples R China.

[Guo, Chunxiu; Zhu, Ruili; Liu, Rui; Hua, Baojin] China Acad Chinese Med Sci, Guangan Men Hosp, 5 Beixiange St, Beijing, Peoples R China.

[Han, Nina] Beijing Tcmages Pharmaceut Co Ltd, Beijing 101301, Peoples R China.

C3 Capital Medical University; China Academy of Chinese Medical Sciences

RP Li, YF; Yu, J (通讯作者)，Capital Med Univ, Beijing Friendship Hosp, 95 Yong Rd, Beijing 100053, Peoples R China.

EM weihuamin6255@126.com; 5627284@qq.com; zhuruili6255@126.com;

zce820@163.com; 445909206@qq.com; drliur@126.com; huabaojin@sohu.com;

lyfdzyx@vip.163.com; lh_js@sohu.com; yujing026@163.com

FU Beijing Natural Science Foundation [7184203]; National Natural Science

Foundation of China [81904102]

FX This work was supported by the Beijing Natural Science Foundation

(No.7184203) and National Natural Science Foundation of China

(No.81904102) .

CR Caras I, 2011, TUMORI J, V97, P647, DOI 10.1700/989.10726

Chen Ci-Hui, 2017, Zhongguo Zhong Xi Yi Jie He Za Zhi, V37, P338

Chen Pei-Feng, 2006, Zhong Xi Yi Jie He Xue Bao, V4, P500, DOI 10.3736/jcim20060512

Chen TL, 2018, SAUDI J BIOL SCI, V25, P917, DOI 10.1016/j.sjbs.2018.01.012

Chen YN, 2019, J ETHNOPHARMACOL, V232, P11, DOI 10.1016/j.jep.2018.12.003

Condamine T, 2011, TRENDS IMMUNOL, V32, P19, DOI 10.1016/j.it.2010.10.002

Corzo CA, 2009, J IMMUNOL, V182, P5693, DOI 10.4049/jimmunol.0900092

Deng YT, 2018, CANCER IMMUNOL IMMUN, V67, P1355, DOI 10.1007/s00262-018-2177-1

Ding XC, 2014, AM J PATHOL, V184, P397, DOI 10.1016/j.ajpath.2013.10.015

Fleming V, 2018, FRONT IMMUNOL, V9, DOI 10.3389/fimmu.2018.00398

Guo Y, 2018, NEUROCHEM RES, V43, P1927, DOI 10.1007/s11064-018-2612-x

Haile LA, 2012, IMMUNOL INVEST, V41, P581, DOI 10.3109/08820139.2012.680635

He NW, 2012, J MED FOOD, V15, P350, DOI 10.1089/jmf.2011.1801

Heukels P, 2019, RESP RES, V20, DOI 10.1186/s12931-019-1195-7

Khan MA, 2020, CURR MED CHEM, V27, P983, DOI 10.2174/0929867325666181001105749

Kim B, 2018, AM J CHINESE MED, V46, P1369, DOI [10.1142/S0192415X18500726, 10.1142/s0192415x18500726]

Kowanetz M, 2010, P NATL ACAD SCI USA, V107, P21248, DOI 10.1073/pnas.1015855107

Li Fei-fei, 2013, Zhongguo Zhong Xi Yi Jie He Za Zhi, V33, P1086

Li TJ, 2019, BIOCHEM BIOPH RES CO, V516, P632, DOI 10.1016/j.bbrc.2019.06.108

Liu HH, 2019, PLOS ONE, V14, DOI 10.1371/journal.pone.0216759

Liu Rui, 2018, Zhongguo Zhong Yao Za Zhi, V43, P3913, DOI 10.19540/j.cnki.cjcmm.20180806.001

Liu Y, 2016, CANCER CELL, V30, P668, DOI 10.1016/j.ccell.2016.09.011

Park SC, 2009, INT J ONCOL, V35, P121, DOI 10.3892/ijo_00000320

Raber PL, 2014, INT J CANCER, V134, P2853, DOI 10.1002/ijc.28622

Shin MS, 2017, INT J BIOL MACROMOL, V103, P1327, DOI 10.1016/j.ijbiomac.2017.05.055

Tcyganov E, 2018, CURR OPIN IMMUNOL, V51, P76, DOI 10.1016/j.coi.2018.03.009

Toh DF, 2011, CHIN MED-UK, V6, DOI 10.1186/1749-8546-6-4

Tu L, 2018, NEUROCHEM RES, V43, P1210, DOI 10.1007/s11064-018-2538-3

Wang PW, 2014, J ETHNOPHARMACOL, V154, P663, DOI 10.1016/j.jep.2014.04.037

Wang ZX, 2018, BIOSCI TRENDS, V12, P220, DOI 10.5582/bst.2018.01144

Wei H.M., 2017, WORLD CHIN MED, V12, P394

[魏华民(综述) Wei Huamin], 2017, [中国癌症杂志, China Oncology], V27, P516

Yan YT, 2018, BEHAV BRAIN RES, V345, P83, DOI 10.1016/j.bbr.2018.02.037

Zhang P, 2000, Zhongguo Zhong Xi Yi Jie He Za Zhi, V20, P31

Zheng Hong-gang, 2010, Zhongguo Zhong Xi Yi Jie He Za Zhi, V30, P1288

Zhou QQ, 2018, ONCOL LETT, V16, P6930, DOI 10.3892/ol.2018.9518

NR 36

TC 5

Z9 5

U1 3

U2 27

PU ELSEVIER IRELAND LTD

PI CLARE

PA ELSEVIER HOUSE, BROOKVALE PLAZA, EAST PARK SHANNON, CO, CLARE, 00000,

IRELAND

SN 0378-8741

EI 1872-7573

J9 J ETHNOPHARMACOL

JI J. Ethnopharmacol.

PD DEC 5

PY 2021

VL 281

AR 113305

DI 10.1016/j.jep.2020.113305

EA AUG 2021

PG 10

WC Plant Sciences; Chemistry, Medicinal; Integrative & Complementary

Medicine; Pharmacology & Pharmacy

WE Science Citation Index Expanded (SCI-EXPANDED)

SC Plant Sciences; Pharmacology & Pharmacy; Integrative & Complementary

Medicine

GA WD2CP

UT WOS:000704755700004

PM 32890710

DA 2023-04-05

ER

PT J

AU Liu, S

Cheng, Y

Chen, WZ

Lv, JX

Zheng, BS

Huang, DD

Xia, XF

Yu, Z

AF Liu, Shu

Cheng, Yue

Chen, Wei-Zhe

Lv, Jin-Xiao

Zheng, Bei-Shi

Huang, Dong-Dong

Xia, Xu-Fen

Yu, Zhen

TI Inflammation Disturbed the Tryptophan Catabolites in Hippocampus of

Post-operative Fatigue Syndrome Rats via Indoleamine 2,3-Dioxygenas

Enzyme and the Improvement Effect of Ginsenoside Rb1

SO FRONTIERS IN NEUROSCIENCE

LA English

DT Article

DE post-operative fatigue syndrome; inflammatory cytokine; p38MAPK;

NF-?B/p65; IDO; ginsenoside Rb1

ID ACTIVATED PROTEIN-KINASE; BREAST-CANCER; SERUM; METAANALYSIS;

KYNURENINES; RECOVERY; PATHWAY; ACID

AB Aim: Post-operative fatigue syndrome (POFS) is a common complication that prolongs the recovery to normal function and activity after surgery. The aim of the present study was to explore the mechanism of central fatigue in POFS and the anti-fatigue effect of ginsenoside Rb1.

Method: We investigated the association between inflammation, indoleamine 2,3-dioxygenase (IDO) enzyme, and tryptophan metabolism in the hippocampus of POFS rats. A POFS rat model was induced by major small intestinal resection. Rats with major small intestinal resection were administered ginsenoside Rb1 (15 mg/kg) once a day from 3 days before surgery to the day of sacrifice, or with saline as corresponding controls. Fatigue was assessed with the open field test (OFT) and sucrose preference test (SPT). ELISA, RT-PCR, Western blot, immunofluorescence, and high-performance liquid chromatography (HPLC) were used to test the inflammatory cytokines; p38MAPK, NF-kappa B/p65, and IDO enzyme expressions; and the concentrations of tryptophan, kynurenine, and serotonin, respectively.

Result: Our results showed that POFS was associated with increased expressions of inflammatory cytokines and p38MAPK and higher concentrations of kynurenine and tryptophan on post-operative days 1 and 3; a lower serotonin level on post-operative day 1; and an enhanced translocation of NF-kappa B/p65 and the IDO enzyme on post-operative days 1, 3, and 5. Ginsenoside Rb1 had an improvement effect on these.

Conclusion: Inflammatory cytokines induced by large abdominal surgery disturb tryptophan metabolism to cause POFS through the activation of the p38MAPK-NF-kappa B/p65-IDO pathway in the hippocampus. Ginsenoside Rb1 had an anti-fatigue effect on POFS by reducing inflammation and IDO enzyme.

C1 [Liu, Shu] City Univ Hong Kong, Coll Vet Med & Life Sci, Dept Biomed Sci, Kowloon Tong, Hong Kong, Peoples R China.

[Cheng, Yue; Xia, Xu-Fen] Tongde Hosp Zhejiang Prov, Dept Clin Lab, Hangzhou, Peoples R China.

[Chen, Wei-Zhe; Lv, Jin-Xiao; Zheng, Bei-Shi; Huang, Dong-Dong] Wenzhou Med Univ, Dept Gastrointestinal Surg, Affiliated Hosp 1, Wenzhou, Peoples R China.

[Yu, Zhen] Tongii Univ, Dept Surg, Shanghai Peoples Hosp 10, Shanghai, Peoples R China.

C3 City University of Hong Kong; Wenzhou Medical University; Tongji

University

RP Xia, XF (通讯作者)，Tongde Hosp Zhejiang Prov, Dept Clin Lab, Hangzhou, Peoples R China.; Yu, Z (通讯作者)，Tongii Univ, Dept Surg, Shanghai Peoples Hosp 10, Shanghai, Peoples R China.

EM xiaxufen123@163.com; scnlhczj@163.com

RI Liu, Shu/HGT-8732-2022; chen, johnny/GQI-1255-2022

OI chen, johnny/0000-0001-5253-9911

FU National Natural Science Foundation of China [81171857]; Natural Science

Foundation of Zhejiang Province [LZ12H07001]; Ministry of Health and

Zhejiang Province Co-founded Program [WKJ2012-2-034]; Health Department

of Zhejiang Program [2013ZDA014]; Clinical Nutriology Area of the

Medical support discipline of Zhejiang Province [11-ZC24]

FX This work was supported by the National Natural Science Foundation of

China (No. 81171857), the Natural Science Foundation of Zhejiang

Province (No. LZ12H07001), the Ministry of Health and Zhejiang Province

Co-founded Program (No. WKJ2012-2-034), and the Health Department of

Zhejiang Program (No. 2013ZDA014). Also partly supported by the Clinical

Nutriology Area of the Medical support discipline of Zhejiang Province

(No. 11-ZC24).

CR Ahles TA, 2002, J CLIN ONCOL, V20, P485, DOI 10.1200/JCO.2002.20.2.485

Barton DL, 2010, SUPPORT CARE CANCER, V18, P179, DOI 10.1007/s00520-009-0642-2

Bjorklund G, 2019, BIOMED PHARMACOTHER, V109, P1000, DOI 10.1016/j.biopha.2018.10.076

Bower JE, 2002, PSYCHOSOM MED, V64, P604, DOI 10.1097/00006842-200207000-00010

CHRISTENSEN T, 1982, BRIT J SURG, V69, P417, DOI 10.1002/bjs.1800690721

DeCherney AH, 2002, OBSTET GYNECOL, V99, P51, DOI 10.1016/S0029-7844(01)01622-2

Du J, 2011, J IMMUNOL, V187, P942, DOI 10.4049/jimmunol.1002579

Fernstrom JD, 2006, J NUTR, V136, p553S, DOI 10.1093/jn/136.2.553S

Fujigaki H, 2006, J BIOCHEM, V139, P655, DOI 10.1093/jb/mvj072

Goehler LE, 2007, BRAIN BEHAV IMMUN, V21, P721, DOI 10.1016/j.bbi.2007.02.005

Guillemin GJ, 2005, GLIA, V49, P15, DOI 10.1002/glia.20090

Herve C, 1996, J CHROMATOGR B, V675, P157, DOI 10.1016/0378-4347(95)00341-X

Huang YS, 2020, FRONT IMMUNOL, V11, DOI 10.3389/fimmu.2020.00388

Kim HJ, 2006, TOXICOLOGY, V225, P36, DOI 10.1016/j.tox.2006.04.053

Kobrzycka A, 2019, J NEUROINFLAMM, V16, DOI 10.1186/s12974-019-1544-y

Lanser L, 2020, FRONT IMMUNOL, V11, DOI 10.3389/fimmu.2020.00249

Larkin D, 2017, NEUROPHYSIOL CLIN, V47, P123, DOI 10.1016/j.neucli.2017.01.012

Lee N, 2016, J ALTERN COMPLEM MED, V22, P859, DOI 10.1089/acm.2016.0057

Liu W, 2017, NEURAL PLAST, V2017, DOI 10.1155/2017/6871089

Lu Y, 2016, J CLIN BIOCHEM NUTR, V58, P210, DOI 10.3164/jcbn.15-72

Manjaly ZM, 2019, J NEUROL NEUROSUR PS, V90, P642, DOI 10.1136/jnnp-2018-320050

Morris G, 2016, MOL NEUROBIOL, V53, P1195, DOI 10.1007/s12035-015-9090-9

Myint AM, 2012, FEBS J, V279, P1375, DOI 10.1111/j.1742-4658.2012.08551.x

Rubin GJ, 2004, J PSYCHOSOM RES, V57, P317, DOI 10.1016/S0022-3999(03)00615-9

Rubin GJ, 2002, BRIT J SURG, V89, P971, DOI 10.1046/j.1365-2168.2002.02138.x

Schreiner P, 2021, ALIMENT PHARM THER, V53, P138, DOI 10.1111/apt.16145

SCHROEDER D, 1991, AUST NZ J SURG, V61, P774, DOI 10.1111/j.1445-2197.1991.tb01682.x

Schwarcz R, 2002, J PHARMACOL EXP THER, V303, P1, DOI 10.1124/jpet.102.034439

Shergis JL, 2013, PHYTOTHER RES, V27, P949, DOI 10.1002/ptr.4832

Suarez AN, 2018, NAT COMMUN, V9, DOI 10.1038/s41467-018-04639-1

Szeitz A, 2018, BIOMED CHROMATOGR, V32, DOI 10.1002/bmc.4135

Wang R, 2006, CHIN J CHROMATOGR, V24, P140, DOI 10.1016/S1872-2059(06)60009-6

Xiong J, 2019, BMC ANESTHESIOL, V19, DOI 10.1186/s12871-019-0885-5

Yamamoto T, 1997, BRAIN RES BULL, V43, P43, DOI 10.1016/S0361-9230(96)00344-9

Yamamoto T, 2012, CAN J NEUROL SCI, V39, P40, DOI 10.1017/S031716710001266X

Yamashita M, 2014, INT J TRYPTOPHAN RES, V7, P9, DOI [10.4137/IJTRTR.S14084, 10.4137/IJTR.S14084]

Zargar-Shoshtari K, 2009, WORLD J SURG, V33, P738, DOI 10.1007/s00268-008-9906-0

Zargar-Shoshtari K, 2009, J SURG RES, V154, P330, DOI 10.1016/j.jss.2008.06.023

Zhang JQ, 2020, PSYCHOPHARMACOLOGY, V237, P2531, DOI 10.1007/s00213-020-05553-5

Zhang XD, 2011, SCAND J GASTROENTERO, V46, P1302, DOI 10.3109/00365521.2011.610001

Zhuang CL, 2014, EUR J PHARMACOL, V740, P480, DOI 10.1016/j.ejphar.2014.06.040

NR 41

TC 2

Z9 2

U1 3

U2 10

PU FRONTIERS MEDIA SA

PI LAUSANNE

PA AVENUE DU TRIBUNAL FEDERAL 34, LAUSANNE, CH-1015, SWITZERLAND

EI 1662-453X

J9 FRONT NEUROSCI-SWITZ

JI Front. Neurosci.

PD AUG 26

PY 2021

VL 15

AR 652817

DI 10.3389/fnins.2021.652817

PG 11

WC Neurosciences

WE Science Citation Index Expanded (SCI-EXPANDED)

SC Neurosciences & Neurology

GA UU0TK

UT WOS:000698517600001

PM 34512234

OA Green Published, gold

DA 2023-04-05

ER

PT J

AU Cai, ZX

Teng, Y

Chen, Y

AF Cai, Zhixing

Teng, Yue

Chen, Yue

TI The Effect of Shenyi Capsule on Non-Small-Cell Lung Cancer Combined with

Chemotherapy from the Yin-Yang Perspective

SO EVIDENCE-BASED COMPLEMENTARY AND ALTERNATIVE MEDICINE

LA English

DT Review

ID NF-KAPPA-B; EPITHELIAL-MESENCHYMAL TRANSITION; PLATINUM-BASED

CHEMOTHERAPY; GINSENOSIDE RG3; BREAST-CANCER; 20(S)-GINSENOSIDE;

NORMALIZATION; CYTOTOXICITY; ANGIOGENESIS; METAANALYSIS

AB As an example of Shenyi capsule on non-small-cell lung cancer combined with chemotherapy, this review discusses the synergistic effect and mechanism of natural drugs in oncotherapy from the yin-yang perspective in ancient Chinese philosophy, so as to reflect the therapeutic principle of natural drugs for tumor more comprehensively. The major focuses of this review are on the philosophical thinking of yin-yang as a tool which can not only explain the effect of Shenyi capsule in NSCLC combined with chemotherapy but also explore the mechanism of Shenyi capsule at the cellular and molecular level. Learning from the "yin-yang" thinking of ancient Chinese philosophy will bring more enlightenment to the research and development of traditional Chinese drugs in the future.

C1 [Cai, Zhixing; Chen, Yue] Shanghai Jiao Tong Univ, Tongren Hosp, Sch Med, Dept TCM, 1111 Xianxia Rd, Shanghai 200336, Peoples R China.

[Teng, Yue] Shanghai Univ Tradit Chinese Med, Yueyang Hosp Integrated Tradit Chinese & Western, Clin Ctr, Outpatient Dept, 110 Ganhe Rd, Shanghai 200437, Peoples R China.

C3 Shanghai Jiao Tong University; Shanghai University of Traditional

Chinese Medicine

RP Chen, Y (通讯作者)，Shanghai Jiao Tong Univ, Tongren Hosp, Sch Med, Dept TCM, 1111 Xianxia Rd, Shanghai 200336, Peoples R China.

EM czx3664@shtrhospital.com; tengyue1110@163.com; cy3371@sina.cn

OI Teng, Yue/0000-0002-8285-9083

FU Horizontal Cooperation Project of NSFC [TR2017T02]; General Discipline

Construction Project of Tongren Hospital [2020xk27]; 7th batch of Young

Talents Training Project of Yueyang Hospital [41.06.75]

FX This article was supported by a Horizontal Cooperation Project of NSFC

(TR2017T02), a General Discipline Construction Project of Tongren

Hospital (2020xk27), and the 7th batch of Young Talents Training Project

of Yueyang Hospital (41.06.75).

CR Arbour KC, 2019, JAMA-J AM MED ASSOC, V322, P764, DOI 10.1001/jama.2019.11058

Aziz F, 2016, TOXICOL IN VITRO, V31, P158, DOI 10.1016/j.tiv.2015.09.025

Bae EA, 2002, BIOL PHARM BULL, V25, P58, DOI 10.1248/bpb.25.58

Bowen JM, 2006, SUPPORT CARE CANCER, V14, P713, DOI 10.1007/s00520-005-0004-7

Bozic I, 2020, NAT CANCER, V1, P580, DOI 10.1038/s43018-020-0079-6

Burdett S, 2008, J CLIN ONCOL, V26, P4617, DOI 10.1200/JCO.2008.17.7162

Chen Ming-wei, 2005, Zhonghua Jiehe He Huxi Zazhi, V28, P37

Cheong JH, 2015, BIOL PHARM BULL, V38, P102, DOI 10.1248/bpb.b14-00603

Dai YM, 2019, EXP THER MED, V17, P953, DOI 10.3892/etm.2018.7001

[董广通 Dong Guangtong], 2017, [中国新药杂志, Chinese Journal New Drugs], V26, P1683

Ghosh S, 2019, BIOORG CHEM, V88, DOI 10.1016/j.bioorg.2019.102925

Goel S, 2011, PHYSIOL REV, V91, P1071, DOI 10.1152/physrev.00038.2010

Gyrd-Hansen M, 2010, NAT REV CANCER, V10, P561, DOI 10.1038/nrc2889

[韩荣龙 Han Ronglong], 2018, [中国实验方剂学杂志, Chinese Journal of Experimental Traditional Medical Formulae], V24, P201

Hong SZ, 2020, ONCOL REP, V44, P1333, DOI 10.3892/or.2020.7728

Kim ES, 2016, ADV EXP MED BIOL, V893, P189, DOI 10.1007/978-3-319-24223-1_10

Kim SM, 2010, EUR J PHARMACOL, V631, P1, DOI 10.1016/j.ejphar.2009.12.018

Kim YJ, 2014, TOXICOLOGY, V322, P23, DOI 10.1016/j.tox.2014.04.002

[寇小格 Kou Xiaoge], 2011, [肿瘤防治研究, Cancer Research on Prevention and Treatment], V38, P319

Kulikov A.Yu., 2015, [Фармакоэкономика: теория и практика, Farmakoekonomika: teoriya i praktika], V3, P86

Phi LTH, 2019, ONCOTARGETS THER, V12, P10885, DOI 10.2147/OTT.S219063

Lee YJ, 2014, ONCOL REP, V32, P1803, DOI 10.3892/or.2014.3452

Li HX, 2019, J CANCER, V10, P5283, DOI 10.7150/jca.31928

Liang HC, 2017, METAB ENG, V44, P60, DOI 10.1016/j.ymben.2017.07.008

Ling Y.X, 2008, LING YAOXINGS LECT N

Liu H, 2012, CANCER IMMUNOL IMMUN, V61, P1849, DOI 10.1007/s00262-012-1231-7

Liu L, 2010, PHYTOCHEMISTRY, V71, P1514, DOI 10.1016/j.phytochem.2010.05.007

Liu TH, 2019, BIOMED PHARMACOTHER, V120, DOI 10.1016/j.biopha.2019.109483

Liu ZY, 2021, MOL BIOL REP, V48, P2639, DOI 10.1007/s11033-021-06187-2

Lu H, 2017, J MOL CELL BIOL, V9, P1, DOI 10.1093/jmcb/mjx003

Lu MX, 2018, BIOMED PHARMACOTHER, V97, P1282, DOI 10.1016/j.biopha.2017.11.006

[陆瑞 Lu Rui], 2019, [中国细胞生物学学报, Chinese Journal of Cell Biology], V41, P1787

Luo G.Y, 2013, APOCALYPSE TRADITION, P307

Mao XH, 2020, EVID-BASED COMPL ALT, V2020, DOI 10.1155/2020/6065124

Min Lee Seong, 2019, [Biomedical Science Letters, 대한의생명과학회지], V25, P293

Mohanan P, 2018, J GINSENG RES, V42, P123, DOI 10.1016/j.jgr.2017.01.008

Nallanthighal S, 2019, FRONT CELL DEV BIOL, V7, DOI 10.3389/fcell.2019.00086

[倪劲松 NI Jingsong], 2006, [肿瘤防治研究, Cancer Research on Prevention and Treatment], V33, P311

Obodnikov O, 2017, J THORAC ONCOL, V12, pS1504, DOI 10.1016/j.jtho.2016.11.2069

Pan LL, 2019, EVID-BASED COMPL ALT, V2019, DOI 10.1155/2019/2417418

Pardoll D, 2003, ANNU REV IMMUNOL, V21, P807, DOI 10.1146/annurev.immunol.21.120601.141135

Park D, 2011, ENVIRON TOXICOL PHAR, V31, P397, DOI 10.1016/j.etap.2011.01.008

Park EH, 2014, J GINSENG RES, V38, P22, DOI 10.1016/j.jgr.2013.11.007

Peng Peng, 2007, Chinese Medical Sciences Journal, V22, P177

Peng Z, 2021, FRONT PHARMACOL, V11, DOI 10.3389/fphar.2020.630825

Perl AE, 2019, NEW ENGL J MED, V381, P1728, DOI 10.1056/NEJMoa1902688

Pirker R, 2020, CURR OPIN ONCOL, V32, P63, DOI 10.1097/CCO.0000000000000592

Qiu XM, 2014, ANTI-CANCER DRUG, V25, P1072, DOI 10.1097/CAD.0000000000000147

Richardet E, 2016, J THORAC ONCOL, V11, pS207, DOI 10.1016/j.jtho.2016.08.062

Rijavec E, 2014, FUTURE ONCOL, V10, P79, DOI [10.2217/FON.13.145, 10.2217/fon.13.145]

Rossi A, 2016, EXPERT REV ANTICANC, V16, P653, DOI 10.1586/14737140.2016.1170596

Shan KZ, 2019, BIOL PHARM BULL, V42, P900, DOI 10.1248/bpb.b18-00852

Shao MW, 2014, J INT TRANSL MED, V2, P299

Song JM, 2015, J TRADIT CHIN MED, V35, P440, DOI 10.1016/S0254-6272(15)30122-9

Storz E, 2018, UROLOGE, V57, P532, DOI 10.1007/s00120-018-0606-6

Sun CX, 2016, J EXP CLIN CANC RES, V35, DOI 10.1186/s13046-015-0274-y

Takeda R, 2015, BREAST CANCER-BASIC, V9, DOI 10.4137/BCBCR.S27534

Viallard C, 2017, ANGIOGENESIS, V20, P409, DOI 10.1007/s10456-017-9562-9

[王婵娟 Wang Chanjuan], 2017, [肿瘤, Tumor], V37, P195

Wang JJ, 2018, CANCER LETT, V415, P73, DOI 10.1016/j.canlet.2017.11.037

[王邈 Wang Miao], 2018, [中华中医药杂志, China Journal of Traditional Chinese Medicine and Pharmacy], V33, P2270

Wang XJ, 2019, EUR J PHARMACOL, V850, P141, DOI 10.1016/j.ejphar.2019.02.023

Wang Xin, 2009, Nan Fang Yi Ke Da Xue Xue Bao, V29, P1823

West H, 2019, LANCET ONCOL, V20, P924, DOI 10.1016/S1470-2045(19)30167-6

Wu Xuesong, 2015, J Investig Dermatol Symp Proc, V17, P34, DOI 10.1038/jidsymp.2015.10

Xie QP, 2017, BIOMED PHARMACOTHER, V85, P16, DOI 10.1016/j.biopha.2016.11.096

[熊明华 Xiong Minghua], 2012, [中国现代医学杂志, China Journal of Modern Medicine], V22, P34

Xu TM, 2007, CHINESE MED J-PEKING, V120, P584, DOI 10.1097/00029330-200704010-00011

Xu Y, 2020, EVID-BASED COMPL ALT, V2020, DOI 10.1155/2020/3957193

[杨玉 Yang Yu], 2019, [中国临床药理学杂志, The Chinese Journal of Clinical Pharmacology], V35, P497

Yu CQ, 2020, J MED CHEM, V63, P13397, DOI 10.1021/acs.jmedchem.0c00950

[于同月 Yu Tongyue], 2018, [中国免疫学杂志, Chinese Journal of Immunology], V34, P1012

Yuan ZG, 2017, BIOMED PHARMACOTHER, V89, P227, DOI 10.1016/j.biopha.2017.02.038

[张会来 Zhang Huilai], 2005, [中华放射医学与防护杂志, Chinese Journal of Radiological Medicine and Protection], V25, P431

Zhang Q, 2016, EXPERT REV CLIN PHAR, V9, P143, DOI 10.1586/17512433.2016.1101340

Zhang QY, 2008, CANCER BIOTHER RADIO, V23, P647, DOI 10.1089/cbr.2008.0532

Zhang Yajuan, 2017, Zhongguo Yi Xue Ke Xue Yuan Xue Bao, V39, P150, DOI 10.3881/j.issn.1000-503X.2017.01.025

[张燕 Zhang Yan], 2020, [中国中医基础医学杂志, Chinese Journal of Basic Medicine in Traditional Chinese Medicine], V26, P1649

Zhang ZG, 2017, CANCER LETT, V393, P1, DOI 10.1016/j.canlet.2017.02.008

Zhao XL, 2020, ONCOL REP, V44, P2165, DOI 10.3892/or.2020.7753

Zou Y, 2020, ACS APPL MATER INTER, V12, P14905, DOI 10.1021/acsami.0c01069

NR 81

TC 0

Z9 0

U1 2

U2 24

PU HINDAWI LTD

PI LONDON

PA ADAM HOUSE, 3RD FLR, 1 FITZROY SQ, LONDON, W1T 5HF, ENGLAND

SN 1741-427X

EI 1741-4288

J9 EVID-BASED COMPL ALT

JI Evid.-based Complement Altern. Med.

PD AUG 18

PY 2021

VL 2021

AR 1653750

DI 10.1155/2021/1653750

PG 9

WC Integrative & Complementary Medicine

WE Science Citation Index Expanded (SCI-EXPANDED)

SC Integrative & Complementary Medicine

GA UP6GN

UT WOS:000695476600001

PM 34512772

OA gold, Green Published

DA 2023-04-05

ER

PT J

AU Kim, S

Kim, N

Jeong, J

Lee, S

Kim, W

Ko, SG

Kim, B

AF Kim, Sejin

Kim, Nayeon

Jeong, JaYeon

Lee, Soojin

Kim, Woojin

Ko, Seong-Gyu

Kim, Bonglee

TI Anti-Cancer Effect of Panax Ginseng and Its Metabolites: From

Traditional Medicine to Modern Drug Discovery

SO PROCESSES

LA English

DT Review

DE Panax ginseng; cancer; compound K; ginsenoside Rh2; apoptosis;

angiogenesis; multidrug resistance

ID KOREAN RED GINSENG; EPITHELIAL-MESENCHYMAL TRANSITION; COMPOUND-K;

INTESTINAL BACTERIA; MULTIDRUG-RESISTANCE; APOPTOSIS PATHWAYS; INDUCED

COLITIS; MIXED MICELLES; MCF-7 CELLS; KAPPA-B

AB Cancer incidence and mortality rate are growing worldwide. The effectiveness of cancer therapy depends on the degree of cancer development. Anticancer prevention, screening tests, detection of precancerous conditions or cancers at an early stage of development help to prevent the development of cancer, and in the event of cancer development, they provide the best chance for a full recovery. However, in most cases of advanced cancer, there is no method that can fully cure this disease. Recently, natural products have gained more attention in cancer therapy. Panax ginseng (PG), one of the most popular natural products, is reported to have a wide range of pharmacological activities in cancer. Therefore, the anti-cancer effects and mechanisms of PG and its metabolites (compound K, Ginsenoside Rh1, Rh2, Rh3 and F1) in five major cancers (lung cancer, breast cancer, colon cancer, prostate cancer and stomach cancer) are reviewed in this study. It is confirmed that PG and its metabolites regulated apoptosis, epithelial mesenchymal transition (EMT), angiogenesis, cell cycle arrest and multidrug resistance (MDR) in vitro and in vivo cancer models. In particular, ginsenoside Rh2 showed anticancer effects in all five major cancers. This review could improve the understanding of anticancer mechanisms of PG and its metabolites against major five cancers. Further clinical studies are needed for development anti-cancer drugs using PG and its metabolites.

C1 [Kim, Sejin; Kim, Nayeon; Jeong, JaYeon; Kim, Bonglee] Kyung Hee Univ, Coll Korean Med, 1 Hoegi Dong, Seoul 05253, South Korea.

[Lee, Soojin] Kyung Hee Univ, Grad Sch, Dept Clin Korean Med, 1 Hoegi Dong, Seoul 05253, South Korea.

[Kim, Woojin; Ko, Seong-Gyu; Kim, Bonglee] Kyung Hee Univ, Coll Korean Med, Korean Med Based Drug Repositioning Canc Res Ctr, 1 Hoegi Dong, Seoul 05253, South Korea.

[Kim, Bonglee] Kyung Hee Univ, Dept Pathol, Coll Korean Med, 1 Hoegi Dong, Seoul 130701, South Korea.

C3 Kyung Hee University; Kyung Hee University; Kyung Hee University; Kyung

Hee University

RP Kim, B (通讯作者)，Kyung Hee Univ, Coll Korean Med, 1 Hoegi Dong, Seoul 05253, South Korea.; Kim, B (通讯作者)，Kyung Hee Univ, Coll Korean Med, Korean Med Based Drug Repositioning Canc Res Ctr, 1 Hoegi Dong, Seoul 05253, South Korea.; Kim, B (通讯作者)，Kyung Hee Univ, Dept Pathol, Coll Korean Med, 1 Hoegi Dong, Seoul 130701, South Korea.

EM khkmksj@khu.ac.kr; kimny26@khu.ac.kr; jyjeong28@khu.ac.kr;

lettergr@khu.ac.kr; wjkim@khu.ac.kr; epiko@khu.ac.kr;

bongleekim@khu.ac.kr

RI Kim, Bonglee/AAH-9077-2020

OI Kim, Bonglee/0000-0002-8678-156X; , Sejin/0000-0002-4297-5362; Kim,

Sejin/0000-0001-5649-3172; Kim, Woojin/0000-0001-8494-4524

FU Basic Science Research Program through the National Research Foundation

of Korea (NRF) - Ministry of Education [NRF-2020R1I1A2066868]; National

Research Foundation of Korea (NRF) - Korea government (MSIT)

[2020R1A5A2019413]; Korea Health Technology R&D Project through the

Korea Health Industry Development Institute (KHIDI) - Ministry of Health

& Welfare, Republic of Korea [HF20C0038]

FX This research was supported by Basic Science Research Program through

the National Research Foundation of Korea (NRF) funded by the Ministry

of Education (NRF-2020R1I1A2066868), the National Research Foundation of

Korea (NRF) grant funded by the Korea government (MSIT) (No.

2020R1A5A2019413), a grant of the Korea Health Technology R&D Project

through the Korea Health Industry Development Institute (KHIDI), funded

by the Ministry of Health & Welfare, Republic of Korea (grant number:

HF20C0116), and a grant of the Korea Health Technology R&D Project

through the Korea Health Industry Development Institute (KHIDI), funded

by the Ministry of Health & Welfare, Republic of Korea (grant number:

HF20C0038).

CR Amaravadi RK, 2007, CLIN CANCER RES, V13, P7271, DOI 10.1158/1078-0432.CCR-07-1595

Amaravadi RK, 2007, J CLIN INVEST, V117, P326, DOI 10.1172/JCI28833

Bae EA, 2002, BIOL PHARM BULL, V25, P743, DOI 10.1248/bpb.25.743

Baraya YS, 2017, ANTI-CANCER AGENT ME, V17, P770, DOI 10.2174/1871520616666160817111242

Bilgi N, 2010, ANN PHARMACOTHER, V44, P926, DOI 10.1345/aph.1M715

Bukowczan J, 2015, CURR PHARM DESIGN, V21, P2284, DOI 10.2174/1381612821666150105152553

Chaabane W, 2013, ARCH IMMUNOL THER EX, V61, P43, DOI 10.1007/s00005-012-0205-y

Chen L, 2016, CELL DEATH DIS, V7, DOI 10.1038/cddis.2016.234

Chung C, 2020, J HEMATOL ONCOL, V13, DOI 10.1186/s13045-020-00936-9

Cort A, 2015, NUTR CANCER, V67, P411, DOI 10.1080/01635581.2015.1002624

Coussens LM, 2002, NATURE, V420, P860, DOI 10.1038/nature01322

de Martel C, 2012, LANCET ONCOL, V13, DOI 10.1016/S1470-2045(12)70137-7

Debatin KM, 2004, CANCER IMMUNOL IMMUN, V53, P153, DOI 10.1007/s00262-003-0474-8

DONNELLY S, 1995, SEMIN ONCOL, V22, P67

Frascarelli S, 2003, BASIC RES CARDIOL, V98, P401, DOI 10.1007/s00395-003-0434-7

Gan T, 2019, J AM COLL SURGEONS, V228, P342, DOI 10.1016/j.jamcollsurg.2018.12.035

Gao QR, 2018, CELL PROLIFERAT, V51, DOI 10.1111/cpr.12438

Ge GQ, 2017, BIOL PHARM BULL, V40, P2117, DOI 10.1248/bpb.b17-00463

Ghobrial IM, 2005, CA-CANCER J CLIN, V55, P178, DOI 10.3322/canjclin.55.3.178

Han S, 2016, J ETHNOPHARMACOL, V194, P83, DOI 10.1016/j.jep.2016.08.039

Hasegawa H, 1996, PLANTA MED, V62, P453, DOI 10.1055/s-2006-957938

Hasegawa H, 2004, J PHARMACOL SCI, V95, P153, DOI 10.1254/jphs.FMJ04001X4

Hasegawa H, 2002, BIOL PHARM BULL, V25, P861, DOI 10.1248/bpb.25.861

Hasegawa H, 2000, BIOL PHARM BULL, V23, P298, DOI 10.1248/bpb.23.298

Hou JG, 2019, INT J MOL SCI, V20, DOI 10.3390/ijms20051244

Huang YQ, 2019, J NANOSCI NANOTECHNO, V19, P1942, DOI 10.1166/jnn.2019.16404

Hwang IH, 2016, AM J CHINESE MED, V44, P1081, DOI 10.1142/S0192415X16500609

Hwang JW, 2015, MOL MED REP, V11, P670, DOI 10.3892/mmr.2014.2704

Jiang SL, 2017, CHIN J INTEGR MED, V23, P331, DOI 10.1007/s11655-015-2146-x

Jin X, 2018, INT J NANOMED, V13, P3827, DOI 10.2147/IJN.S167529

Kawaguchi M, 2015, CANCER SCI, V106, P1130, DOI 10.1111/cas.12725

Kee JY, 2018, J ETHNOPHARMACOL, V220, P177, DOI 10.1016/j.jep.2018.03.036

Kent LN, 2019, NAT REV CANCER, V19, P326, DOI 10.1038/s41568-019-0143-7

Kim EJ, 2018, J GINSENG RES, V42, P288, DOI 10.1016/j.jgr.2017.03.008

Kim HS, 2017, NUTRIENTS, V9, DOI 10.3390/nu9070772

Kim JK, 2016, ORIENTAL PHARM EXP M, V16, P243, DOI 10.1007/s13596-016-0246-6

Kim JH, 2017, J GINSENG RES, V41, P435, DOI 10.1016/j.jgr.2016.08.004

Kim JH, 2018, J GINSENG RES, V42, P264, DOI 10.1016/j.jgr.2017.10.004

Kojima M, 1999, NATURE, V402, P656, DOI 10.1038/45230

Kwak CW, 2015, J MICROBIOL BIOTECHN, V25, P1170, DOI 10.4014/jmb.1505.05057

Kwak JH, 2014, BIOORG MED CHEM LETT, V24, P5409, DOI 10.1016/j.bmcl.2014.10.041

Kwekkeboom KL, 2016, SEMIN ONCOL NURS, V32, P373, DOI 10.1016/j.soncn.2016.08.004

Lee DG, 2016, CHIN J INTEGR MED, V22, P344, DOI 10.1007/s11655-014-1789-8

Lee H, 2018, J GINSENG RES, V42, P455, DOI 10.1016/j.jgr.2017.05.003

Li T, 2018, ONCOL LETT, V16, P4640, DOI 10.3892/ol.2018.9192

Li X, 2020, EUR J MED CHEM, V203, DOI 10.1016/j.ejmech.2020.112627

Li Y, 2015, J THORAC DIS, V7, P400, DOI 10.3978/j.issn.2072-1439.2015.01.03

Liu GW, 2018, HUM CELL, V31, P189, DOI 10.1007/s13577-017-0189-3

Liu HF, 2009, DRUG METAB DISPOS, V37, P2290, DOI 10.1124/dmd.109.029819

Ma J, 2019, EXP THER MED, V18, P630, DOI 10.3892/etm.2019.7604

Maduzia D, 2015, J PHYSIOL PHARMACOL, V66, P875

Matuszyk A, 2016, INT J MOL SCI, V17, DOI 10.3390/ijms17091455

Miller KD, 2019, CA-CANCER J CLIN, V69, P363, DOI 10.3322/caac.21565

Myers AP, 2015, PHARMACOTHERAPY, V35, pE9, DOI 10.1002/phar.1550

Oh JK, 2014, ANN GLOB HEALTH, V80, P384, DOI 10.1016/j.aogh.2014.09.013

Oh J, 2019, J GINSENG RES, V43, P421, DOI 10.1016/j.jgr.2018.05.004

Oh J, 2015, J MED FOOD, V18, P421, DOI 10.1089/jmf.2014.3248

Paci E, 2017, THORAX, V72, P825, DOI 10.1136/thoraxjnl-2016-209825

Qi LW, 2011, CURR DRUG METAB, V12, P818, DOI 10.2174/138920011797470128

Qian Jun, 2016, Asian Pac J Cancer Prev, V17, P1817

Rajabian A, 2019, EXPERT OPIN THER PAT, V29, P55, DOI 10.1080/13543776.2019.1556258

Ren GX, 2018, MOLECULES, V23, DOI 10.3390/molecules23112908

Rubio-Patino C, 2018, CELL METAB, V27, P828, DOI 10.1016/j.cmet.2018.02.009

Schabath MB, 2019, CANCER EPIDEM BIOMAR, V28, P1563, DOI 10.1158/1055-9965.EPI-19-0221

Schirrmacher V, 2019, INT J ONCOL, V54, P407, DOI 10.3892/ijo.2018.4661

Shacter E, 2002, ONCOLOGY-NY, V16, P217

SHIBATA S, 1963, CHEM PHARM BULL, V11, P759

Shin BK, 2015, J GINSENG RES, V39, P287, DOI 10.1016/j.jgr.2014.12.005

Shin HJ, 2020, J TRADIT COMPL MED, V10, P198, DOI 10.1016/j.jtcme.2020.04.004

Shin HR, 2000, CANCER CAUSE CONTROL, V11, P565, DOI 10.1023/A:1008980200583

Slattery ML, 2018, APOPTOSIS, V23, P237, DOI 10.1007/s10495-018-1451-1

Smith BN, 2016, J CLIN MED, V5, DOI 10.3390/jcm5020017

Song BK, 2017, J MICROBIOL BIOTECHN, V27, P1233, DOI 10.4014/jmb.1701.01077

Stempniewicz A, 2019, INT J MOL SCI, V20, DOI 10.3390/ijms20071534

Sung H, 2021, CA-CANCER J CLIN, V71, P209, DOI [10.3322/caac.21492, 10.3322/caac.21660, 10.3322/caac.21442]

Takeda R, 2006, J AM SOC NEPHROL, V17, P113, DOI 10.1681/ASN.2004080626

TAKINO Y, 1994, YAKUGAKU ZASSHI, V114, P550, DOI 10.1248/yakushi1947.114.8_550

Terzic J, 2010, GASTROENTEROLOGY, V138, P2101, DOI 10.1053/j.gastro.2010.01.058

Tischlerova V, 2017, WORLD J GASTROENTERO, V23, P4341, DOI 10.3748/wjg.v23.i24.4341

Tong-Lin Wu Tony, 2018, Oncotarget, V9, P11109, DOI 10.18632/oncotarget.24326

Torre LA, 2016, CANCER EPIDEM BIOMAR, V25, P16, DOI 10.1158/1055-9965.EPI-15-0578

Uemura N, 2001, NEW ENGL J MED, V345, P784, DOI 10.1056/NEJMoa001999

Vayghan HJ, 2014, ASIAN PAC J CANCER P, V15, P585, DOI 10.7314/APJCP.2014.15.2.585

Walsh D, 2000, SUPPORT CARE CANCER, V8, P175, DOI 10.1007/s005200050281

Wang YC, 2018, EXP THER MED, V15, P4916, DOI 10.3892/etm.2018.6067

Warzecha Z, 2014, J PHYSIOL PHARMACOL, V65, P95

Warzecha Z, 2013, J PHYSIOL PHARMACOL, V64, P657

Warzecha Z, 2012, MED SCI MONITOR, V18, pBR181, DOI 10.12659/MSM.882727

Wen Xu, 2015, Asian Pac J Cancer Prev, V16, P1105

Wild C.P., 2020, WORLD CANC REPORT CA

Xiao H, 2019, AM J CHINESE MED, V47, P1737, DOI 10.1142/S0192415X19500885

Yan ZK, 2017, COMB CHEM HIGH T SCR, V20, P423, DOI 10.2174/1386207320666170116141818

Yang JJ, 2016, J GINSENG RES, V40, P400, DOI 10.1016/j.jgr.2016.03.007

Yang L, 2017, INT J NANOMED, V12, P7653, DOI 10.2147/IJN.S144305

Yao HQ, 2018, ONCOL LETT, V15, P8339, DOI 10.3892/ol.2018.8414

Yoo HS, 2017, ONCOL REP, V37, P3287, DOI 10.3892/or.2017.5590

Yu JS, 2018, J GINSENG RES, V42, P562, DOI 10.1016/j.jgr.2018.02.004

Yu K, 2012, CURR DRUG METAB, V13, P577, DOI 10.2174/1389200211209050577

Zhang H, 2015, INT J CLIN EXP PATHO, V8, P4444

Zhang KQ, 2016, PHARM BIOL, V54, P561, DOI 10.3109/13880209.2015.1101142

Zhang QC, 2015, TUMOR BIOL, V36, P2377, DOI 10.1007/s13277-014-2845-5

Zhu CL, 2016, ARCH IRAN MED, V19, P23, DOI 0161901/AIM.005

NR 102

TC 3

Z9 3

U1 3

U2 20

PU MDPI

PI BASEL

PA ST ALBAN-ANLAGE 66, CH-4052 BASEL, SWITZERLAND

EI 2227-9717

J9 PROCESSES

JI Processes

PD AUG

PY 2021

VL 9

IS 8

AR 1344

DI 10.3390/pr9081344

PG 21

WC Engineering, Chemical

WE Science Citation Index Expanded (SCI-EXPANDED)

SC Engineering

GA UH3QZ

UT WOS:000689851300001

OA gold

DA 2023-04-05

ER

PT J

AU Luo, X

Wang, H

Ji, DG

AF Luo, Xiao

Wang, Hui

Ji, Degang

TI Carbon nanotubes (CNT)-loaded ginsenosides Rb3 suppresses the PD-1/PD-L1

pathway in triple-negative breast cancer

SO AGING-US

LA English

DT Article

DE triple-negative breast cancer; progression; CNTs; Rg3; PD-1/PD-L1 axis

ID IMMUNE CHECKPOINT BLOCKADE; PD-L1; CYTOTOXICITY; EXPRESSION

AB Carbon nanotubes (CNTs), as advanced nanotechnology with specific properties and structures, have presented practical drug delivery properties. Ginsenoside Rg3 is a component of puffed ginseng and demonstrates anticancer activities. To explore the effect of CNTs-loaded Rg3 (Rg3-CNT) on the PD-1/PD-L1 signaling and the development of triple-negative breast cancer (TNBC). Our data revealed that Rg3 inhibited the cell viability of TNBC cells, in which Rg3-CNT further enhanced this effect in the system. Similarly, the colony formation of TNBC cells was decreased by Rg3, while Rg3-CNT could reinforce its effect in the cells. Besides, the treatment of Rg3 induced apoptosis of TNBC cells, in which Rg3-CNT treatment further increased the phenotype in the cells. Remarkably, Rg3-CNT, but not Rg3, attenuated PD-L1 expression in TNBC cells. Rg3-CNT decreased the PD-L1 upregulation induced by interferon-gamma (IFN-gamma) in breast cancer cells. Importantly, Rg3-CNT was able to reduce PD-1 expression in activated T cells. Specifically, Rg3-CNT reduced the PD-1/PD-L1 axis in a T cell/triple-negative TNBC cell co-culture system. Moreover, the levels of IFN-gamma, interleukins-2 (IL-2), interleukins-9 (IL-9), interleukins-10 (IL-10), interleukins-22 (IL-22), and interleukins-23 (IL-23) were significantly stimulated in the activated T cells, while the treatment of Rg3-CNT could reverse these phenotypes in the cells. Rg3-CNT attenuated the TNBC cell growth in vivo. The Rg3-CNT improved the anti-cancer effect of Rg3 toward TNBC by inhibiting the PD-1/PD-L1 axis. Our finding provides new insights into the mechanism by which Rg3-CNT attenuates the development of TNBC. Rg3-CNT may be applied as the potential therapeutic strategy for immunotherapy of TNBC.

C1 [Luo, Xiao] Jilin Univ, Dept Breast Surg, China Japan Union Hosp, Changchun 130033, Jilin, Peoples R China.

[Wang, Hui] Jilin Univ, Dept Ultrasound, China Japan Union Hosp, Changchun 130033, Jilin, Peoples R China.

[Ji, Degang] Jilin Univ, Dept Hepatobiliary Pancreat Surg, China Japan Union Hosp, Changchun 130033, Jilin, Peoples R China.

C3 Jilin University; Jilin University; Jilin University

RP Wang, H (通讯作者)，Jilin Univ, Dept Ultrasound, China Japan Union Hosp, Changchun 130033, Jilin, Peoples R China.; Ji, DG (通讯作者)，Jilin Univ, Dept Hepatobiliary Pancreat Surg, China Japan Union Hosp, Changchun 130033, Jilin, Peoples R China.

EM whui66@jlu.edu.cn; Jdg@jlu.edu.cn

CR Abdullah A, 2021, ONCOGENE, V40, P322, DOI 10.1038/s41388-020-01530-6

Alshehri R, 2016, J MED CHEM, V59, P8149, DOI 10.1021/acs.jmedchem.5b01770

Andrieu GP, 2019, CANCER LETT, V465, P45, DOI 10.1016/j.canlet.2019.08.013

Bates JP, 2018, BMC CANCER, V18, DOI 10.1186/s12885-018-4441-3

Cao Y, 2019, TOXICOL APPL PHARM, V385, DOI 10.1016/j.taap.2019.114801

Chen XP, 2011, INT J CLIN ONCOL, V16, P519, DOI 10.1007/s10147-011-0222-6

Emens LA, 2018, CLIN CANCER RES, V24, P511, DOI 10.1158/1078-0432.CCR-16-3001

Guo Q, 2017, J HUAZHONG U SCI-MED, V37, P635, DOI 10.1007/s11596-017-1783-z

Hartkopf AD, 2016, BREAST CARE, V11, P385, DOI 10.1159/000453569

Hong SZ, 2020, ONCOL REP, V44, P1333, DOI 10.3892/or.2020.7728

Jiang ZS, 2017, BIOMED PHARMACOTHER, V96, P378, DOI 10.1016/j.biopha.2017.09.129

Jing X, 2020, EXP CELL RES, V392, DOI 10.1016/j.yexcr.2020.112034

Lahiani MH, 2017, NANOTECHNOLOGY, V28, DOI 10.1088/0957-4484/28/1/015101

Liu TH, 2019, BIOMED PHARMACOTHER, V120, DOI 10.1016/j.biopha.2019.109483

Mall C, 2016, ONCOIMMUNOLOGY, V5, DOI 10.1080/2162402X.2015.1075114

Mao Z, 2020, SMALL, V16, DOI 10.1002/smll.201904047

Melita ED, 2015, ROM J MORPHOL EMBRYO, V56, P349

Mir M, 2017, NANOSCALE RES LETT, V12, DOI 10.1186/s11671-017-2249-8

Oh J, 2019, J GINSENG RES, V43, P421, DOI 10.1016/j.jgr.2018.05.004

Ozgen PSO, 2020, J MATER CHEM B, V8, P3123, DOI 10.1039/c9tb02711d

Rodgers RJ, 2017, HUM REPROD, V32, P1033, DOI 10.1093/humrep/dex027

Romano-Feinholz S, 2017, INT J NANOMED, V12, P6005, DOI 10.2147/IJN.S139004

Saleh R, 2020, GENES-BASEL, V11, DOI 10.3390/genes11060703

Santoni M, 2018, BBA-REV CANCER, V1869, P78, DOI 10.1016/j.bbcan.2017.10.007

Sheikhpour M, 2017, MAT SCI ENG C-MATER, V76, P1289, DOI 10.1016/j.msec.2017.02.132

Son KH, 2016, INT J NANOMED, V11, P5163, DOI 10.2147/IJN.S112660

Song JH, 2020, PLOS ONE, V15, DOI 10.1371/journal.pone.0240533

Tang W, 2019, CHEM SOC REV, V48, P2967, DOI 10.1039/c8cs00805a

Topalian SL, 2016, NAT REV CANCER, V16, P275, DOI 10.1038/nrc.2016.36

Tray N, 2019, CANCER TREAT REV, V79, DOI 10.1016/j.ctrv.2019.08.004

van der Vlist M, 2016, NAT REV RHEUMATOL, V12, P593, DOI 10.1038/nrrheum.2016.131

Vinay DS, 2015, SEMIN CANCER BIOL, V35, pS185, DOI 10.1016/j.semcancer.2015.03.004

Wang YK, 2014, PLOS ONE, V9, DOI 10.1371/journal.pone.0104209

Woolston C, 2015, NATURE, V527, pS101, DOI 10.1038/527S101a

Wu WS, 2018, J MED FOOD, V21, P849, DOI 10.1089/jmf.2017.4144

Yuan ZG, 2017, BIOMED PHARMACOTHER, V89, P227, DOI 10.1016/j.biopha.2017.02.038

Zhang R, 2019, SMALL, V15, DOI 10.1002/smll.201903881

Zhang Y, 2016, ASIAN PAC J TROP MED, V9, P178, DOI 10.1016/j.apjtm.2016.01.010

Zhao LQ, 2019, ONCOL REP, V41, P3209, DOI 10.3892/or.2019.7115

Zou JF, 2020, J BIOCHEM MOL TOXIC, V34, DOI 10.1002/jbt.22480

NR 40

TC 11

Z9 11

U1 5

U2 21

PU IMPACT JOURNALS LLC

PI ORCHARD PARK

PA 6666 E QUAKER ST, STE 1, ORCHARD PARK, NY 14127 USA

SN 1945-4589

J9 AGING-US

JI Aging-US

PD JUL 15

PY 2021

VL 13

IS 13

BP 17177

EP 17189

PG 13

WC Cell Biology; Geriatrics & Gerontology

WE Science Citation Index Expanded (SCI-EXPANDED)

SC Cell Biology; Geriatrics & Gerontology

GA TP3KG

UT WOS:000677491700017

PM 34111025

OA Green Published, gold

DA 2023-04-05

ER

PT J

AU Jeon, H

Jin, Y

Myung, CS

Heo, KS

AF Jeon, Hyesu

Jin, Yujin

Myung, Chang-Seon

Heo, Kyung-Sun

TI Ginsenoside-Rg2 exerts anti-cancer effects through ROS-mediated AMPK

activation associated mitochondrial damage and oxidation in MCF-7 cells

SO ARCHIVES OF PHARMACAL RESEARCH

LA English

DT Article

DE AMPK; Breast cancer; Cell cycle; Ginsenoside-Rg2; Reactive oxygen

species; Mitochondrial membrane potential

ID BREAST-CANCER CELLS; APOPTOSIS; INDUCTION; STRESS; PROLIFERATION;

PGC-1-ALPHA; METABOLISM; CISPLATIN; GROWTH

AB In this study, we investigated the anti-cancer effects of ginsenoside Rg2 (G-Rg2) and its underlying signaling pathways in breast cancer (BC) cells. G-Rg2 significantly induced cytotoxicity and reactive oxygen species (ROS) production in MCF-7 cells among various types of BC cells including HCC1428, T47D, and BT-549. G-Rg2 significantly inhibited protein and mRNA expression of cell cycle G1-S phase regulators, including p-Rb, cyclin D1, CDK4, and CDK6, whereas it enhanced the protein and mRNA expression of cell cycle arrest and apoptotic molecules including cleaved PARP, p21, p27, p53 and Bak through ROS production. These effects were abrogated by the antioxidant N-acetyl-I-cysteine, or NADPH oxidase inhibitors, such as diphenyleneiodonium chloride and apocynin. Interestingly, G-Rg2 induced mitochondrial damage by reducing the membrane potential. G-Rg2 further activated the ROS-sensor protein, AMPK and downstream targets of AMPK activation, including PGC-1 alpha, FOXO1, and IDH2, and downregulated mTOR activation and antioxidant response element-driven luciferase activity. Together, our data demonstrate that G-Rg2 mediates anti-cancer effects by activating cell cycle arrest and signaling pathways related to mitochondrial damage-induced ROS production and apoptosis.

C1 [Jeon, Hyesu; Jin, Yujin; Myung, Chang-Seon; Heo, Kyung-Sun] Chungnam Natl Univ, Coll Pharm, 99 Daehak Ro, Daejeon, South Korea.

C3 Chungnam National University

RP Heo, KS (通讯作者)，Chungnam Natl Univ, Coll Pharm, 99 Daehak Ro, Daejeon, South Korea.

EM kheo@cnu.ac.kr

OI Heo, Kyung-Sun/0000-0003-3800-7665

FU National Research Foundation of Korea [KNRF-2019R1C1C100733112]

FX This research was supported by National Research Foundation of Korea

(KNRF-2019R1C1C100733112).

CR Ahn J, 2020, ARCH PHARM RES, V43, P735, DOI 10.1007/s12272-020-01254-x

Azamjah Nasrindokht, 2019, Asian Pac J Cancer Prev, V20, P2015, DOI 10.31557/APJCP.2019.20.7.2015

Bak MJ, 2014, INT J MOL MED, V34, P1516, DOI 10.3892/ijmm.2014.1972

Bost F, 2019, AM J CANCER RES, V9, P198

Chaube B, 2015, CELL DEATH DISCOV, V1, DOI 10.1038/cddiscovery.2015.63

Cui J, 2017, CHEM-BIOL INTERACT, V275, P152, DOI 10.1016/j.cbi.2017.07.021

Cui L, 2018, ARCH PHARM RES, V41, P299, DOI 10.1007/s12272-017-0990-2

Dai XF, 2017, J CANCER, V8, P3131, DOI 10.7150/jca.18457

Duarte FV, 2015, CURR MED CHEM, V22, P2468, DOI 10.2174/0929867322666150514095910

Gou DX, 2020, J GINSENG RES, V44, P717, DOI 10.1016/j.jgr.2019.06.005

Green DR, 1998, SCIENCE, V281, P1309, DOI 10.1126/science.281.5381.1309

Gross DN, 2008, ONCOGENE, V27, P2320, DOI 10.1038/onc.2008.25

Guo CY, 2013, NEURAL REGEN RES, V8, P2003, DOI 10.3969/j.issn.1673-5374.2013.21.009

Huynh DTN, 2021, CANCERS, V13, DOI 10.3390/cancers13081892

Huynh DTN, 2020, INT J MOL SCI, V21, DOI 10.3390/ijms21186656

Jeon H, 2021, PHYTOMEDICINE, V85, DOI 10.1016/j.phymed.2021.153549

Jiang SS, 2019, J CELL MOL MED, V23, P3951, DOI 10.1111/jcmm.14279

Jin Y, 2020, ARCH PHARM RES, V43, P773, DOI [10.1007/s12272-020-01265-8, 10.1007/s12272-020-01255-w]

Jin Y, 2019, BMB REP, V52, P706, DOI 10.5483/BMBRep.2019.52.12.234

Kang SW, 2015, ARCH PHARM RES, V38, P338, DOI 10.1007/s12272-015-0550-6

Kim MY, 2018, BIOL PHARM BULL, V41, P713, DOI 10.1248/bpb.b17-00898

Kong MJ, 2018, CELL DEATH DIS, V9, DOI 10.1038/s41419-018-0537-6

Lee YJ, 2019, ARCH PHARM RES, V42, P890, DOI 10.1007/s12272-019-01181-6

Li XJ, 2019, PHYTOMEDICINE, V53, P124, DOI 10.1016/j.phymed.2018.09.033

Liu HM, 2019, FOOD FUNCT, V10, P3603, DOI 10.1039/c9fo00027e

Nguyen TL, 2021, ARCH PHARM RES, V44, P241, DOI 10.1007/s12272-020-01304-4

Pena-Blanco A, 2018, FEBS J, V285, P416, DOI 10.1111/febs.14186

Pluchino LA, 2016, CANCER LETT, V381, P124, DOI 10.1016/j.canlet.2016.07.036

Pordeli M, 2017, BREAST CANCER-TOKYO, V24, P299, DOI 10.1007/s12282-016-0704-5

Radde BN, 2015, BIOCHEM J, V465, P49, DOI 10.1042/BJ20131608

Shao C, 2020, REDOX BIOL, V36, DOI 10.1016/j.redox.2020.101685

Shin MK, 2019, ARCH PHARM RES, V42, P113, DOI 10.1007/s12272-019-01127-y

Skonieczna M, 2017, OXID MED CELL LONGEV, V2017, DOI 10.1155/2017/9420539

Tan ZQ, 2016, MOL CANCER THER, V15, P774, DOI 10.1158/1535-7163.MCT-15-0621

Wallace DC, 2012, NAT REV CANCER, V12, P685, DOI 10.1038/nrc3365

Yi B, 2013, ONCOL LETT, V6, P583, DOI 10.3892/ol.2013.1403

Zhang HS, 2019, J CELL MOL MED, V23, P3451, DOI 10.1111/jcmm.14241

Zhang HS, 2018, INT J BIOCHEM CELL B, V95, P85, DOI 10.1016/j.biocel.2017.12.016

Zhang Y, 2007, CELL RES, V17, P363, DOI 10.1038/cr.2007.11

Zhao Y, 2017, MOL CANCER, V16, DOI 10.1186/s12943-017-0648-1

NR 40

TC 16

Z9 16

U1 2

U2 12

PU PHARMACEUTICAL SOC KOREA

PI SEOUL

PA 1489-3 SUHCHO-DONG, SUHCHO-KU, SEOUL 137-071, SOUTH KOREA

SN 0253-6269

EI 1976-3786

J9 ARCH PHARM RES

JI Arch. Pharm. Res.

PD JUL

PY 2021

VL 44

IS 7

BP 702

EP 712

DI 10.1007/s12272-021-01345-3

EA JUL 2021

PG 11

WC Chemistry, Medicinal; Pharmacology & Pharmacy

WE Science Citation Index Expanded (SCI-EXPANDED)

SC Pharmacology & Pharmacy

GA TQ9EI

UT WOS:000677252800002

PM 34302638

DA 2023-04-05

ER

PT J

AU Nakhjavani, M

Smith, E

Palethorpe, HM

Tomita, Y

Yeo, K

Price, TJ

Townsend, AR

Hardingham, JE

AF Nakhjavani, Maryam

Smith, Eric

Palethorpe, Helen M.

Tomita, Yoko

Yeo, Kenny

Price, Tim J.

Townsend, Amanda R.

Hardingham, Jennifer E.

TI Anti-Cancer Effects of an Optimised Combination of Ginsenoside Rg3

Epimers on Triple Negative Breast Cancer Models

SO PHARMACEUTICALS

LA English

DT Article

DE ginsenoside Rg3; Epimer; triple negative breast cancer; metastasis;

response surface methodology; nod scid gamma mice

ID RSK FAMILY; STEM-CELLS; PHOSPHORYLATION; PROLIFERATION; SUPPRESSES;

RESISTANCE; INHIBITOR; SURVIVAL; RECEPTOR; PATHWAY

AB Key problems of chemotherapies, as the mainstay of treatment for triple-negative breast cancer (TNBC), are toxicity and development of tumour resistance. Using response surface methodology, we previously optimised the combination of epimers of ginsenoside Rg3 (Rg3) for anti-angiogenic action. Here, we show that the optimised combination of 50 mu M SRg3 and 25 mu M RRg3 (C3), derived from an RSM model of migration of TNBC cell line MDA-MB-231, inhibited migration of MDA-MB-231 and HCC1143, in 2D and 3D migration assays (p < 0.0001). C3 inhibited mammosphere formation efficiency in both cell lines and decreased the CD44(+) stem cell marker in the mammospheres. Molecular docking predicted that Rg3 epimers had a better binding score with IGF-1R than with EGFR, HER-2 or PDGFR, and predicted an mTOR inhibitory function of Rg3. C3 affected the signalling of AKT in MDA-MB-231 and HCC1143 mammospheres. In a mouse model of metastatic TNBC, an equivalent dose of C3 (23 mg/kg SRg3 + 11 mg/kg RRg3) or an escalated dose of 46 mg/kg SRg3 + 23 mg/kg RRg3 was administered to NSG mice bearing MDA-MB-231-Luc cells. Calliper and IVIS spectrum measurement of the primary and secondary tumour showed that the treatment shrunk the primary tumour and decreased the load of metastasis in mice. In conclusion, this combination of Rg3 epimers showed promising results as a potential treatment option for TNBC patients.

C1 [Nakhjavani, Maryam; Smith, Eric; Tomita, Yoko; Yeo, Kenny; Hardingham, Jennifer E.] Queen Elizabeth Hosp, Mol Oncol, Basil Hetzel Inst, Woodville South, SA 5011, Australia.

[Nakhjavani, Maryam; Smith, Eric; Tomita, Yoko; Yeo, Kenny; Price, Tim J.; Townsend, Amanda R.; Hardingham, Jennifer E.] Univ Adelaide, Adelaide Med Sch, Adelaide, SA 5005, Australia.

[Palethorpe, Helen M.] Univ South Australia, Ctr Canc Biol, Adelaide, SA 5000, Australia.

[Palethorpe, Helen M.] SA Pathol, Adelaide, SA 5000, Australia.

[Tomita, Yoko; Price, Tim J.; Townsend, Amanda R.] Queen Elizabeth Hosp, Oncol Unit, Woodville South, SA 5011, Australia.

C3 University of Adelaide; Centre for Cancer Biology; University of South

Australia; SA Pathology

RP Smith, E (通讯作者)，Queen Elizabeth Hosp, Mol Oncol, Basil Hetzel Inst, Woodville South, SA 5011, Australia.; Smith, E (通讯作者)，Univ Adelaide, Adelaide Med Sch, Adelaide, SA 5005, Australia.

EM maryam.nakhjavani@adelaide.edu.au; eric.smith@adelaide.edu.au;

helen.palethorpe@unisa.edu.au; yoko.tomita@adelaide.edu.au;

a1811332@student.adelaide.edu.au; timothy.price@sa.gov.au;

amanda.townsend@sa.gov.au; jennifer.hardingham@adelaide.edu.au

RI Palethorpe, Helen Marie/AAC-4219-2022

OI Palethorpe, Helen Marie/0000-0003-3803-5113; Hardingham,

Jennifer/0000-0001-8277-1199; Smith, Eric/0000-0003-2958-3492; Tomita,

Yoko/0000-0001-7934-2203; Yeo, Kenny/0000-0001-5733-0463

FU Margaret Elcombe Hospital Research Foundation Research Grant

FX This work was kindly supported by the Margaret Elcombe Hospital Research

Foundation Research Grant.

CR Aboushady D, 2020, PHARMACEUTICALS-BASE, V13, DOI 10.3390/ph13110364

Abraham BK, 2005, CLIN CANCER RES, V11, P1154

Anjum R, 2008, NAT REV MOL CELL BIO, V9, P747, DOI 10.1038/nrm2509

Bahhnassy A, 2015, MOL MED REP, V12, P851, DOI 10.3892/mmr.2015.3560

Biswas T, 2017, ONCOTARGET, V8, P112712, DOI 10.18632/oncotarget.22521

Cardillo TM, 2013, BMC CANCER, V13, DOI 10.1186/1471-2407-13-170

Chen BJ, 2015, J EXP CLIN CANC RES, V34, DOI 10.1186/s13046-015-0239-1

Chen JZ, 2015, CLIN EXP PHARMACOL P, V42, P1317, DOI 10.1111/1440-1681.12493

Choi S, 2009, PHARM RES-DORDR, V26, P2280, DOI 10.1007/s11095-009-9944-9

Chui CH, 2006, ONCOL REP, V16, P1313

Garrido-Castro AC, 2019, CANCER DISCOV, V9, P176, DOI 10.1158/2159-8290.CD-18-1177

Giatromanolaki A, 2011, MED ONCOL, V28, P745, DOI 10.1007/s12032-010-9530-3

Grimshaw MJ, 2008, BREAST CANCER RES, V10, DOI 10.1186/bcr2106

Groza IM, 2020, CANCERS, V12, DOI 10.3390/cancers12103053

Guerrero-Zotano A, 2016, CANCER METAST REV, V35, P515, DOI 10.1007/s10555-016-9637-x

Guo L, 2007, INT J BIOL MARKER, V22, P1

Hartman NW, 2013, CELL REP, V5, P433, DOI 10.1016/j.celrep.2013.09.017

Hekman M, 2005, FEBS LETT, V579, P464, DOI 10.1016/j.febslet.2004.11.105

Herbert SP, 2019, ESSAYS BIOCHEM, V63, P595, DOI 10.1042/EBC20190009

Hiraga T, 2011, ONCOL REP, V25, P289, DOI 10.3892/or_00001073

Hu J, 2017, CELL DEATH DIS, V8, DOI 10.1038/cddis.2017.72

Ji P, 2016, ONCOL REP, V35, P3293, DOI 10.3892/or.2016.4739

Kwak JH, 2014, BIOORG MED CHEM LETT, V24, P5409, DOI 10.1016/j.bmcl.2014.10.041

Lamhamedi-Cherradi SE, 2016, JNCI-J NATL CANCER I, V108, DOI 10.1093/jnci/djw182

Li WZ, 2017, SCI REP-UK, V7, DOI 10.1038/s41598-017-14364-2

Li XL, 2018, LIFE SCI, V196, P56, DOI 10.1016/j.lfs.2018.01.014

Lombardo Y, 2015, JOVE-J VIS EXP, DOI 10.3791/52671

Lu CW, 2016, EXPERT REV MOL MED, V18, DOI 10.1017/erm.2015.20

Lu YZ, 2014, ARCH MED SCI, V10, P149, DOI 10.5114/aoms.2013.36927

Ludwik KA, 2016, MOL CANCER THER, V15, P2598, DOI 10.1158/1535-7163.MCT-16-0106

Masuda H, 2012, BREAST CANCER RES TR, V136, P331, DOI 10.1007/s10549-012-2289-9

Mollard S, 2016, SCI REP-UK, V6, DOI 10.1038/srep36173

Nakhjavani M, 2021, CANCERS, V13, DOI 10.3390/cancers13092223

Nakhjavani M, 2020, MOLECULES, V25, DOI 10.3390/molecules25214905

Nakhjavani M, 2019, J BREAST CANCER, V22, P341, DOI 10.4048/jbc.2019.22.e39

Nakhjavani M, 2019, PHARMACEUTICALS-BASE, V12, DOI 10.3390/ph12030117

Nakhjavani Maryam, 2019, Medicines (Basel), V6, DOI 10.3390/medicines6010017

Oh J, 2019, J GINSENG RES, V43, P421, DOI 10.1016/j.jgr.2018.05.004

Paltoglou S, 2017, CANCER RES, V77, P3417, DOI 10.1158/0008-5472.CAN-16-1616

Peng B, 2019, PHARMACOL RES, V142, P1, DOI 10.1016/j.phrs.2019.02.003

Phuc PV, 2011, ONCOTARGETS THER, V4, P71, DOI 10.2147/OTT.S21431

Pons B, 2011, INT J ONCOL, V39, P1337, DOI 10.3892/ijo.2011.1118

Romeo Y, 2012, BIOCHEM J, V441, P553, DOI 10.1042/BJ20110289

Rutkovsky AC, 2019, BMC CANCER, V19, DOI 10.1186/s12885-019-5667-4

Ruvinsky I, 2005, GENE DEV, V19, P2199, DOI 10.1101/gad.351605

Sheridan C, 2006, BREAST CANCER RES, V8, DOI 10.1186/bcr1610

Shipitsin M, 2007, CANCER CELL, V11, P259, DOI 10.1016/j.ccr.2007.01.013

Shipitsin M, 2014, PROTEOME SCI, V12, DOI 10.1186/1477-5956-12-40

Smith E, 2018, CELLS-BASEL, V7, DOI 10.3390/cells7070081

Toyama T, 2008, BMC CANCER, V8, DOI 10.1186/1471-2407-8-309

Willett M, 2011, BIOCHEM J, V438, P217, DOI 10.1042/BJ20110435

Wu CW, 2012, J EXP BIOL, V215, P1720, DOI 10.1242/jeb.066225

Xia P, 2015, AM J CANCER RES, V5, P1602

Xiao L, 2009, J EXP CLIN CANC RES, V28, DOI 10.1186/1756-9966-28-152

Xu HX, 2015, ONCOTARGETS THER, V8, P3783, DOI 10.2147/OTT.S95470

Yousefnia Saghar, 2019, Tumour Biol, V41, p1010428319869101, DOI 10.1177/1010428319869101

Zhao HS, 2016, ANTICANCER RES, V36, P1267

Zhao W, 2014, J BIOMOL SCREEN, V19, P817, DOI 10.1177/1087057114521867

Zou WY, 2020, INT J CLIN EXP PATHO, V13, P1008

NR 59

TC 5

Z9 5

U1 1

U2 5

PU MDPI

PI BASEL

PA ST ALBAN-ANLAGE 66, CH-4052 BASEL, SWITZERLAND

EI 1424-8247

J9 PHARMACEUTICALS-BASE

JI Pharmaceuticals

PD JUL

PY 2021

VL 14

IS 7

AR 633

DI 10.3390/ph14070633

PG 20

WC Chemistry, Medicinal; Pharmacology & Pharmacy

WE Science Citation Index Expanded (SCI-EXPANDED)

SC Pharmacology & Pharmacy

GA TN9XO

UT WOS:000676578700001

PM 34208799

OA Green Accepted, gold, Green Published

DA 2023-04-05

ER

PT J

AU Jeon, H

Huynh, DTN

Baek, N

Nguyen, TLL

Heo, KS

AF Jeon, Hyesu

Diem Thi Ngoc Huynh

Baek, Naehwan

Thuy Le Lam Nguyen

Heo, Kyung-Sun

TI Ginsenoside-Rg2 affects cell growth via regulating ROS-mediated AMPK

activation and cell cycle in MCF-7 cells

SO PHYTOMEDICINE

LA English

DT Article

DE Ginsenoside; Breast cancer; ROS production; Cell cycle; Apoptosis

ID CANCER; AUTOPHAGY; INHIBITORS; APOPTOSIS; PATHWAY; ARREST

AB Background: Ginsenoside-Rg2 (G-Rg2) is a protopanaxatriol-type ginsenoside isolated from ginseng. It has been found to exhibit various pharmacological effects, including antioxidant, anti-inflammatory, and anticancer effects.

Purpose: This study aimed to investigate the anticancer effects of G-Rg2 on estrogen receptor-positive MCF-7 breast cancer (BC) cells, and the underlying mechanisms involving in reactive oxygen species (ROS) production.

Study design/Methods: Cell viability, cell cycle distribution, apoptosis, and ROS production were measured following exposure to G-Rg2. The protein expression levels of p-ERK1/2, p-Akt, PARP, p-Rb, cyclin D1, CDK6, and p-AMPK were quantified using western blot analysis. The in vivo activity of G-Rg2 was assessed in a xenograft model. Immunohistochemistry staining for p-Rb and p-AMPK was performed in tumor tissues.

Results: G-Rg2 significantly decreased cell viability but increased cell apoptosis. In MCF-7 cells, G-Rg2 increased ROS production by inhibiting ERK1/2 and Akt activation. G-Rg2-induced ROS induced G0/G1 cell cycle arrest and AMPK phosphorylation. In the xenograft model, the 5 mg/kg G-Rg2-treated group showed decreased tumor volume and weight, similar to the 5 mg/kg 4-OHT-treated group, compared to the control group. Immunohistochemistry staining showed that G-Rg2 treatment decreased Rb phosphorylation, while increasing AMPK phosphorylation in tumor tissues.

Conclusion: G-Rg2 has potential anticancer effects by increasing the ROS-AMPK signaling pathway and inhibiting ERK1/2 and Akt activation-mediated cell proliferation and cell cycle progression in MCF-7 BC cells.

C1 [Jeon, Hyesu; Diem Thi Ngoc Huynh; Baek, Naehwan; Thuy Le Lam Nguyen; Heo, Kyung-Sun] Chungnam Natl Univ, Coll Pharm, 99 Daehak Ro, Daejeon, South Korea.

[Jeon, Hyesu; Diem Thi Ngoc Huynh; Baek, Naehwan; Thuy Le Lam Nguyen; Heo, Kyung-Sun] Chungnam Natl Univ, Inst Drug Res & Dev, 99 Daehak Ro, Daejeon, South Korea.

[Diem Thi Ngoc Huynh] Da Nang Univ Med Technol & Pharm, Dept Pharm, Da Nang, Vietnam.

C3 Chungnam National University; Chungnam National University; Danang

University of Medical Technology & Pharmacy

RP Heo, KS (通讯作者)，Chungnam Natl Univ, Coll Pharm, 99 Daehak Ro, Daejeon, South Korea.; Heo, KS (通讯作者)，Chungnam Natl Univ, Inst Drug Res & Dev, 99 Daehak Ro, Daejeon, South Korea.

EM kheo@cnu.ac.kr

OI Huynh, Diem Thi Ngoc/0000-0003-1822-5518; Heo,

Kyung-Sun/0000-0003-3800-7665

FU National Research Foundation of Korea [KNRF-2019R1C1C100733112]

FX This research was supported by National Research Foundation of Korea

(KNRF-2019R1C1C100733112).

CR An W, 2019, FRONT PHARMACOL, V10, DOI 10.3389/fphar.2019.00758

Asati V, 2016, EUR J MED CHEM, V109, P314, DOI 10.1016/j.ejmech.2016.01.012

Bi YL, 2018, PHYTOMEDICINE, V39, P10, DOI 10.1016/j.phymed.2017.12.001

Carneiro BA, 2020, NAT REV CLIN ONCOL, V17, P395, DOI 10.1038/s41571-020-0341-y

Chen X, 2018, CELL PHYSIOL BIOCHEM, V50, P1201, DOI 10.1159/000494547

Chung Y, 2018, ANIM CELLS SYST, V22, P382, DOI 10.1080/19768354.2018.1545696

Ferlay J, 2015, INT J CANCER, V136, pE359, DOI 10.1002/ijc.29210

Heo KS, 2019, BMB REP, V52, P113, DOI 10.5483/BMBRep.2019.52.2.017

Huynh DTN, 2020, INT J MOL SCI, V21, DOI 10.3390/ijms21186656

Jin FJ, 2019, CANCER BIOL MED, V16, P38, DOI 10.20892/j.issn.2095-3941.2018.0253

Jin Y, 2020, ARCH PHARM RES, V43, P773, DOI [10.1007/s12272-020-01265-8, 10.1007/s12272-020-01255-w]

Jin Y, 2019, BMB REP, V52, P706, DOI 10.5483/BMBRep.2019.52.12.234

Liu HM, 2019, FOOD FUNCT, V10, P3603, DOI 10.1039/c9fo00027e

Liu SL, 2019, PLOS ONE, V14, DOI 10.1371/journal.pone.0211905

Mills CC, 2018, CANCER RES, V78, P320, DOI 10.1158/0008-5472.CAN-17-2782

Montalesi E, 2020, CANCERS, V12, DOI 10.3390/cancers12010167

Palesh O, 2018, BREAST J, V24, P167, DOI 10.1111/tbj.12862

Park HS, 2018, KOREAN J PHYSIOL PHA, V22, P349, DOI 10.4196/kjpp.2018.22.3.349

Ren GW, 2016, PHYTOMEDICINE, V23, P939, DOI 10.1016/j.phymed.2016.05.008

Schwartz GK, 2005, J CLIN ONCOL, V23, P9408, DOI 10.1200/JCO.2005.01.5594

Song X, 2014, CELL DEATH DIS, V5, DOI 10.1038/cddis.2014.463

Sun MY, 2017, INT J MOL MED, V39, P507, DOI 10.3892/ijmm.2017.2857

Wang GW, 2014, PLOS ONE, V9, DOI 10.1371/journal.pone.0115151

Wang L, 2019, J EXP CLIN CANC RES, V38, DOI 10.1186/s13046-019-1109-z

Wang Y, 2018, J EXP CLIN CANC RES, V37, DOI 10.1186/s13046-018-0731-5

Yang J, 2019, MOL CANCER, V18, DOI 10.1186/s12943-019-0954-x

Zhao Y, 2017, MOL CANCER, V16, DOI 10.1186/s12943-017-0648-1

Zhu X, 2014, TOXICOL LETT, V227, P65, DOI 10.1016/j.toxlet.2014.03.015

Zou ZZ, 2017, APOPTOSIS, V22, P1321, DOI 10.1007/s10495-017-1424-9

NR 29

TC 19

Z9 19

U1 1

U2 17

PU ELSEVIER GMBH

PI MUNICH

PA HACKERBRUCKE 6, 80335 MUNICH, GERMANY

SN 0944-7113

EI 1618-095X

J9 PHYTOMEDICINE

JI Phytomedicine

PD MAY

PY 2021

VL 85

AR 153549

DI 10.1016/j.phymed.2021.153549

EA APR 2021

PG 8

WC Plant Sciences; Chemistry, Medicinal; Integrative & Complementary

Medicine; Pharmacology & Pharmacy

WE Science Citation Index Expanded (SCI-EXPANDED)

SC Plant Sciences; Pharmacology & Pharmacy; Integrative & Complementary

Medicine

GA RN1IG

UT WOS:000640107200047

PM 33819767

DA 2023-04-05

ER

PT J

AU Li, S

Wang, P

Yang, WZ

Zhao, CH

Zhang, LQ

Zhang, JB

Qin, YW

Xu, HY

Huang, LQ

AF Li, Sen

Wang, Ping

Yang, Wenzhi

Zhao, Chunhui

Zhang, Luoqi

Zhang, Jingbo

Qin, Yuewen

Xu, Haiyu

Huang, Luqi

TI Characterization of the Components and Pharmacological Effects of

Mountain-Cultivated Ginseng and Garden Ginseng Based on the Integrative

Pharmacology Strategy

SO FRONTIERS IN PHARMACOLOGY

LA English

DT Article

DE mountain-cultivated ginseng; garden ginseng; UHPLC; IM-QTOF-HDMSE;

metabolomics analysis; TCMIP V2.0

AB Panax ginseng C. A. Mey (PGCAM) is a herbaceous perennial belonging to the Araliaceae family, mainly including Mountain-Cultivated Ginseng (MCG) and Garden Ginseng (GG) on the market. We aimed to establish a rapid, accurate and effective method to distinguish 15-year-old MCG and GG using ultra-performance liquid chromatography-quadrupole time-of-flight-tandem mass spectrometry (UPLC-QTOF-MS/MS), and also explored the pharmacological mechanisms of the main components using the Integrative Pharmacology-based Network Computational Research Platform of Traditional Chinese Medicine (TCMIP V2.0; ). Altogether, 23 potential quality markers were characterized to distinguish 15-year-old MCG and GG, including ginsenosides Ra2, Rg1, and Ra1, and malonyl-ginsenoside Ra3, etc. The contents of 19 constituents (mainly protopanaxadiol-type) were higher in MCG compared with that in GG, and four constituents (mainly carbohydrate compounds) were higher in GG. The 105 putative targets corresponding to 23 potential quality markers were mainly involved in 30 pathways, which could be divided into 10 models, such as immune regulation, systems (metabolic, nervous, cardiovascular, reproductive), blood-pressure regulation, as well as antitumor, antiaging, antibacterial and anti-inflammatory effects. Furthermore, the potential quality markers of MCG and GG could inhibit the proliferation of breast cancer by regulating the mRNA expression of PSA, S6K, MDM2, and P53 genes by acting on AR, MTOR, PI3K and other targets. The Integrative Pharmacology Strategy may provide an efficient way to identify chemical constituents and explore the pharmacological actions of TCM formulations.

C1 [Li, Sen; Zhang, Luoqi] Jilin Agr Univ, Coll Chinese Med Mat, Changchun, Peoples R China.

[Li, Sen; Zhang, Luoqi; Huang, Luqi] China Acad Chinese Med Sci, Natl Resource Ctr Chinese Mat Med, Beijing, Peoples R China.

[Wang, Ping; Zhao, Chunhui; Qin, Yuewen; Xu, Haiyu] China Acad Chinese Med Sci, Inst Chinese Mat Med, Beijing, Peoples R China.

[Yang, Wenzhi] Tianjin Univ Tradit Chinese Med, Tianjin State Key Lab Modern Chinese Med, Tianjin, Peoples R China.

[Zhang, Jingbo] Heilongjiang Univ Chinese Med, Coll Pharm, Harbin, Peoples R China.

C3 Jilin Agricultural University; China Academy of Chinese Medical

Sciences; National Resource Center for Chinese Materia Medica, CACMS;

China Academy of Chinese Medical Sciences; Institute of Chinese Materia

Medica, CACMS; Tianjin University of Traditional Chinese Medicine;

Heilongjiang University of Chinese Medicine

RP Huang, LQ (通讯作者)，China Acad Chinese Med Sci, Natl Resource Ctr Chinese Mat Med, Beijing, Peoples R China.; Xu, HY (通讯作者)，China Acad Chinese Med Sci, Inst Chinese Mat Med, Beijing, Peoples R China.

EM hyxu@icmm.ac.cn; huangluqi01@126.com

RI Zhang, Jing/GWZ-7332-2022; Qin, Yue/GWV-0264-2022

FU National Key Research and Development Program of China [2017YFC1702303,

2017YFC1702104]; National Natural Science Foundation of China [81830111,

81774201]; National Science and Technology Major Project of China

[2019ZX09201005-001-003]; Youth Innovation Team of Shaanxi Universities

[2016SF-378]; Shaanxi Provincial Science and Technology Department

Project [2016SF-378]; Fundamental Research Funds for the Central Public

Welfare Research Institutes [ZXKT17058]

FX This work was supported by grants from the National Key Research and

Development Program of China (2017YFC1702303, 2017YFC1702104), National

Natural Science Foundation of China (81830111, 81774201), National

Science and Technology Major Project of China (2019ZX09201005-001-003),

the Youth Innovation Team of Shaanxi Universities and Shaanxi Provincial

Science and Technology Department Project (2016SF-378), and the

Fundamental Research Funds for the Central Public Welfare Research

Institutes (ZXKT17058). These funding agencies had no role in the study

design, collection, analyses, or interpretation of data, writing of the

report, or the decision to submit the manuscript for publication.

CR Balk SP, 2003, J CLIN ONCOL, V21, P383, DOI 10.1200/JCO.2003.02.083

Bin BH, 2017, ARCH DERMATOL RES, V309, P217, DOI 10.1007/s00403-017-1717-4

Chang Xiang-Wei, 2016, Zhongguo Zhong Yao Za Zhi, V41, P3609, DOI 10.4268/cjcmm20161918

Chang XW, 2017, J PHARMACEUT BIOMED, V141, P108, DOI 10.1016/j.jpba.2017.04.009

[崔丽丽 Cui Lili], 2013, [吉林农业大学学报, Journal of Jilin Agricultural University], V35, P427

Du N, 2018, DRUG DES DEV THER, V12, P3517, DOI 10.2147/DDDT.S171286

Guo XJ, 2019, ARTIF CELL NANOMED B, V47, P2972, DOI 10.1080/21691401.2019.1640712

Gupta A, 2019, BIOMED PHARMACOTHER, V109, P484, DOI 10.1016/j.biopha.2018.10.155

Jeong HJ, 2019, J GINSENG RES, V43, P475, DOI 10.1016/j.jgr.2018.04.005

Jia L, 2019, MOLECULES, V24, DOI 10.3390/molecules24112188

Kim JH, 2018, J GINSENG RES, V42, P264, DOI 10.1016/j.jgr.2017.10.004

Kim J, 2020, EVID-BASED COMPL ALT, V2020, DOI 10.1155/2020/2584783

Konopleva M, 2020, LEUKEMIA, V34, P2858, DOI 10.1038/s41375-020-0949-z

Li G, 2019, SCI REP-UK, V9, DOI 10.1038/s41598-019-43537-4

Liang S, 2016, PESTIC BIOCHEM PHYS, V134, P63, DOI 10.1016/j.pestbp.2016.04.005

Liu JQ, 2021, PHYTOMEDICINE, V86, DOI 10.1016/j.phymed.2019.153077

Liu Z, 2013, J ETHNOPHARMACOL, V145, P233, DOI 10.1016/j.jep.2012.10.058

Mancuso C, 2017, FOOD CHEM TOXICOL, V107, P362, DOI 10.1016/j.fct.2017.07.019

Mou Z, 2017, BIOMED PHARMACOTHER, V92, P962, DOI 10.1016/j.biopha.2017.05.119

Nguyen NH, 2019, INFLAMMOPHARMACOLOGY, V27, P871, DOI 10.1007/s10787-019-00630-4

Qin F, 2006, CHEM BIODIVERS, V3, P1144, DOI 10.1002/cbdv.200690116

Ruan XiaoJia, 2018, Shipin Kexue / Food Science, V39, P195

Shen Liang, 2019, Zhongguo Zhong Yao Za Zhi, V44, P5124, DOI 10.19540/j.cnki.cjcmm.20191009.106

Shi ZY, 2019, MOLECULES, V24, DOI 10.3390/molecules24132443

Sim U, 2020, FOOD CHEM, V312, DOI 10.1016/j.foodchem.2019.126075

Sridharan S, 2020, INT J MOL SCI, V21, DOI 10.3390/ijms21041199

Stevens B, 2016, COCHRANE DB SYST REV, DOI 10.1002/14651858.CD001069.pub5

Sun HX, 2005, CHEM BIODIVERS, V2, P510, DOI 10.1002/cbdv.200590032

Wang HP, 2016, J GINSENG RES, V40, P382, DOI 10.1016/j.jgr.2015.12.001

Wu W, 2018, PHYTOCHEM ANALYSIS, V29, P331, DOI 10.1002/pca.2752

Xu HY, 2019, NUCLEIC ACIDS RES, V47, pD976, DOI 10.1093/nar/gky987

Xu Hai-Yu, 2014, Zhongguo Zhong Yao Za Zhi, V39, P357

Xu XF, 2016, J GINSENG RES, V40, P344, DOI 10.1016/j.jgr.2015.11.001

Xu ZM, 2018, INT J MOL SCI, V19, DOI 10.3390/ijms19113658

Yang WZ, 2012, ANAL CHIM ACTA, V739, P56, DOI 10.1016/j.aca.2012.06.017

Yang WZ, 2016, ACTA PHARM SIN B, V6, P568, DOI 10.1016/j.apsb.2016.05.005

Yang WZ, 2016, J PHARMACEUT BIOMED, V128, P322, DOI 10.1016/j.jpba.2016.05.035

Yang X.B., 2013, MOD CHIN MED, V15

Zhang CX, 2019, MOLECULES, V24, DOI 10.3390/molecules24152708

Zhu HL, 2019, MOLECULES, V24, DOI 10.3390/molecules24010033

Zhu LL, 2021, J SCI FOOD AGR, V101, P1491, DOI 10.1002/jsfa.10762

Zuo TT, 2020, J PHARM ANAL, V10, P597, DOI 10.1016/j.jpha.2019.11.001

NR 42

TC 7

Z9 8

U1 1

U2 43

PU FRONTIERS MEDIA SA

PI LAUSANNE

PA AVENUE DU TRIBUNAL FEDERAL 34, LAUSANNE, CH-1015, SWITZERLAND

SN 1663-9812

J9 FRONT PHARMACOL

JI Front. Pharmacol.

PD APR 26

PY 2021

VL 12

AR 659954

DI 10.3389/fphar.2021.659954

PG 13

WC Pharmacology & Pharmacy

WE Science Citation Index Expanded (SCI-EXPANDED)

SC Pharmacology & Pharmacy

GA RZ4ZL

UT WOS:000648606300001

PM 33981239

OA gold, Green Published

DA 2023-04-05

ER

PT J

AU Huynh, DTN

Jin, Y

Myung, CS

Heo, KS

AF Huynh Diem Thi Ngoc

Jin, Yujin

Myung, Chang-Seon

Heo, Kyung-Sun

TI Ginsenoside Rh1 Induces MCF-7 Cell Apoptosis and Autophagic Cell Death

through ROS-Mediated Akt Signaling

SO CANCERS

LA English

DT Article

DE Akt; apoptosis; autophagy; ginsenoside Rh1; reactive oxygen species

AB Simple Summary

Breast cancer (BC) is the most common cause of cancer-related deaths among women worldwide, and its incidence has been increasing. However, current therapeutic approaches, such as chemotherapy, radiation, and hormonal therapy, have become increasingly ineffective because of their severe adverse effects and multidrug resistance. Therefore, the discovery of new potential candidates for BC therapy is essential. Here, we investigated whether ginsenoside Rh1 exhibits anticancer effects on BC. We found that this ginsenoside effectively inhibited the growth of BC cells in both cell cultures and mice. Therefore, ginsenoside Rh1 is a promising candidate for BC treatment.

Breast cancer (BC) is the leading cause of cancer-related deaths among women worldwide. Ginsenosides exhibit anticancer activity against various cancer cells. However, the effects of ginsenoside Rh1 on BC and the underlying mechanisms remain unknown. Here, we investigated the anticancer effects of Rh1 on human BC MCF-7 and HCC1428 cells and the underlying signaling pathways. The anticancer effects of Rh1 in vitro were evaluated using sulforhodamine B (SRB), 3-(4, 5-dimethylthiazole-2-yl)-2, 5-diphenyltetrazolium bromide (MTT), clonogenic assay, propidium iodide (PI)/Hoechst staining, Western blotting, flow cytometry, and immunofluorescence analysis. The in vivo effects of Rh1 were determined using a xenograft model via hematoxylin and eosin and the immunohistochemistry staining of tumor tissues. We found that Rh1 exerted cytotoxicity in the cells by increasing cell apoptosis, autophagy, and cell cycle arrest. These effects were further enhanced by a phosphatidylinositol 3-kinase (PI3K) inhibitor but were rescued by the inhibition of reactive oxygen species (ROS). Moreover, enhanced ROS generation by Rh1 inhibited the activation of the PI3K/Akt pathway. Consistently, Rh1 treatment significantly reduced tumor growth in vivo and increased the ROS production and protein expression of LC3B and cleaved caspase-3 but decreased the phosphorylation of Akt and retinoblastoma (Rb) in tumor tissues. Taken together, Rh1 exerted a potential anticancer effect on BC cells by inducing cell cycle arrest, apoptosis, and autophagy via inhibition of the ROS-mediated PI3K/Akt pathway.

C1 [Huynh Diem Thi Ngoc; Jin, Yujin; Myung, Chang-Seon; Heo, Kyung-Sun] Chungnam Natl Univ, Coll Pharm, Daejeon 34134, South Korea.

[Huynh Diem Thi Ngoc] Da Nang Univ Med Technol & Pharm, Dept Pharm, Da Nang 550000, Vietnam.

C3 Chungnam National University; Danang University of Medical Technology &

Pharmacy

RP Heo, KS (通讯作者)，Chungnam Natl Univ, Coll Pharm, Daejeon 34134, South Korea.

EM ngocdiemphar@gmail.com; 201850535@o.cnu.ac.kr; cm8r@cnu.ac.kr;

kheo@cnu.ac.kr

OI Myung, Chang-Seon/0000-0002-6292-2911; Huynh, Diem Thi

Ngoc/0000-0003-1822-5518

FU [2017R1A4A1015860]; [2019R1C1C100733112]; National Research Foundation

of Korea [4120200813639] Funding Source: Korea Institute of Science &

Technology Information (KISTI), National Science & Technology

Information Service (NTIS)

FX This researchwas funded fromtheNational Research Foundation ofKorea

(2017R1A4A1015860 and 2019R1C1C100733112).

CR Aldieri E, 2008, CURR DRUG METAB, V9, P686, DOI 10.2174/138920008786049285

Aoki M, 2017, CURR TOP MICROBIOL, V407, P153, DOI 10.1007/82_2017_6

Augsburger F, 2019, REDOX BIOL, V26, DOI 10.1016/j.redox.2019.101272

Carneiro BA, 2020, NAT REV CLIN ONCOL, V17, P395, DOI 10.1038/s41571-020-0341-y

Choi YJ, 2011, FITOTERAPIA, V82, P911, DOI 10.1016/j.fitote.2011.05.005

Chumakova OV, 2006, ULTRASOUND MED BIOL, V32, P751, DOI 10.1016/j.ultrasmedbio.2006.01.011

Cui L, 2018, ARCH PHARM RES, V41, P299, DOI 10.1007/s12272-017-0990-2

Tam DNH, 2018, PLANTA MED, V84, P139, DOI 10.1055/s-0043-124087

Huynh DTN, 2020, BIOCHEM BIOPH RES CO, V523, P267, DOI 10.1016/j.bbrc.2019.12.053

Franken NAP, 2006, NAT PROTOC, V1, P2315, DOI 10.1038/nprot.2006.339

Fruman DA, 2014, NAT REV DRUG DISCOV, V13, P140, DOI 10.1038/nrd4204

Greten FR, 2019, IMMUNITY, V51, P27, DOI 10.1016/j.immuni.2019.06.025

He W, 2008, J AGR FOOD CHEM, V56, P1921, DOI 10.1021/jf073364k

Huynh DTN, 2020, INT J MOL SCI, V21, DOI 10.3390/ijms21186656

Hwang JT, 2007, ANN NY ACAD SCI, V1095, P441, DOI 10.1196/annals.1397.047

Jeon H, 2021, PHYTOMEDICINE, V85, DOI 10.1016/j.phymed.2021.153549

Jin Y, 2020, ARCH PHARM RES, V43, P773, DOI [10.1007/s12272-020-01265-8, 10.1007/s12272-020-01255-w]

Jung JS, 2013, NEUROCHEM INT, V63, P80, DOI 10.1016/j.neuint.2013.05.002

Kim SM, 2020, J NUTR BIOCHEM, V83, DOI 10.1016/j.jnutbio.2020.108427

Kim YS, 2008, J MICROBIOL BIOTECHN, V18, P1109

Kma L, 2021, BIOTECHNOL APPL BIOC, DOI 10.1002/bab.2104

Lee HJ, 2018, NUTRIENTS, V10, DOI 10.3390/nu10081043

Lee Y, 2003, J STEROID BIOCHEM, V84, P463, DOI 10.1016/S0960-0760(03)00067-0

Liu B, 2010, ACTA PHARMACOL SIN, V31, P1154, DOI 10.1038/aps.2010.118

Lyu X, 2019, ONCOL LETT, V18, P4160, DOI 10.3892/ol.2019.10742

Mathew R, 2007, NAT REV CANCER, V7, P961, DOI 10.1038/nrc2254

Mathiyalagan R, 2019, MOLECULES, V24, DOI 10.3390/molecules24234367

Mitra S, 2018, EVID-BASED COMPL ALT, V2018, DOI 10.1155/2018/8324696

Neve RM, 2006, CANCER CELL, V10, P515, DOI 10.1016/j.ccr.2006.10.008

Nguyen TL, 2021, ARCH PHARM RES, V44, P241, DOI 10.1007/s12272-020-01304-4

Nitulescu GM, 2018, INT J ONCOL, V53, P2319, DOI 10.3892/ijo.2018.4597

Nowsheen S, 2012, CANCER RES, V72, P4796, DOI 10.1158/0008-5472.CAN-12-1287

Otto T, 2017, NAT REV CANCER, V17, P93, DOI 10.1038/nrc.2016.138

Palesh O, 2018, BREAST J, V24, P167, DOI 10.1111/tbj.12862

Park KR, 2011, CANCER LETT, V312, P178, DOI 10.1016/j.canlet.2011.08.001

Qu ZP, 2016, ONCOTARGET, V7, P66003, DOI 10.18632/oncotarget.11788

Quan K, 2015, SCI REP-UK, V5, DOI 10.1038/srep08598

Ren GW, 2016, PHYTOMEDICINE, V23, P939, DOI 10.1016/j.phymed.2016.05.008

Rybstein MD, 2018, NAT CELL BIOL, V20, P243, DOI 10.1038/s41556-018-0042-2

Ryu H, 2018, J CELL PHYSIOL, V233, P4666, DOI 10.1002/jcp.26234

Saleem MZ, 2020, ONCOTARGETS THER, V13, P435, DOI 10.2147/OTT.S228702

Shahbandi A, 2020, CELL DEATH DIFFER, V27, P3097, DOI 10.1038/s41418-020-0564-6

Silva C, 2019, METABOLITES, V9, DOI 10.3390/metabo9050102

Siraj FM, 2015, MOL SIMULAT, V41, P1219, DOI 10.1080/08927022.2014.970188

Sobhani N, 2019, CELLS-BASEL, V8, DOI 10.3390/cells8040321

Stoica GE, 2003, ONCOGENE, V22, P7998, DOI 10.1038/sj.onc.1206769

Sun HX, 2006, INT IMMUNOPHARMACOL, V6, P14, DOI 10.1016/j.intimp.2005.07.003

Vichai V, 2006, NAT PROTOC, V1, P1112, DOI 10.1038/nprot.2006.179

Wu K, 2019, ANTIOXID REDOX SIGN, V30, P1983, DOI 10.1089/ars.2017.7486

Wu Q, 2018, BIOCHEM PHARMACOL, V148, P64, DOI 10.1016/j.bcp.2017.12.004

Zhang DD, 2017, ARCH PHARM RES, V40, P579, DOI 10.1007/s12272-017-0899-9

Zhang HW, 2018, SCI REP-UK, V8, DOI 10.1038/s41598-018-29308-7

Zhao Y, 2017, MOL CANCER, V16, DOI 10.1186/s12943-017-0648-1

Zielke S, 2018, CELL DEATH DIS, V9, DOI 10.1038/s41419-018-1003-1

Zou ZZ, 2017, APOPTOSIS, V22, P1321, DOI 10.1007/s10495-017-1424-9

NR 55

TC 14

Z9 14

U1 1

U2 12

PU MDPI

PI BASEL

PA ST ALBAN-ANLAGE 66, CH-4052 BASEL, SWITZERLAND

EI 2072-6694

J9 CANCERS

JI Cancers

PD APR

PY 2021

VL 13

IS 8

AR 1892

DI 10.3390/cancers13081892

PG 18

WC Oncology

WE Science Citation Index Expanded (SCI-EXPANDED)

SC Oncology

GA RS7QZ

UT WOS:000643971000001

PM 33920802

OA Green Published, gold

DA 2023-04-05

ER

PT J

AU Liu, G

Qi, XY

Li, XT

Sun, FY

AF Liu, Guang

Qi, Xiaoyong

Li, Xingtao

Sun, Fangyi

TI Ginsenoside Rg2 protects cardiomyocytes against trastuzumab-induced

toxicity by inducing autophagy

SO EXPERIMENTAL AND THERAPEUTIC MEDICINE

LA English

DT Article

DE trastuzumab; ginsenoside Rg2; apoptosis; autophagy

AB Trastuzumab (TZM) significantly improves the outcomes of patients with breast cancer; however, it is associated with severe cardiotoxicity. Ginsenoside Rg2 was reported to exert protective effects against myocardial injury and apoptosis in human cardiomyocytes (HCMs). However, whether ginsenoside Rg2 protects HCMs against TZM-induced toxicity remains unclear. The present study investigated the proliferation of HCMs using a Cell Counting Kit-8 assay and Ki67 immunofluorescence staining. Apoptotic cells were detected by Annexin V/propidium iodide staining and flow cytometry. Furthermore, monodansylcadaverine staining was performed to detect cell autophagy. In addition, western blotting was used to detect the expression levels of phosphorylated (p)-Akt, p-mTOR, beclin 1, microtubule associated protein 1 light chain 3 alpha (LC3) and autophagy protein 5 (ATG5) in HCMs. Pretreatment with ginsenoside Rg2 significantly protected HCMs against TZM-induced cytotoxicity by inhibiting apoptosis. Furthermore, pretreatment with ginsenoside Rg2 induced autophagy in HCMs by upregulating the expression levels of p-Akt, p-mTOR, beclin 1, LC3 and ATG5. The results obtained in the present study suggested that ginsenoside Rg2 could protect HCMs against TZM-induced cardiotoxicity by activating autophagy. Therefore, ginsenoside Rg2 may serve as a potential therapeutic agent to prevent TZM-related cardiotoxicity in patients with breast cancer.

C1 [Liu, Guang] Hebei Med Univ, Dept Cardiovasc Med, Shijiazhuang, Hebei, Peoples R China.

[Qi, Xiaoyong] Hebei Gen Hosp, Dept Cardiovasc Med, 348 West Heping Rd, Shijiazhuang 050000, Hebei, Peoples R China.

[Li, Xingtao; Sun, Fangyi] Hebei Med Univ, Affiliated Hosp 4, Dept Cardiovasc Med, Shijiazhuang 050000, Hebei, Peoples R China.

C3 Hebei Medical University; Hebei Medical University

RP Qi, XY (通讯作者)，Hebei Gen Hosp, Dept Cardiovasc Med, 348 West Heping Rd, Shijiazhuang 050000, Hebei, Peoples R China.

EM xiaoyongqi28@126.com

RI Liu, Han/HMD-9231-2023; Li, Xiaomei/HNI-0635-2023; zhang,

yu/HNS-5948-2023; li, xiao/HKV-8405-2023

OI Liu, Han/0000-0002-5269-8477;

CR BIEDERBICK A, 1995, EUR J CELL BIOL, V66, P3

Chen XJ, 2016, EVID-BASED COMPL ALT, V2016, DOI 10.1155/2016/5738694

Chung YH, 2018, ANIM CELLS SYST, V22, P400, DOI 10.1080/19768354.2018.1523806

Chung Y, 2018, ANIM CELLS SYST, V22, P382, DOI 10.1080/19768354.2018.1545696

Fan YY, 2017, AUTOPHAGY, V13, P41, DOI 10.1080/15548627.2016.1240855

Fu WW, 2015, INT J CLIN EXP MED, V8, P19938

Gatica D, 2015, CIRC RES, V116, P456, DOI 10.1161/CIRCRESAHA.114.303788

Gershon N, 2019, COST EFFECT RESOUR A, V17, DOI 10.1186/s12962-019-0174-7

Han SQ, 2014, MABS-AUSTIN, V6, P403, DOI 10.4161/mabs.27443

Hu PF, 2015, CELL PHYSIOL BIOCHEM, V37, P697, DOI 10.1159/000430388

Jeong SJ, 2007, INT J TOXICOL, V26, P151, DOI 10.1080/10915810701226370

Kang HJ, 2016, J PHOTOCH PHOTOBIO B, V165, P232, DOI 10.1016/j.jphotobiol.2016.10.034

Mazzotta M, 2019, J CLIN MED, V8, DOI 10.3390/jcm8020254

Mondaca-Ruff D, 2019, FRONT PHARMACOL, V9, DOI 10.3389/fphar.2018.01553

Sato A, 2019, MOL CLIN ONCOL, V10, P37, DOI 10.3892/mco.2018.1764

Slamon D, 2011, NEW ENGL J MED, V365, P1273, DOI 10.1056/NEJMoa0910383

Sukawa Y, 2014, DIGESTION, V89, P12, DOI 10.1159/000356201

Wang CZ, 2008, ANTICANCER RES, V28, P2545

Xu QM, 2013, J FUNCT FOODS, V5, P2012, DOI 10.1016/j.jff.2013.08.005

Yang Y, 2018, FRONT PHARMACOL, V9, DOI 10.3389/fphar.2018.01392

Ye J, 2016, MOL MED REP, V13, P3083, DOI 10.3892/mmr.2016.4914

Yu JSL, 2016, DEVELOPMENT, V143, P3050, DOI 10.1242/dev.137075

Zhang GZ, 2008, J ETHNOPHARMACOL, V115, P441, DOI 10.1016/j.jep.2007.10.026

NR 23

TC 5

Z9 7

U1 0

U2 9

PU SPANDIDOS PUBL LTD

PI ATHENS

PA POB 18179, ATHENS, 116 10, GREECE

SN 1792-0981

EI 1792-1015

J9 EXP THER MED

JI Exp. Ther. Med.

PD MAY

PY 2021

VL 21

IS 5

AR 473

DI 10.3892/etm.2021.9904

PG 7

WC Medicine, Research & Experimental

WE Science Citation Index Expanded (SCI-EXPANDED)

SC Research & Experimental Medicine

GA RA5ZB

UT WOS:000631495900001

PM 33767768

OA gold, Green Published

DA 2023-04-05

ER

PT J

AU Luo, Z

An, JX

Shi, WJ

Li, CQ

Gao, H

AF Luo, Zhong

An, Jinxia

Shi, Wenjie

Li, Chaoqi

Gao, Hui

TI One step assembly of ginsenoside Rb1-based nanovehicles with fast

cellular transport in photothermal-chemical combined cancer therapy

SO NANOTECHNOLOGY

LA English

DT Article

DE gambogic acid; fast cellular transport; ginsenoside Rb1; mild

photothermal-chemical combined cancer therapy

AB Nowadays, the research of photothermal-chemical co-therapy provides new ideas for the treatment of cancer. However, the harsh photothermal temperature hinders the clinical development of photothermal therapy. To ensure low-temperature photothermal-chemical combined therapy, a safe and feasible drug delivery system is highly desirable. Herein, through one step co-precipitation method, ginsenoside Rb1-based nanovehicles composed of the hydrophobic drug doxorubicin, the photochemical reagent Cypate and the heat shock protein inhibitor gambogic acid was prepared, resulting from the amphiphilicity and membrane permeability of Rb1. Encouragingly, this platform exhibited excellent biocompatibility and rapid cellular uptake, both of which led to significant and irreversible death of breast cancer cells under the trigger of short-term near-infrared light.

C1 [Luo, Zhong; An, Jinxia; Shi, Wenjie; Li, Chaoqi; Gao, Hui] Tianjin Univ Technol, Tianjin Enterprise Key Lab Applicat Res Hyaluron, Tianjin Key Lab Drug Targeting & Bioimaging, Sch Chem & Chem Engn, Tianjin 300384, Peoples R China.

C3 Tianjin University of Technology

RP Gao, H (通讯作者)，Tianjin Univ Technol, Tianjin Enterprise Key Lab Applicat Res Hyaluron, Tianjin Key Lab Drug Targeting & Bioimaging, Sch Chem & Chem Engn, Tianjin 300384, Peoples R China.

EM hgao@tjut.edu.cn

RI Gao, Hui/G-8400-2012

OI Gao, Hui/0000-0002-5009-9999

FU National Natural Science Foundation of China [21674080, 22075209,

U20A20260]; Leading Talents Program of the Tianjin Educational

Committee; Key program of Tianjin Municipal Natural Science Foundation

[18JCZDJC37700, 18JCQNJC72300]; Training Project of Innovation Team of

Colleges and Universities in Tianjin [TD13-5020]; Tianjin Enterprise Key

Laboratory for Application Research of Hyaluronic Acid [KTRDHA-Z201901]

FX The authors thank the National Natural Science Foundation of China

(21674080, 22075209, U20A20260); a distinguished professor of Tianjin,

the Leading Talents Program of the Tianjin Educational Committee; the

Key program of Tianjin Municipal Natural Science Foundation (No.

18JCZDJC37700, 18JCQNJC72300); the Training Project of Innovation Team

of Colleges and Universities in Tianjin (TD13-5020); and the Tianjin

Enterprise Key Laboratory for Application Research of Hyaluronic Acid

(KTRDHA-Z201901) for the financial support.

CR An XN, 2016, ACS NANO, V10, P5947, DOI 10.1021/acsnano.6b01296

Banerji U, 2005, CLIN CANCER RES, V11, P7023, DOI 10.1158/1078-0432.CCR-05-0518

Chen Q, 2016, NAT COMMUN, V7, DOI 10.1038/ncomms13193

Dai Q, 2018, ADV HEALTHC MATER, V7, DOI 10.1002/adhm.201700575

Davenport J, 2011, J NAT PROD, V74, P1085, DOI 10.1021/np200029q

ELIZZI A, 1992, PLANTA MED, V58, P229, DOI 10.1055/s-2006-961441

Han ZH, 2017, J BIOPHOTONICS, V10, P1607, DOI 10.1002/jbio.201600270

Hatami E, 2020, BBA-REV CANCER, V1874, DOI 10.1016/j.bbcan.2020.188381

Joh EH, 2011, BIOCHEM PHARMACOL, V82, P278, DOI 10.1016/j.bcp.2011.05.003

Leung KW, 2007, BRIT J PHARMACOL, V152, P207, DOI 10.1038/sj.bjp.0707359

LI GC, 1995, INT J HYPERTHER, V11, P459, DOI 10.3109/02656739509022483

Li R, 2009, TOXICOLOGY, V262, P98, DOI 10.1016/j.tox.2009.04.059

Li YC, 2006, AM J PATHOL, V168, P1107, DOI 10.2353/ajpath.2006.050959

Liu D, 2017, EXP THER MED, V14, P2895, DOI 10.3892/etm.2017.4889

Liu DD, 2016, ADV FUNCT MATER, V26, P4749, DOI 10.1002/adfm.201600031

Liu ZC, 2013, MOL MED REP, V8, P1397, DOI 10.3892/mmr.2013.1658

Lorent JH, 2014, ORG BIOMOL CHEM, V12, P8803, DOI 10.1039/c4ob01652a

Nicol A, 2017, J AM CHEM SOC, V139, P14792, DOI 10.1021/jacs.7b08710

SCHLOSSER E, 1969, Z NATURFORSCH PT B, VB 24, P1284

Sparg SG, 2004, J ETHNOPHARMACOL, V94, P219, DOI 10.1016/j.jep.2004.05.016

Tang XC, 2018, ACTA PHARM SIN B, V8, P587, DOI 10.1016/j.apsb.2018.05.011

Wang BK, 2016, BIOMATERIALS, V78, P27, DOI 10.1016/j.biomaterials.2015.11.025

Wang SP, 2015, CHEM-BIOL INTERACT, V235, P76, DOI 10.1016/j.cbi.2015.03.017

WASSLER M, 1987, BIOCHEM J, V247, P407, DOI 10.1042/bj2470407

Xia GG, 2017, J EXP CLIN CANC RES, V36, DOI 10.1186/s13046-017-0579-0

Yan WJ, 2013, TUMOR BIOL, V34, P1391, DOI 10.1007/s13277-012-0584-z

Ye YP, 2003, J AM CHEM SOC, V125, P7766, DOI 10.1021/ja034186o

Yi T, 2008, CANCER RES, V68, P1843, DOI 10.1158/0008-5472.CAN-07-5944

Yoo D, 2013, ANGEW CHEM INT EDIT, V52, P13047, DOI 10.1002/anie.201306557

Zhang YX, 2020, CHEM COMMUN, V56, P762, DOI 10.1039/c9cc09357e

Zhou J, 2018, ACS NANO, V12, P2858, DOI 10.1021/acsnano.8b00309

NR 31

TC 2

Z9 3

U1 3

U2 43

PU IOP PUBLISHING LTD

PI BRISTOL

PA TEMPLE CIRCUS, TEMPLE WAY, BRISTOL BS1 6BE, ENGLAND

SN 0957-4484

EI 1361-6528

J9 NANOTECHNOLOGY

JI Nanotechnology

PD MAY 7

PY 2021

VL 32

IS 19

AR 195103

DI 10.1088/1361-6528/abe1f0

PG 10

WC Nanoscience & Nanotechnology; Materials Science, Multidisciplinary;

Physics, Applied

WE Science Citation Index Expanded (SCI-EXPANDED)

SC Science & Technology - Other Topics; Materials Science; Physics

GA QK6JH

UT WOS:000620489700001

PM 33524967

DA 2023-04-05

ER

PT J

AU Zhang, H

Yi, J

Kim, E

Choo, Y

Hai, H

Kim, K

Kim, EK

Ryoo, Z

Kim, M

AF Zhang, Haibo

Yi, Junkoo

Kim, Eungyung

Choo, Yeonsik

Hai, Huang

Kim, Kirim

Kim, Eun-Kyong

Ryoo, Zaeyoung

Kim, Myoungok

TI 20(S)-Ginsenoside Rh2 Suppresses Oral Cancer Cell Growth by Inhibiting

the Src-Raf-ERK Signaling Pathway

SO ANTICANCER RESEARCH

LA English

DT Article

DE 20(S)-Ginsenoside Rh2; Src; ERK; oral cancer

ID EXERTS ANTICANCER ACTIVITY; GINSENOSIDE RH2; CARCINOMA-CELLS;

BREAST-CANCER; PROLIFERATION; APOPTOSIS; RESISTANCE; KINASES; ROS

AB Background: 20(S)-Ginsenoside Rh2 (G-Rh2) has demonstrated therapeutic effects in many types of cancers. We aimed to investigate the potential anticancer activity and underlying mechanisms of G-Rh2 in oral cancer cells. Materials and Methods: The antigrowth effect of G-Rh2 in oral cancer cells was stimulated by cell proliferation, soft agar colony formation, and migration and invasion assay. The cell cycle and apoptosis were detected by flow cytometry. The underlying mechanism of G-Rh2 in oral cancer cells was explored by immunoblotting . Results: G-Rh2 significantly inhibited oral cancer cell growth by inducing apoptosis and cell cycle G(0)/G(1)-phase arrest. G-Rh2 inhibited oral cancer cell migration and invasion through regulation of epithelial-mesenchymal transition (EMT)-related proteins. G-Rh2 inhibited the Src/Raf/ERK signaling pathway in YD10B and Ca9-22 cells. Conclusion: G-Rh2 exerted anticancer activity in vitro by inhibiting the Src/Raf/ERK signaling pathway in oral cancer. G-Rh2 is a potential therapeutic drug for oral cancer treatment.

C1 [Zhang, Haibo; Kim, Eungyung; Hai, Huang; Kim, Myoungok] Kyungpook Natl Univ, Dept Anim Sci & Biotechnol, Sangju, South Korea.

[Yi, Junkoo] Gyeongsangbukdo Livestock Inst Res, Yeongju, South Korea.

[Choo, Yeonsik] Kyungpook Natl Univ, Dept Biol, Coll Natl Sci, Daegu, South Korea.

[Kim, Kirim; Kim, Eun-Kyong] Kyungpook Natl Univ, Dept Dent Hyg, Sangju, South Korea.

[Ryoo, Zaeyoung] Kyungpook Natl Univ, Sch Life Sci, Daegu 41566, South Korea.

C3 Kyungpook National University; Kyungpook National University; Kyungpook

National University; Kyungpook National University

RP Ryoo, Z (通讯作者)，Kyungpook Natl Univ, Sch Life Sci, Daegu 41566, South Korea.; Kim, M (通讯作者)，Kyungpook Natl Univ, Dept Anim Sci & Biotechnol, Sangju Si 37224, Gyeongsangbuk D, South Korea.

EM jaewoong64@hanmail.net; ok4325@knu.ac.kr

FU Basic Science Research Program through the National Research Foundation

of Korea (NRF) - Ministry of Education [2016R1A6A1A05011910]

FX This research was supported by the Basic Science Research Program

through the National Research Foundation of Korea (NRF) funded by the

Ministry of Education (2016R1A6A1A05011910).

CR Akhtar MF, 2020, EUR J PHARMACOL, V888, DOI 10.1016/j.ejphar.2020.173488

An EJ, 2020, MOLECULES, V25, DOI 10.3390/molecules25102301

Bley N, 2021, RNA BIOL, V18, P391, DOI 10.1080/15476286.2020.1812894

Brabletz T, 2018, NAT REV CANCER, V18, P128, DOI 10.1038/nrc.2017.118

Chen Y, 2016, ONCOL REP, V36, P137, DOI 10.3892/or.2016.4774

Chen YY, 2020, ANTI-CANCER AGENT ME, V20, P495, DOI 10.2174/1871520619666191209091230

Chi AC, 2015, CA-CANCER J CLIN, V65, P401, DOI 10.3322/caac.21293

Choi WY, 2013, J PHARM PHARMACOL, V65, P310, DOI 10.1111/j.2042-7158.2012.01598.x

Day Terry A, 2003, Curr Treat Options Oncol, V4, P27, DOI 10.1007/s11864-003-0029-4

Demain AL, 2011, MICROB BIOTECHNOL, V4, P687, DOI 10.1111/j.1751-7915.2010.00221.x

Du PX, 2020, CELL DEATH DIS, V11, DOI 10.1038/s41419-020-02981-1

Dubravka D, 2000, CELL RES, V10, P1, DOI 10.1038/sj.cr.7290031

Gao QR, 2018, CELL PROLIFERAT, V51, DOI 10.1111/cpr.12438

Ge GQ, 2017, BIOL PHARM BULL, V40, P2117, DOI 10.1248/bpb.b17-00463

Guan XM, 2015, ACTA PHARM SIN B, V5, P402, DOI 10.1016/j.apsb.2015.07.005

Han S, 2016, J ETHNOPHARMACOL, V194, P83, DOI 10.1016/j.jep.2016.08.039

Huang JJ, 2016, ACTA BIOCH BIOPH SIN, V48, P750, DOI 10.1093/abbs/gmw049

Jain S, 2015, CANCER RES, V75, P4863, DOI 10.1158/0008-5472.CAN-14-2345

Johnson DE, 2008, ADV ENZYME REGUL, V48, P98, DOI 10.1016/j.advenzreg.2007.11.002

Kabil A, 2008, CARCINOGENESIS, V29, P1862, DOI 10.1093/carcin/bgn138

Kim JH, 2017, J GINSENG RES, V41, P435, DOI 10.1016/j.jgr.2016.08.004

Kim JH, 2017, BIOMED PHARMACOTHER, V96, P871, DOI 10.1016/j.biopha.2017.09.033

Lee H, 2018, J GINSENG RES, V42, P455, DOI 10.1016/j.jgr.2017.05.003

Lee TY, 2020, BIOMOLECULES, V10, DOI 10.3390/biom10081150

Li XJ, 2020, ACTA BIOCHIM POL, V67, P165, DOI 10.18388/abp.2020_5158

LI Y, 2020, ONCOGENE 1013, P4562, DOI DOI 10.1038/S41388-020-01499-2

Li Y, 2018, CELL DEATH DIS, V9, DOI 10.1038/s41419-018-0574-1

Liu GW, 2018, HUM CELL, V31, P189, DOI 10.1007/s13577-017-0189-3

Liu WS, 2015, ONCOTARGET, V6, P35522, DOI 10.18632/oncotarget.5849

Ma J, 2019, EXP THER MED, V18, P630, DOI 10.3892/etm.2019.7604

Mikesh LM, 2010, MELANOMA RES, V20, P485, DOI 10.1097/CMR.0b013e32833fafb4

Petitprez A, 2013, CURR PHARM DESIGN, V19, P958, DOI 10.2174/138161213804547204

Peyressatre M, 2015, CANCERS, V7, P179, DOI 10.3390/cancers7010179

Ribeiro AS, 2018, CELL COMMUN SIGNAL, V16, DOI 10.1186/s12964-018-0286-2

Rivera C, 2015, INT J CLIN EXP PATHO, V8, P11884

Saeed M, 2015, FRONT PHARMACOL, V6, DOI 10.3389/fphar.2015.00267

Shi QQ, 2016, ONCOL REP, V36, P2059, DOI 10.3892/or.2016.5033

Shi X, 2018, MOL MED REP, V17, P4811, DOI 10.3892/mmr.2018.8454

Simpkins F, 2012, CLIN CANCER RES, V18, P5911, DOI 10.1158/1078-0432.CCR-12-1257

Tang XP, 2013, WORLD J GASTROENTERO, V19, P1582, DOI 10.3748/wjg.v19.i10.1582

Tong-Lin Wu Tony, 2018, Oncotarget, V9, P11109, DOI 10.18632/oncotarget.24326

Wang YS, 2020, BIOMOLECULES, V10, DOI 10.3390/biom10040528

Xia T, 2020, J GINSENG RES, V44, P725, DOI 10.1016/j.jgr.2019.07.003

Yang JJ, 2016, J GINSENG RES, V40, P400, DOI 10.1016/j.jgr.2016.03.007

Yang P, 2018, CANCER LETT, V438, P76, DOI 10.1016/j.canlet.2018.09.006

Zhang BP, 2020, BIOMED ENVIRON SCI, V33, P713, DOI 10.3967/bes2020.093

Zhou J, 2020, ONCOTARGETS THER, V13, P4635, DOI 10.2147/OTT.S245749

NR 47

TC 3

Z9 4

U1 5

U2 14

PU INT INST ANTICANCER RESEARCH

PI ATHENS

PA EDITORIAL OFFICE 1ST KM KAPANDRITIOU-KALAMOU RD KAPANDRITI, PO BOX 22,

ATHENS 19014, GREECE

SN 0250-7005

EI 1791-7530

J9 ANTICANCER RES

JI Anticancer Res.

PD JAN

PY 2021

VL 41

IS 1

BP 227

EP 235

DI 10.21873/anticanres.14769

PG 9

WC Oncology

WE Science Citation Index Expanded (SCI-EXPANDED)

SC Oncology

GA PT4ER

UT WOS:000608568800011

PM 33419817

DA 2023-04-05

ER

PT J

AU Kim, H

Ji, HW

Kim, HW

Yun, SH

Park, JE

Kim, SJ

AF Kim, Heejoo

Ji, Hwee Won

Kim, Hyeon Woo

Yun, Sung Hwan

Park, Jae Eun

Kim, Sun Jung

TI Ginsenoside Rg3 Prevents Oncogenic Long Noncoding RNA ATXN8OS from

Inhibiting Tumor-Suppressive microRNA-424-5p in Breast Cancer Cells

SO BIOMOLECULES

LA English

DT Article

DE ceRNA; CpG methylation; ginsenoside Rg3; long noncoding RNA; microRNA

AB Ginsenoside Rg3 exerts antiproliferation activity on cancer cells by regulating diverse noncoding RNAs. However, little is known about the role of long noncoding RNAs (lncRNAs) or their relationship with competitive endogenous RNA (ceRNA) in Rg3-treated cancer cells. Here, a lncRNA (ATXN8OS) was found to be downregulated via Rg3-mediated promoter hypermethylation in MCF-7 breast cancer cells. SiRNA-induced downregulation of ATXN8OS decreased cell proliferation but increased apoptosis, suggesting that the noncoding RNA possessed proproliferation activity. An in silico search for potential ATXN8OS-targeting microRNAs (miRs) identified a promising candidate (miR-424-5p) based on its high binding score. As expected, miR-424-5p suppressed proliferation and stimulated apoptosis of the MCF-7 cells. The in silico miR-target-gene prediction identified 200 potential target genes of miR-424-5p, which were subsequently narrowed down to seven that underwent hypermethylation at their promoter by Rg3. Among them, three genes (EYA1, DACH1, and CHRM3) were previously known oncogenes and were proven to be oppositely regulated by ATXN8OS and miR-424-5p. When taken together, Rg3 downregulated ATXN8OS that inhibited the tumor-suppressive miR-424-5p, leading to the downregulation of the oncogenic target genes.

C1 [Kim, Heejoo; Ji, Hwee Won; Kim, Hyeon Woo; Yun, Sung Hwan; Park, Jae Eun; Kim, Sun Jung] Dongguk Univ Seoul, Dept Life Sci, Goyang 10326, South Korea.

C3 Dongguk University

RP Kim, SJ (通讯作者)，Dongguk Univ Seoul, Dept Life Sci, Goyang 10326, South Korea.

EM heejoo0923@dongguk.edu; hweewon96@dongguk.edu; opopr5@dongguk.edu;

skskbby@dongguk.edu; 201511717@dongguk.edu; sunjungk@dongguk.edu

RI kim, sun jung/T-1013-2019

OI Kim, Hyeon Woo/0000-0002-8951-6008

FU Basic Science Research Program of the National Research Foundation of

Korea - Ministry of Education, Science, and Technology

[NRF-2016R1D1A1B01009235]

FX This study was supported by the Basic Science Research Program

(NRF-2016R1D1A1B01009235) of the National Research Foundation of Korea

funded by the Ministry of Education, Science, and Technology.

CR Cai SX, 2018, EXP CELL RES, V367, P37, DOI 10.1016/j.yexcr.2018.02.028

Cen DZ, 2019, ONCOTARGETS THER, V12, P1929, DOI 10.2147/OTT.S194463

Cheng Z, 2019, EUR J PHARMACOL, V853, P353, DOI 10.1016/j.ejphar.2019.03.040

Dastmalchi N, 2020, LIFE SCI, V259, DOI 10.1016/j.lfs.2020.118239

Deng Z, 2019, MOL MED REP, V20, P1057, DOI 10.3892/mmr.2019.10367

Figliuzzi M, 2013, BIOPHYS J, V104, P1203, DOI 10.1016/j.bpj.2013.01.012

Ham J, 2019, J GINSENG RES, V43, P625, DOI 10.1016/j.jgr.2019.02.004

Ham J, 2018, AM J CHINESE MED, V46, P1333, DOI 10.1142/S0192415X18500702

Hu X, 2020, EBIOMEDICINE, V56, DOI 10.1016/j.ebiom.2020.102800

Kang S, 2015, INT J ONCOL, V47, P1923, DOI 10.3892/ijo.2015.3164

Kim B.-M., 2014, EUR J CANCER PREV, V19, P23, DOI [10.15430/JCP.2014.19.1.23, DOI 10.15430/JCP.2014.19.1.23]

Kim Bo-Min, 2013, J Cancer Prev, V18, P177

Kim SJ, 2011, CANCER LETT, V302, P47, DOI 10.1016/j.canlet.2010.12.010

Phi LTH, 2019, ONCOTARGETS THER, V12, P10885, DOI 10.2147/OTT.S219063

Lee A, 2020, J GINSENG RES, V44, P300, DOI 10.1016/j.jgr.2019.01.003

Lee S, 2016, SCI REP-UK, V6, DOI 10.1038/srep30005

Li BQ, 2019, J CANCER RES THER, V15, P1642, DOI 10.4103/jcrt.JCRT_77_17

Li J, 2017, ONCOTARGET, V8, P53375, DOI 10.18632/oncotarget.18482

Li JL, 2019, EXP MOL PATHOL, V106, P131, DOI 10.1016/j.yexmp.2019.01.003

Li XX, 2020, BIOMED RES INT, V2020, DOI 10.1155/2020/8970340

Liu XL, 2019, MOL THER-ONCOLYTICS, V15, P248, DOI 10.1016/j.omto.2019.10.008

Lu JJ, 2020, CLIN EXP PHARMACOL P, V47, P1455, DOI 10.1111/1440-1681.13321

Mao XH, 2020, EVID-BASED COMPL ALT, V2020, DOI 10.1155/2020/6065124

Nakhjavani Maryam, 2019, Medicines (Basel), V6, DOI 10.3390/medicines6010017

Samukawa M, 2019, CEREBELLUM, V18, P76, DOI 10.1007/s12311-018-0955-0

Schmitt AM, 2016, CANCER CELL, V29, P452, DOI 10.1016/j.ccell.2016.03.010

Shen XY, 2020, CANCER MANAG RES, V12, P6807, DOI 10.2147/CMAR.S240000

Sun MY, 2017, INT J MOL MED, V39, P507, DOI 10.3892/ijmm.2017.2857

Tay Y, 2014, NATURE, V505, P344, DOI 10.1038/nature12986

Teng SY, 2017, MOL MED REP, V15, P2029, DOI 10.3892/mmr.2017.6255

Tian M, 2020, CHIN J NAT MEDICINES, V18, P526, DOI 10.1016/S1875-5364(20)30063-7

Vimalraj S, 2020, LIFE SCI, V256, DOI 10.1016/j.lfs.2020.118011

Wang NT, 2015, CLIN CANCER RES, V21, P4676, DOI 10.1158/1078-0432.CCR-14-3163

Wu JB, 2019, INT J BIOL SCI, V15, P1591, DOI 10.7150/ijbs.34113

Wu KR, 2019, AM J TRANSL RES, V11, P5776

Wu KM, 2013, CANCER RES, V73, P4488, DOI 10.1158/0008-5472.CAN-12-4078

Xiang YX, 2020, AGING-US, V12, P3156, DOI 10.18632/aging.102790

Xu HX, 2017, SCI REP-UK, V7, DOI 10.1038/s41598-017-04709-2

Xue LP, 2018, BIOCHEM BIOPH RES CO, V501, P827, DOI 10.1016/j.bbrc.2018.04.055

Yan L, 2020, ENVIRON TOXICOL, V35, P1015, DOI 10.1002/tox.22938

Yue X, 2020, CANCER MANAG RES, V12, P7539, DOI 10.2147/CMAR.S267665

Zheng X, 2018, CELL PHYSIOL BIOCHEM, V51, P1340, DOI 10.1159/000495552

Zhou K, 2019, ONCOTARGETS THER, V12, P7095, DOI 10.2147/OTT.S208329

Zou JF, 2020, J BIOCHEM MOL TOXIC, V34, DOI 10.1002/jbt.22480

NR 44

TC 11

Z9 13

U1 2

U2 9

PU MDPI

PI BASEL

PA ST ALBAN-ANLAGE 66, CH-4052 BASEL, SWITZERLAND

EI 2218-273X

J9 BIOMOLECULES

JI Biomolecules

PD JAN

PY 2021

VL 11

IS 1

AR 118

DI 10.3390/biom11010118

PG 13

WC Biochemistry & Molecular Biology

WE Science Citation Index Expanded (SCI-EXPANDED)

SC Biochemistry & Molecular Biology

GA PV2XT

UT WOS:000609856300001

PM 33477683

OA gold, Green Published

DA 2023-04-05

ER

PT J

AU Chu, Y

Zhang, WT

Kanimozhi, G

Brindha, GR

Tian, DF

AF Chu, Yan

Zhang, Wentao

Kanimozhi, G.

Brindha, G. R.

Tian, Defu

TI Ginsenoside Rg1 Induces Apoptotic Cell Death in Triple-Negative Breast

Cancer Cell Lines and Prevents Carcinogen-Induced Breast Tumorigenesis

in Sprague Dawley Rats

SO EVIDENCE-BASED COMPLEMENTARY AND ALTERNATIVE MEDICINE

LA English

DT Article

ID OXIDATIVE STRESS; DNA-DAMAGE; IN-VITRO; ACID; ANTIOXIDANT; MIGRATION;

SURVIVAL; INVASION; GROWTH

AB The objective of this study is to investigate the anticancer potential of ginsenoside Rg1 using in vitro and in vivo experimental models. In this study, we found that ginsenoside Rg1 induces cytotoxicity and apoptotic cell death through reactive oxygen species (ROS) generation and alterations in mitochondrial membrane potential (MMP) in the triple-negative breast cancer cells (MDA-MB-MD-231 cell lines). We found that ginsenoside Rg1 induces the formation of gamma H2AX foci, an indication of DNA damage, and subsequent TUNEL positive apoptotic nuclei in the MDA-MB-MD-231 cell lines. Further, we found that ginsenoside Rg1 prevents 7,12-dimethylbenz (a) anthracene (DMBA; 20 mg/rat) induced mammary gland carcinogenesis in experimental rats. We observed oral administration of ginsenoside Rg1 inhibited the DMBA-mediated tumor incidence, prevented the elevation of oxidative damage markers, and restored antioxidant enzymes near to normal. Furthermore, qRT-PCR gene expression studies revealed that ginsenoside Rg1 prevents the expression of markers associated with cell proliferation and survival, modulates apoptosis markers, downregulates invasion and angiogenesis markers, and regulates the EMT markers. Therefore, the present results suggest that ginsenoside Rg1 shows significant anticancer properties against breast cancer in experimental models.

C1 [Chu, Yan] Second Peoples Hosp Yunnan Prov, Dept Gen Surg, Kunming 650021, Yunnan, Peoples R China.

[Zhang, Wentao] Zhengzhou Univ, Dept Breast & Thyroid, Zhengzhou Cent Hosp Affiliated, Zhengzhou 450000, Henan, Peoples R China.

[Kanimozhi, G.] Dharmapuram Gnanambigai Govt Arts & Sci Coll Wome, Dept Biochem, Mayiladuthurai, Tamilnadu, India.

[Brindha, G. R.] SASTRA Deemed Univ, Sch Comp, Thanjavur 613401, Tamilnadu, India.

[Tian, Defu] Fourth Peoples Hosp Shaanxi, Dept Gen Surg, 512 Xianning East Rd, Xian 710043, Shaanxi, Peoples R China.

C3 Zhengzhou University; Shanmugha Arts, Science, Technology & Research

Academy (SASTRA)

RP Tian, DF (通讯作者)，Fourth Peoples Hosp Shaanxi, Dept Gen Surg, 512 Xianning East Rd, Xian 710043, Shaanxi, Peoples R China.

EM tiandefu_5@sina.com

CR Ahire V, 2017, NUTR CANCER, V69, P904, DOI 10.1080/01635581.2017.1339811

Akhdar H, 2012, TOPICS ON DRUG METABOLISM, P137

American Cancer Society, 2019, BREAST CANC FACTS FI

An IS, 2013, ONCOL REP, V29, P523, DOI 10.3892/or.2012.2136

Anjugam C., 2018, ASIAN J PHARM CLIN R, V11, DOI [10.22159/ajpcr.2018.v11i2.21652, DOI 10.22159/AJPCR.2018.V11I2.21652]

Attele AS, 1999, BIOCHEM PHARMACOL, V58, P1685, DOI 10.1016/S0006-2952(99)00212-9

Baskaran N, 2010, EUR J PHARMACOL, V637, P22, DOI 10.1016/j.ejphar.2010.03.054

Berardinis R. J. D., 2016, SCI ADV, V2, DOI 10.1126/sciadv.1600200

Casey SC, 2015, CARCINOGENESIS, V36, pS160, DOI 10.1093/carcin/bgv035

Chen TL, 2018, SAUDI J BIOL SCI, V25, P917, DOI 10.1016/j.sjbs.2018.01.012

Coleman ML, 2003, EMBO J, V22, P2036, DOI 10.1093/emboj/cdg189

Davison CA, 2013, CANCER RES, V73, P3704, DOI 10.1158/0008-5472.CAN-12-2482

Delahousse J, 2019, CANCER CHEMOTH PHARM, V84, P937, DOI 10.1007/s00280-019-03906-2

Deng YP, 2015, PLOS ONE, V10, DOI 10.1371/journal.pone.0135435

Djuzenova CS, 2013, RADIAT ONCOL, V8, DOI 10.1186/1748-717X-8-98

Espinosa-Diez C, 2015, REDOX BIOL, V6, P183, DOI 10.1016/j.redox.2015.07.008

Etti IC, 2017, PLOS ONE, V12, DOI 10.1371/journal.pone.0182357

Fang F, 2012, BBA-MOL BASIS DIS, V1822, P286, DOI 10.1016/j.bbadis.2011.10.004

Farhan M, 2016, TOXINS, V8, DOI 10.3390/toxins8020037

Gao HW, 2020, PHYTOMEDICINE, V69, DOI 10.1016/j.phymed.2020.153197

George BP, 2019, OXID MED CELL LONGEV, V2019, DOI 10.1155/2019/6797921

Giustarini D, 2017, FREE RADICAL BIO MED, V112, P360, DOI 10.1016/j.freeradbiomed.2017.08.008

He F, 2020, FRONT PHARMACOL, V10, DOI 10.3389/fphar.2019.01565

Hecht F, 2016, TUMOR BIOL, V37, P4281, DOI 10.1007/s13277-016-4873-9

Hugo H, 2007, J CELL PHYSIOL, V213, P374, DOI 10.1002/jcp.21223

JIANG ZY, 1992, ANAL BIOCHEM, V202, P384, DOI 10.1016/0003-2697(92)90122-N

Jing XM, 2019, MOL CANCER, V18, DOI 10.1186/s12943-019-1089-9

Juang YL, 2016, MOL CARCINOGEN, V55, P2247, DOI 10.1002/mc.22465

Kanimozhi G., 2012, Biomedicine & Preventive Nutrition, V2, P186

Karthikeyan R, 2018, LIFE SCI, V212, P150, DOI 10.1016/j.lfs.2018.10.004

Korivi M, 2012, EVID-BASED COMPL ALT, V2012, DOI 10.1155/2012/932165

Li BH, 2011, CANCER LETT, V301, P185, DOI 10.1016/j.canlet.2010.11.015

Li L, 2014, ONCOL REP, V32, P1779, DOI 10.3892/or.2014.3422

Li QF, 2008, INT J BIOCHEM CELL B, V40, P1918, DOI 10.1016/j.biocel.2008.01.031

Lien EC, 2016, NAT CELL BIOL, V18, P572, DOI 10.1038/ncb3341

Lind, 2020, MEDICINE, V48, P90, DOI DOI 10.1016/J.MPMED.2019.11.005

Liu ZQ, 2003, J AGR FOOD CHEM, V51, P2555, DOI 10.1021/jf026228i

Long J, 2018, AM J CANCER RES, V8, P778

Lugano R, 2020, CELL MOL LIFE SCI, V77, P1745, DOI 10.1007/s00018-019-03351-7

Minari J, 2014, EGYPT J MED HUM GENE, V15, P327, DOI DOI 10.1016/J.EJMHG.2014.05.001

Muzaffer U, 2018, BIOCHEM BIOPHYS REP, V13, P109, DOI 10.1016/j.bbrep.2018.01.004

Nag SA, 2012, FRONT PHARMACOL, V3, DOI 10.3389/fphar.2012.00025

Ni XJ, 2018, BRAZ J MED BIOL RES, V51, DOI [10.1590/1414-431X20176611, 10.1590/1414-431x20176611]

Ogata K, 2010, J CLIN PHARMACOL, V50, P743, DOI 10.1177/0091270009351883

Oh JM, 2019, INT J MOL SCI, V20, DOI 10.3390/ijms20174279

Ong WY, 2015, FRONT AGING NEUROSCI, V7, DOI 10.3389/fnagi.2015.00129

PC Registry, 2019, GLOBAL CANC OBSERVAT

Pietenpol JA, 2002, TOXICOLOGY, V181, P475, DOI 10.1016/S0300-483X(02)00460-2

Radad K, 2004, J NEURAL TRANSM, V111, P37, DOI 10.1007/s00702-003-0063-1

Ramachandran S, 2008, CHEM-BIOL INTERACT, V176, P99, DOI 10.1016/j.cbi.2008.08.010

Sharmila R, 2012, INDIAN J EXP BIOL, V50, P187

Shi DD, 2019, MOL NEUROBIOL, V56, P5626, DOI 10.1007/s12035-019-1474-9

Sun MY, 2017, INT J MOL MED, V39, P507, DOI 10.3892/ijmm.2017.2857

Sung H, 2021, CA-CANCER J CLIN, V71, P209, DOI [10.3322/caac.21492, 10.3322/caac.21660, 10.3322/caac.21442]

Suresh K, 2010, PHARMACOL REP, V62, P1178, DOI 10.1016/S1734-1140(10)70380-7

Wang Chong-Zhi, 2015, Diseases, V3, P193

Wang W, 2007, CANCER CHEMOTH PHARM, V59, P589, DOI 10.1007/s00280-006-0300-z

Welm AL, 2005, P NATL ACAD SCI USA, V102, P4324, DOI 10.1073/pnas.0500470102

Weydert CJ, 2010, NAT PROTOC, V5, P51, DOI 10.1038/nprot.2009.197

Xu YS, 2018, BIOL PHARM BULL, V41, P1638, DOI 10.1248/bpb.b18-00132

Xu ZM, 2019, J CELL BIOCHEM, V120, P18388, DOI 10.1002/jcb.29150

Yang C. C., 2017, RES REPORTS CHEM, V1, P1

Zeb A, 2016, J ANAL METHODS CHEM, V2016, DOI 10.1155/2016/9412767

Zhang J, 2015, DRUG METAB DISPOS, V43, P1181, DOI 10.1124/dmd.115.063800

Zhang X, 2012, NEUROSCIENCE, V220, P191, DOI 10.1016/j.neuroscience.2012.06.027

Zhu C, 2017, ONCOTARGET, V8, P83792, DOI 10.18632/oncotarget.19698

Zorov DB, 2014, PHYSIOL REV, V94, P909, DOI 10.1152/physrev.00026.2013

NR 67

TC 9

Z9 9

U1 3

U2 12

PU HINDAWI LTD

PI LONDON

PA ADAM HOUSE, 3RD FLR, 1 FITZROY SQ, LONDON, W1T 5HF, ENGLAND

SN 1741-427X

EI 1741-4288

J9 EVID-BASED COMPL ALT

JI Evid.-based Complement Altern. Med.

PD OCT 23

PY 2020

VL 2020

AR 8886955

DI 10.1155/2020/8886955

PG 12

WC Integrative & Complementary Medicine

WE Science Citation Index Expanded (SCI-EXPANDED)

SC Integrative & Complementary Medicine

GA OU5EH

UT WOS:000591551000003

PM 33178325

OA gold, Green Published

DA 2023-04-05

ER

PT J

AU Song, JH

Eum, DY

Park, SY

Jin, YH

Shim, JW

Park, SJ

Kim, MY

Park, SJ

Heo, K

Choi, YJ

AF Song, Joong-Hyun

Eum, Da-Young

Park, Soon-Yong

Jin, Yun-Ho

Shim, Jae-Woong

Park, Shin-Ji

Kim, Min-Young

Park, Seong-Jun

Heo, Kyu

Choi, Yoo-Jin

TI Inhibitory effect of ginsenoside Rg3 on cancer stemness and mesenchymal

transition in breast cancer via regulation of myeloid-derived suppressor

cells

SO PLOS ONE

LA English

DT Article

ID CROSS-TALK; ACTIVATION; STAT3

AB Ginsenoside Rg3 (Rg3) has been studied in several cancer models and is suggested to act through various pharmacological effects. We investigated the anticancer properties of Rg3 through myeloid-derived suppressor cell (MDSC) modulation in FM3A mouse mammary carcinoma cells. The effects of Rg3 on MDSCs and consequent changes in cancer stem-like cells (CSCs) and epithelial-mesenchymal transition (EMT) were evaluated by diverse methods. MDSCs promoted cancer by enhancing breast cancer stemness and promoting EMT. Rg3 at a dose without obvious cytotoxicity downregulated MDSCs and repressed MDSC-induced cancer stemness and EMT. Mechanistic investigations suggested that these inhibitory effects of Rg3 on MDSCs and corresponding cancer progression depend upon suppression of the STAT3-dependent pathway, tumor-derived cytokines, and the NOTCH signaling pathway. In a mouse model, MDSCs accelerated tumor progression, and Rg3 delayed tumor growth, which is consistent with the results ofin vitroexperiments. These results indicated that Rg3 could effectively inhibit the progression of breast cancer. The anticancer effect of Rg3 might be partially due to its downregulation of MDSCs and consequent repression of cancer stemness and EMT in breast cancer. Hence, we suggest the regulation of MDSCs through Rg3 treatment as an effective therapeutic strategy for breast cancer patients.

C1 [Song, Joong-Hyun; Eum, Da-Young; Park, Soon-Yong; Jin, Yun-Ho; Shim, Jae-Woong; Park, Shin-Ji; Kim, Min-Young; Park, Seong-Jun; Heo, Kyu; Choi, Yoo-Jin] Dongnam Inst Radiol & Med Sci, Dept Res Ctr, Busan, South Korea.

C3 Korea Institute of Radiological & Medical Sciences

RP Choi, YJ (通讯作者)，Dongnam Inst Radiol & Med Sci, Dept Res Ctr, Busan, South Korea.

EM cyj5325@dirams.re.kr

RI jin, yun/GQZ-6618-2022

OI Heo, Kyu/0000-0001-8833-4731

FU National Research Foundation of Korea (DIRAMS) grant - Korea government

(MSIP) [50590-2020]

FX This work was supported by the National Research Foundation of Korea

(DIRAMS) grant funded by the Korea government (MSIP) (50590-2020). The

funders had no role in study design, data collection and analysis,

decision to publish, or preparation of the manuscript.

CR Almand B, 2000, CLIN CANCER RES, V6, P1755

[Anonymous], 2019, QUALITY MANAGEMENT, V11, pS1007

[Anonymous], 2005, PSYCHOL HEALTH MED, V5, P263

Chalmin F, 2010, J CLIN INVEST, V120, P457, DOI 10.1172/JCI40483

Chen XP, 2011, INT J CLIN ONCOL, V16, P519, DOI 10.1007/s10147-011-0222-6

Corzo CA, 2009, J IMMUNOL, V182, P5693, DOI 10.4049/jimmunol.0900092

Cui TX, 2013, IMMUNITY, V39, P611, DOI 10.1016/j.immuni.2013.08.025

DeNardo DG, 2009, CANCER CELL, V16, P91, DOI 10.1016/j.ccr.2009.06.018

Diaz-Montero CM, 2009, CANCER IMMUNOL IMMUN, V58, P49, DOI 10.1007/s00262-008-0523-4

Du R, 2008, CANCER CELL, V13, P206, DOI 10.1016/j.ccr.2008.01.034

Fukuda A, 2011, CANCER CELL, V19, P441, DOI 10.1016/j.ccr.2011.03.002

Gabrilovich DI, 2017, CANCER IMMUNOL RES, V5, P3, DOI 10.1158/2326-6066.CIR-16-0297

Gabrilovich DI, 2009, NAT REV IMMUNOL, V9, P162, DOI 10.1038/nri2506

Ham SW, 2019, J ETHNOPHARMACOL, V236, P393, DOI 10.1016/j.jep.2019.03.031

Jeon C, 2011, J GINSENG RES, V35, P462, DOI 10.5142/jgr.2011.35.4.462

Kim HS, 2004, ARCH PHARM RES, V27, P429, DOI 10.1007/BF02980085

Lesina M, 2011, CANCER CELL, V19, P456, DOI 10.1016/j.ccr.2011.03.009

Ostrand-Rosenberg S, 2018, J IMMUNOL, V200, P422, DOI 10.4049/jimmunol.1701019

Ostrand-Rosenberg S, 2012, SEMIN CANCER BIOL, V22, P275, DOI 10.1016/j.semcancer.2012.01.011

Panni RZ, 2014, CANCER IMMUNOL IMMUN, V63, P513, DOI 10.1007/s00262-014-1527-x

Park D, 2011, ENVIRON TOXICOL PHAR, V31, P397, DOI 10.1016/j.etap.2011.01.008

Peng DJ, 2016, CANCER RES, V76, P3156, DOI 10.1158/0008-5472.CAN-15-2528

Reya T, 2001, NATURE, V414, P105, DOI 10.1038/35102167

Sica A, 2017, CANCER IMMUNOL IMMUN, V66, P1025, DOI 10.1007/s00262-017-1997-8

Siegel Rebecca L, 2019, CA Cancer J Clin, V69, P7, DOI 10.3322/caac.21551

Sun MY, 2017, INT J MOL MED, V39, P507, DOI 10.3892/ijmm.2017.2857

Tang YC, 2018, INT J ONCOL, V52, P127, DOI 10.3892/ijo.2017.4183

Toor SM, 2017, CANCER IMMUNOL IMMUN, V66, P753, DOI 10.1007/s00262-017-1977-z

Veglia F, 2018, NAT IMMUNOL, V19, P108, DOI 10.1038/s41590-017-0022-x

Wang JJ, 2018, CANCER LETT, V415, P73, DOI 10.1016/j.canlet.2017.11.037

Weigelt B, 2005, NAT REV CANCER, V5, P591, DOI 10.1038/nrc1670

Xu TM, 2008, CHINESE MED J-PEKING, V121, P1394, DOI 10.1097/00029330-200808010-00012

Yang L, 2004, CANCER CELL, V6, P409, DOI 10.1016/j.ccr.2004.08.031

Yang L, 2008, CANCER CELL, V13, P23, DOI 10.1016/j.ccr.2007.12.004

2018, CURR OPIN TOXICOL, V10

NR 35

TC 11

Z9 12

U1 1

U2 9

PU PUBLIC LIBRARY SCIENCE

PI SAN FRANCISCO

PA 1160 BATTERY STREET, STE 100, SAN FRANCISCO, CA 94111 USA

SN 1932-6203

J9 PLOS ONE

JI PLoS One

PD OCT 22

PY 2020

VL 15

IS 10

AR e0240533

DI 10.1371/journal.pone.0240533

PG 15

WC Multidisciplinary Sciences

WE Science Citation Index Expanded (SCI-EXPANDED)

SC Science & Technology - Other Topics

GA ON4AZ

UT WOS:000586647200043

PM 33091036

OA gold, Green Published

DA 2023-04-05

ER

PT J

AU Xia, XJ

Tao, J

Ji, ZW

Long, CC

Hu, Y

Zhao, ZY

AF Xia, Xiaojing

Tao, Jin

Ji, Zhuwa

Long, Chencheng

Hu, Ying

Zhao, Zhiying

TI Increased antitumor efficacy of ginsenoside Rh-2 via mixed micelles: in

vivo and in vitro evaluation

SO DRUG DELIVERY

LA English

DT Article

DE Ginsenoside Rh2; mixed micelles; Solutol(R) HS15; TPGS; A549 cell;

antitumor

ID HYALURONIC-ACID; P-GLYCOPROTEIN; BREAST-CANCER; COMPOUND K; RH2; TPGS;

DELIVERY; NANOPARTICLES; LIPOSOMES; HS15

AB The aim of this work is to apply Solutol(R)HS15 and TPGS to prepare self-assembled micelles loading with ginsenoside Rh(2)to increase the solubility of ginsenoside Rh-2, hence, improving the antitumor efficacy. Ginsenoside Rh-2-mixed micelles (Rh-2-M) were prepared by thin film dispersion method. The optimal Rh-2-M was characterized by particle size, morphology, and drug encapsulation efficiency. The enhancement ofin vivoanti-tumor efficacy of Rh-2-M was evaluated by nude mice bearing tumor model. The solubility of Rh(2)in self-assembled micelles was increased approximately 150-folds compared to free Rh-2.In vitroresults demonstrated that the particle size of Rh-2-M is 74.72 +/- 2.63 nm(PDI = 0.147 +/- 0.15), and the morphology of Rh-2-M is spherical or spheroid, and the EE% and LE% are 95.27 +/- 1.26% and 7.68 +/- 1.34%, respectively. The results ofin vitrocell uptake andin vivoimaging showed that Rh-2-M could not only increase the cell uptake of drugs, but also transport drug to tumor sites, prolonging the retention time.In vitrocytotoxicity andin vivoantitumor results showed that the anti-tumor effect of Rh(2)can be effectively improved by Rh-2-M. Therefore, Solutol(R)HS15 and TPGS could be used to entrapping Rh(2)into micelles, enhancing solubility and antitumor efficacy.

C1 [Xia, Xiaojing; Tao, Jin; Ji, Zhuwa; Long, Chencheng; Hu, Ying] Zhejiang Pharmaceut Coll, Dept Pharmaceut, 888 East Yinxian Rd, Ningbo 315100, Peoples R China.

[Zhao, Zhiying] China Pharmaceut Univ, Dept Tradit Chinese Med, Nanjing, Peoples R China.

C3 China Pharmaceutical University

RP Hu, Y (通讯作者)，Zhejiang Pharmaceut Coll, Dept Pharmaceut, 888 East Yinxian Rd, Ningbo 315100, Peoples R China.; Zhao, ZY (通讯作者)，China Pharmaceut Univ, Dept Tradit Chinese Med, Coll Tradit Chinese Pharm, 639 Longmian Ave, Nanjing 211198, Peoples R China.

EM pharmhawk@126.com; zhaozhiying608@126.com

FU National Natural Science Foundation of China [81803462]; Natural Science

Foundation of Ningbo [2018A610433]

FX This work was supported by the National Natural Science Foundation of

China under Grant [81803462] and the Natural Science Foundation of

Ningbo under Grant [2018A610433].

CR An IS, 2013, ONCOL REP, V29, P523, DOI 10.3892/or.2012.2136

Chen DQ, 2014, ARTIF CELL NANOMED B, V42, P205, DOI 10.3109/21691401.2013.794358

Chen WW, 2015, CELL BIOCHEM BIOPHYS, V72, P325, DOI 10.1007/s12013-014-0456-9

Chen Y, 2018, J CELL BIOCHEM, V119, P6527, DOI 10.1002/jcb.26716

Choudhury H, 2017, INT J PHARMACEUT, V529, P506, DOI 10.1016/j.ijpharm.2017.07.018

Chu JMT, 2014, NEUROPHARMACOLOGY, V85, P215, DOI 10.1016/j.neuropharm.2014.05.029

Collnot EM, 2007, MOL PHARM, V4, P465, DOI 10.1021/mp060121r

de Souza JB, 2019, PHARM DEV TECHNOL, V24, P283, DOI 10.1080/10837450.2018.1472610

Dintaman JM, 1999, PHARMACEUT RES, V16, P1550, DOI 10.1023/A:1015000503629

Gao L, 2016, COLLOID SURFACE B, V140, P307, DOI 10.1016/j.colsurfb.2015.12.057

Gu Y, 2009, FOOD CHEM TOXICOL, V47, P2257, DOI 10.1016/j.fct.2009.06.013

Honary S, 2013, TROP J PHARM RES, V12, P255, DOI 10.4314/tjpr.v12i2.19

Hou J, 2016, DRUG DELIV, V23, P3248, DOI 10.3109/10717544.2016.1167270

Hou J, 2016, INT J PHARMACEUT, V512, P186, DOI 10.1016/j.ijpharm.2016.08.045

Hulkower Keren I, 2011, Pharmaceutics, V3, P107, DOI 10.3390/pharmaceutics3010107

Jiang TY, 2012, BIOMATERIALS, V33, P9246, DOI 10.1016/j.biomaterials.2012.09.027

Jin X, 2017, NANOMED-NANOTECHNOL, V13, P1105, DOI 10.1016/j.nano.2016.10.017

Jin X, 2016, INT J PHARMACEUT, V515, P359, DOI 10.1016/j.ijpharm.2016.10.027

Kim SY, 2007, BIOCHEM PHARMACOL, V74, P1642, DOI 10.1016/j.bcp.2007.08.015

Kim YJ, 2019, INT J NANOMED, V14, P8195, DOI 10.2147/IJN.S221328

Lai JR, 2010, J APPL PHYS, V107, DOI 10.1063/1.3357344

Lee H, 2018, J GINSENG RES, V42, P455, DOI 10.1016/j.jgr.2017.05.003

Li P, 2017, DRUG DELIV, V24, P834, DOI 10.1080/10717544.2017.1326540

Li Shaoyi, 2015, Tumour Biol

Liu TF, 2018, POLYM CHEM-UK, V9, P1827, DOI 10.1039/c8py00344k

Lo SH, 2017, INT J MOL SCI, V18, DOI 10.3390/ijms18071364

Patel J, 2011, INT J PHARM INVESTIG, V1, P112, DOI 10.4103/2230-973X.82431

Quandt D, 2014, J TRANSL MED, V12, DOI 10.1186/1479-5876-12-151

Rivolta I, 2011, J PHYSIOL PHARMACOL, V62, P45

Shaji Jessy, 2016, Pharm Nanotechnol, V4, P167, DOI 10.2174/2211738504666160720162323

Singh P, 2017, INT J NANOMED, V12, P4073, DOI 10.2147/IJN.S125154

Song ZM, 2014, INT J PHARMACEUT, V471, P312, DOI 10.1016/j.ijpharm.2014.05.059

Wang M, 2017, ONCOL LETT, V13, P681, DOI 10.3892/ol.2016.5490

Wang WJ, 2019, FRONT CHEM, V7, DOI 10.3389/fchem.2019.00093

Wen Xu, 2015, Asian Pac J Cancer Prev, V16, P1105

Williams AJ, 2018, INT J PHARMACEUT, V535, P113, DOI 10.1016/j.ijpharm.2017.10.029

Yang FF, 2017, DRUG DELIV TRANSL RE, V7, P731, DOI 10.1007/s13346-017-0402-7

Yang L, 2017, INT J NANOMED, V12, P7653, DOI 10.2147/IJN.S144305

Yang L, 2016, J PHARM PHARMACOL, V68, P1109, DOI 10.1111/jphp.12590

Yang ZQ, 2016, SCI REP-UK, V6, DOI 10.1038/srep19383

Zare-Zardini H, 2018, SCI REP-UK, V8, DOI 10.1038/s41598-017-18938-y

Zhang XF, 2014, J MATER CHEM B, V2, P4034, DOI 10.1039/c4tb00439f

Zhuang JJ, 2018, NUTRIENTS, V10, DOI 10.3390/nu10030328

NR 43

TC 3

Z9 4

U1 3

U2 17

PU TAYLOR & FRANCIS LTD

PI ABINGDON

PA 2-4 PARK SQUARE, MILTON PARK, ABINGDON OR14 4RN, OXON, ENGLAND

SN 1071-7544

EI 1521-0464

J9 DRUG DELIV

JI Drug Deliv.

PD JAN 1

PY 2020

VL 27

IS 1

BP 1369

EP 1377

DI 10.1080/10717544.2020.1825542

PG 9

WC Pharmacology & Pharmacy

WE Science Citation Index Expanded (SCI-EXPANDED)

SC Pharmacology & Pharmacy

GA NU9CQ

UT WOS:000573934200001

PM 32998576

OA gold, Green Published

DA 2023-04-05

ER

PT J

AU He, LH

Wang, XR

Ma, Q

Zhao, WP

Jia, YS

Dong, GL

Zhu, YH

Jia, XC

Tong, ZS

AF He, Lihong

Wang, Xiaorui

Ma, Qing

Zhao, Weipeng

Jia, Yongsheng

Dong, Guolei

Zhu, Yuehong

Jia, Xiaochen

Tong, Zhongsheng

TI Ginsenoside induces cell death in breast cancer cells via ROS/PI3K/Akt

signaling pathway

SO TROPICAL JOURNAL OF PHARMACEUTICAL RESEARCH

LA English

DT Article

DE Breast cancer cells; Ginsenoside; Apoptosis; ROS/PI3K/Akt signaling

pathway

ID PI3K/AKT; APOPTOSIS

AB Purpose: To study the influence of ginsenoside on breast carcinoma, and the mechanism of action involved.

Methods: Different concentrations of ginsenoside were used to treat MCF-7 breast cancer cell line. Cell viability was measured by MTT assay, while protein expressions of p-Akt and p-PI3K were determined using Western blotting. The concentrations of reactive oxidative reactants and reactive oxygen species (ROS) were assessed using fluorescence immunoassay and immunofiuorescence assay. The mechanism of action involved in ginsenoside-mediated apoptosis was determined based on ROS/PI3K/Akt signaling pathway.

Results: There was no change in the inhibition of MCF-7 cell proliferation in control cells with time (p > 0.05). However, inhibition of MCF-7 cell proliferation in ginsenoside group was significantly higher than that in the control group (p < 0.05); furthermore, it increased with time and ginsenoside concentration. Apoptosis was markedly and concentration-dependently higher in ginsenoside-treated MCF-7 cells than in controls (p > 0.05). There were lower protein levels of p-PI3K and p-Akt in ginsenoside-exposed MCF-7 cells than in control group; the protein expressions decreased with increase in ginsenoside concentration (p < 0.05). The expressions of ROS in ginsenoside-treated MCF-7 cells declined, relative to the untreated group; in addition, the expressions decreased with increase in ginsenoside concentration (p < 0.05).

Conclusion: Ginsenoside suppresses proliferation of MCF-7 cell line, and exerts apoptotic effect on the cells via inhibition of the ROS/PI3K/Akt signal pathway. This provides a new approach to treat breast cancer.

C1 [He, Lihong; Wang, Xiaorui; Zhao, Weipeng; Jia, Yongsheng; Dong, Guolei; Zhu, Yuehong; Jia, Xiaochen; Tong, Zhongsheng] Tianjin Med Univ, Dept Breast Canc, Canc Inst & Hosp, Tianjin, Peoples R China.

[Ma, Qing] Tianjin Med Univ, Dept Oncol, Gen Hosp, Tianjin, Peoples R China.

C3 Tianjin Medical University; Tianjin Medical University

RP Tong, ZS (通讯作者)，Tianjin Med Univ, Dept Breast Canc, Canc Inst & Hosp, Tianjin, Peoples R China.

EM msst19@163.com

FU Science and Technology Fundation of Tianjin Municipal Health and Health

Committee [2015KZ089]; Anticancer Key Technologies R&D Program of

Tianjin [12ZCDZSY16200]; Natural Science Foundation of Tianjin

[18JCYBJC91600]

FX This study was supported by Science and Technology Fundation of Tianjin

Municipal Health and Health Committee (no. 2015KZ089), Anticancer Key

Technologies R&D Program of Tianjin (no. 12ZCDZSY16200), and Natural

Science Foundation of Tianjin (no. 18JCYBJC91600).

CR Camp E, 2017, BONE, V94, P98, DOI 10.1016/j.bone.2016.09.019

Chen XB, 2017, REDOX BIOL, V12, P987, DOI 10.1016/j.redox.2017.04.031

Cong BB, 2016, ONCOTARGET, V7, P41996, DOI 10.18632/oncotarget.9634

Guo CH, 2019, ACTA MEDICA MEDITERR, V35, P2625, DOI 10.19193/0393-6384_2019_5_412

Jin YQ, 2017, N-S ARCH PHARMACOL, V390, P1

Karaman S, 2019, ACTA MEDICA MEDITERR, V35, P3269, DOI 10.19193/0393-6384_2019_6_514

Liu AL, 2016, INT J CHRONIC OBSTR, V11, P1721, DOI 10.2147/COPD.S107396

Msaki A, 2016, ONCOTARGET, V7, P33081, DOI 10.18632/oncotarget.8859

Nguyen NM, 2016, CANCER RES, V76, DOI 10.1158/1538-7445.AM2016-901

Pingwara R, 2017, J PHYSIOL PHARMACOL, V68, P573

Prasad A, 2016, ONCOTARGET, V7, P79374, DOI 10.18632/oncotarget.12692

Rybicka A, 2016, J PHYSIOL PHARMACOL, V67, P491

Song M, 2016, CELL MOL BIOL, V62, P50, DOI 10.14715/cmb/2016.62.6.9

Telo S, 2017, IRAN J PHARM RES, V16, P164

Verma V, 2016, INT J RADIAT ONCOL, V95, P617, DOI 10.1016/j.ijrobp.2016.01.058

Xuan NT, 2016, J RECEPT SIG TRANSD, V37, P1

[于宁 Yu Ning], 2016, [中国病理生理杂志, Chinese Journal of Pathophysiology], V32, P1370

NR 17

TC 4

Z9 4

U1 6

U2 23

PU PHARMACOTHERAPY GROUP

PI BENIN CITY

PA UNIV BENIN, FACULTY PHARMACY, BENIN CITY, 00000, NIGERIA

SN 1596-5996

J9 TROP J PHARM RES

JI Trop. J. Pharm. Res.

PD AUG

PY 2020

VL 19

IS 8

BP 1631

EP 1636

DI 10.4314/tjpr.v19i8.10

PG 6

WC Pharmacology & Pharmacy

WE Science Citation Index Expanded (SCI-EXPANDED)

SC Pharmacology & Pharmacy

GA NG9ZL

UT WOS:000564339800010

OA gold

DA 2023-04-05

ER

PT J

AU Jin, Y

Huynh, DTN

Nguyen, TLL

Jeon, H

Heo, KS

AF Jin, Yujin

Huynh, Diem Thi Ngoc

Nguyen, Thuy Le Lam

Jeon, Hyesu

Heo, Kyung-Sun

TI Therapeutic effects of ginsenosides on breast cancer growth and

metastasis

SO ARCHIVES OF PHARMACAL RESEARCH

LA English

DT Review

DE Breast cancer; Cell growth; Ginsenoside; Metastasis;

Epithelial-mesenchymal transition; Cancer resistance

ID EPITHELIAL-MESENCHYMAL TRANSITION; CELL-CYCLE; KAPPA-B; EPIGENETIC

REGULATION; DRUG-DELIVERY; IN-VITRO; NANOPARTICLES; PROLIFERATION;

APOPTOSIS; INHIBITION

AB Breast cancer is the most common cause of cancer-related deaths among women worldwide. Thus, the development of new and effective low-toxicity drugs is vital. The specific characteristics of breast cancer have allowed for the development of targeted therapy towards each breast cancer subtype. Nevertheless, increasing drug resistance is displayed by the changing phenotype and microenvironments of the tumor through mutation or dysregulation of various mechanisms. Recently, emerging data on the therapeutic potential of biocompounds isolated from ginseng have been reported. Therefore, in this review, various roles of ginsenosides in the treatment of breast cancer, including apoptosis, autophagy, metastasis, epithelial-mesenchymal transition, epigenetic changes, combination therapy, and drug delivery system, have been discussed.

C1 [Jin, Yujin; Huynh, Diem Thi Ngoc; Nguyen, Thuy Le Lam; Jeon, Hyesu; Heo, Kyung-Sun] Chungnam Natl Univ, Dept Pharmacol, Coll Pharm, Daejeon, South Korea.

[Heo, Kyung-Sun] Chungnam Natl Univ, Inst Drug Res & Dev, Daejeon, South Korea.

C3 Chungnam National University; Chungnam National University

RP Heo, KS (通讯作者)，Chungnam Natl Univ, Dept Pharmacol, Coll Pharm, Daejeon, South Korea.; Heo, KS (通讯作者)，Chungnam Natl Univ, Inst Drug Res & Dev, Daejeon, South Korea.

EM kheo@cnu.ac.kr

OI Huynh, Diem Thi Ngoc/0000-0003-1822-5518; Heo,

Kyung-Sun/0000-0003-3800-7665

FU National Research Foundation of Korea [KNRF-2016232004]

FX This research was supported by National Research Foundation of Korea

(KNRF-2016232004 and -2019025901).

CR Abdel-Hafiz HA, 2015, EPIGENOMICS-UK, V7, P847, DOI 10.2217/epi.15.10

Balanis N, 2017, MOL CELL ENDOCRINOL, V451, P24, DOI 10.1016/j.mce.2017.01.013

Chaudhary P, 2019, ARCH PHARM RES, V42, P1040, DOI 10.1007/s12272-019-01195-0

Chen L, 2018, ARCH PHARM RES, V41, P101, DOI 10.1007/s12272-017-0980-4

Chen WW, 2019, J ASIAN NAT PROD RES, V21, P742, DOI 10.1080/10286020.2018.1490273

Cho ES, 2019, ARCH PHARM RES, V42, P14, DOI 10.1007/s12272-018-01108-7

Choi S, 2011, J CELL BIOCHEM, V112, P330, DOI 10.1002/jcb.22932

Choi S, 2009, PHARM RES-DORDR, V26, P2280, DOI 10.1007/s11095-009-9944-9

Christensen LP, 2009, ADV FOOD NUTR RES, V55, P1, DOI 10.1016/S1043-4526(08)00401-4

Chung Y, 2018, ANIM CELLS SYST, V22, P382, DOI 10.1080/19768354.2018.1545696

Cong ZY, 2020, PHARMACOLOGY, V105, P329, DOI 10.1159/000503821

Cui L, 2018, ARCH PHARM RES, V41, P299, DOI 10.1007/s12272-017-0990-2

Huynh DTN, 2020, BIOCHEM BIOPH RES CO, V523, P267, DOI 10.1016/j.bbrc.2019.12.053

Huynh DTN, 2019, ARCH PHARM RES, V42, P848, DOI 10.1007/s12272-019-01180-7

Dong B, 2017, CELL MOL BIOL, V63, P1, DOI 10.14715/cmb/2017.63.8.1

Dong YN, 2019, INT J NANOMED, V14, P6971, DOI 10.2147/IJN.S210882

Duan ZG, 2018, BIOCHEM BIOPH RES CO, V499, P482, DOI 10.1016/j.bbrc.2018.03.174

Elzoghby AO, 2012, J CONTROL RELEASE, V157, P168, DOI 10.1016/j.jconrel.2011.07.031

Escriva-de-Romani S, 2018, BREAST, V39, P80, DOI 10.1016/j.breast.2018.03.006

Feng Y, 2018, J PHARMACOL SCI, V138, P96, DOI 10.1016/j.jphs.2018.08.004

Gao H, 2020, BIOMED PHARMACOTHER, V124, DOI 10.1016/j.biopha.2020.109891

Gao QR, 2018, CELL PROLIFERAT, V51, DOI 10.1111/cpr.12438

Ge GQ, 2017, BIOL PHARM BULL, V40, P2117, DOI 10.1248/bpb.b17-00463

Gong J, 2017, CELL REP, V21, P1968, DOI 10.1016/j.celrep.2017.10.070

Gu MQ, 2018, MATURITAS, V114, P60, DOI 10.1016/j.maturitas.2018.06.004

Ham J, 2019, J GINSENG RES, V43, P625, DOI 10.1016/j.jgr.2019.02.004

Ham J, 2018, AM J CHINESE MED, V46, P1333, DOI 10.1142/S0192415X18500702

Hong C, 2019, THERANOSTICS, V9, P4437, DOI 10.7150/thno.34953

Hong YN, 2019, TOXICOLOGY, V418, P22, DOI 10.1016/j.tox.2019.02.010

Hou JG, 2019, INT J MOL SCI, V20, DOI 10.3390/ijms20051244

Jaenisch R, 2003, NAT GENET, V33, P245, DOI 10.1038/ng1089

Jeong D, 2019, AM J CHINESE MED, V47, P1643, DOI 10.1142/S0192415X19500848

Ji XW, 2019, BIOMED PHARMACOTHER, V114, DOI 10.1016/j.biopha.2019.108800

Jimenez-Gardnno AM, 2017, BIOCHEM BIOPH RES CO, V490, P780, DOI 10.1016/j.bbrc.2017.06.117

Jin X, 2017, J BIOL CHEM, V292, P6269, DOI 10.1074/jbc.M116.764407

Jin Yujin, 2019, Journal of Bacteriology and Virology, V49, P221

Jin Y, 2019, BMB REP, V52, P706, DOI 10.5483/BMBRep.2019.52.12.234

Jin Yujin, 2018, Journal of Bacteriology and Virology, V48, P156, DOI 10.4167/jbv.2018.48.4.156

Jung J, 2019, ARCH PHARM RES, V42, P815, DOI 10.1007/s12272-019-01132-1

Kang JH, 2011, PLANT FOOD HUM NUTR, V66, P298, DOI 10.1007/s11130-011-0242-4

Kim Bo-Min, 2014, J Cancer Prev, V19, P23

Kim BJ, 2013, J GINSENG RES, V37, P201, DOI 10.5142/jgr.2013.37.201

Kim DH, 2018, J GINSENG RES, V42, P255, DOI 10.1016/j.jgr.2017.04.011

Kim H, 2018, J GINSENG RES, V42, P361

Kim JH, 2017, J GINSENG RES, V41, P435, DOI 10.1016/j.jgr.2016.08.004

Kim SJ, 2015, J GINSENG RES, V39, P125, DOI 10.1016/j.jgr.2014.09.003

Kim YJ, 2019, INT J NANOMED, V14, P8195, DOI 10.2147/IJN.S221328

Klutstein M, 2016, CANCER RES, V76, P3446, DOI 10.1158/0008-5472.CAN-15-3278

Lee H, 2018, J GINSENG RES, V42, P455, DOI 10.1016/j.jgr.2017.05.003

Lee JH, 2011, J GINSENG RES, V35, P375, DOI 10.5142/jgr.2011.35.3.375

Li C, 2019, J EXP CLIN CANC RES, V38, DOI 10.1186/s13046-019-1069-3

Li HL, 2018, BMC CANCER, V18, DOI 10.1186/s12885-018-4299-4

Li JL, 2019, EXP MOL PATHOL, V106, P131, DOI 10.1016/j.yexmp.2019.01.003

Li L, 2014, ONCOL REP, V32, P1779, DOI 10.3892/or.2014.3422

Li Y, 2018, BIOCHEM BIOPH RES CO, V497, P473, DOI 10.1016/j.bbrc.2018.01.192

Liu B, 2010, ACTA PHARMACOL SIN, V31, P1154, DOI 10.1038/aps.2010.118

Liu FB, 2017, NAT CELL BIOL, V19, P1358, DOI 10.1038/ncb3630

Liu J, 2015, BBA-REV CANCER, V1855, P50, DOI 10.1016/j.bbcan.2014.11.005

Liu T, 2017, TUMOR BIOL, V39, DOI 10.1177/1010428317692225

Liu Y, 2020, NUTRIENTS, DOI [10.3390/nu120102467399924, DOI 10.3390/NU120102467399924]

Liu YN, 2018, FOOD FUNCT, V9, DOI 10.1039/c8fo01122b

Liu ZH, 2015, CHEM-BIOL INTERACT, V242, P227, DOI 10.1016/j.cbi.2015.10.014

Lu GM, 2018, J EXP CLIN CANC RES, V37, DOI 10.1186/s13046-018-0945-6

Luo YW, 2017, ONCOL LETT, V13, P867, DOI 10.3892/ol.2016.5482

Malicka I, 2019, HEALTH CARE WOMEN IN, V40, P682, DOI 10.1080/07399332.2019.1608208

Mathiyalagan R, 2019, MOLECULES, V24, DOI 10.3390/molecules24234367

McGuire A, 2015, CANCER METAST REV, V34, P145, DOI 10.1007/s10555-015-9551-7

Mekala JR, 2018, GENE, V641, P248, DOI 10.1016/j.gene.2017.10.018

Morrow JK, 2015, FUTURE MED CHEM, V7, P2333, DOI 10.4155/fmc.15.148

Nakhjavani M, 2019, PHARMACEUTICALS-BASE, V12, DOI 10.3390/ph12030117

Nosrati H, 2018, BIOORG CHEM, V76, P501, DOI 10.1016/j.bioorg.2017.12.033

Omarini C, 2018, CANCER MANAG RES, V10, P91, DOI 10.2147/CMAR.S146658

Otto T, 2017, NAT REV CANCER, V17, P93, DOI 10.1038/nrc.2016.138

Peng B, 2019, PHARMACOL RES, V142, P1, DOI 10.1016/j.phrs.2019.02.003

Pokharel YR, 2010, NUTR CANCER, V62, P252, DOI 10.1080/01635580903407171

Qiu RN, 2019, BIOMED MICRODEVICES, V21, DOI 10.1007/s10544-019-0374-0

Reinbolt RE, 2015, SEMIN ONCOL NURS, V31, P146, DOI 10.1016/j.soncn.2015.02.002

Ren ZG, 2020, SMALL, V16, DOI 10.1002/smll.201905233

Schwartz GK, 2005, J CLIN ONCOL, V23, P9408, DOI 10.1200/JCO.2005.01.5594

Shi YJ, 2014, J NANOBIOTECHNOL, V12, DOI 10.1186/s12951-014-0043-7

Shi Y, 2019, BRAZ J MED BIOL RES, V52, DOI [10.1590/1414-431X20198657, 10.1590/1414-431x20198657]

Shin SA, 2019, ARCH PHARM RES, V42, P658, DOI 10.1007/s12272-019-01169-2

Tang YC, 2018, INT J ONCOL, V52, P127, DOI 10.3892/ijo.2017.4183

Taveira MD, 2017, J CANCER RES CLIN, V143, P1255, DOI 10.1007/s00432-017-2358-x

Toh TB, 2017, MOL CANCER, V16, DOI 10.1186/s12943-017-0596-9

Tong-Lin Wu Tony, 2018, Oncotarget, V9, P11109, DOI 10.18632/oncotarget.24326

Mai TT, 2012, CANCER LETT, V321, P144, DOI 10.1016/j.canlet.2012.01.045

Waks AG, 2019, JAMA-J AM MED ASSOC, V321, P288, DOI 10.1001/jama.2018.19323

Wang B, 2014, J NANOSCI NANOTECHNO, V14, P5688, DOI 10.1166/jnn.2014.8876

Wang PW, 2016, SCI REP-UK, V6, DOI 10.1038/srep33709

Wang RX, 2019, ONCOLOGIST, V24, P753, DOI 10.1634/theoncologist.2017-0602

Wang W, 2012, PLOS ONE, V7, DOI 10.1371/journal.pone.0041586

Wen Xu, 2015, Asian Pac J Cancer Prev, V16, P1105

Xing Z, 2018, CANCER RES, V78, P4524, DOI 10.1158/0008-5472.CAN-17-0385

Yang JY, 2006, MOL CELL BIOL, V26, P7269, DOI 10.1128/MCB.00172-06

Yang JJ, 2016, J GINSENG RES, V40, P400, DOI 10.1016/j.jgr.2016.03.007

Yang J, 2014, HISTOCHEM CELL BIOL, V142, P401, DOI 10.1007/s00418-014-1222-1

Yang R, 2014, INT J NANOMED, V9, P1, DOI 10.2147/IJN.S67210

Yang X, 2017, DISCOV MED, V24, P75

Yu L, 2019, CANCER LETT, V448, P40, DOI 10.1016/j.canlet.2019.01.039

Yu X, 2015, CHEM PHARM BULL, V63, P361, DOI 10.1248/cpb.c15-00045

Yuan ZG, 2017, BIOMED PHARMACOTHER, V89, P227, DOI 10.1016/j.biopha.2017.02.038

Zare-Zardini H, 2018, SCI REP-UK, V8, DOI 10.1038/s41598-017-18938-y

Zhan JM, 2020, CARBOHYD POLYM, V230, DOI 10.1016/j.carbpol.2019.115576

Zhang EY, 2017, ONCOL REP, V38, P359, DOI 10.3892/or.2017.5652

Zhang JW, 2012, DRUG METAB DISPOS, V40, P1900, DOI 10.1124/dmd.112.045187

Zhang KQ, 2016, PHARM BIOL, V54, P561, DOI 10.3109/13880209.2015.1101142

Zhang Y, 2016, ASIAN PAC J TROP MED, V9, P178, DOI 10.1016/j.apjtm.2016.01.010

Zhao HG, 2018, ARCH PHARM RES, V41, P986, DOI 10.1007/s12272-018-1049-8

Zhao LQ, 2019, ONCOL REP, V41, P3209, DOI 10.3892/or.2019.7115

Zhu Y, 2019, ARCH BIOCHEM BIOPHYS, V662, P93, DOI 10.1016/j.abb.2018.11.023

Zou JF, 2020, J BIOCHEM MOL TOXIC, V34, DOI 10.1002/jbt.22480

NR 112

TC 23

Z9 23

U1 4

U2 23

PU PHARMACEUTICAL SOC KOREA

PI SEOUL

PA 1489-3 SUHCHO-DONG, SUHCHO-KU, SEOUL 137-071, SOUTH KOREA

SN 0253-6269

EI 1976-3786

J9 ARCH PHARM RES

JI Arch. Pharm. Res.

PD AUG

PY 2020

VL 43

IS 8

BP 773

EP 787

DI 10.1007/s12272-020-01265-8

EA AUG 2020

PG 15

WC Chemistry, Medicinal; Pharmacology & Pharmacy

WE Science Citation Index Expanded (SCI-EXPANDED)

SC Pharmacology & Pharmacy

GA NF7BA

UT WOS:000562358300001

PM 32839835

DA 2023-04-05

ER

PT J

AU Kim, J

Yoo, JM

Kim, JS

Kim, SG

Park, JE

Seok, YM

Son, JH

Kim, HJ

AF Kim, Jungeun

Yoo, Jae-Myung

Kim, Jin Soo

Kim, Sun-Gun

Park, Jong Eel

Seok, Young Mi

Son, Jun-Ho

Kim, Hyo Jung

TI Anticancer Effect of Mountain Ginseng on Human Breast Cancer: Comparison

with Farm-Cultivated Ginseng

SO EVIDENCE-BASED COMPLEMENTARY AND ALTERNATIVE MEDICINE

LA English

DT Article

ID PANAX-GINSENG; APOPTOSIS

AB Mountain ginseng has been used generally as a pharmacopuncture for cancer therapy in clinical practice in Northeast Asia. Nonetheless, there have been few scientific reports for the anticancer action of mountain ginseng. In this study, we investigated whether mountain ginseng extract (MGE) could inhibit the growth of breast cancer inin vitroandin vivomodels. MGE showed stronger cytotoxicity than farm-cultivated ginseng extract (FGE) through promoting ROS generation. Also MGE dose-dependently brought about mitochondrial dysfunction in MCF-7 cells. In addition, MGE induced apoptosis through enhancing the activities of caspase-3/7 by regulation of expression of Bcl-2, Bax, cytochrome c, and cleaved caspase-3 in the MCF-7 cells. Consistent with thein vitroresults, MGE significantly reduced tumor weights compared with FGE in mice transplanted with MCF-7 cells, and it regulated the expression of apoptosis-related proteins, such as Bcl-2, Bax, cytochrome c, cleaved caspase-3, and cleaved PARP, in the tumor tissues. Additionally, MGE included higher total ginsenoside contents than FGE. In conclusion, MGE, which is richer in ginsenosides, exerts a stronger anticancer action than FGE in breast cancer. The anticancer action of MGE may be closely correlated with caspase-mediated apoptosis through upregulating ROS generation. Therefore, these findings may be helpful for a clinical understanding of the anticancer mechanism of MGE for breast cancer patients.

C1 [Kim, Jungeun; Yoo, Jae-Myung; Kim, Jin Soo; Kim, Sun-Gun; Park, Jong Eel; Seok, Young Mi; Son, Jun-Ho; Kim, Hyo Jung] Natl Inst Korean Med Dev, Korean Med R&D Team 1, Gyongsan 38540, South Korea.

[Park, Jong Eel] Unimed Pharmaceut Inc, Seoul 05567, South Korea.

RP Kim, HJ (通讯作者)，Natl Inst Korean Med Dev, Korean Med R&D Team 1, Gyongsan 38540, South Korea.

EM astely88@nikom.or.kr; jmyoo@cnu.ac.kr; 1211pepero@nikom.or.kr;

sungun@nikom.or.kr; lycos2239@nate.com; imaria@nikom.or.kr;

bio115@nikom.or.kr; indersee31@nikom.or.kr

RI Yoo, Jae-Myung/M-9151-2017

OI Yoo, Jae-Myung/0000-0002-3924-6919

FU Standardization Project of Korean Medicine Acupuncture - Korean Ministry

of Health and Welfare [3243-302]

FX This work was supported by the Standardization Project of Korean

Medicine Acupuncture funded by the Korean Ministry of Health and Welfare

(Grant no. 3243-302).

CR Baeg IH, 2013, J GINSENG RES, V37, P1, DOI 10.5142/jgr.2013.37.1

Choi KT, 2008, ACTA PHARMACOL SIN, V29, P1109, DOI 10.1111/j.1745-7254.2008.00869.x

Elrod HA, 2008, PPAR RES, V2008, DOI 10.1155/2008/704165

Emamaullee JA, 2006, DIABETES, V55, P1907, DOI 10.2337/db05-1254

Galluzzi L, 2008, PLOS PATHOG, V4, DOI 10.1371/journal.ppat.1000018

Jung KW, 2019, CANCER RES TREAT, V51, P417, DOI 10.4143/crt.2019.138

Kang HM, 2016, SPRINGERPLUS, V5, DOI 10.1186/s40064-016-2031-x

Kim KH, 2018, J GINSENG RES, V42, P239, DOI 10.1016/j.jgr.2017.03.011

Kim S, 2018, PLOS ONE, V13, DOI 10.1371/journal.pone.0207737

Lee JY, 2019, FOOD SCI BIOTECHNOL, V28, P1845, DOI 10.1007/s10068-019-00632-6

Lee KH, 2016, J PHARMACOPUNCT, V19, P101, DOI 10.3831/KPI.2016.19.010

Majeed F, 2018, BIOMED PHARMACOTHER, V100, P52, DOI 10.1016/j.biopha.2018.01.155

Mancini M, 1997, J CELL BIOL, V138, P449, DOI 10.1083/jcb.138.2.449

Moloney JN, 2018, SEMIN CELL DEV BIOL, V80, P50, DOI 10.1016/j.semcdb.2017.05.023

Naik A, 2019, FRONT IMMUNOL, V10, DOI 10.3389/fimmu.2019.01940

Park EH, 2017, J BREAST CANCER, V20, P1, DOI 10.4048/jbc.2017.20.1.1

Park ES, 2014, MOL CARCINOGEN, V53, pE23, DOI 10.1002/mc.21990

SOULE HD, 1973, J NATL CANCER I, V51, P1409, DOI 10.1093/jnci/51.5.1409

Suh H, 2010, FOR SCI TECHNOL, V6, P118, DOI 10.1080/21580103.2010.9671979

Xu XF, 2016, J GINSENG RES, V40, P344, DOI 10.1016/j.jgr.2015.11.001

Yoo JM, 2019, PHYTOMEDICINE, V55, P229, DOI 10.1016/j.phymed.2018.06.034

Yoo JM, 2019, AM J CHINESE MED, V47, P203, DOI [10.1142/s0192415x19500101, 10.1142/S0192415X19500101]

Zou ZZ, 2017, APOPTOSIS, V22, P1321, DOI 10.1007/s10495-017-1424-9

NR 23

TC 10

Z9 11

U1 1

U2 4

PU HINDAWI LTD

PI LONDON

PA ADAM HOUSE, 3RD FLR, 1 FITZROY SQ, LONDON, W1T 5HF, ENGLAND

SN 1741-427X

EI 1741-4288

J9 EVID-BASED COMPL ALT

JI Evid.-based Complement Altern. Med.

PD JUL 26

PY 2020

VL 2020

AR 2584783

DI 10.1155/2020/2584783

PG 10

WC Integrative & Complementary Medicine

WE Science Citation Index Expanded (SCI-EXPANDED)

SC Integrative & Complementary Medicine

GA ND3OA

UT WOS:000561811900004

PM 32774407

OA Green Published, gold

DA 2023-04-05

ER

PT J

AU Zare-Zardini, H

Alemi, A

Taheri-Kafrani, A

Hosseini, SA

Soltaninejad, H

Hamidieh, AA

Karamallah, MH

Farrokhifar, M

Farrokhifar, M

AF Zare-Zardini, Hadi

Alemi, Ashraf

Taheri-Kafrani, Asghar

Hosseini, Seyed Ahmad

Soltaninejad, Hossein

Hamidieh, Amir Ali

Karamallah, Mojtaba Haghi

Farrokhifar, Majid

Farrokhifar, Mohammad

TI Assessment of a New Ginsenoside Rh2 Nanoniosomal Formulation for

Enhanced Antitumor Efficacy on Prostate Cancer: An in vitro Study

SO DRUG DESIGN DEVELOPMENT AND THERAPY

LA English

DT Article

DE nanoniosomal; Ginsenoside Rh2; chemotherapy; PC3 prostate cancer cell

line

ID DELIVERY-SYSTEM; NIOSOMES; PACLITAXEL; CURCUMIN; NANOPARTICLES; DRUGS;

ACID

AB Introduction: Ginsenoside Rh2, purified from the Panax ginseng root, has been demon-strated to possess anticancer properties against various cancerous cells including colorectal, breast, skin, ovarian, prostate, and liver cancerous cells. However, the poor bioavailability, low stability on gastrointestinal systems, and fast plasma elimination limit further clinical applications of Ginsenoside Rh2 for cancer treatments. In this study, a novel formulation of niosomal Ginsenoside Rh2 was prepared using the thin film hydration technique.

Methods: The niosomal formulation contained Span 60 and cholesterol, and cationic lipid DOTAP was evaluated by determining particle size distribution, encapsulation efficiency, the polydispersity index (PDI), and surface morphology. The cytotoxic effects of free Ginsenoside Rh2 and Ginsenoside Rh2-loaded niosomes were determined using the MTT method in the PC3 prostate cancer cell line. For the investigation of the in vitro cellular uptake of Ginsenoside Rh2-loaded niosome, two formulations were prepared: the Ginsenoside Rh2-loaded niosomal formula containing 5% DOTAP and the Ginsenoside Rh2-loaded niosomal formula without DOTAP.

Results: The mean size, DPI, zeta potential, and encapsulation efficiency of the Ginsenoside Rh2-loaded nanoniosomal formulation containing DOTAP were 93.5 +/- 2.1 nm, 0.203 +/- 0.01, +4.65 +/- 0.65, and 98.32% +/- 2.4, respectively. The niosomal vesicles were found to be round and have a smooth surface. The release profile of Ginsenoside Rh2 from niosome was biphasic. Furthermore, a two-fold reduction in the Ginsenoside Rh2 concentration was measured when Ginsenoside Rh2 was administered in a nanoniosomal form compared to free Ginsenoside Rh2 solutions in the PC3 prostate cancer cell line. After storage for 90 days, the encapsulation efficiency, vesicle size, PDI, and zeta potential of the optimized formulation did not significantly change compared to the freshly prepared samples. The cellular uptake experiments of the niosomal formulation demonstrated that by adding DOTAP to the niosomal formulation, the cellular uptake was enhanced.

Discussion: The enhanced cellular uptake and cytotoxic activity of the Ginsenoside Rh2 nanoniosomal formulation on the PC3 cell make it an attractive candidate for application as a nano-sized delivery vehicle to transfer Ginsenoside Rh2 to cancer cells.

C1 [Zare-Zardini, Hadi] Shahid Sadoughi Univ Med Sci, Hematol & Oncol Res Ctr, Yazd, Iran.

[Zare-Zardini, Hadi] Farhangian Univ, Dept Sci, Esfahan, Iran.

[Zare-Zardini, Hadi] Shahid Sadoughi Univ Med Sci, Med Nanotechnol & Tissue Engn Res Ctr, Yazd Reprod Sci Inst, Yazd, Iran.

[Alemi, Ashraf] Abadan Fac Med Sci, Abadan, Iran.

[Taheri-Kafrani, Asghar] Univ Isfahan, Fac Adv Sci & Technol, Dept Biotechnol, Esfahan, Iran.

[Hosseini, Seyed Ahmad] Ahvaz Jundishapur Univ Med Sci, Nutr & Metab Dis Res Ctr, Ahvaz, Iran.

[Soltaninejad, Hossein] Univ Tehran Med Sci, Tissue Bank & Res Ctr, Tehran, Iran.

[Soltaninejad, Hossein] Tarbiat Modares Univ, Fac Biol Sci, Dept Nanobiotechnol, Tehran, Iran.

[Hamidieh, Amir Ali] Univ Tehran Med Sci, Stem Cell & Regenerat Med Inst, Tehran, Iran.

[Karamallah, Mojtaba Haghi] Shoushtar Fac Med Sci, Shoushtar, Iran.

[Farrokhifar, Majid] Sabzevar Univ Med Sci, Dept Pediat, Sabzevar, Iran.

[Farrokhifar, Mohammad] Kar Higher Educ Inst Rafsanjan, Rafsanjan, Iran.

C3 University of Isfahan; Ahvaz Jundishapur University of Medical Sciences

(AJUMS); Tehran University of Medical Sciences; Tarbiat Modares

University; Tehran University of Medical Sciences

RP Alemi, A (通讯作者)，Abadan Fac Med Sci, Abadan, Iran.

EM alemi.ashraf@gmail.com

RI Taheri-Kafrani, Asghar/AAL-4297-2021; alemi, ashraf/D-6533-2017;

Hosseini, Ahmad/HCH-2017-2022; Zare-Zardini, Hadi/ABR-4283-2022

OI alemi, ashraf/0000-0003-1780-7540; Zare-Zardini,

Hadi/0000-0002-1501-2560; Hosseini, Seyed Ahmad/0000-0002-9075-4126

CR Abu Lila AS, 2010, PHARM RES-DORDR, V27, P1171, DOI 10.1007/s11095-010-0110-1

Alemi A, 2018, IRAN J PEDIATR HEMAT, V8, P153

Alemi A, 2018, J NANOBIOTECHNOL, V16, DOI 10.1186/s12951-018-0351-4

Azandeh SS, 2017, IRAN J PHARM RES, V16, P868

Baek JS, 2017, ONCOTARGET, V8, P30369, DOI 10.18632/oncotarget.16153

Chen DQ, 2014, ARTIF CELL NANOMED B, V42, P205, DOI 10.3109/21691401.2013.794358

Chen SH, 2014, EVID-BASED COMPL ALT, V2014, DOI 10.1155/2014/168940

Chen Y, 2016, ONCOL REP, V36, P137, DOI 10.3892/or.2016.4774

Ertekin ZC, 2015, CURR DRUG DELIV, V12, P192, DOI 10.2174/1567201811666140723115852

Gonzalgo ML, 2003, J UROLOGY, V170, P2444, DOI 10.1097/01.ju.0000085381.20139.b6

Han M, 2009, BIOL PHARM BULL, V32, P1069, DOI 10.1248/bpb.32.1069

Hong C, 2019, THERANOSTICS, V9, P4437, DOI 10.7150/thno.34953

Hong MH, 2009, J CONTROL RELEASE, V133, P96, DOI 10.1016/j.jconrel.2008.09.005

Jin X, 2020, NANOMEDICINE-UK, V15, P41, DOI 10.2217/nnm-2018-0479

Karim KM, 2010, J ADV PHARM TECHNOL, V1, P374, DOI 10.4103/0110-5558.76435

Kim AD, 2010, ENVIRON TOXICOL PHAR, V30, P134, DOI 10.1016/j.etap.2010.04.008

Kim H, 2018, J GINSENG RES, V42, P361

Lee H, 2018, J GINSENG RES, V42, P455, DOI 10.1016/j.jgr.2017.05.003

Leung Kar Wah, 2010, Chin Med, V5, P20, DOI 10.1186/1749-8546-5-20

Li BH, 2011, CANCER LETT, V301, P185, DOI 10.1016/j.canlet.2010.11.015

Li LA, 2011, DRUG METAB DISPOS, V39, P472, DOI 10.1124/dmd.110.036723

Lin YL, 2012, NANOMED-NANOTECHNOL, V8, P318, DOI 10.1016/j.nano.2011.06.011

Lv SX, 2014, BIOMATERIALS, V35, P6118, DOI 10.1016/j.biomaterials.2014.04.034

Nag SA, 2012, FRONT PHARMACOL, V3, DOI 10.3389/fphar.2012.00025

Paek IP, 2006, BIOPHARM DRUG DISPOS, V27, P39, DOI 10.1002/bdd.481

Rahman MA, 2011, CURR DRUG DELIV, V8, P330

Rajera R, 2011, BIOL PHARM BULL, V34, P945, DOI 10.1248/bpb.34.945

Richie JP, 1999, UROLOGY, V54, P15, DOI 10.1016/S0090-4295(99)00449-5

Sezgin-Bayindir Z, 2015, AAPS PHARMSCITECH, V16, P108, DOI 10.1208/s12249-014-0213-9

Shaker DS, 2015, INT J PHARMACEUT, V493, P285, DOI 10.1016/j.ijpharm.2015.07.041

Sharma V, 2015, MAT SCI ENG C-MATER, V56, P393, DOI 10.1016/j.msec.2015.06.049

Shilakari Asthana G, 2016, SCIENTIFICA, V2016, DOI [10.1155/2016/649295326., DOI 10.1155/2016/649295326]

Shrestha H, 2014, J PHARMACEUTICS, V2014, DOI 10.1155/2014/801820

Song BK, 2017, J MICROBIOL BIOTECHN, V27, P1233, DOI 10.4014/jmb.1701.01077

Sun MY, 2017, INT J MOL MED, V39, P507, DOI 10.3892/ijmm.2017.2857

Uchegbu IF, 1998, INT J PHARM, V172, P33, DOI 10.1016/S0378-5173(98)00169-0

Wang JN, 2016, J MATER CHEM B, V4, P2954, DOI 10.1039/c5tb02450a

Wang M, 2017, ONCOL LETT, V13, P681, DOI 10.3892/ol.2016.5490

Xu LQ, 2015, J NANOPART RES, V17, DOI 10.1007/s11051-015-3214-z

Xu YQ, 2016, J NANOMATER, V2016, DOI 10.1155/2016/6365295

Zare-Zardini H, 2018, SCI REP-UK, V8, DOI 10.1038/s41598-017-18938-y

Zhuang JJ, 2018, NUTRIENTS, V10, DOI 10.3390/nu10030328

NR 42

TC 10

Z9 11

U1 6

U2 31

PU DOVE MEDICAL PRESS LTD

PI ALBANY

PA PO BOX 300-008, ALBANY, AUCKLAND 0752, NEW ZEALAND

SN 1177-8881

J9 DRUG DES DEV THER

JI Drug Des. Dev. Ther.

PY 2020

VL 14

BP 3315

EP 3324

DI 10.2147/DDDT.S261027

PG 10

WC Chemistry, Medicinal; Pharmacology & Pharmacy

WE Science Citation Index Expanded (SCI-EXPANDED)

SC Pharmacology & Pharmacy

GA NC3FP

UT WOS:000561100600001

PM 32884236

OA Green Published, gold

DA 2023-04-05

ER

PT J

AU My, PL

My, HTK

Phuong, NTX

Dat, TD

Thanh, VH

Nam, HM

Phong, MT

Hieu, NH

AF My, Phan Le Thao

My, Huynh Thi Kieu

Phuong, Nguyen Tran Xuan

Dat, Tran Do

Thanh, Vuong Hoai

Nam, Hoang Minh

Thanh Phong, Mai

Hieu, Nguyen Huu

TI Optimization of enzyme-assisted extraction of ginsenoside Rb1 from

VietnamesePanax notoginseng(BURK.) FH Chen roots and anticancer activity

examination of the extract

SO SEPARATION SCIENCE AND TECHNOLOGY

LA English

DT Article

DE Panax notoginseng(Burk; ) F; H; Chen; response surface methodology;

central composite design; ginsenoside Rb1; anticancer

ID PANAX-NOTOGINSENG; ANTIOXIDANT ACTIVITIES; POLYSACCHARIDES; TEMPERATURE;

FLAVONOIDS; CAPACITY; SAPONINS

AB In this study, the conditions of ginsenoside Rb1 extraction fromPanax notoginseng(Burk.) F.H. Chen (P. notoginseng) roots by enzyme-assisted method were investigated by using the central composite design (CCD). This design was applied to survey the effects of pH, temperature, and time on Rb1 content. The concentration of Rb1 was determined based on high-performance liquid chromatography (HPLC). The investigation of anticancer activities ofP. notoginsengextract on breast carcinoma (MCF-7) cell lines and human lung carcinoma (Lu) showed that the extract had remarkable cytotoxic activity against these cancer cell lines.

C1 [My, Phan Le Thao; Phuong, Nguyen Tran Xuan; Dat, Tran Do; Thanh, Vuong Hoai; Hieu, Nguyen Huu] VNU HCMC Key Lab Chem Engn & Petr Proc CEPP Lab, Ho Chi Minh City, Vietnam.

[My, Huynh Thi Kieu; Nam, Hoang Minh; Thanh Phong, Mai; Hieu, Nguyen Huu] Ho Chi Minh City Univ Technol, Fac Chem Engn, Ho Chi Minh City, Vietnam.

[Phuong, Nguyen Tran Xuan] Hong Bang Int Univ, Dept Chem, Ho Chi Minh City, Vietnam.

[Nam, Hoang Minh; Thanh Phong, Mai; Hieu, Nguyen Huu] Vietnam Natl Univ Ho Chi Minh City, Ho Chi Minh City, Vietnam.

C3 Ho Chi Minh City University of Technology (HCMCUT); Vietnam National

University Hochiminh City; Hong Bang International University; Vietnam

National University Hochiminh City

RP Hieu, NH (通讯作者)，VNU HCMC Key Lab Chem Engn & Petr Proc CEPP Lab, Ho Chi Minh City, Vietnam.

EM nhhieubk@hcmut.edu.vn

RI Vuong, Hoai-Thanh/AAF-7510-2021

OI Vuong, Hoai-Thanh/0000-0001-9669-7209; Thanh Phong,

Mai/0000-0003-4532-3447

CR Chen S, 2011, ENZYME MICROB TECH, V48, P100, DOI 10.1016/j.enzmictec.2010.09.017

Chien YS, 2016, SEP SCI TECHNOL, V51, P954, DOI 10.1080/01496395.2016.1140202

Dai CY, 2018, MOLECULES, V23, DOI 10.3390/molecules23092219

Daniel RM, 2008, EXTREMOPHILES, V12, P51, DOI 10.1007/s00792-007-0089-7

Dinkova R, 2014, FOOD RES INT, V65, P35, DOI 10.1016/j.foodres.2014.05.066

Gupta Neelima, 2012, Trends in Parasitology Research, V1, P1

Kim SJ, 2007, SEP PURIF TECHNOL, V56, P401, DOI 10.1016/j.seppur.2007.06.014

Lau AJ, 2003, J CHROMATOGR A, V1011, P77, DOI 10.1016/S0021-9673(03)01135-X

Liu L, 2020, J ETHNOPHARMACOL, V263, DOI 10.1016/j.jep.2020.112792

Michiels JA, 2012, FOOD CHEM, V130, P986, DOI 10.1016/j.foodchem.2011.07.117

Mohanan P, 2018, J GINSENG RES, V42, P123, DOI 10.1016/j.jgr.2017.01.008

Nadar SS, 2018, FOOD RES INT, V108, P309, DOI 10.1016/j.foodres.2018.03.006

Nag SA, 2012, FRONT PHARMACOL, V3, DOI 10.3389/fphar.2012.00025

Phuong NTX, 2018, AIP CONF PROC, V1954, DOI 10.1063/1.5033403

Park TY, 2008, BIOL PHARM BULL, V31, P1802, DOI 10.1248/bpb.31.1802

Phuong N. T. X., 2018, VIETNAM J CHEM, V56

Puri M, 2012, TRENDS BIOTECHNOL, V30, P37, DOI 10.1016/j.tibtech.2011.06.014

Ratan Z. A., 2020, JGR

Renjie L, 2008, CARBOHYD POLYM, V74, P858, DOI 10.1016/j.carbpol.2008.05.005

Saw CLL, 2012, CHEM RES TOXICOL, V25, P1574, DOI 10.1021/tx2005025

Song YR, 2018, INT J BIOL MACROMOL, V116, P1089, DOI 10.1016/j.ijbiomac.2018.05.132

Sowbhagya HB, 2010, CRIT REV FOOD SCI, V50, P146, DOI 10.1080/10408390802248775

Kim TD, 2016, ASIAN PAC J TROP BIO, V6, P795, DOI 10.1016/j.apjtb.2016.04.013

Tijskens LMM, 2001, BIOTECHNOL BIOENG, V72, P323, DOI 10.1002/1097-0290(20010205)72:3<323::AID-BIT9>3.0.CO;2-I

Tomaz I, 2016, SEP SCI TECHNOL, V51, P255, DOI 10.1080/01496395.2015.1085881

Uzayisenga R, 2014, PHYTOTHER RES, V28, P510, DOI 10.1002/ptr.5026

Vieira JM, 2019, CARBOHYD POLYM, V213, P217, DOI 10.1016/j.carbpol.2019.02.078

Wang CZ, 2009, PHYTOTHER RES, V23, P6, DOI 10.1002/ptr.2383

Wang SP, 2013, INT J BIOL MACROMOL, V62, P387, DOI 10.1016/j.ijbiomac.2013.09.029

Yin XL, 2011, CARBOHYD POLYM, V86, P1358, DOI 10.1016/j.carbpol.2011.06.053

Zhang WC, 2017, SEP SCI TECHNOL, V52, P1350, DOI 10.1080/01496395.2017.1287736

NR 31

TC 3

Z9 3

U1 3

U2 32

PU TAYLOR & FRANCIS INC

PI PHILADELPHIA

PA 530 WALNUT STREET, STE 850, PHILADELPHIA, PA 19106 USA

SN 0149-6395

EI 1520-5754

J9 SEP SCI TECHNOL

JI Sep. Sci. Technol.

PD JUL 3

PY 2021

VL 56

IS 10

BP 1687

EP 1698

DI 10.1080/01496395.2020.1795676

EA AUG 2020

PG 12

WC Chemistry, Multidisciplinary; Engineering, Chemical

WE Science Citation Index Expanded (SCI-EXPANDED)

SC Chemistry; Engineering

GA SL8GD

UT WOS:000555165600001

DA 2023-04-05

ER

PT J

AU Hong, C

Liang, JM

Xia, JX

Zhu, Y

Guo, YZ

Wang, AN

Lu, CY

Ren, HW

Chen, C

Li, SY

Wang, D

Zhan, HX

Wang, JX

AF Hong, Chao

Liang, Jianming

Xia, Jiaxuan

Zhu, Ying

Guo, Yizhen

Wang, Anni

Lu, Chunyi

Ren, Hongwei

Chen, Chen

Li, Shiyi

Wang, Dan

Zhan, Huaxing

Wang, Jianxin

TI One Stone Four Birds: A Novel Liposomal Delivery System

Multi-functionalized with Ginsenoside Rh2 for Tumor Targeting Therapy

SO NANO-MICRO LETTERS

LA English

DT Article

DE Ginsenoside Rh2; Liposomes; Cholesterol; Multifunction; Tumor targeting

ID PROTEIN CORONA; NANOPARTICLES; DOXORUBICIN; CELLS; NANOCARRIER;

STRATEGIES; MECHANISM; CARCINOMA

AB Liposomes hold great potential in anti-cancer drug delivery and the targeting treatment of tumors. However, the clinical therapeutic efficacy of liposomes is still limited by the complexity of tumor microenvironment (TME) and the insufficient accumulation in tumor sites. Meanwhile, the application of cholesterol and polyethylene glycol (PEG), which are usually used to prolong the blood circulation and stabilize the structure of liposomes respectively, has been questioned due to various disadvantages. Herein, we developed a ginsenoside Rh2-based multifunctional liposome system (Rh2-lipo) to effectively address these challenges once for all. Different with the conventional 'wooden' liposomes, Rh2-lipo is a much more brilliant carrier with multiple functions. In Rh2-lipo, both cholesterol and PEG were substituted by Rh2, which works as membrane stabilizer, long-circulating stealther, active targeting ligand, and chemotherapy adjuvant at the same time. Firstly, Rh2 could keep the stability of liposomes and avoid the shortcomings caused by cholesterol. Secondly, Rh2-lipo showed a specifically prolonged circulation behavior in the blood. Thirdly, the accumulation of the liposomes in the tumor was significantly enhanced by the interaction of glucose transporter of tumor cells with Rh2. Fourth, Rh2-lipo could remodel the structure and reverse the immunosuppressive environment in TME. When tested in a 4T1 breast carcinoma xenograft model, the paclitaxel-loaded Rh2-lipo realized high efficient tumor growth suppression. Therefore, Rh2-lipo not only innovatively challenges the position of cholesterol as a liposome component, but also provides another innovative potential system with multiple functions for anti-cancer drug delivery.

C1 [Hong, Chao; Liang, Jianming; Xia, Jiaxuan; Guo, Yizhen; Wang, Anni; Ren, Hongwei; Chen, Chen; Li, Shiyi; Wang, Dan; Wang, Jianxin] Fudan Univ, Sch Pharm, Dept Pharmaceut, Shanghai 201203, Peoples R China.

[Hong, Chao; Liang, Jianming; Xia, Jiaxuan; Guo, Yizhen; Wang, Anni; Ren, Hongwei; Chen, Chen; Li, Shiyi; Wang, Dan; Wang, Jianxin] Minist Educ, Key Lab Smart Drug Delivery, Shanghai 201203, Peoples R China.

[Liang, Jianming] Guangzhou Univ Chinese Med, Inst Trop Med, Guangzhou 510006, Peoples R China.

[Zhu, Ying] Guangzhou Univ Chinese Med, Inst Clin Pharmacol, Guangzhou 510006, Peoples R China.

[Lu, Chunyi] Shanghai Jiao Tong Univ, Sch Pharm, Shanghai 200240, Peoples R China.

[Wang, Dan; Zhan, Huaxing] Shanghai Ginposome Pharmatech Co Ltd, Shanghai 201600, Peoples R China.

[Wang, Jianxin] Fudan Univ, Inst Integrated Chinese & Western Med, Shanghai 200040, Peoples R China.

C3 Fudan University; Guangzhou University of Chinese Medicine; Guangzhou

University of Chinese Medicine; Shanghai Jiao Tong University; Fudan

University

RP Wang, JX (通讯作者)，Fudan Univ, Sch Pharm, Dept Pharmaceut, Shanghai 201203, Peoples R China.; Wang, JX (通讯作者)，Minist Educ, Key Lab Smart Drug Delivery, Shanghai 201203, Peoples R China.; Wang, JX (通讯作者)，Fudan Univ, Inst Integrated Chinese & Western Med, Shanghai 200040, Peoples R China.

EM jxwang@fudan.edu.cn

FU National Natural Science Foundation of China [81773911, 81690263,

81573616]; Development Project of Shanghai Peak Disciplines-Integrated

Medicine [20150407]

FX This work was supported by National Natural Science Foundation of China

(Nos. 81773911, 81690263 and 81573616) and the Development Project of

Shanghai Peak Disciplines-Integrated Medicine (No. 20150407).

CR Akoev VR, 1996, BIOL MEMBRANY, V13, P605

Alberts DS, 1997, DRUGS, V54, P30, DOI 10.2165/00003495-199700544-00007

Bertrand N, 2017, NAT COMMUN, V8, DOI 10.1038/s41467-017-00600-w

Bigdeli A, 2016, ACS NANO, V10, P3723, DOI 10.1021/acsnano.6b00261

Blenke EO, 2013, EXPERT OPIN DRUG DEL, V10, P1399, DOI 10.1517/17425247.2013.805742

Chang TC, 2007, J AGR FOOD CHEM, V55, P1993, DOI 10.1021/jf062714k

Chen SH, 2014, EVID-BASED COMPL ALT, V2014, DOI 10.1155/2014/168940

Chen YC, 2016, NAT COMMUN, V7, DOI 10.1038/ncomms13443

Chen Y, 2017, INT J CLIN EXP MED, V10, P14904

deMarie S, 1996, LEUKEMIA, V10, pS93

FUKUDA K, 1985, BIOCHIM BIOPHYS ACTA, V820, P199, DOI 10.1016/0005-2736(85)90113-0

Gallova J, 2011, J MEMBRANE BIOL, V243, P1, DOI 10.1007/s00232-011-9387-1

Han S, 2016, J ETHNOPHARMACOL, V194, P83, DOI 10.1016/j.jep.2016.08.039

Hong SS, 2016, INT J NANOMED, V11, P4465, DOI 10.2147/IJN.S113723

Hu CMJ, 2011, P NATL ACAD SCI USA, V108, P10980, DOI 10.1073/pnas.1106634108

Inbaraj JJ, 2006, J AM CHEM SOC, V128, P9549, DOI 10.1021/ja0622204

Jain S, 2012, BIOMATERIALS, V33, P6758, DOI 10.1016/j.biomaterials.2012.05.026

Jia WWG, 2004, CAN J PHYSIOL PHARM, V82, P431, DOI [10.1139/y04-049, 10.1139/Y04-049]

Johnson L, 2010, DISCOV MED, V9, P374

LEVINE SJ, 1991, ANN INTERN MED, V114, P664, DOI 10.7326/0003-4819-114-8-664

Li SD, 2010, J CONTROL RELEASE, V145, P178, DOI 10.1016/j.jconrel.2010.03.016

Barran-Berdon AL, 2013, LANGMUIR, V29, P6485, DOI 10.1021/la401192x

Lin YJ, 2017, J GINSENG RES, V41, P330, DOI 10.1016/j.jgr.2016.06.005

Lv Q, 2016, PLANTA MED, V82, P705, DOI 10.1055/s-0042-101764

MacDermaid CM, 2015, J CHEM PHYS, V143, DOI 10.1063/1.4937153

Mahmoudi M, 2013, ACS NANO, V7, P6555, DOI 10.1021/nn305337c

Medina RA, 2002, BIOL RES, V35, P9, DOI 10.4067/S0716-97602002000100004

Miao L, 2015, J CONTROL RELEASE, V219, P192, DOI 10.1016/j.jconrel.2015.08.017

Moghimi SM, 2006, J LIPOSOME RES, V16, P167, DOI 10.1080/08982100600848801

O'Neill FH, 2005, AM J CARDIOL, V96, p29D, DOI 10.1016/j.amjcard.2005.03.017

Papi M, 2017, NANOSCALE, V9, P10327, DOI 10.1039/c7nr03042h

Qi LW, 2011, PHYTOCHEMISTRY, V72, P689, DOI 10.1016/j.phytochem.2011.02.012

Quail DF, 2013, NAT MED, V19, P1423, DOI 10.1038/nm.3394

Riaz MK, 2018, INT J MOL SCI, V19, DOI 10.3390/ijms19010195

RINGDEN O, 1994, LANCET, V344, P1156, DOI 10.1016/S0140-6736(94)90663-7

Rosenblum D, 2018, NAT COMMUN, V9, DOI 10.1038/s41467-018-03705-y

Schottler S, 2016, NAT NANOTECHNOL, V11, P372, DOI [10.1038/NNANO.2015.330, 10.1038/nnano.2015.330]

Sercombe L, 2015, FRONT PHARMACOL, V6, DOI 10.3389/fphar.2015.00286

Skubitz KM, 1998, ANTI-CANCER DRUG, V9, P45, DOI 10.1097/00001813-199801000-00005

Su YQ, 2018, ASIAN J PHARM SCI, V13, P44, DOI 10.1016/j.ajps.2017.07.003

Subczynski WK, 2009, FREE RADICAL BIO MED, V46, P707, DOI 10.1016/j.freeradbiomed.2008.11.024

Sun XL, 2017, THERANOSTICS, V7, P319, DOI 10.7150/thno.18078

Suntres ZE, 2011, J TOXICOL-US, V2011, DOI 10.1155/2011/152474

Szebeni J, 2000, AM J PHYSIOL-HEART C, V279, pH1319, DOI 10.1152/ajpheart.2000.279.3.H1319

Tenzer S, 2013, NAT NANOTECHNOL, V8, P772, DOI 10.1038/nnano.2013.181

UZIELY B, 1995, J CLIN ONCOL, V13, P1777, DOI 10.1200/JCO.1995.13.7.1777

VERBIK DJ, 1995, INT J ONCOL, V7, P205

Verhoef JJF, 2013, DRUG DELIV TRANSL RE, V3, P499, DOI 10.1007/s13346-013-0176-5

Wang YZ, 2018, FRONT PHARMACOL, V9, DOI 10.3389/fphar.2018.00398

Xia SQ, 2015, COLLOID SURFACE B, V128, P172, DOI 10.1016/j.colsurfb.2015.02.004

Yin T, 2016, COLLOID SURFACE B, V146, P902, DOI 10.1016/j.colsurfb.2016.07.040

Zhu CL, 2016, ARCH IRAN MED, V19, P23, DOI 0161901/AIM.005

NR 52

TC 25

Z9 27

U1 12

U2 99

PU SHANGHAI JIAO TONG UNIV PRESS

PI SHANGHAI

PA SHANGHAI JIAO TONG UNIV, 800 DONGCHUAN RD, SHANGHAI, 200240, PEOPLES R

CHINA

SN 2311-6706

EI 2150-5551

J9 NANO-MICRO LETT

JI Nano-Micro Lett.

PD JUN 16

PY 2020

VL 12

IS 1

AR 129

DI 10.1007/s40820-020-00472-8

PG 18

WC Nanoscience & Nanotechnology; Materials Science, Multidisciplinary;

Physics, Applied

WE Science Citation Index Expanded (SCI-EXPANDED)

SC Science & Technology - Other Topics; Materials Science; Physics

GA MB7TR

UT WOS:000542802300001

PM 34138128

OA gold, Green Published

DA 2023-04-05

ER

PT J

AU Wang, PP

Song, D

Wan, DH

Li, LY

Mei, WH

Li, XY

Han, L

Zhu, XF

Yang, L

Cai, Y

Zhang, RH

AF Wang, Panpan

Song, Dan

Wan, Danhong

Li, Lingyu

Mei, Wenhui

Li, Xiaoyun

Han, Li

Zhu, Xiaofeng

Yang, Li

Cai, Yu

Zhang, Ronghua

TI Ginsenoside panaxatriol reverses TNBC paclitaxel resistance by

inhibiting the IRAK1/NF-kappa B and ERK pathways

SO PEERJ

LA English

DT Article

DE TNBC; Paclitaxel resistance; IRAK1/NF-kappa B pathway; ERK pathway;

Ginsenoside panaxatriol

ID NF-KAPPA-B; BREAST-CANCER CELLS; STEM-CELLS; TARGET; CHEMORESISTANCE;

EXPRESSION; BLOCKING; ANTIBODY; THERAPY; FAMILY

AB Background: Paclitaxel (PTX) resistance is a major obstacle in the treatment of triple-negative breast cancer (TNBC). Previously, we have reported that interleukin-1 receptor-associated kinase 1 (IRAK1) and its downstream pathways are associated with PTX resistance in TNBC cells. In this study, we sought to investigate the combination treatment of ginsenoside panaxatriol (GPT), one of the main active components in Panax ginseng, with PTX on viability and apoptosis of TNBC PTX resistant cells, and explore the role of IRAK1 mediated signaling pathways in the therapeutic effects.

Methods: CellTiter-Glo and colony formation assays were used to assess cell viability. Flow cytometry was used to analyze subGI and apoptosis. Western blot was used to detect expressions of proteins involved in apoptosis and the IRAK1/NF-kappa B and ERK pathways. The mRNA expression of inflammatory cytokines, S100A7/8/9 and cancer stem cell (CSC)-related genes were examined by qPCR. Stem cells were identified by tumor sphere assay. Cell invasion ability was examined by transwell assay.

Results: We show that GPT inhibits MDA-MB-231 PTX resistant (MB231-PR) cell viability in a dose-dependent manner. When combined with PTX, GPT synergistically causes more cell death, induces subG1 accumulation and cell apoptosis. Besides, up-regulation of BAX/BCL-2 ratio, and down-regulation of MCL-1 are also observed. Moreover, this combination inhibits IRAK1, NF-kappa B and ERK1/2 activation, and leads to down-regulation of inflammatory cytokines (IL6, IL8, CXCL1, CCL2), S100A7/9 and CSC-related genes (OCT4, SOX2, NANOG, ALDH1, CD44) expression. In addition, the combination treatment suppresses MB231-PR cell invasion ability, and impairs tumor sphere growth both in MB231-PR and SUM159 PTX resistant (SUM159-PR) cells.

Conclusion: Our study demonstrates that GPT can resensitize TNBC PTX resistant cells to PTX by inhibiting the IRAK1/NF-kappa B and ERK pathways and reducing stem cell characteristics.

C1 [Wang, Panpan; Song, Dan; Yang, Li; Cai, Yu; Zhang, Ronghua] Jinan Univ, Coll Pharm, Guangzhou, Peoples R China.

[Wang, Panpan; Han, Li; Zhu, Xiaofeng] Jinan Univ, Affiliated Hosp 1, Guangzhou, Peoples R China.

[Wan, Danhong; Li, Lingyu; Mei, Wenhui; Li, Xiaoyun] Jinan Univ, Coll Tradit Chinese Med, Guangzhou, Peoples R China.

[Cai, Yu; Zhang, Ronghua] Jinan Univ, Canc Res Inst, Guangzhou, Peoples R China.

C3 Jinan University; Jinan University; Jinan University; Jinan University

RP Cai, Y; Zhang, RH (通讯作者)，Jinan Univ, Coll Pharm, Guangzhou, Peoples R China.; Cai, Y; Zhang, RH (通讯作者)，Jinan Univ, Canc Res Inst, Guangzhou, Peoples R China.

EM tcaiyu@jnu.edu.cn; tzrh@jnu.edu.cn

FU National Natural Science Foundation of China [81603342]; Administration

of Traditional Chinese Medicine of Guangdong Province [20171074]

FX This work was supported by the National Natural Science Foundation of

China (No. 81603342) and the Administration of Traditional Chinese

Medicine of Guangdong Province (No. 20171074). The funders had no role

in study design, data collection and analysis, decision to publish, or

preparation of the manuscript.

CR Acharyya S, 2012, CELL, V150, P165, DOI 10.1016/j.cell.2012.04.042

Aung TN, 2017, INT J MOL SCI, V18, DOI 10.3390/ijms18030656

Baig S, 2016, CELL DEATH DIS, V7, DOI 10.1038/cddis.2015.275

Baud V, 2009, NAT REV DRUG DISCOV, V8, P33, DOI 10.1038/nrd2781

Bousquet G, 2017, ONCOTARGET, V8, P35205, DOI 10.18632/oncotarget.16925

Campbell KJ, 2018, OPEN BIOL, V8, DOI 10.1098/rsob.180002

Campbell KJ, 2018, CELL DEATH DIS, V9, DOI 10.1038/s41419-017-0035-2

Cancemi Patrizia, 2018, Oncotarget, V9, P29064, DOI 10.18632/oncotarget.25561

Chaturvedi MM, 2011, ONCOGENE, V30, P1615, DOI 10.1038/onc.2010.566

Chen HY, 2014, AM J CANCER RES, V4, P89

Christian F, 2016, CELLS-BASEL, V5, DOI 10.3390/cells5010012

Denkert C, 2017, LANCET, V389, P2430, DOI 10.1016/S0140-6736(16)32454-0

Dey P, 2019, BREAST CANCER-TARGET, V11, P115, DOI 10.2147/BCTT.S189224

Foulkes WD, 2010, NEW ENGL J MED, V363, P1938, DOI 10.1056/NEJMra1001389

Goh JY, 2017, NAT MED, V23, P1319, DOI 10.1038/nm.4405

Hartman ZC, 2013, CANCER RES, V73, P3470, DOI 10.1158/0008-5472.CAN-12-4524-T

Hata AN, 2015, CANCER DISCOV, V5, P475, DOI 10.1158/2159-8290.CD-15-0011
[truncated: 1,146,144 more chars]
